# Integrated bioinformatics and machine learning for constructing a diagnostic model of major depressive disorder leveraging shared signatures from hemodialysis: A cross-sectional study

**DOI:** 10.1097/MD.0000000000049113

**Published:** 2026-06-05

**Authors:** Meng Zheng, Pingli Huang, Rongrong Wu, Huihui Wang, Ping Xu, Xuejing Zheng

**Affiliations:** a Hemodialysis Center, The Third Affiliated People’s Hospital, Fujian University of Traditional Chinese Medicine, Fuzhou, Fujian, China.

**Keywords:** Biomarker, Diagnostic model, Hemodialysis, Immune cells infiltration, Machine learning, Major depressive disorder

## Abstract

Major depressive disorder (MDD) is a highly prevalent and debilitating condition in patients undergoing long-term hemodialysis (HD), severely impairing quality of life and imposing a substantial economic burden. Despite the clinical significance of this frequent co-occurrence, the shared molecular mechanisms linking HD and MDD remain poorly understood. Multiple gene expression omnibus transcriptomic datasets were integrated and preprocessed through standardized workflows to ensure comparability. Differentially expressed genes (DEGs) were identified separately for HD and MDD cohorts. Shared DEGs were subjected to functional enrichment analysis. A diagnostic model for MDD was constructed using machine learning algorithms based on the identified core genes. The model’s performance was evaluated across the training cohort, external validation cohort, and subgroups. Additionally, immune cell infiltration patterns were analyzed, and associations between core genes and immune cells were assessed. Micro ribonucleic acid analysis identified potential upstream regulators of the core genes. A total of 34 DEGs were identified, which were significantly enriched in immune and inflammatory pathways. Using Lasso regression and gradient boosting algorithms, we identified 6 core genes (microsomal glutathione S-transferase 1 [MGST1], BAF chromatin remodeling complex subunit BCL7A [BCL7A], carnitine O-acetyltransferase [CRAT], fucosyltransferase 8 [FUT8], MAF BZIP transcription factor G [MAFG] and SDAD1 ribosome assembly factor [SDAD1]). In this exploratory analysis, the diagnostic model based on these genes exhibited consistent discriminative performance across different validation cohorts (area under the curve: 0.858–0.891). Immune infiltration analysis revealed significant alterations in immune phenotypes, particularly in monocytes and γδ T cells. Correlation analysis identified specific gene-immune associations. Furthermore, hsa-let-7b-5p was identified as a key upstream micro ribonucleic acid, which was upregulated in patients with MDD and exhibited predictive regulatory relationships with the core genes. This study established a molecular framework for MDD by integrating bioinformatics and machine learning, centered on the shared immune dysregulation and key genes between HD and MDD.

## 1. Introduction

Major depressive disorder (MDD) is a common complication in patients undergoing hemodialysis (HD), seriously affecting the quality of life of patients and the healthcare costs associated with chronic kidney disease (CKD).^[1,2]^ More than 300 million people worldwide are affected by major depression,^[3]^ which exacerbates the challenges patients undergoing HD face in managing their overall health, leading to increased morbidity and mortality.^[1,4]^ The complex interplay between the physical burden of end-stage renal disease and the psychological toll of depression is further compounded by well-documented health inequalities across sociodemographic groups,^[5]^ which influence both disease risk and outcomes. This multifaceted complexity underscores the critical need to elucidate the intrinsic biological links underlying the frequent co-occurrence of these conditions.

Currently, the molecular basis of MDD remains unclear,^[6]^ especially in the context of CKD. Existing diagnostic models rely heavily on patient self-assessment scales and physician subjectivity,^[7,8]^ which can result in delayed MDD diagnosis, thus impeding the timely initiation of therapeutic interventions that can improve patient prognosis.^[3,8]^ Current treatment protocols focus on medication and psychotherapy, often with poor efficacy, poor patient compliance, and notable side effects.^[9,10]^ To develop more effective means of early identification and intervention programs, it is particularly important to identify specific biomarkers involved in MDD in patients undergoing HD and to understand their biological pathways.

This study primarily aimed to identify the shared molecular mechanisms and biomarkers linking HD and MDD. To this end, we employed an integrated bioinformatics approach encompassing weighted gene co-expression network analysis, differential gene expression analysis, machine learning, and enrichment analysis to uncover common pathogenic pathways. Subsequently, we constructed and validated a machine learning model based on these shared signatures to distinguish MDD from controls. The identified core genes and pathways are proposed as candidate mechanisms underlying the comorbidity, which may inform the future development of targeted screening or therapeutic strategies for MDD in the HD population. Previous research^[11,12]^ has suggested that immune system dysregulation may play a critical role in the development of depressive symptoms. Existing immune cell assays have not shown sufficient sensitivity and specificity for clinical application.^[13–15]^ Therefore, by integrating multisource omics data and computational analytical techniques, we aimed to explore the underlying mechanisms linking HD and MDD through immune responses and cellular signaling pathways, thereby identifying core genes associated with this comorbidity. This study provides new insights into the complex interactions between immune responses and depressive symptoms.

## 2. Methods

### 2.1. Data collection

In this retrospective analysis, gene expression datasets were obtained by searching the National Center for Biotechnology Information gene expression omnibus database using the keywords “hemodialysis” and “major depressive disorder.” The HD cohort data originated from the GSE37171 dataset, from which samples that did not undergo standard HD were excluded, resulting in a final set of 32 patients and 40 healthy controls.

For the analysis of MDD, multiple cohorts were utilized. The GSE98793 dataset, comprising 64 patients and 64 controls, served as the training cohort following the exclusion of patients with comorbid anxiety disorders. Two independent datasets, GSE76826 and GSE52790, were employed for external validation. The GSE76826 dataset included 10 patients and 12 controls after further excluding patients in remission, while the GSE52790 dataset included 10 patients and 12 controls. The supplemental dataset GSE251778 comprised 80 MDD patients and 89 controls, while GSE251779 included 69 MDD patients and 85 controls, specifically designated for subsequent micro ribonucleic acid (RNA) validation. Detailed sample sizes, demographic characteristics, and platform information for all datasets are summarized in Table 1.

**Table 1:** Basic information on the GEO dataset.

Dataset	Sample	Platform	Attribute	Sex		Age	
				Disease group	Control group	Disease group	Control group
GSE37171	32 HD patients and 40 normal controls	GPL570	Training set	12 females 20 males	16 females 24 males	46.81 ± 13.11	42.45 ± 11.07
GSE98793	64 MDD patients and 64 normal controls	GPL570	Training set	48 females 16 males	48 females 16 males	51.59 ± 12.12	50.94 ± 11.96
GSE76826	10 MDD patients and 12 normal controls	GPL17077	Validation set	6 females 4 males	7 females 5 males	69.3 ± 12.85	61.75 ± 10.27
GSE52790	10 MDD patients and 12 normal controls	GPL17976	Validation set	6 females 6 males	5 females 5 males	41.42 ± 9.72	43.17 ± 8.66
GSE251778	80 MDD patients and 89 normal controls	GPL20301 GPL24676	Validation set	50 females 30 males	55 females 34 males	-	-
GSE251779	69 MDD patients and 85 normal controls	GPL18573	Validation set	39 females 30 males	50 females 35 males	-	-

GEO = gene expression omnibus, HD = hemodialysis, MDD = major depressive disorder.

### 2.2. Data preprocessing and normalization

To ensure model transportability across different profiling platforms, we implemented a consistent cross-platform preprocessing strategy. All datasets underwent independent but identical steps: background correction, followed by quantile normalization within each dataset. The normalized data were log_2_-transformed to stabilize variance, and gene identifiers were uniformly converted to official gene symbols using platform annotation files. To assess normalization effectiveness, we generated comparative boxplots and principal component analysis plots for the key datasets (GSE37171 and GSE98793) before and after processing.

### 2.3. Identification of differentially expressed genes (DEGs)

We performed differential expression analysis on the HD (GSE37171) and MDD (GSE98793) datasets using the “limma” package in R software (R Foundation for Statistical Computing). To rigorously control false positives and ensure fair cross-dataset comparisons, we adopted fully unified statistical criteria. First, we performed multiple testing correction on *P* values using the Benjamini–Hochberg method, controlling the false discovery rate to < 0.1. Second, we uniformly set the threshold for differentially expressed effect values to |log_2_FC| > 0.2. The selection of false discovery rate < 0.1 balances statistical rigor with exploratory research needs, while the unified |log_2_FC| > 0.2 threshold aims to capture the subtle transcriptomic signals commonly observed in psychiatric disorders. Analysis results were visualized using volcano plots to display overall distribution. Additionally, we generated a gene ranking scatter plot and a heatmap of the top 30 DEGs. To select candidate genes for subsequent modeling, we took the intersection of the DEG sets from both datasets and visualized it using a Venn diagram.

### 2.4. Enrichment analysis of intersecting genes

To explore the relationships between the intersecting genes, a protein-protein interaction network was constructed and visualized using the GeneMANIA (http://genemania.org/) website. For gene set functional enrichment analysis, we used gene ontology annotations of the genes in the “org.Hs.e.g..db package” in R. The KEGG RESTAPI (https://www.kegg.jp/kegg/rest/keggapi.html) was used to obtain the latest Kyoto Encyclopedia of Genes and Genomes (KEGG) pathways. At the same time, we used the KEGG REST API to obtain the latest gene annotations of the KEGG pathway, and finally used the R “clusterProfiler” package to perform enrichment analysis. The minimum gene set was set to 5, and the maximum gene set was set to 5000 (*P* < .05).

### 2.5. Machine learning algorithms and model evaluation

The GSE98793 dataset (n = 128) served as the training set, while the GSE76826 (n = 22) and GSE52790 (n = 22) datasets were reserved as independent external validation sets. To eliminate batch effects across studies and ensure feature comparability, the pooled training data underwent *Z*-score normalization after Combat correction. The mean and standard deviation required for normalization were calculated using only the training set data, and these identical parameters were then applied to all external validation sets. To screen for the optimal predictive model, we constructed a screening framework comprising 130 candidate models. This was achieved through pairwise combinations of 12 machine learning algorithms (Table S1). Model training and hyperparameter optimization were performed via grid search on the training set, with internal validation using *k*-fold cross-validation (*k* = 5 or 10) to prevent overfitting and determine optimal parameters. Model performance was evaluated using the area under the curve (AUC) metric. AUC values across all model combinations were visualized and compared via heatmaps, with the model yielding the highest AUC selected as the final model. We comprehensively evaluated the predictive accuracy and potential clinical research value of the selected model through calibration curves, decision curve analysis, and clinical impact curves, supplemented by subgroup analyses. The final model was locked after training and was not updated or retrained using validation data to ensure an unbiased estimate of its generalization performance.

### 2.6. Single-gene enrichment analysis of core genes

We acquired the gene set enrichment analysis software (version 3.0) from the gene set enrichment analysis website, classified the samples into high (≥ 50%) and low-expression (< 50%) groups according to the expression level of each gene, and extracted c2.cp.kegg.v7.4 symbols from the molecular characterization database. This gene matrix transposed subset was utilized to examine the pertinent pathways and molecular mechanisms based on gene expression profiles and phenotypic classification, with a minimum gene set of 5 and a maximum gene set of 5000 genes, alongside 1000 resamplings (*P* < .05).

### 2.7. Immunological infiltration assessment

To characterize and compare the immune microenvironments across different pathologies, we performed immune cell deconvolution analysis using the cell-type identification by estimating relative subsets of RNA transcripts (CIBERSORT) algorithm on transcriptomic datasets from HD (GSE37171) and MDD (GSE98793). Gene expression matrices from both cohorts were imported into R software and normalized using the normalize Between Arrays function from the limma package to ensure no negative values or missing data. Subsequently, CIBERSORT analyzed the normalized expression data, using the leukocyte signature matrix 22 signature matrix as a reference benchmark to estimate the relative proportions of 22 immune cell types. To ensure decomposition reliability, only samples yielding significant results (*P* < .05) in CIBERSORT output were retained for subsequent analysis. Box plots visually represented comparative differences in immune cell infiltration between the HD and MDD groups. Furthermore, to explore associations between the immune landscape and common molecular features of identified diseases, we assessed correlations between estimated immune cell proportions and expression levels of relevant feature genes, presenting results via heatmaps.

### 2.8. Model gene-related miRNA prediction and validation

The miRNet database (https://www.mirnet.ca/miRNet/home.xhtml) was used to predict miRNAs potentially targeting the core genes. The interaction network was visualized and analyzed using Cytoscape software (version 3.10.1) (). Subsequently, to explore the potential regulatory relationships between core genes and miRNAs, we performed an integrative in silico analysis of the GSE251778 and GSE251779 datasets. Since these 2 datasets are derived from the same study cohort and share identical sample identifiers, we were able to precisely pair the messenger RNA and miRNA expression data from the same individual. The differential expression of the predicted miRNAs was assessed using the GSE251779 dataset. Finally, Spearman correlation analysis was conducted to examine the relationships between the expression levels of these candidate miRNAs and the 6 core genes using the matched GSE251778 dataset.

### 2.9. Statistical analysis

Data were analyzed using R software (v4.2.2). Differences between groups were compared using an unpaired *t*-test, with *P* < .05 considered statistically significant. Diagnostic accuracy was assessed by AUC, sensitivity, and specificity, with 95% confidence intervals (CIs) calculated using the DeLong method to provide mathematically bounded estimates that respect the logical 0 to 1 range.

## 3. Results

### 3.1. Data collection

**Figure 1.**
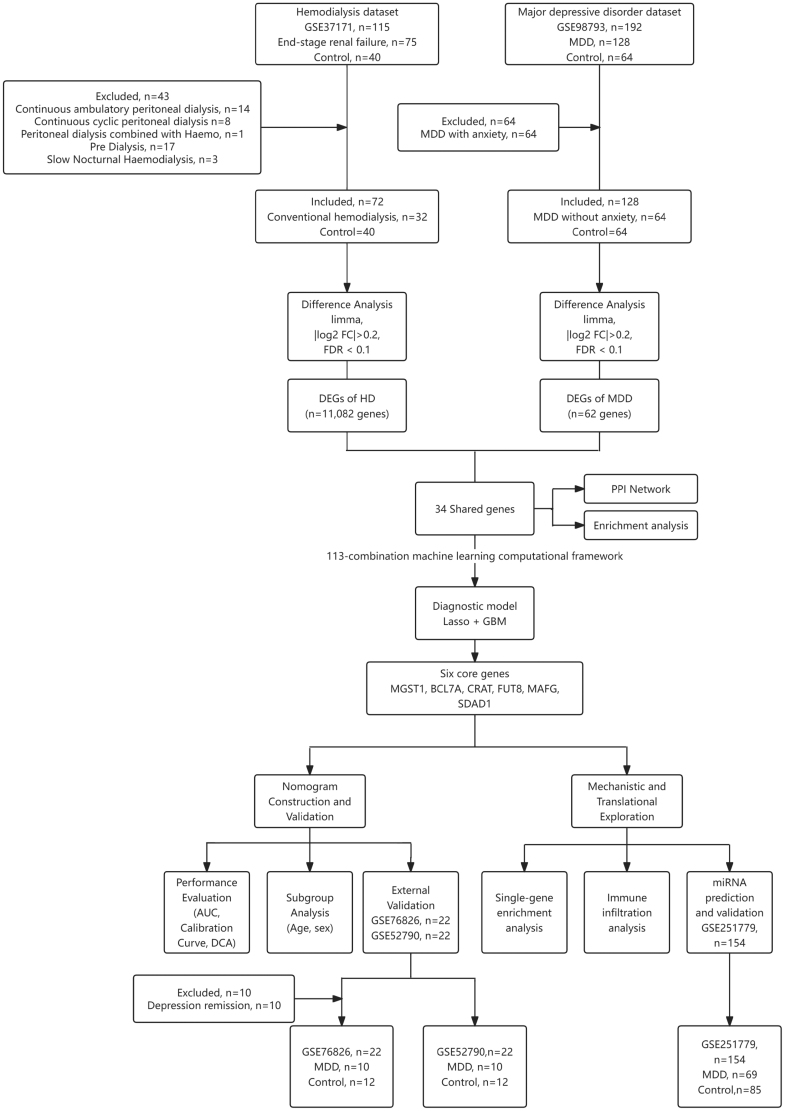
Main flowchart of the study. DEG = differentially expressed genes, HD = hemodialysis, MDD = major depressive disorder, MHC = major histocompatibility complex, n = number of study, PPI = protein-protein interaction.

**Figure 2.**
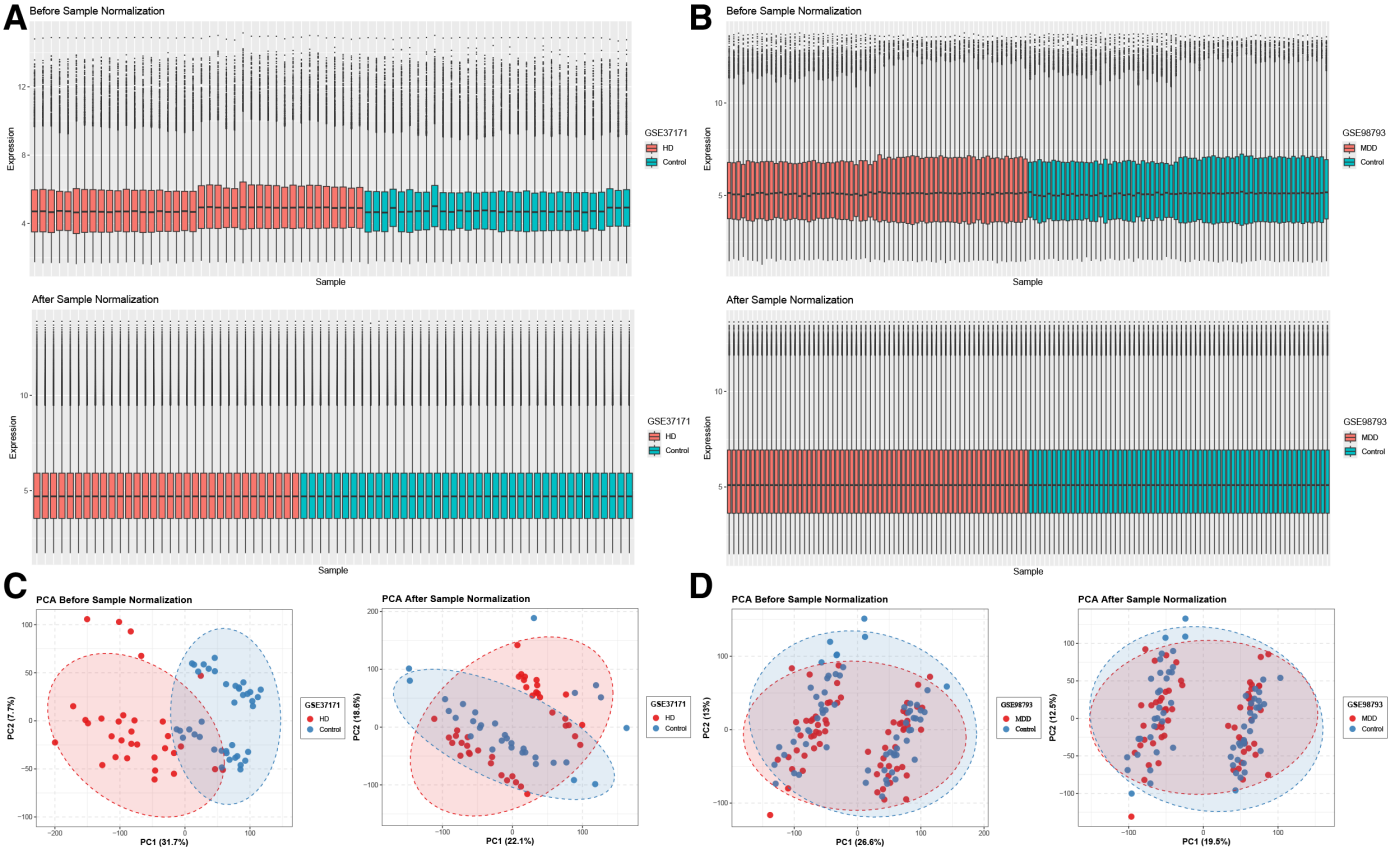
Normalization of HD Dataset and MDD Dataset. (A) Boxplots before and after normalization of the GSE37171 dataset. (B) Boxplots before and after normalization of the GSE98793 dataset. (C) PCA of the GSE37171 dataset before and after normalization. (D) PCA of the GSE98793 dataset before and after normalization. HD = hemodialysis, MDD = major depressive disorder, PC = principal component, PCA = principal component analysis.

The workflow of this study is illustrated in Figure 1. Following the application of a standardized preprocessing workflow, the expression data distribution was effectively normalized. As shown in Figures 2A–2D, comparative box plots and principal component analysis plots of the GSE37171 and GSE98793 datasets demonstrate that quantile normalization successfully reduced technical variability, thereby validating the effectiveness of the preprocessing method.

### 3.2. Identification of DEGs associated with HD and MDD

**Figure 3.**
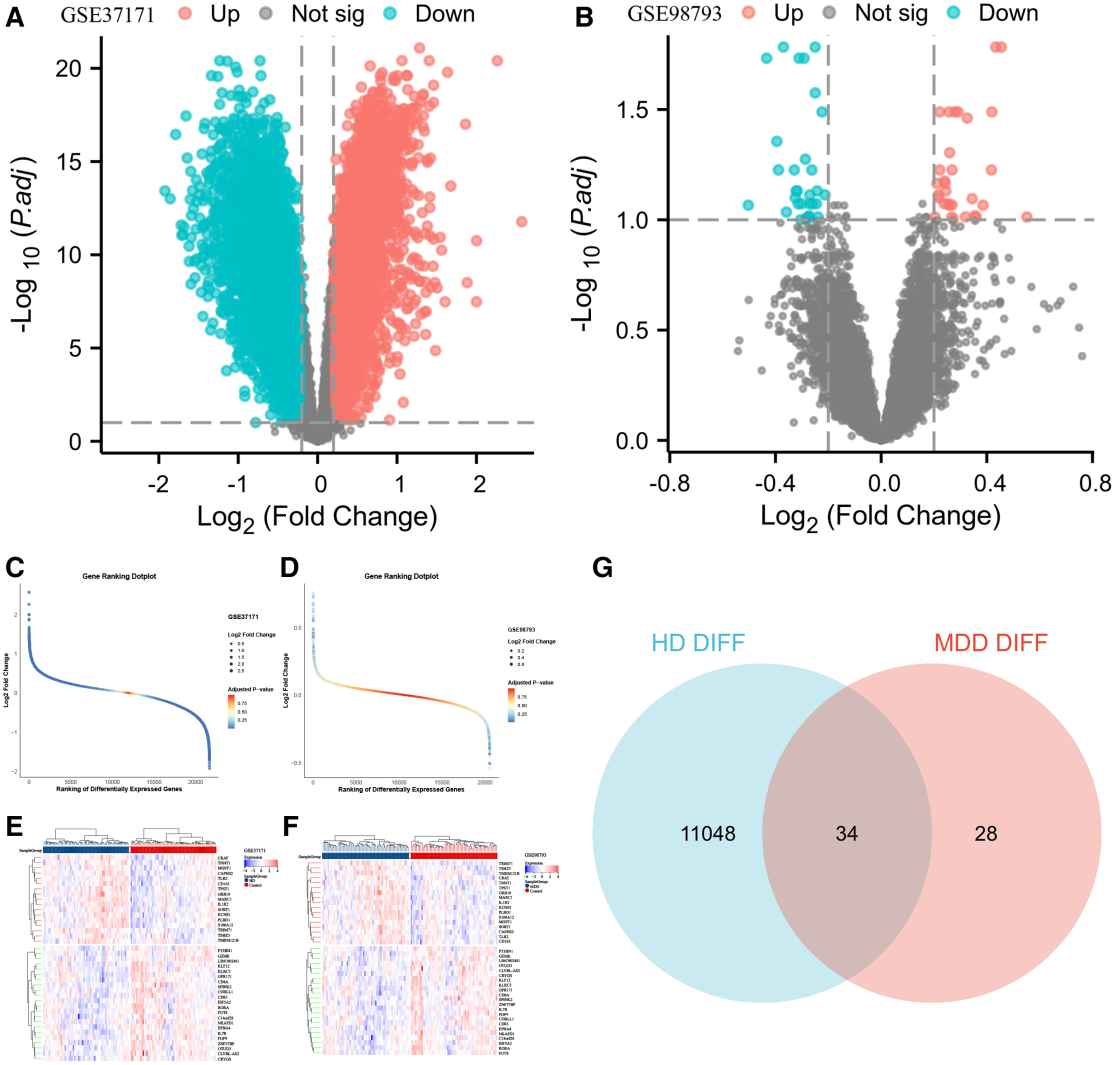
Screening of shared signature genes associated with HD and MDD. (A) Volcano plot of DEGs in the HD dataset. (B) Volcano plot of DEGs in the MDD dataset. (C) Gene ranking dot plot for the HD dataset. (D) Gene ranking dot plot for the MDD dataset. (E) Heatmap showing the expression of the top 30 DEGs in the HD dataset. (F) Heatmap showing the expression of the top 30 DEGs in the MDD dataset. (G) Venn diagram of common genes in HD and major depression. DEG = differentially expressed genes, HD = hemodialysis, MDD = major depressive disorder.

To determine the association between HD and MDD, the HD dataset GSE37171 identified a total of 11,082 DEGs, comprising 5736 upregulated genes and 5346 downregulated genes (Fig. 3A). The MDD dataset GSE98793 yielded 62 DEGs, including 31 upregulated genes and 31 downregulated genes (Fig. 3B). Figures 3C and 3D visually present a combined ranking of gene effect magnitude and statistical significance across both datasets, where dot size represents |log_2_FC| values and color denotes −log10 (adjusted *P* value). Figures 3E and 3F highlight the 30 most significantly upregulated and 30 most significantly downregulated DEGs across both datasets. Further intersection analysis of the 2 gene sets identified 34 common genes for subsequent analysis (Fig. 3G, Table S2).

### 3.3. Functional enrichment analysis of shared signature genes between HD and MDD

**Figure 4.**
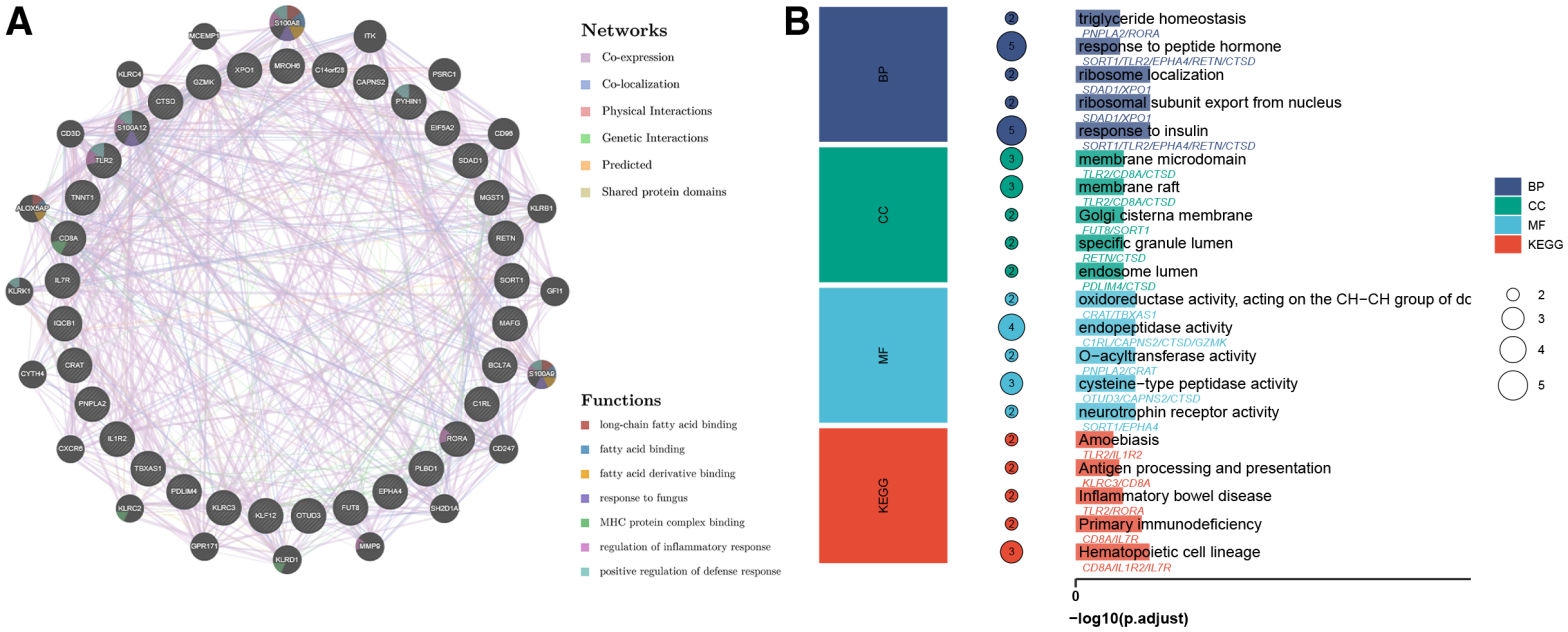
PPI network and enrichment analyses of shared signature genes between HD and MDD. (A) PPI network of the overlapping DEGs identified from independent HD and MDD cohorts. (B) Bar chart showing GO and KEGG pathway enrichment analysis for biological processes, cellular components, and molecular functions. BP = biological processes, CC = cellular components, DEG = differentially expressed genes, HD = hemodialysis, KEGG = Kyoto Encyclopedia of Genes and Genomes, MDD = major depressive disorder, MF = molecular functions, MHC = major histocompatibility complex, PPI = protein-protein interaction.

We constructed a protein-protein interaction network of the shared signature genes using the GeneMANIA database (Fig. 4A) and analyzed the gene ontology and KEGG functional enrichment of these genes using the R package “clusterProfiler” (Fig. 4B). Gene ontology enrichment analysis revealed that the biological processes were significantly overrepresented in response to insulin, ribosomal subunit export from the nucleus, ribosome localization, response to peptide hormone, and triglyceride homeostasis. For cellular components, the genes were notably enriched in the endosome lumen, specific granule lumen, golgi cisterna membrane, membrane raft, and membrane microdomain. The overrepresented molecular functions comprised neurotrophin receptor activity, cysteine-type peptidase activity, O-acyltransferase activity, endopeptidase activity, and oxidoreductase activity acting on the CH-CH group of donors (Table S3). KEGG pathway analysis further indicated significant enrichment in pathways related to immune regulation and inflammation, specifically hematopoietic cell lineage, primary immunodeficiency, inflammatory bowel disease, antigen processing and presentation, and amoebiasis (Table S4).

### 3.4. A shared signature-based diagnostic model for MDD

**Figure 5.**
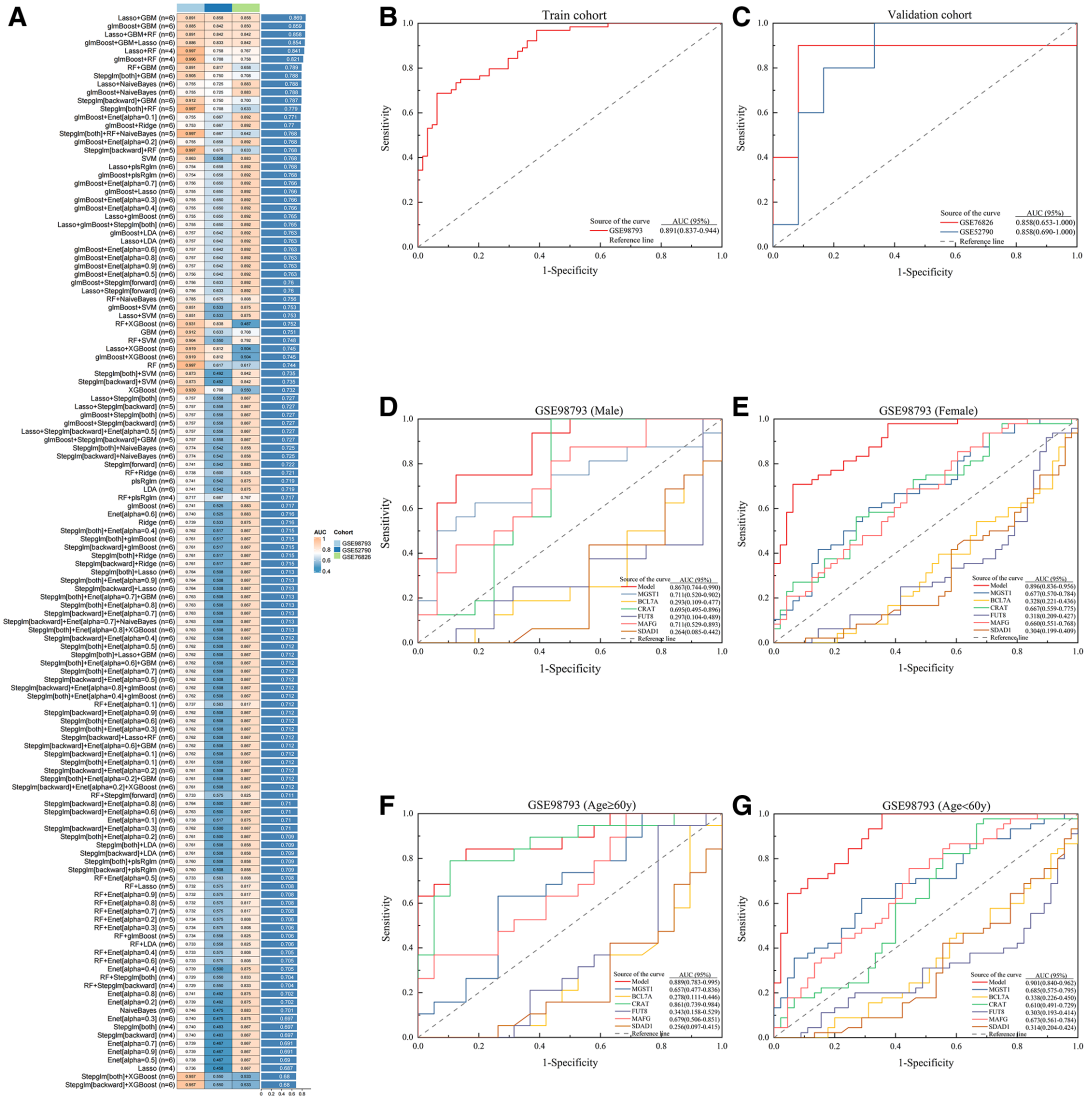
Diagnostic performance of the machine learning models. (A) A total of 130 combinations of machine learning algorithms were evaluated using 10-fold cross-validation. (B) ROC curves for the training cohort. (C) ROC curves for the validation cohort. ROC curves for the model and individual shared signature genes in the (D) male, (E) female, (F) aged ≥ 60 years, and (G) aged < 60 years subgroups. AUC = area under the curve, ROC = receiver operating characteristic.

To construct an MDD diagnostic model based on molecular features shared with HD, we systematically evaluated 130 machine learning algorithms. The model combining least absolute shrinkage and selection operator-based feature selection and gradient boosting machine demonstrated optimal performance in the MDD training cohort (GSE98793), achieving an AUC of 0.891 (95% CI: 0.837–0.944) (Fig. 5A; Table S5). This diagnostic efficacy was further evaluated in 2 independent MDD cohorts: GSE52790 (AUC: 0.858, 95% CI: 0.690–1.000) and GSE76826 (AUC: 0.858, 95% CI: 0.653–1.000) (Figs. 5B and 5C). Furthermore, the model maintained high performance across gender and age subgroups within the training cohort (AUC range: 0.867–0.901), outperforming the diagnostic efficacy of any individual core gene (Figs. 5D–5G).

**Figure 6.**
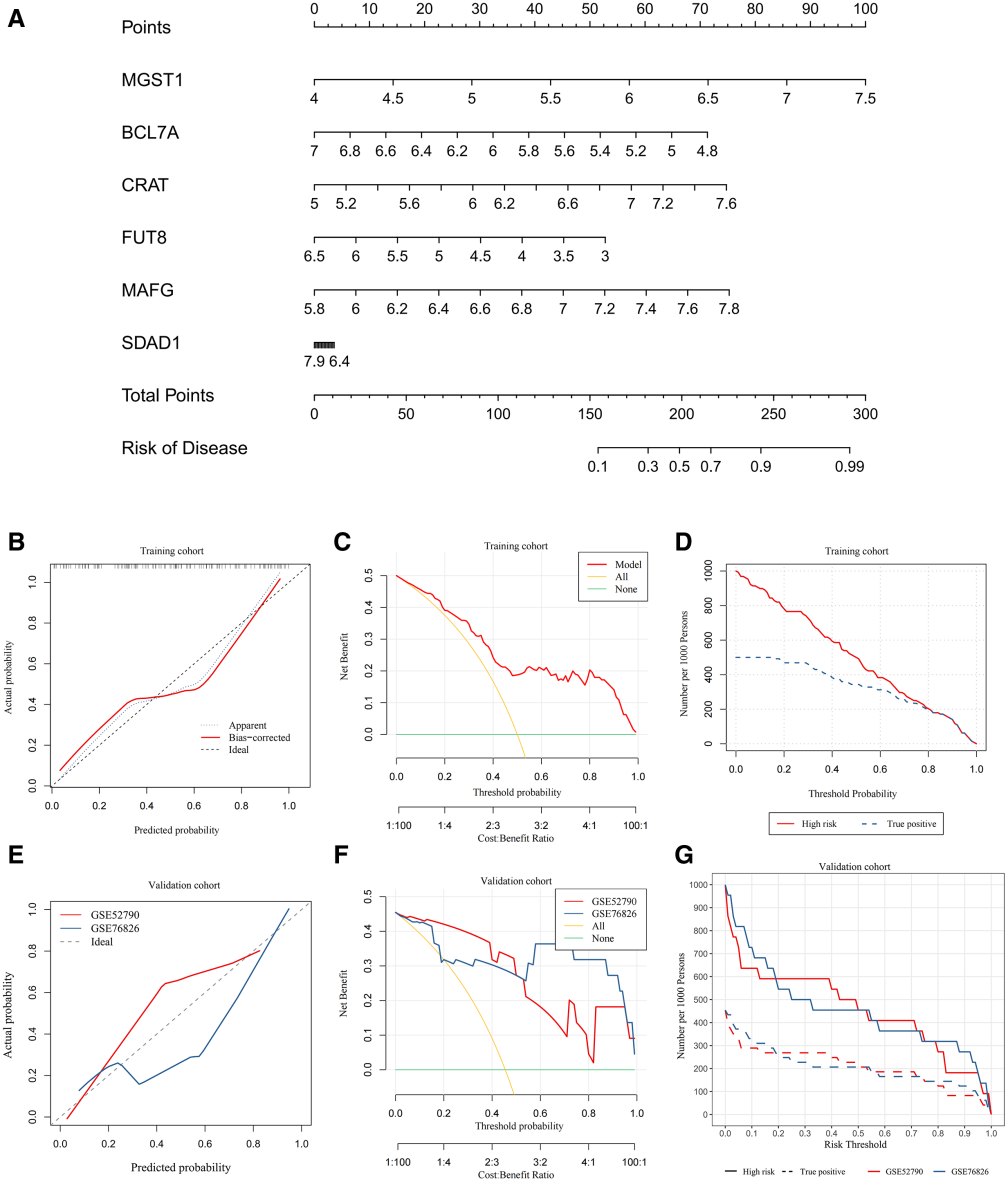
Construction and validation of a nomogram based on core shared signature genes. (A) Nomogram constructed based on 6 core shared signature genes (the 6 model genes). (B–D) Calibration, DCA, and CIC curves for the training cohort. (E–G) Calibration, DCA, and CIC curves for the validation cohort. CIC = clinical impact curve, DCA = decision curve analysis.

The final model incorporated 6 core genes (MGST1, BCL7A, CRAT, FUT8, MAFG, and SDAD1). Based on this, we constructed a visual bar chart of the model output (Fig. 6A). In the training cohort, calibration curves demonstrated high consistency between the model’s predicted probabilities and actual observed outcomes (Fig. 6B). Decision curve analysis and clinical impact curves indicated that applying this nomogram could potentially yield a net benefit for future clinical decision-making strategies (Figs. 6C and 6D). The model’s discriminative power, calibration, and potential clinical utility were consistently observed in 2 external MDD validation cohorts (Figs. 6E–6G).

### 3.5. Single-gene enrichment analysis of model genes

**Figure 7.**
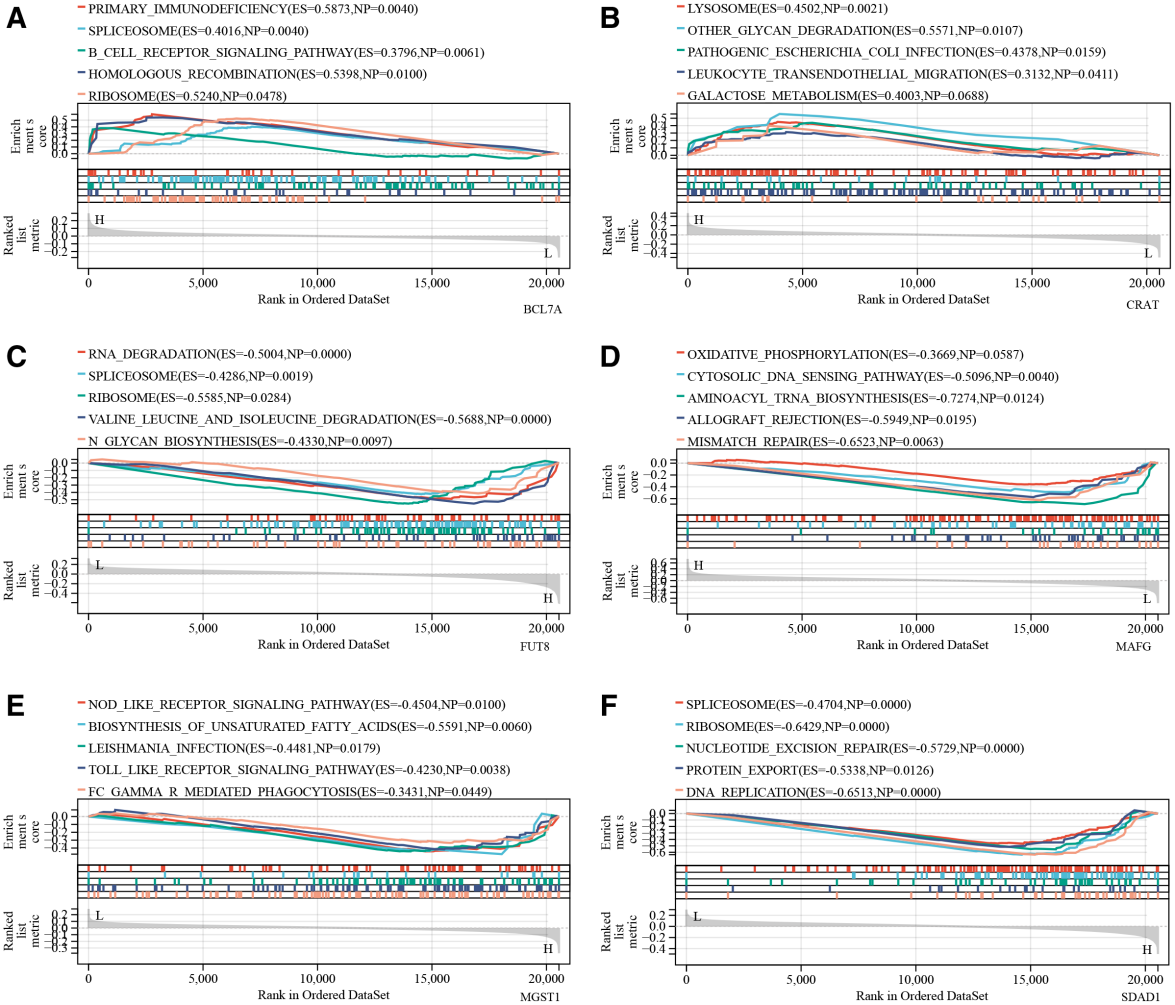
GSEA of core shared signature genes between HD and MDD. GSEA for (A) BCL7A, (B) CRAT, (C) FUT8, (D) MAFG, (E) MGST1, and (F) SDAD1. BCL7A = BAF chromatin remodeling complex subunit, CRAT =carnitine O-acetyltransferase, FUT8 = fucosyltransferase 8, GSEA = gene set enrichment analysis, HD = hemodialysis, MAFG = MAF BZIP transcription factor G, MDD = major depressive disorder, MGST1 = microsomal glutathione S-transferase 1, SDAD1 = SDAD1 ribosome assembly factor.

Single-gene enrichment analysis was performed for the 6 model genes (Figs. 7A–7F). The results showed that BCL7A, FUT8, CRAT, MGST1, SDAD1, and MAFG were significantly enriched in 22, 18, 14, 15, 22, and 15 pathways, respectively. Several pathways were co‑enriched across multiple genes: nucleotide excision repair, valine‑leucine‑and‑isoleucine degradation, and DNA replication were shared by at least 4 genes; spliceosome, ribosome, peroxisome, RNA degradation, and aminoacyl‑tRNA biosynthesis were shared by at least 3 genes (Table S6).

### 3.6. Analysis of immune cell infiltration

**Figure 8.**
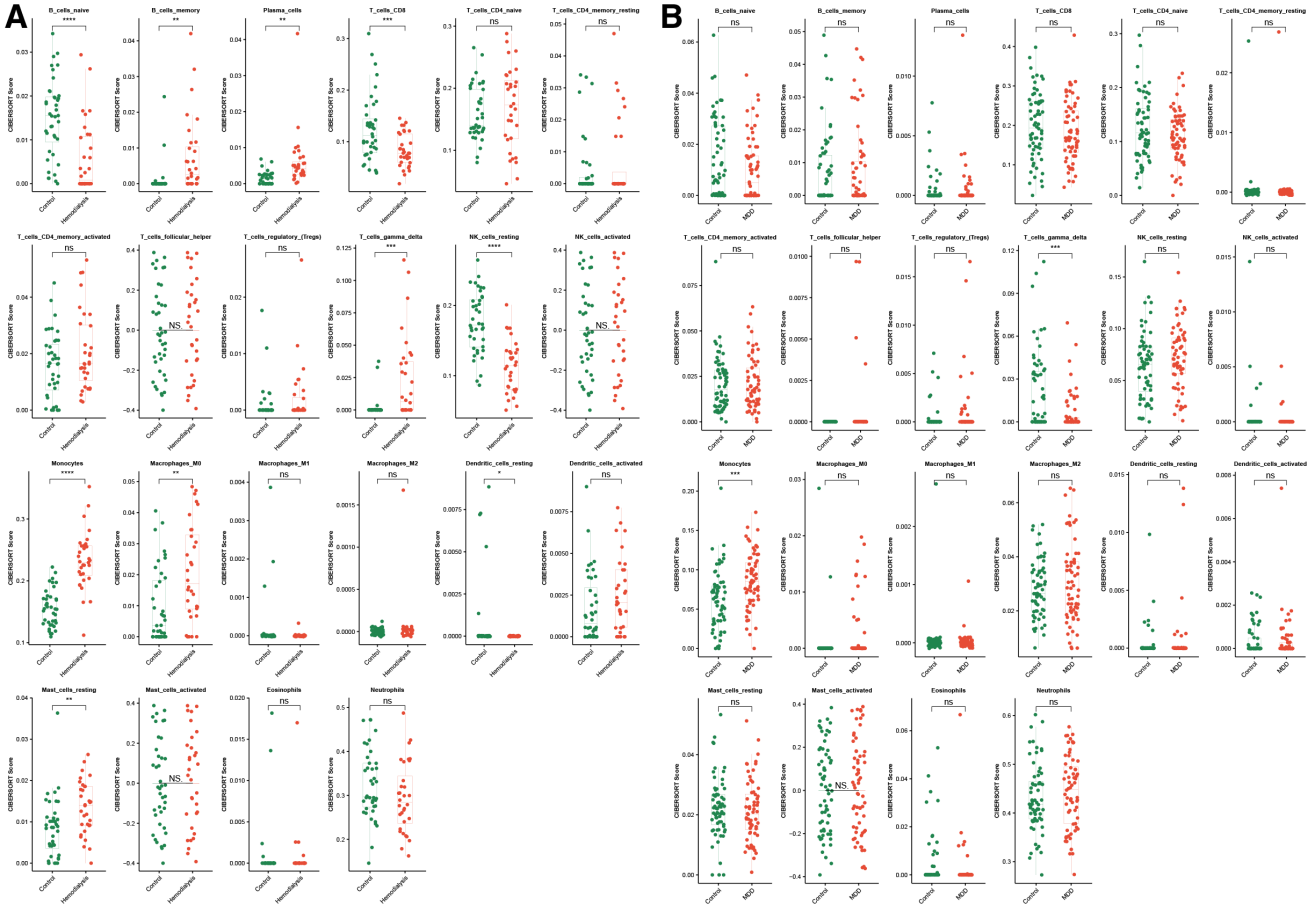
Immune infiltration analysis in the HD and major depressive disorder groups. (A) Visualization of CIBERSORT scores of immune cells in the HD and control groups, with each panel representing a different immune cell subtype. (B) Visualization of CIBERSORT scores of immune cells in the MDD and control groups, with each panel representing a different immune cell subtype. *P < .05, **P < .01, ***P < .001. CIBERSORT = cell-type identification by estimating relative subsets of RNA transcripts, HD = hemodialysis, MDD = major depressive disorder,

Using CIBERSORT, we performed compositional feature analysis of 22 immune cell subsets in patients undergoing HD, patients with depression, and healthy controls. The analysis revealed significant immunophenotypic differences: compared with the control group, patients undergoing HD exhibited significantly higher infiltration abundance of monocytes, memory B cells, plasma cells, γδ T cells, M0 macrophages, and resting mast cells. Conversely, the infiltration abundance of resting natural killer cells, naive B cells, CD8^+^ T cells, and resting dendritic cells was significantly lower (Fig. 8A). Compared with the control group, the infiltration abundance of monocytes was significantly increased, whereas that of γδ T cells was significantly decreased in patients with MDD (Fig. 8B).

**Figure 9.**
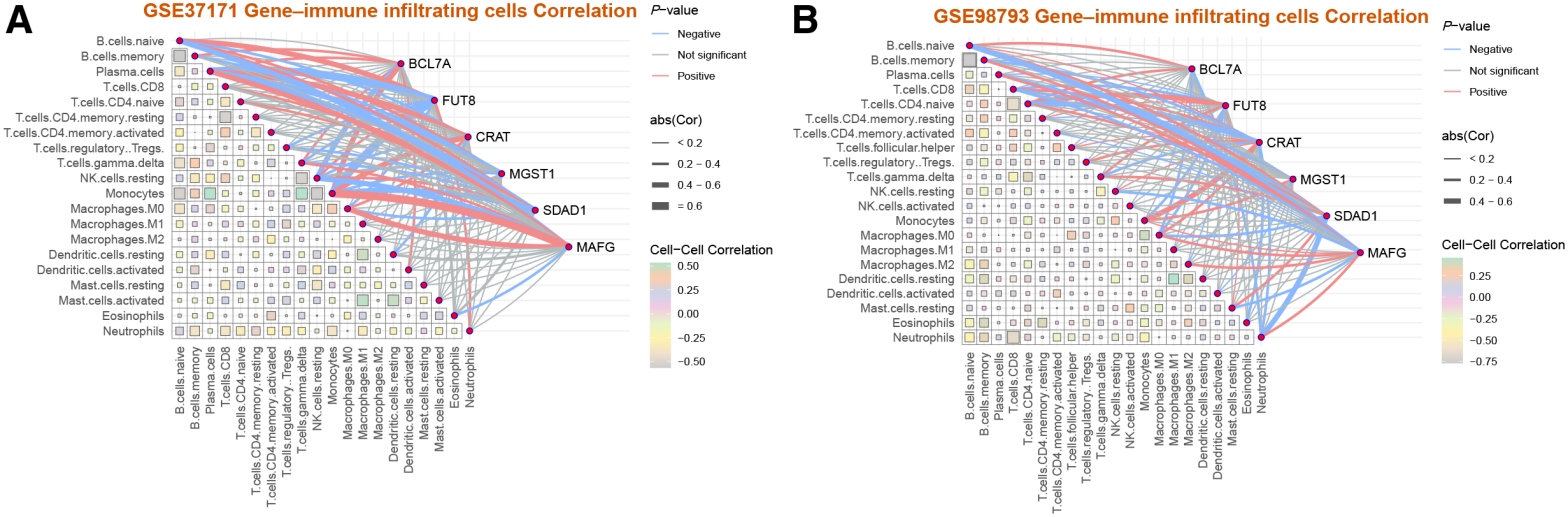
Heatmaps illustrate the association of shared signature genes with 22 immune cell types across different datasets. (A) Heatmap displays the association of shared signature genes with 22 immune cell types in the HD dataset. (B) Heatmap displays the association of shared signature genes with 22 immune cell types in the MDD dataset. HD = hemodialysis, MDD = major depressive disorder,

We further explored the relationship between these altered immune landscapes and the core HD-MDD shared genes. Correlation analysis revealed distinct gene-immune interaction patterns in each cohort. In the HD cohort, monocytes showed a strong negative correlation with FUT8 (*r* = −0.733, *P* < .001) and a strong positive correlation with MAFG (*r* = 0.628, *P* < .001), while resting natural killer cells were negatively correlated with BCL7A (*r* = −0.434, *P* < .001) and MAFG (*r* = −0.405, *P* < .001). In the MDD cohort, monocytes were positively correlated with CRAT (*r* = 0.437, *P* < .001), and γδ T cells were negatively correlated with CRAT (*r* = −0.375, *P* < .001). These specific correlations were visually summarized in a correlation heatmap (Figs. 9A and 9B). Table S7 details all immune cell subpopulations that were highly correlated with the core genes in each dataset (absolute value of the correlation coefficient > 0.50).

### 3.7. Predicting potential core miRNAs for model genes

**Figure 10.**
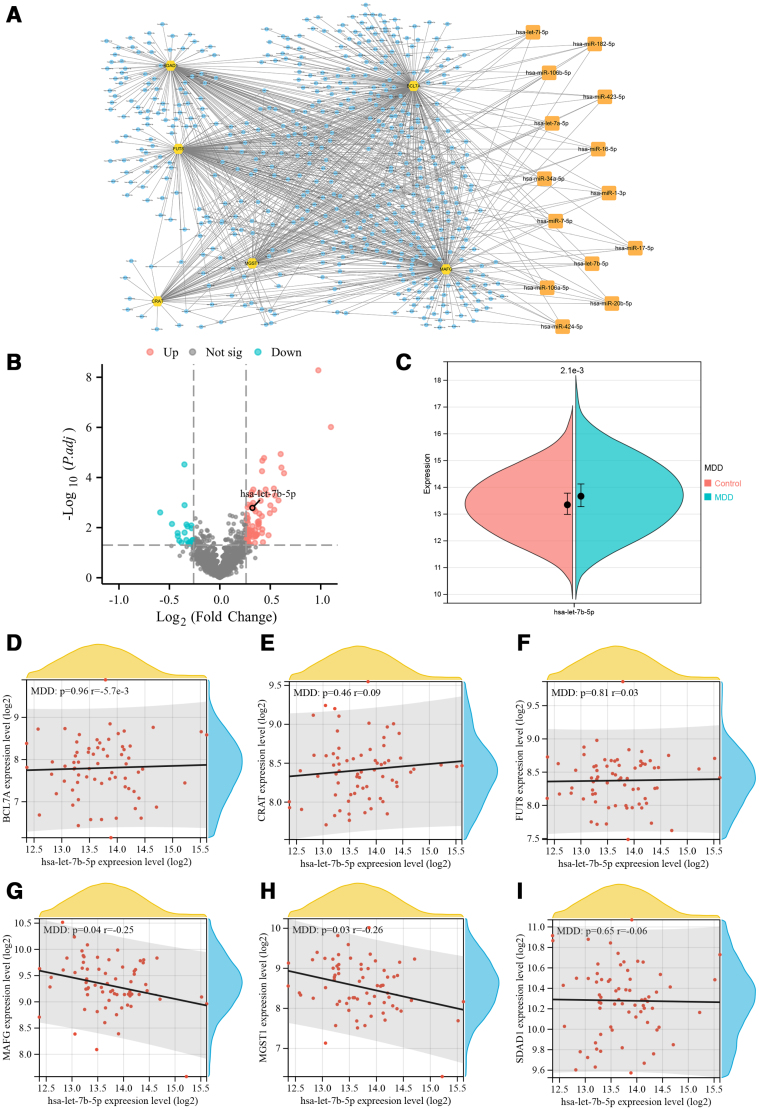
Screening and validation of potential miRNAs targeting core shared signature genes. (A) Interaction network between 6 core shared signature genes and potential miRNA targets. (B) Volcano plot of differentially expressed miRNAs between the MDD and control groups in GSE251779. (C) Violin plot of hsa-let-7b-5p expression levels in different groups in GSE251779. (D-I) Correlation between hsa-let-7b-5p expression levels and those of the 6 core shared signature genes in GSE251778 and GSE251779. HD = hemodialysis, MDD = major depressive disorder.

To investigate the posttranscriptional regulation of the 6 model genes, we computationally predicted their potential targeting MicroRNAs (miRNAs) using the miRNet database, which yielded 522 candidate miRNAs (Table S8). Among these, 14 miRNAs were predicted to target all 6 genes (Fig. 10A). To assess this prediction in an independent cohort, we analyzed an MDD miRNA dataset (GSE251779) and identified 180 differentially expressed miRNAs (Fig. 10B). Intersection with the 14 predicted miRNAs revealed hsa-let-7b-5p as the sole overlapping candidate. In this dataset, the expression of hsa-let-7b-5p was significantly higher in the MDD group compared to controls (*P* = .0021, Fig. 10C). Subsequently, Spearman correlation analysis was performed to explore the relationship between hsa-let-7b-5p expression and those of its predicted target genes, revealing negative correlations with MGST1 and MAFG (Figs. 10D–10I).

## 4. Discussion

The prevalence of MDD is particularly high among patients with CKD undergoing HD.^[1]^ Chronic illness and mental health create a complex interplay that can exacerbate the overall decline in the quality of life of patients and complicate treatment outcomes.^[16,17]^ Exploring the molecular mechanisms underlying the association between HD and MDD is critical for understanding their comorbidity and identifying potential therapeutic targets. Through integrating multicenter, cross-platform gene expression datasets, this study systematically analyzed the association between HD and MDD, identifying and validating shared molecular signatures and disrupted immune profiles linking HD and MDD. By intersecting the DEGs identified in independent HD and MDD transcriptomic datasets, we identified a core set of 34 genes commonly dysregulated in both conditions. This significant genetic overlap points to a convergent underlying biological pathway, corroborating prior observations of shared transcriptomic alterations in depression and chronic somatic illnesses.^[18–20]^

The 34 genes commonly dysregulated in both HD and MDD datasets were used as the initial set of candidate features. To construct a diagnostic model for MDD informed by these shared molecular features, we systematically evaluated combinations of machine learning algorithms. The model integrating least absolute shrinkage and selection operator for feature selection and gradient boosting machine for classification achieved an AUC of 0.891 in the training cohort (GSE98793). The model’s generalizability was further demonstrated in 2 independent MDD validation cohorts (GSE52790 and GSE76826), with consistent AUC values of 0.858. The final model incorporated 6 key genes: BCL7A, FUT8, CRAT, MGST1, SDAD1, and MAFG. This is consistent with previous research on the importance of machine learning methods in improving the accuracy of psychiatric diagnoses.^[21,22]^ Niemann et al^[23]^ showed that machine learning models can effectively predict the severity of depression based on clinical and demographic data, thereby facilitating timely intervention. In addition, the model we developed showed good diagnostic performance in subgroups but differed slightly in different age groups and across sexes, suggesting that demographic factors may influence the efficacy of the model. While our model showed stable performance across the cohorts, subgroup analyses indicated slight variations in diagnostic efficacy across different age and sex groups, consistent with the known influence of demographics on depressive symptom presentation.^[24,25]^ This suggests that while the model shows potential, its future development must account for these demographic factors to ensure equitable performance across diverse patient subgroups.

Functional enrichment analysis revealed that these shared characteristic genes exhibit coordinated dysregulation across multiple biological levels. At the level of BP, genes were significantly enriched in “response to insulin,” “response to peptide hormones,” and “triglyceride homeostasis.” This finding suggests that disturbances in metabolic and neuroendocrine signaling pathways may serve as a crucial bridge linking HD to MDD.^[26,27]^ Insulin resistance and metabolic syndrome have been demonstrated to be associated with depression;^[28,29]^ the shared disruption of these pathways may reflect a broad metabolic network impaired in both conditions. KEGG analysis revealed that genes were highly enriched in pathways including “Hematopoietic Cell Lineage,” “Primary Immunodeficiency,” and “Antigen Processing and Presentation.” These pathways are critical for immune system function, suggesting that immune dysregulation may serve as a key biological bridge linking HD and MDD. Subsequently, KEGG pathway analysis further localized these molecular functions to specific systems-level BP. Analysis revealed that genes were highly enriched in pathways such as “Hematopoietic Cell Lineage,” “Primary Immunodeficiency,” and “Antigen Processing and Presentation.” These pathways are critical for immune system function, indicating that immune dysregulation represents another key biological bridge connecting HD and MDD.^[30,31]^ Furthermore, the identification of pathways related to “inflammatory bowel disease” further underscores the role of chronic inflammatory states. Research on the gut-brain axis has demonstrated that intestinal inflammation can influence central nervous system inflammatory processes, thereby participating in mood regulation.^[32]^ The enrichment of this pathway may suggest that gastrointestinal health and systemic immune responses are interconnected factors warranting deeper exploration in the context of the HD-MDD association.^[33]^

The 6 core genes identified by our model are directly associated with key processes such as oxidative stress, immune regulation, and metabolism, providing a mechanistic hypothesis for the association between HD and MDD. MGST1 and MAFG both point to redox imbalance as a common pathological pathway. As a key detoxification enzyme, MGST1 expression may be associated with counteracting oxidative stress damage commonly observed in HD and MDD.^[34,35]^ The transcription factor MAFG regulates a series of oxidative stress response genes,^[36]^ whose function complements MGST1, jointly amplifying the potential dysregulation of oxidative stress responses in both disorders. In terms of immune regulation, the roles of BCL7A and FUT8 are particularly prominent. BCL7A participates in regulating apoptosis and proliferation and is closely associated with immune-inflammatory responses;^[37,38]^ its upregulation and correlation with immune cell infiltration patterns suggest it may play a role in immune cell dysfunction during chronic inflammatory states. FUT8-mediated protein glycosylation constitutes a critical modification in immune cell signaling pathways.^[39]^ Its dysregulation is associated with chronic inflammation,^[40]^ suggesting this gene may contribute to immune dysregulation linking the 2 diseases by affecting immune cell function. Additionally, enzymes encoded by CRAT are crucial for fatty acid metabolism,^[41,42]^ and alterations in their expression may serve as a molecular link connecting metabolic disorders to mood disorders. SDAD1 participates in RNA metabolism and viral responses,^[43,44]^ and its selection as a core gene suggests potential complex interactions between infection history, persistent immune activation, and both disease states.

Analysis of immune cell infiltration in HD patients and MDD patients revealed significant alterations in the immune landscape, highlighting the potential role of immune dysregulation in the comorbidity of these diseases. We found markedly elevated monocyte abundance in both patient groups, suggesting a shared inflammatory response mechanism that may contribute to the pathophysiology of HD and MDD. Monocytes play a pivotal role in inflammation mediation^[45]^ and are associated with multiple neuropsychiatric disorders.^[46–48]^ They may contribute to the development of depression by influencing neuroinflammatory processes and neuroplasticity.^[49,50]^ The significant negative correlation of monocytes with the FUT8 gene and the positive correlation with the MAFG gene in the HD cohort suggest these genes may influence the inflammatory environment in patients with HD by regulating monocyte activity. Compared to patients with HD, patients with MDD exhibited a reduced trend in γδ T cell infiltration. As a critical bridge linking innate and adaptive immunity,^[51]^ the diminished γδ T cell population may reflect impaired immune surveillance in the context of depression. This finding aligns with previous research suggesting γδ T cell involvement in neuroimmune inflammation.^[52,53]^ Therefore, γδT cells may play a dual role in the progression of HD-associated MDD: initially acting as inflammatory amplifiers to promote tissue damage, and later causing a loss of immune surveillance and inducing neuroinflammation due to exhaustion. Moreover, the association analysis between core genes and specific immune cell populations provides crucial clues for elucidating the molecular mechanisms of immune dysregulation in these disorders. CRAT showed a significant correlation with monocytes in both diseases, suggesting it may influence shared immune-inflammatory pathways by regulating the same immune cell type. These findings underscore the exploratory value of these novel biomarkers, providing hypothesis-generating evidence for future investigations into therapeutic targets and clinical management optimization.

Through in silico analysis of the GSE251779 dataset, we explored miRNAs associated with our core model genes and identified hsa-let-7b-5p as a key candidate. This finding contrasts with previous prospective studies suggesting that lower baseline plasma let-7b-5p levels correlate with an increased future risk of MDD.^[54]^ In this study, patients in the MDD group exhibited elevated let-7b-5p levels, suggesting a dynamic role for hsa-let-7b-5p in MDD pathogenesis. Recent research^[55]^ further reveals that let-7b plays a crucial role in regulating synaptic plasticity, potentially contributing to the pathophysiological mechanisms of treatment-resistant depression. This highlights its potential value as a therapeutic target for intervening in disease progression. Further bioinformatics analysis showed that both MGST1 and its co-pathway gene MAFG exhibited negative correlations with hsa-let-7b-5p expression. This suggests that the miRNA may jointly influence oxidative stress levels and immune cell homeostasis in MDD by suppressing these antioxidant and inflammation-regulating genes. This finding points the way toward elucidating the specific mechanisms of the “miRNA-core gene-immune cell” axis in MDD.

This study has several important limitations. First, the diagnostic model was developed and validated exclusively on a cohort of patients with MDD. Although the model is based on molecular features shared with HD transcriptomes, its ability to specifically identify MDD within the HD patient population and its clinical utility require prospective validation in an independent cohort. Therefore, this model is currently a proof-of-concept tool intended for research and risk stratification, not for standalone clinical diagnosis, and requires prospective validation in HD populations and translation into a clinically feasible assay. Second, despite standardized preprocessing, the retrospective integration of multi-platform public datasets implies residual unmeasured confounders and potential batch effects, which may simultaneously impact the model’s generalization ability across new data sources. Finally, all current mechanistic inferences derive from computational simulations and correlation analyses. The causal biological roles of these factors in HD-MDD interactions require direct experimental validation.

## 5. Conclusion

Collectively, by integrating bioinformatics and machine learning, this study identified shared molecular and immune signatures between HD and MDD. Based on these shared signatures, we constructed a 6-gene diagnostic model for MDD that yielded consistent discriminative performance across independent validation cohorts in this exploratory study. These findings not only provide a novel molecular framework for understanding the frequent co-occurrence of these conditions but also provide the foundation for future validation of this diagnostic approach in HD populations and the exploration of targeted therapeutic strategies.

## Acknowledgments

We express our gratitude to GEO for offering the platform and to the data proprietors for supplying the data.

## Author contributions

**Conceptualization:** Xuejing Zheng.

**Data curation:** Meng Zheng, Rongrong Wu.

**Formal analysis:** Meng Zheng, Pingli Huang, Rongrong Wu, Huihui Wang.

**Methodology:** Meng Zheng, Pingli Huang, Huihui Wang, Xuejing Zheng.

**Project administration:** Meng Zheng, Xuejing Zheng.

**Resources:** Meng Zheng.

**Software:** Meng Zheng, Huihui Wang, Ping Xu.

**Supervision:** Ping Xu, Xuejing Zheng.

**Validation:** Meng Zheng, Ping Xu.

**Writing – original draft:** Meng Zheng.

**Writing – review & editing:** Meng Zheng, Pingli Huang, Xuejing Zheng.
















