# Supplementary material for: Integrated bioinformatics and machine learning for constructing a diagnostic model of major depressive disorder leveraging shared signatures from hemodialysis: A cross-sectional study
Source: Medicine (Baltimore). 2026 Jun 5;105(23):e49113. doi: 10.1097/MD.0000000000049113 (PMC13246050; doi:10.1097/MD.0000000000049113)
Supplement: Supplementary file 1 [file medi-105-e49113-s001.docx]

# ****Supplementary Table 1. Machine Learning Model Hyperparameter Optimization Strategies****

| **Model** | **Hyperparameter** | **Range/Candidate Values** | **Optimization Strategy** |
| --- | --- | --- | --- |
| **Elastic Net** | α (L1/L2 mixing ratio) | [0, 0.2, 0.4, 0.6, 0.8, 1] | Grid search + 10-fold cross-validation |
|  | λ (regularization strength) | Auto-computed (100 values) | Minimize binomial deviance via |
| **Lasso/Ridge** | λ (regularization strength) | Auto-computed (100 values) | Minimize binomial deviance via |
| **SVM** | C (cost) | [0.1, 1, 10] | Grid search + 5-fold cross-validation |
|  | γ (kernel width) | Adaptive calculation | Formula: γ = 1/(n_features · var(X)) |
| **GBM** | N trees (number of trees) | Dynamic (max=10,000) | Early stopping (10-fold CV, patience=50) |
|  | Interaction depth | 3 (fixed) | Literature-based recommendation |
|  | shrinkage (learning rate) | 0.001 (fixed) | Ensure stable convergence |
| **XGBoost** | nround (iterations) | Dynamic (max=10) | 5-fold cross-validation |
|  | Max depth (tree depth) | 2 (fixed) | Pre-experimental validation |
|  | eta (learning rate) | 1 (fixed) | Benchmark testing results |
| **Random Forest** | N tree (number of trees) | 1,000 (fixed) | Empirical balance of efficiency/stability |
|  | Node size (min node size) | 5 (fixed) | Limit tree complexity |
| **glmBoost** | M stop (iterations) | Dynamic (max=40) | Cross-validation (cvrisk) |
| **plsRglm** | nt (number of components) | 10 (fixed) | Pre-experimental cv.plsRglm validation |
| **Naive Bayes** | None | - | Probability density estimation |
