# Supplementary material for: Integrated bioinformatics and machine learning for constructing a diagnostic model of major depressive disorder leveraging shared signatures from hemodialysis: A cross-sectional study
Source: Medicine (Baltimore). 2026 Jun 5;105(23):e49113. doi: 10.1097/MD.0000000000049113 (PMC13246050; doi:10.1097/MD.0000000000049113)
Supplement: Supplementary file 2 [file medi-105-e49113-s002.docx]

## ****Supplementary Table 2. 34 differentially intersecting genes in hemodialysis and major depressive disorder****

| **No** | **Gene ID** | **No** | **Gene ID** | **No** | **Gene ID** | **No** | **Gene ID** |
| --- | --- | --- | --- | --- | --- | --- | --- |
| 1 | FUT8 | 10 | MROH6 | 19 | CD8A | 28 | CAPNS2 |
| 2 | PDLIM4 | 11 | CRAT | 20 | TNNT1 | 29 | IL1R2 |
| 3 | OTUD3 | 12 | KLRC3 | 21 | RORA | 30 | IL7R |
| 4 | PYHIN1 | 13 | SORT1 | 22 | PLBD1 | 31 | C14orf28 |
| 5 | KLF12 | 14 | IQCB1 | 23 | RETN | 32 | CTSD |
| 6 | MAFG | 15 | BCL7A | 24 | EIF5A2 | 33 | TBXAS1 |
| 7 | PNPLA2 | 16 | TLR2 | 25 | XPO1 | 34 | GZMK |
| 8 | ZNF37BP | 17 | EPHA4 | 26 | C1RL |  |  |
| 9 | S100A12 | 18 | SDAD1 | 27 | MGST1 |  |  |
