# Supplementary material for: Integrated bioinformatics and machine learning for constructing a diagnostic model of major depressive disorder leveraging shared signatures from hemodialysis: A cross-sectional study
Source: Medicine (Baltimore). 2026 Jun 5;105(23):e49113. doi: 10.1097/MD.0000000000049113 (PMC13246050; doi:10.1097/MD.0000000000049113)
Supplement: Supplementary file 3 [file medi-105-e49113-s003.docx]

# ****Supplementary Table 3. GO function enrichment analysis****

| **ID** | **ONTOLOGY** | **Description** | **GeneRatio** | **BgRatio** | **pvalue** | **p.adjust** | **qvalue** | **geneID** | **Count** |
| --- | --- | --- | --- | --- | --- | --- | --- | --- | --- |
| BP | GO:0032868 | response to insulin | 5/30 | 324/18805 | 0.000147243 | 0.068805562 | 0.055038239 | SORT1/TLR2/EPHA4/RETN/CTSD | 5 |
| BP | GO:0000054 | ribosomal subunit export from nucleus | 2/30 | 14/18805 | 0.00022124 | 0.068805562 | 0.055038239 | SDAD1/XPO1 | 2 |
| BP | GO:0033750 | ribosome localization | 2/30 | 14/18805 | 0.00022124 | 0.068805562 | 0.055038239 | SDAD1/XPO1 | 2 |
| BP | GO:0043434 | response to peptide hormone | 5/30 | 494/18805 | 0.001014727 | 0.202497201 | 0.161979484 | SORT1/TLR2/EPHA4/RETN/CTSD | 5 |
| BP | GO:0070328 | triglyceride homeostasis | 2/30 | 44/18805 | 0.00223255 | 0.202497201 | 0.161979484 | PNPLA2/RORA | 2 |
| BP | GO:0055090 | acylglycerol homeostasis | 2/30 | 45/18805 | 0.002334079 | 0.202497201 | 0.161979484 | PNPLA2/RORA | 2 |
| BP | GO:0002449 | lymphocyte mediated immunity | 4/30 | 384/18805 | 0.003082168 | 0.202497201 | 0.161979484 | KLRC3/CD8A/C1RL/IL7R | 4 |
| BP | GO:0002460 | adaptive immune response based on somatic recombination of immune receptors built from immunoglobulin superfamily domains | 4/30 | 396/18805 | 0.003441614 | 0.202497201 | 0.161979484 | CD8A/RORA/C1RL/IL7R | 4 |
| BP | GO:0036006 | cellular response to macrophage colony-stimulating factor stimulus | 2/30 | 60/18805 | 0.0041116 | 0.202497201 | 0.161979484 | TLR2/EPHA4 | 2 |
| BP | GO:0070542 | response to fatty acid | 2/30 | 60/18805 | 0.0041116 | 0.202497201 | 0.161979484 | TLR2/TBXAS1 | 2 |
| BP | GO:0045599 | negative regulation of fat cell differentiation | 2/30 | 62/18805 | 0.004384002 | 0.202497201 | 0.161979484 | SORT1/RORA | 2 |
| BP | GO:0030162 | regulation of proteolysis | 4/30 | 430/18805 | 0.004613929 | 0.202497201 | 0.161979484 | PYHIN1/EPHA4/XPO1/IL1R2 | 4 |
| BP | GO:0036005 | response to macrophage colony-stimulating factor | 2/30 | 64/18805 | 0.004664568 | 0.202497201 | 0.161979484 | TLR2/EPHA4 | 2 |
| BP | GO:0050821 | protein stabilization | 3/30 | 234/18805 | 0.006027282 | 0.202497201 | 0.161979484 | OTUD3/PYHIN1/EPHA4 | 3 |
| BP | GO:0043112 | receptor metabolic process | 2/30 | 75/18805 | 0.006351353 | 0.202497201 | 0.161979484 | FUT8/CTSD | 2 |
| BP | GO:1903050 | regulation of proteolysis involved in protein catabolic process | 3/30 | 239/18805 | 0.00638944 | 0.202497201 | 0.161979484 | PYHIN1/EPHA4/XPO1 | 3 |
| BP | GO:0002443 | leukocyte mediated immunity | 4/30 | 483/18805 | 0.006938164 | 0.202497201 | 0.161979484 | KLRC3/CD8A/C1RL/IL7R | 4 |
| BP | GO:0045685 | regulation of glial cell differentiation | 2/30 | 80/18805 | 0.007196934 | 0.202497201 | 0.161979484 | TLR2/EPHA4 | 2 |
| BP | GO:0002274 | myeloid leukocyte activation | 3/30 | 251/18805 | 0.007311078 | 0.202497201 | 0.161979484 | S100A12/TLR2/RORA | 3 |
| BP | GO:0045444 | fat cell differentiation | 3/30 | 252/18805 | 0.007391259 | 0.202497201 | 0.161979484 | SORT1/RORA/RETN | 3 |
| BP | GO:0001906 | cell killing | 3/30 | 263/18805 | 0.008308011 | 0.202497201 | 0.161979484 | S100A12/KLRC3/IL7R | 3 |
| BP | GO:0046209 | nitric oxide metabolic process | 2/30 | 88/18805 | 0.008649793 | 0.202497201 | 0.161979484 | TLR2/RORA | 2 |
| BP | GO:0071347 | cellular response to interleukin-1 | 2/30 | 88/18805 | 0.008649793 | 0.202497201 | 0.161979484 | RORA/IL1R2 | 2 |
| BP | GO:2001057 | reactive nitrogen species metabolic process | 2/30 | 90/18805 | 0.009031919 | 0.202497201 | 0.161979484 | TLR2/RORA | 2 |
| BP | GO:0001910 | regulation of leukocyte mediated cytotoxicity | 2/30 | 100/18805 | 0.011053596 | 0.202497201 | 0.161979484 | KLRC3/IL7R | 2 |
| BP | GO:0002220 | innate immune response activating cell surface receptor signaling pathway | 2/30 | 100/18805 | 0.011053596 | 0.202497201 | 0.161979484 | KLRC3/TLR2 | 2 |
| BP | GO:0051099 | positive regulation of binding | 2/30 | 102/18805 | 0.011479807 | 0.202497201 | 0.161979484 | PYHIN1/EPHA4 | 2 |
| BP | GO:0031341 | regulation of cell killing | 2/30 | 110/18805 | 0.013255994 | 0.202497201 | 0.161979484 | KLRC3/IL7R | 2 |
| BP | GO:0001666 | response to hypoxia | 3/30 | 316/18805 | 0.01364462 | 0.202497201 | 0.161979484 | TLR2/EPHA4/RORA | 3 |
| BP | GO:0007249 | canonical NF-kappaB signal transduction | 3/30 | 318/18805 | 0.013876554 | 0.202497201 | 0.161979484 | S100A12/TLR2/RORA | 3 |
| BP | GO:0042116 | macrophage activation | 2/30 | 113/18805 | 0.013951078 | 0.202497201 | 0.161979484 | TLR2/RORA | 2 |
| BP | GO:0030217 | T cell differentiation | 3/30 | 324/18805 | 0.014585925 | 0.202497201 | 0.161979484 | CD8A/RORA/IL7R | 3 |
| BP | GO:0070555 | response to interleukin-1 | 2/30 | 116/18805 | 0.014661744 | 0.202497201 | 0.161979484 | RORA/IL1R2 | 2 |
| BP | GO:0002218 | activation of innate immune response | 3/30 | 325/18805 | 0.014706136 | 0.202497201 | 0.161979484 | PYHIN1/KLRC3/TLR2 | 3 |
| BP | GO:0019882 | antigen processing and presentation | 2/30 | 117/18805 | 0.014902069 | 0.202497201 | 0.161979484 | CD8A/CTSD | 2 |
| BP | GO:0036293 | response to decreased oxygen levels | 3/30 | 330/18805 | 0.015315716 | 0.202497201 | 0.161979484 | TLR2/EPHA4/RORA | 3 |
| BP | GO:0051092 | positive regulation of NF-kappaB transcription factor activity | 2/30 | 120/18805 | 0.015633273 | 0.202497201 | 0.161979484 | S100A12/TLR2 | 2 |
| BP | GO:0001915 | negative regulation of T cell mediated cytotoxicity | 3/30 | 10/18805 | 0.015842927 | 0.202497201 | 0.161979484 | IL7R | 1 |
| BP | GO:0007252 | I-kappaB phosphorylation | 1/30 | 10/18805 | 0.015842927 | 0.202497201 | 0.161979484 | TLR2 | 1 |
| BP | GO:0019371 | cyclooxygenase pathway | 1/30 | 10/18805 | 0.015842927 | 0.202497201 | 0.161979484 | TBXAS1 | 1 |
| BP | GO:0030002 | intracellular monoatomic anion homeostasis | 1/30 | 10/18805 | 0.015842927 | 0.202497201 | 0.161979484 | TBXAS1 | 1 |
| BP | GO:0030644 | intracellular chloride ion homeostasis | 1/30 | 10/18805 | 0.015842927 | 0.202497201 | 0.161979484 | TBXAS1 | 1 |
| BP | GO:0034635 | glutathione transport | 1/30 | 10/18805 | 0.015842927 | 0.202497201 | 0.161979484 | MGST1 | 1 |
| BP | GO:0035356 | intracellular triglyceride homeostasis | 1/30 | 10/18805 | 0.015842927 | 0.202497201 | 0.161979484 | PNPLA2 | 1 |
| BP | GO:0035457 | cellular response to interferon-alpha | 1/30 | 10/18805 | 0.015842927 | 0.202497201 | 0.161979484 | PYHIN1 | 1 |
| BP | GO:0051964 | negative regulation of synapse assembly | 1/30 | 10/18805 | 0.015842927 | 0.202497201 | 0.161979484 | TLR2 | 1 |
| BP | GO:0070339 | response to bacterial lipopeptide | 1/30 | 10/18805 | 0.015842927 | 0.202497201 | 0.161979484 | TLR2 | 1 |
| BP | GO:0071220 | cellular response to bacterial lipoprotein | 1/30 | 10/18805 | 0.015842927 | 0.202497201 | 0.161979484 | TLR2 | 1 |
| BP | GO:0071221 | cellular response to bacterial lipopeptide | 1/30 | 10/18805 | 0.015842927 | 0.202497201 | 0.161979484 | TLR2 | 1 |
| BP | GO:1900038 | negative regulation of cellular response to hypoxia | 1/30 | 10/18805 | 0.015842927 | 0.202497201 | 0.161979484 | EPHA4 | 1 |
| BP | GO:0002429 | immune response-activating cell surface receptor signaling pathway | 1/30 | 340/18805 | 0.01657762 | 0.202497201 | 0.161979484 | KLRC3/TLR2/CD8A | 3 |
| BP | GO:0006690 | icosanoid metabolic process | 2/30 | 124/18805 | 0.016631867 | 0.202497201 | 0.161979484 | TLR2/TBXAS1 | 2 |
| BP | GO:0042742 | defense response to bacterium | 3/30 | 343/18805 | 0.016967341 | 0.202497201 | 0.161979484 | S100A12/TLR2/IL7R | 3 |
| BP | GO:0006805 | xenobiotic metabolic process | 2/30 | 126/18805 | 0.017141202 | 0.202497201 | 0.161979484 | S100A12/RORA | 2 |
| BP | GO:0032493 | response to bacterial lipoprotein | 1/30 | 11/18805 | 0.017413808 | 0.202497201 | 0.161979484 | TLR2 | 1 |
| BP | GO:0033089 | positive regulation of T cell differentiation in thymus | 1/30 | 11/18805 | 0.017413808 | 0.202497201 | 0.161979484 | IL7R | 1 |
| BP | GO:0035871 | protein K11-linked deubiquitination | 1/30 | 11/18805 | 0.017413808 | 0.202497201 | 0.161979484 | OTUD3 | 1 |
| BP | GO:0070391 | response to lipoteichoic acid | 1/30 | 11/18805 | 0.017413808 | 0.202497201 | 0.161979484 | TLR2 | 1 |
| BP | GO:0071223 | cellular response to lipoteichoic acid | 1/30 | 11/18805 | 0.017413808 | 0.202497201 | 0.161979484 | TLR2 | 1 |
| BP | GO:0072178 | nephric duct morphogenesis | 1/30 | 11/18805 | 0.017413808 | 0.202497201 | 0.161979484 | EPHA4 | 1 |
| BP | GO:0090160 | Golgi to lysosome transport | 1/30 | 11/18805 | 0.017413808 | 0.202497201 | 0.161979484 | SORT1 | 1 |
| BP | GO:0140507 | granzyme-mediated programmed cell death signaling pathway | 1/30 | 11/18805 | 0.017413808 | 0.202497201 | 0.161979484 | GZMK | 1 |
| BP | GO:0016042 | lipid catabolic process | 3/30 | 350/18805 | 0.01789677 | 0.202497201 | 0.161979484 | PNPLA2/CRAT/PLBD1 | 3 |
| BP | GO:0050830 | defense response to Gram-positive bacterium | 2/30 | 131/18805 | 0.018443454 | 0.202497201 | 0.161979484 | TLR2/IL7R | 2 |
| BP | GO:0001553 | luteinization | 1/30 | 12/18805 | 0.018982266 | 0.202497201 | 0.161979484 | RETN | 1 |
| BP | GO:0021534 | cell proliferation in hindbrain | 1/30 | 12/18805 | 0.018982266 | 0.202497201 | 0.161979484 | RORA | 1 |
| BP | GO:0021924 | cell proliferation in external granule layer | 1/30 | 12/18805 | 0.018982266 | 0.202497201 | 0.161979484 | RORA | 1 |
| BP | GO:0021930 | cerebellar granule cell precursor proliferation | 1/30 | 12/18805 | 0.018982266 | 0.202497201 | 0.161979484 | RORA | 1 |
| BP | GO:0032610 | interleukin-1 alpha production | 1/30 | 12/18805 | 0.018982266 | 0.202497201 | 0.161979484 | IL1R2 | 1 |
| BP | GO:0032650 | regulation of interleukin-1 alpha production | 1/30 | 12/18805 | 0.018982266 | 0.202497201 | 0.161979484 | IL1R2 | 1 |
| BP | GO:1904464 | regulation of matrix metallopeptidase secretion | 1/30 | 12/18805 | 0.018982266 | 0.202497201 | 0.161979484 | TLR2 | 1 |
| BP | GO:1990773 | matrix metallopeptidase secretion | 1/30 | 12/18805 | 0.018982266 | 0.202497201 | 0.161979484 | TLR2 | 1 |
| BP | GO:2000659 | regulation of interleukin-1-mediated signaling pathway | 1/30 | 12/18805 | 0.018982266 | 0.202497201 | 0.161979484 | IL1R2 | 1 |
| BP | GO:2000833 | positive regulation of steroid hormone secretion | 1/30 | 12/18805 | 0.018982266 | 0.202497201 | 0.161979484 | RETN | 1 |
| BP | GO:0070482 | response to oxygen levels | 3/30 | 358/18805 | 0.018993483 | 0.202497201 | 0.161979484 | TLR2/EPHA4/RORA | 3 |
| BP | GO:0002456 | T cell mediated immunity | 2/30 | 137/18805 | 0.020059895 | 0.202497201 | 0.161979484 | CD8A/IL7R | 2 |
| BP | GO:0009437 | carnitine metabolic process | 1/30 | 13/18805 | 0.020548302 | 0.202497201 | 0.161979484 | CRAT | 1 |
| BP | GO:0010896 | regulation of triglyceride catabolic process | 1/30 | 13/18805 | 0.020548302 | 0.202497201 | 0.161979484 | PNPLA2 | 1 |
| BP | GO:0021702 | cerebellar Purkinje cell differentiation | 1/30 | 13/18805 | 0.020548302 | 0.202497201 | 0.161979484 | RORA | 1 |
| BP | GO:0032490 | detection of molecule of bacterial origin | 1/30 | 13/18805 | 0.020548302 | 0.202497201 | 0.161979484 | TLR2 | 1 |
| BP | GO:0048521 | negative regulation of behavior | 1/30 | 13/18805 | 0.020548302 | 0.202497201 | 0.161979484 | RETN | 1 |
| BP | GO:1905939 | regulation of gonad development | 1/30 | 13/18805 | 0.020548302 | 0.202497201 | 0.161979484 | RETN | 1 |
| BP | GO:0002768 | immune response-regulating cell surface receptor signaling pathway | 3/30 | 372/18805 | 0.021001596 | 0.202497201 | 0.161979484 | KLRC3/TLR2/CD8A | 3 |
| BP | GO:0010927 | cellular component assembly involved in morphogenesis | 2/30 | 141/18805 | 0.021169543 | 0.202497201 | 0.161979484 | TLR2/TNNT1 | 2 |
| BP | GO:0032989 | cellular anatomical entity morphogenesis | 2/30 | 141/18805 | 0.021169543 | 0.202497201 | 0.161979484 | TLR2/TNNT1 | 2 |
| BP | GO:0006448 | regulation of translational elongation | 1/30 | 14/18805 | 0.022111923 | 0.202497201 | 0.161979484 | EIF5A2 | 1 |
| BP | GO:0014733 | regulation of skeletal muscle adaptation | 1/30 | 14/18805 | 0.022111923 | 0.202497201 | 0.161979484 | TNNT1 | 1 |
| BP | GO:0038180 | nerve growth factor signaling pathway | 1/30 | 14/18805 | 0.022111923 | 0.202497201 | 0.161979484 | SORT1 | 1 |
| BP | GO:1900272 | negative regulation of long-term synaptic potentiation | 1/30 | 14/18805 | 0.022111923 | 0.202497201 | 0.161979484 | EPHA4 | 1 |
| BP | GO:1905809 | negative regulation of synapse organization | 1/30 | 14/18805 | 0.022111923 | 0.202497201 | 0.161979484 | TLR2 | 1 |
| BP | GO:0045598 | regulation of fat cell differentiation | 2/30 | 147/18805 | 0.02288118 | 0.202497201 | 0.161979484 | SORT1/RORA | 2 |
| BP | GO:0031647 | regulation of protein stability | 3/30 | 386/18805 | 0.023123154 | 0.202497201 | 0.161979484 | OTUD3/PYHIN1/EPHA4 | 3 |
| BP | GO:0010889 | regulation of triglyceride storage | 1/30 | 15/18805 | 0.02367313 | 0.202497201 | 0.161979484 | PNPLA2 | 1 |
| BP | GO:0014004 | microglia differentiation | 1/30 | 15/18805 | 0.02367313 | 0.202497201 | 0.161979484 | TLR2 | 1 |
| BP | GO:0021694 | cerebellar Purkinje cell layer formation | 1/30 | 15/18805 | 0.02367313 | 0.202497201 | 0.161979484 | RORA | 1 |
| BP | GO:0032621 | interleukin-18 production | 1/30 | 15/18805 | 0.02367313 | 0.202497201 | 0.161979484 | TLR2 | 1 |
| BP | GO:0032661 | regulation of interleukin-18 production | 1/30 | 15/18805 | 0.02367313 | 0.202497201 | 0.161979484 | TLR2 | 1 |
| BP | GO:0042159 | lipoprotein catabolic process | 1/30 | 15/18805 | 0.02367313 | 0.202497201 | 0.161979484 | CTSD | 1 |
| BP | GO:0048681 | negative regulation of axon regeneration | 1/30 | 15/18805 | 0.02367313 | 0.202497201 | 0.161979484 | EPHA4 | 1 |
| BP | GO:0051791 | medium-chain fatty acid metabolic process | 1/30 | 15/18805 | 0.02367313 | 0.202497201 | 0.161979484 | CRAT | 1 |
| BP | GO:0072176 | nephric duct development | 1/30 | 15/18805 | 0.02367313 | 0.202497201 | 0.161979484 | EPHA4 | 1 |
| BP | GO:0030534 | adult behavior | 2/30 | 152/18805 | 0.024350045 | 0.202497201 | 0.161979484 | MAFG/EPHA4 | 2 |
| BP | GO:0045089 | positive regulation of innate immune response | 3/30 | 395/18805 | 0.024547032 | 0.202497201 | 0.161979484 | PYHIN1/KLRC3/TLR2 | 3 |
| BP | GO:0001909 | leukocyte mediated cytotoxicity | 2/30 | 153/18805 | 0.024648391 | 0.202497201 | 0.161979484 | KLRC3/IL7R | 2 |
| BP | GO:0006004 | fucose metabolic process | 1/30 | 16/18805 | 0.025231927 | 0.202497201 | 0.161979484 | FUT8 | 1 |
| BP | GO:0016045 | detection of bacterium | 1/30 | 16/18805 | 0.025231927 | 0.202497201 | 0.161979484 | TLR2 | 1 |
| BP | GO:0046459 | short-chain fatty acid metabolic process | 1/30 | 16/18805 | 0.025231927 | 0.202497201 | 0.161979484 | CRAT | 1 |
| BP | GO:0061098 | positive regulation of protein tyrosine kinase activity | 1/30 | 16/18805 | 0.025231927 | 0.202497201 | 0.161979484 | EPHA4 | 1 |
| BP | GO:0070571 | negative regulation of neuron projection regeneration | 1/30 | 16/18805 | 0.025231927 | 0.202497201 | 0.161979484 | EPHA4 | 1 |
| BP | GO:0098883 | synapse pruning | 1/30 | 16/18805 | 0.025231927 | 0.202497201 | 0.161979484 | EPHA4 | 1 |
| BP | GO:1901142 | insulin metabolic process | 1/30 | 16/18805 | 0.025231927 | 0.202497201 | 0.161979484 | CTSD | 1 |
| BP | GO:0071456 | cellular response to hypoxia | 2/30 | 156/18805 | 0.025552479 | 0.202497201 | 0.161979484 | EPHA4/RORA | 2 |
| BP | GO:0006577 | amino-acid betaine metabolic process | 1/30 | 17/18805 | 0.026788319 | 0.202497201 | 0.161979484 | CRAT | 1 |
| BP | GO:0006895 | Golgi to endosome transport | 1/30 | 17/18805 | 0.026788319 | 0.202497201 | 0.161979484 | SORT1 | 1 |
| BP | GO:0034134 | toll-like receptor 2 signaling pathway | 1/30 | 17/18805 | 0.026788319 | 0.202497201 | 0.161979484 | TLR2 | 1 |
| BP | GO:0036065 | fucosylation | 1/30 | 17/18805 | 0.026788319 | 0.202497201 | 0.161979484 | FUT8 | 1 |
| BP | GO:0046068 | cGMP metabolic process | 1/30 | 17/18805 | 0.026788319 | 0.202497201 | 0.161979484 | RORA | 1 |
| BP | GO:0036294 | cellular response to decreased oxygen levels | 2/30 | 163/18805 | 0.027714147 | 0.202497201 | 0.161979484 | EPHA4/RORA | 2 |
| BP | GO:0050729 | positive regulation of inflammatory response | 2/30 | 163/18805 | 0.027714147 | 0.202497201 | 0.161979484 | S100A12/TLR2 | 2 |
| BP | GO:0006415 | translational termination | 1/30 | 18/18805 | 0.028342308 | 0.202497201 | 0.161979484 | EIF5A2 | 1 |
| BP | GO:0030730 | triglyceride storage | 1/30 | 18/18805 | 0.028342308 | 0.202497201 | 0.161979484 | PNPLA2 | 1 |
| BP | GO:0042753 | positive regulation of circadian rhythm | 1/30 | 18/18805 | 0.028342308 | 0.202497201 | 0.161979484 | RORA | 1 |
| BP | GO:0045861 | negative regulation of proteolysis | 2/30 | 165/18805 | 0.028344985 | 0.202497201 | 0.161979484 | EPHA4/IL1R2 | 2 |
| BP | GO:0120254 | olefinic compound metabolic process | 2/30 | 165/18805 | 0.028344985 | 0.202497201 | 0.161979484 | PNPLA2/TBXAS1 | 2 |
| BP | GO:0002833 | positive regulation of response to biotic stimulus | 3/30 | 425/18805 | 0.029632665 | 0.202497201 | 0.161979484 | PYHIN1/KLRC3/TLR2 | 3 |
| BP | GO:0006857 | oligopeptide transport | 1/30 | 19/18805 | 0.029893899 | 0.202497201 | 0.161979484 | MGST1 | 1 |
| BP | GO:0006896 | Golgi to vacuole transport | 1/30 | 19/18805 | 0.029893899 | 0.202497201 | 0.161979484 | SORT1 | 1 |
| BP | GO:0021692 | cerebellar Purkinje cell layer morphogenesis | 1/30 | 19/18805 | 0.029893899 | 0.202497201 | 0.161979484 | RORA | 1 |
| BP | GO:0055064 | chloride ion homeostasis | 1/30 | 19/18805 | 0.029893899 | 0.202497201 | 0.161979484 | TBXAS1 | 1 |
| BP | GO:0098543 | detection of other organism | 1/30 | 19/18805 | 0.029893899 | 0.202497201 | 0.161979484 | TLR2 | 1 |
| BP | GO:0042692 | muscle cell differentiation | 3/30 | 429/18805 | 0.030350148 | 0.202497201 | 0.161979484 | SORT1/TNNT1/RORA | 3 |
| BP | GO:2000058 | regulation of ubiquitin-dependent protein catabolic process | 2/30 | 172/18805 | 0.030598324 | 0.202497201 | 0.161979484 | PYHIN1/XPO1 | 2 |
| BP | GO:0030098 | lymphocyte differentiation | 3/30 | 432/18805 | 0.030894332 | 0.202497201 | 0.161979484 | CD8A/RORA/IL7R | 3 |
| BP | GO:0051168 | nuclear export | 2/30 | 174/18805 | 0.031254942 | 0.202497201 | 0.161979484 | SDAD1/XPO1 | 2 |
| BP | GO:0010950 | positive regulation of endopeptidase activity | 1/30 | 20/18805 | 0.031443094 | 0.202497201 | 0.161979484 | EPHA4 | 1 |
| BP | GO:0033194 | response to hydroperoxide | 1/30 | 20/18805 | 0.031443094 | 0.202497201 | 0.161979484 | MGST1 | 1 |
| BP | GO:0035455 | response to interferon-alpha | 1/30 | 20/18805 | 0.031443094 | 0.202497201 | 0.161979484 | PYHIN1 | 1 |
| BP | GO:0098761 | cellular response to interleukin-7 | 1/30 | 20/18805 | 0.031443094 | 0.202497201 | 0.161979484 | IL7R | 1 |
| BP | GO:1900037 | regulation of cellular response to hypoxia | 1/30 | 20/18805 | 0.031443094 | 0.202497201 | 0.161979484 | EPHA4 | 1 |
| BP | GO:0008286 | insulin receptor signaling pathway | 2/30 | 176/18805 | 0.031917177 | 0.202497201 | 0.161979484 | EPHA4/CTSD | 2 |
| BP | GO:0050806 | positive regulation of synaptic transmission | 2/30 | 177/18805 | 0.032250389 | 0.202497201 | 0.161979484 | EPHA4/RETN | 2 |
| BP | GO:0050727 | regulation of inflammatory response | 3/30 | 442/18805 | 0.032745805 | 0.202497201 | 0.161979484 | S100A12/TLR2/RORA | 3 |
| BP | GO:0071453 | cellular response to oxygen levels | 2/30 | 179/18805 | 0.032920977 | 0.202497201 | 0.161979484 | EPHA4/RORA | 2 |
| BP | GO:0007413 | axonal fasciculation | 1/30 | 21/18805 | 0.032989898 | 0.202497201 | 0.161979484 | EPHA4 | 1 |
| BP | GO:0048535 | lymph node development | 1/30 | 21/18805 | 0.032989898 | 0.202497201 | 0.161979484 | IL7R | 1 |
| BP | GO:0055081 | monoatomic anion homeostasis | 1/30 | 21/18805 | 0.032989898 | 0.202497201 | 0.161979484 | TBXAS1 | 1 |
| BP | GO:0098760 | response to interleukin-7 | 1/30 | 21/18805 | 0.032989898 | 0.202497201 | 0.161979484 | IL7R | 1 |
| BP | GO:0106030 | neuron projection fasciculation | 1/30 | 21/18805 | 0.032989898 | 0.202497201 | 0.161979484 | EPHA4 | 1 |
| BP | GO:0010720 | positive regulation of cell development | 3/30 | 447/18805 | 0.033693155 | 0.203192137 | 0.16253537 | TLR2/EPHA4/IL7R | 3 |
| BP | GO:0006491 | N-glycan processing | 1/30 | 22/18805 | 0.034534313 | 0.203192137 | 0.16253537 | FUT8 | 1 |
| BP | GO:0050765 | negative regulation of phagocytosis | 1/30 | 22/18805 | 0.034534313 | 0.203192137 | 0.16253537 | TLR2 | 1 |
| BP | GO:0090208 | positive regulation of triglyceride metabolic process | 1/30 | 22/18805 | 0.034534313 | 0.203192137 | 0.16253537 | PNPLA2 | 1 |
| BP | GO:1902004 | positive regulation of amyloid-beta formation | 1/30 | 22/18805 | 0.034534313 | 0.203192137 | 0.16253537 | EPHA4 | 1 |
| BP | GO:0055088 | lipid homeostasis | 2/30 | 186/18805 | 0.035311234 | 0.203192137 | 0.16253537 | PNPLA2/RORA | 2 |
| BP | GO:0022411 | cellular component disassembly | 3/30 | 456/18805 | 0.035434598 | 0.203192137 | 0.16253537 | PNPLA2/EPHA4/EIF5A2 | 3 |
| BP | GO:0002223 | stimulatory C-type lectin receptor signaling pathway | 1/30 | 23/18805 | 0.036076344 | 0.203192137 | 0.16253537 | KLRC3 | 1 |
| BP | GO:0010888 | negative regulation of lipid storage | 1/30 | 23/18805 | 0.036076344 | 0.203192137 | 0.16253537 | PNPLA2 | 1 |
| BP | GO:0010955 | negative regulation of protein processing | 1/30 | 23/18805 | 0.036076344 | 0.203192137 | 0.16253537 | IL1R2 | 1 |
| BP | GO:1905244 | regulation of modification of synaptic structure | 1/30 | 23/18805 | 0.036076344 | 0.203192137 | 0.16253537 | EPHA4 | 1 |
| BP | GO:1990840 | response to lectin | 1/30 | 23/18805 | 0.036076344 | 0.203192137 | 0.16253537 | KLRC3 | 1 |
| BP | GO:1990858 | cellular response to lectin | 1/30 | 23/18805 | 0.036076344 | 0.203192137 | 0.16253537 | KLRC3 | 1 |
| BP | GO:1905952 | regulation of lipid localization | 2/30 | 189/18805 | 0.036355908 | 0.203192137 | 0.16253537 | PNPLA2/RETN | 2 |
| BP | GO:0021533 | cell differentiation in hindbrain | 1/30 | 24/18805 | 0.037615995 | 0.203192137 | 0.16253537 | RORA | 1 |
| BP | GO:0046628 | positive regulation of insulin receptor signaling pathway | 1/30 | 24/18805 | 0.037615995 | 0.203192137 | 0.16253537 | CTSD | 1 |
| BP | GO:1900407 | regulation of cellular response to oxidative stress | 1/30 | 24/18805 | 0.037615995 | 0.203192137 | 0.16253537 | FUT8 | 1 |
| BP | GO:2000831 | regulation of steroid hormone secretion | 1/30 | 24/18805 | 0.037615995 | 0.203192137 | 0.16253537 | RETN | 1 |
| BP | GO:0051091 | positive regulation of DNA-binding transcription factor activity | 2/30 | 195/18805 | 0.038481057 | 0.203192137 | 0.16253537 | S100A12/TLR2 | 2 |
| BP | GO:0032288 | myelin assembly | 1/30 | 25/18805 | 0.039153267 | 0.203192137 | 0.16253537 | TLR2 | 1 |
| BP | GO:0045932 | negative regulation of muscle contraction | 1/30 | 25/18805 | 0.039153267 | 0.203192137 | 0.16253537 | TNNT1 | 1 |
| BP | GO:0070233 | negative regulation of T cell apoptotic process | 1/30 | 25/18805 | 0.039153267 | 0.203192137 | 0.16253537 | IL7R | 1 |
| BP | GO:1903318 | negative regulation of protein maturation | 1/30 | 25/18805 | 0.039153267 | 0.203192137 | 0.16253537 | IL1R2 | 1 |
| BP | GO:0071466 | cellular response to xenobiotic stimulus | 2/30 | 198/18805 | 0.039561274 | 0.203192137 | 0.16253537 | S100A12/RORA | 2 |
| BP | GO:0002706 | regulation of lymphocyte mediated immunity | 2/30 | 200/18805 | 0.040287865 | 0.203192137 | 0.16253537 | KLRC3/IL7R | 2 |
| BP | GO:0060759 | regulation of response to cytokine stimulus | 2/30 | 201/18805 | 0.040653081 | 0.203192137 | 0.16253537 | TLR2/IL1R2 | 2 |
| BP | GO:0008045 | motor neuron axon guidance | 1/30 | 26/18805 | 0.040688166 | 0.203192137 | 0.16253537 | EPHA4 | 1 |
| BP | GO:0036315 | cellular response to sterol | 1/30 | 26/18805 | 0.040688166 | 0.203192137 | 0.16253537 | RORA | 1 |
| BP | GO:0043501 | skeletal muscle adaptation | 1/30 | 26/18805 | 0.040688166 | 0.203192137 | 0.16253537 | TNNT1 | 1 |
| BP | GO:0046339 | diacylglycerol metabolic process | 1/30 | 26/18805 | 0.040688166 | 0.203192137 | 0.16253537 | PNPLA2 | 1 |
| BP | GO:0048011 | neurotrophin TRK receptor signaling pathway | 1/30 | 26/18805 | 0.040688166 | 0.203192137 | 0.16253537 | SORT1 | 1 |
| BP | GO:0060259 | regulation of feeding behavior | 1/30 | 26/18805 | 0.040688166 | 0.203192137 | 0.16253537 | RETN | 1 |
| BP | GO:0098581 | detection of external biotic stimulus | 1/30 | 26/18805 | 0.040688166 | 0.203192137 | 0.16253537 | TLR2 | 1 |
| BP | GO:1900078 | positive regulation of cellular response to insulin stimulus | 1/30 | 26/18805 | 0.040688166 | 0.203192137 | 0.16253537 | CTSD | 1 |
| BP | GO:0045088 | regulation of innate immune response | 3/30 | 482/18805 | 0.040725541 | 0.203192137 | 0.16253537 | PYHIN1/KLRC3/TLR2 | 3 |
| BP | GO:0009312 | oligosaccharide biosynthetic process | 1/30 | 27/18805 | 0.042220695 | 0.204097747 | 0.163259776 | FUT8 | 1 |
| BP | GO:0021697 | cerebellar cortex formation | 1/30 | 27/18805 | 0.042220695 | 0.204097747 | 0.163259776 | RORA | 1 |
| BP | GO:0022010 | central nervous system myelination | 1/30 | 27/18805 | 0.042220695 | 0.204097747 | 0.163259776 | TLR2 | 1 |
| BP | GO:0032291 | axon ensheathment in central nervous system | 1/30 | 27/18805 | 0.042220695 | 0.204097747 | 0.163259776 | TLR2 | 1 |
| BP | GO:0048714 | positive regulation of oligodendrocyte differentiation | 1/30 | 27/18805 | 0.042220695 | 0.204097747 | 0.163259776 | TLR2 | 1 |
| BP | GO:0071108 | protein K48-linked deubiquitination | 1/30 | 27/18805 | 0.042220695 | 0.204097747 | 0.163259776 | OTUD3 | 1 |
| BP | GO:0051098 | regulation of binding | 2/30 | 209/18805 | 0.043620265 | 0.204097747 | 0.163259776 | PYHIN1/EPHA4 | 2 |
| BP | GO:0021680 | cerebellar Purkinje cell layer development | 1/30 | 28/18805 | 0.043750857 | 0.204097747 | 0.163259776 | RORA | 1 |
| BP | GO:0033081 | regulation of T cell differentiation in thymus | 1/30 | 28/18805 | 0.043750857 | 0.204097747 | 0.163259776 | IL7R | 1 |
| BP | GO:0035458 | cellular response to interferon-beta | 1/30 | 28/18805 | 0.043750857 | 0.204097747 | 0.163259776 | PYHIN1 | 1 |
| BP | GO:0050996 | positive regulation of lipid catabolic process | 1/30 | 28/18805 | 0.043750857 | 0.204097747 | 0.163259776 | PNPLA2 | 1 |
| BP | GO:1902993 | positive regulation of amyloid precursor protein catabolic process | 1/30 | 28/18805 | 0.043750857 | 0.204097747 | 0.163259776 | EPHA4 | 1 |
| BP | GO:2000819 | regulation of nucleotide-excision repair | 1/30 | 28/18805 | 0.043750857 | 0.204097747 | 0.163259776 | BCL7A | 1 |
| BP | GO:0045860 | positive regulation of protein kinase activity | 2/30 | 210/18805 | 0.043996776 | 0.204223841 | 0.16336064 | S100A12/EPHA4 | 2 |
| BP | GO:0021952 | central nervous system projection neuron axonogenesis | 1/30 | 29/18805 | 0.045278655 | 0.205072745 | 0.164039687 | EPHA4 | 1 |
| BP | GO:0035929 | steroid hormone secretion | 1/30 | 29/18805 | 0.045278655 | 0.205072745 | 0.164039687 | RETN | 1 |
| BP | GO:0045954 | positive regulation of natural killer cell mediated cytotoxicity | 1/30 | 29/18805 | 0.045278655 | 0.205072745 | 0.164039687 | KLRC3 | 1 |
| BP | GO:0060384 | innervation | 1/30 | 29/18805 | 0.045278655 | 0.205072745 | 0.164039687 | EPHA4 | 1 |
| BP | GO:0061744 | motor behavior | 1/30 | 29/18805 | 0.045278655 | 0.205072745 | 0.164039687 | BCL7A | 1 |
| BP | GO:0021953 | central nervous system neuron differentiation | 2/30 | 215/18805 | 0.045897715 | 0.206872309 | 0.165479175 | EPHA4/RORA | 2 |
| BP | GO:0031503 | protein-containing complex localization | 2/30 | 216/18805 | 0.046281548 | 0.207599442 | 0.166060816 | SDAD1/XPO1 | 2 |
| BP | GO:0010952 | positive regulation of peptidase activity | 1/30 | 30/18805 | 0.046804094 | 0.207625434 | 0.166081607 | EPHA4 | 1 |
| BP | GO:0019433 | triglyceride catabolic process | 1/30 | 31/18805 | 0.048327177 | 0.207625434 | 0.166081607 | PNPLA2 | 1 |
| BP | GO:0019886 | antigen processing and presentation of exogenous peptide antigen via MHC class II | 1/30 | 31/18805 | 0.048327177 | 0.207625434 | 0.166081607 | CTSD | 1 |
| BP | GO:0099558 | maintenance of synapse structure | 1/30 | 31/18805 | 0.048327177 | 0.207625434 | 0.166081607 | SORT1 | 1 |
| BP | GO:0150146 | cell junction disassembly | 1/30 | 31/18805 | 0.048327177 | 0.207625434 | 0.166081607 | EPHA4 | 1 |
| BP | GO:0033674 | positive regulation of kinase activity | 2/30 | 223/18805 | 0.049001849 | 0.207625434 | 0.166081607 | S100A12/EPHA4 | 2 |
| BP | GO:0045862 | positive regulation of proteolysis | 2/30 | 223/18805 | 0.049001849 | 0.207625434 | 0.166081607 | PYHIN1/EPHA4 | 2 |
| BP | GO:0001516 | prostaglandin biosynthetic process | 1/30 | 32/18805 | 0.049847907 | 0.207625434 | 0.166081607 | TBXAS1 | 1 |
| BP | GO:0001911 | negative regulation of leukocyte mediated cytotoxicity | 1/30 | 32/18805 | 0.049847907 | 0.207625434 | 0.166081607 | IL7R | 1 |
| BP | GO:0002710 | negative regulation of T cell mediated immunity | 1/30 | 32/18805 | 0.049847907 | 0.207625434 | 0.166081607 | IL7R | 1 |
| BP | GO:0006622 | protein targeting to lysosome | 1/30 | 32/18805 | 0.049847907 | 0.207625434 | 0.166081607 | SORT1 | 1 |
| BP | GO:0010575 | positive regulation of vascular endothelial growth factor production | 1/30 | 32/18805 | 0.049847907 | 0.207625434 | 0.166081607 | RORA | 1 |
| BP | GO:0019082 | viral protein processing | 1/30 | 32/18805 | 0.049847907 | 0.207625434 | 0.166081607 | FUT8 | 1 |
| BP | GO:0034250 | positive regulation of amide metabolic process | 1/30 | 32/18805 | 0.049847907 | 0.207625434 | 0.166081607 | EPHA4 | 1 |
| BP | GO:0046457 | prostanoid biosynthetic process | 1/30 | 32/18805 | 0.049847907 | 0.207625434 | 0.166081607 | TBXAS1 | 1 |
| BP | GO:0048679 | regulation of axon regeneration | 1/30 | 32/18805 | 0.049847907 | 0.207625434 | 0.166081607 | EPHA4 | 1 |
| BP | GO:0016485 | protein processing | 2/30 | 227/18805 | 0.05058221 | 0.209747565 | 0.167779121 | C1RL/IL1R2 | 2 |
| BP | GO:0045907 | positive regulation of vasoconstriction | 1/30 | 33/18805 | 0.051366288 | 0.211122231 | 0.168878729 | TBXAS1 | 1 |
| BP | GO:0061097 | regulation of protein tyrosine kinase activity | 1/30 | 33/18805 | 0.051366288 | 0.211122231 | 0.168878729 | EPHA4 | 1 |
| BP | GO:0006691 | leukotriene metabolic process | 1/30 | 34/18805 | 0.052882323 | 0.211756254 | 0.169385891 | TLR2 | 1 |
| BP | GO:0007628 | adult walking behavior | 1/30 | 34/18805 | 0.052882323 | 0.211756254 | 0.169385891 | EPHA4 | 1 |
| BP | GO:0048710 | regulation of astrocyte differentiation | 1/30 | 34/18805 | 0.052882323 | 0.211756254 | 0.169385891 | EPHA4 | 1 |
| BP | GO:0070498 | interleukin-1-mediated signaling pathway | 1/30 | 34/18805 | 0.052882323 | 0.211756254 | 0.169385891 | IL1R2 | 1 |
| BP | GO:0070570 | regulation of neuron projection regeneration | 1/30 | 34/18805 | 0.052882323 | 0.211756254 | 0.169385891 | EPHA4 | 1 |
| BP | GO:1901889 | negative regulation of cell junction assembly | 1/30 | 34/18805 | 0.052882323 | 0.211756254 | 0.169385891 | TLR2 | 1 |
| BP | GO:0050769 | positive regulation of neurogenesis | 2/30 | 234/18805 | 0.053392147 | 0.212309438 | 0.169828388 | TLR2/EPHA4 | 2 |
| BP | GO:0002495 | antigen processing and presentation of peptide antigen via MHC class II | 1/30 | 35/18805 | 0.054396016 | 0.212309438 | 0.169828388 | CTSD | 1 |
| BP | GO:0002717 | positive regulation of natural killer cell mediated immunity | 1/30 | 35/18805 | 0.054396016 | 0.212309438 | 0.169828388 | KLRC3 | 1 |
| BP | GO:0031342 | negative regulation of cell killing | 1/30 | 35/18805 | 0.054396016 | 0.212309438 | 0.169828388 | IL7R | 1 |
| BP | GO:0035456 | response to interferon-beta | 1/30 | 35/18805 | 0.054396016 | 0.212309438 | 0.169828388 | PYHIN1 | 1 |
| BP | GO:0050775 | positive regulation of dendrite morphogenesis | 1/30 | 35/18805 | 0.054396016 | 0.212309438 | 0.169828388 | EPHA4 | 1 |
| BP | GO:0019318 | hexose metabolic process | 2/30 | 237/18805 | 0.05461336 | 0.212309438 | 0.169828388 | FUT8/RORA | 2 |
| BP | GO:0001782 | B cell homeostasis | 1/30 | 36/18805 | 0.05590737 | 0.212904393 | 0.170304298 | IL7R | 1 |
| BP | GO:0036314 | response to sterol | 1/30 | 36/18805 | 0.05590737 | 0.212904393 | 0.170304298 | RORA | 1 |
| BP | GO:0043388 | positive regulation of DNA binding | 1/30 | 36/18805 | 0.05590737 | 0.212904393 | 0.170304298 | PYHIN1 | 1 |
| BP | GO:0140058 | neuron projection arborization | 1/30 | 36/18805 | 0.05590737 | 0.212904393 | 0.170304298 | BCL7A | 1 |
| BP | GO:1902882 | regulation of response to oxidative stress | 1/30 | 36/18805 | 0.05590737 | 0.212904393 | 0.170304298 | FUT8 | 1 |
| BP | GO:0002504 | antigen processing and presentation of peptide or polysaccharide antigen via MHC class II | 1/30 | 37/18805 | 0.057416389 | 0.215138518 | 0.172091397 | CTSD | 1 |
| BP | GO:0010770 | positive regulation of cell morphogenesis | 1/30 | 37/18805 | 0.057416389 | 0.215138518 | 0.172091397 | EPHA4 | 1 |
| BP | GO:0021955 | central nervous system neuron axonogenesis | 1/30 | 37/18805 | 0.057416389 | 0.215138518 | 0.172091397 | EPHA4 | 1 |
| BP | GO:0046825 | regulation of protein export from nucleus | 1/30 | 37/18805 | 0.057416389 | 0.215138518 | 0.172091397 | XPO1 | 1 |
| BP | GO:0021696 | cerebellar cortex morphogenesis | 1/30 | 38/18805 | 0.058923076 | 0.215574724 | 0.172440322 | RORA | 1 |
| BP | GO:0043243 | positive regulation of protein-containing complex disassembly | 1/30 | 38/18805 | 0.058923076 | 0.215574724 | 0.172440322 | EIF5A2 | 1 |
| BP | GO:0070316 | regulation of G0 to G1 transition | 1/30 | 38/18805 | 0.058923076 | 0.215574724 | 0.172440322 | BCL7A | 1 |
| BP | GO:0009595 | detection of biotic stimulus | 1/30 | 39/18805 | 0.060427436 | 0.215574724 | 0.172440322 | TLR2 | 1 |
| BP | GO:0034389 | lipid droplet organization | 1/30 | 39/18805 | 0.060427436 | 0.215574724 | 0.172440322 | PNPLA2 | 1 |
| BP | GO:0070229 | negative regulation of lymphocyte apoptotic process | 1/30 | 39/18805 | 0.060427436 | 0.215574724 | 0.172440322 | IL7R | 1 |
| BP | GO:0034599 | cellular response to oxidative stress | 2/30 | 254/18805 | 0.061719127 | 0.215574724 | 0.172440322 | FUT8/MGST1 | 2 |
| BP | GO:0006958 | complement activation, classical pathway | 1/30 | 40/18805 | 0.06192947 | 0.215574724 | 0.172440322 | C1RL | 1 |
| BP | GO:0038179 | neurotrophin signaling pathway | 1/30 | 40/18805 | 0.06192947 | 0.215574724 | 0.172440322 | SORT1 | 1 |
| BP | GO:0045023 | G0 to G1 transition | 1/30 | 40/18805 | 0.06192947 | 0.215574724 | 0.172440322 | BCL7A | 1 |
| BP | GO:0052652 | cyclic purine nucleotide metabolic process | 1/30 | 40/18805 | 0.06192947 | 0.215574724 | 0.172440322 | RORA | 1 |
| BP | GO:0072337 | modified amino acid transport | 1/30 | 40/18805 | 0.06192947 | 0.215574724 | 0.172440322 | MGST1 | 1 |
| BP | GO:0090659 | walking behavior | 1/30 | 40/18805 | 0.06192947 | 0.215574724 | 0.172440322 | EPHA4 | 1 |
| BP | GO:0002478 | antigen processing and presentation of exogenous peptide antigen | 1/30 | 41/18805 | 0.063429183 | 0.215574724 | 0.172440322 | CTSD | 1 |
| BP | GO:0009187 | cyclic nucleotide metabolic process | 1/30 | 41/18805 | 0.063429183 | 0.215574724 | 0.172440322 | RORA | 1 |
| BP | GO:0018279 | protein N-linked glycosylation via asparagine | 1/30 | 41/18805 | 0.063429183 | 0.215574724 | 0.172440322 | FUT8 | 1 |
| BP | GO:0032728 | positive regulation of interferon-beta production | 1/30 | 41/18805 | 0.063429183 | 0.215574724 | 0.172440322 | TLR2 | 1 |
| BP | GO:0032814 | regulation of natural killer cell activation | 1/30 | 41/18805 | 0.063429183 | 0.215574724 | 0.172440322 | KLRC3 | 1 |
| BP | GO:0072539 | T-helper 17 cell differentiation | 1/30 | 41/18805 | 0.063429183 | 0.215574724 | 0.172440322 | RORA | 1 |
| BP | GO:1900016 | negative regulation of cytokine production involved in inflammatory response | 1/30 | 41/18805 | 0.063429183 | 0.215574724 | 0.172440322 | IL1R2 | 1 |
| BP | GO:0010001 | glial cell differentiation | 2/30 | 259/18805 | 0.063867153 | 0.215574724 | 0.172440322 | TLR2/EPHA4 | 2 |
| BP | GO:0002703 | regulation of leukocyte mediated immunity | 2/30 | 260/18805 | 0.064299832 | 0.215574724 | 0.172440322 | KLRC3/IL7R | 2 |
| BP | GO:0005996 | monosaccharide metabolic process | 2/30 | 260/18805 | 0.064299832 | 0.215574724 | 0.172440322 | FUT8/RORA | 2 |
| BP | GO:0032869 | cellular response to insulin stimulus | 2/30 | 260/18805 | 0.064299832 | 0.215574724 | 0.172440322 | EPHA4/CTSD | 2 |
| BP | GO:0032509 | endosome transport via multivesicular body sorting pathway | 1/30 | 42/18805 | 0.064926578 | 0.215574724 | 0.172440322 | SORT1 | 1 |
| BP | GO:0032733 | positive regulation of interleukin-10 production | 1/30 | 42/18805 | 0.064926578 | 0.215574724 | 0.172440322 | TLR2 | 1 |
| BP | GO:0044060 | regulation of endocrine process | 1/30 | 42/18805 | 0.064926578 | 0.215574724 | 0.172440322 | RETN | 1 |
| BP | GO:0046427 | positive regulation of receptor signaling pathway via JAK-STAT | 1/30 | 42/18805 | 0.064926578 | 0.215574724 | 0.172440322 | IL7R | 1 |
| BP | GO:0046461 | neutral lipid catabolic process | 1/30 | 42/18805 | 0.064926578 | 0.215574724 | 0.172440322 | PNPLA2 | 1 |
| BP | GO:0046464 | acylglycerol catabolic process | 1/30 | 42/18805 | 0.064926578 | 0.215574724 | 0.172440322 | PNPLA2 | 1 |
| BP | GO:0070232 | regulation of T cell apoptotic process | 1/30 | 42/18805 | 0.064926578 | 0.215574724 | 0.172440322 | IL7R | 1 |
| BP | GO:0099563 | modification of synaptic structure | 1/30 | 42/18805 | 0.064926578 | 0.215574724 | 0.172440322 | EPHA4 | 1 |
| BP | GO:0051347 | positive regulation of transferase activity | 2/30 | 263/18805 | 0.065603939 | 0.217051331 | 0.173621474 | S100A12/EPHA4 | 2 |
| BP | GO:0032570 | response to progesterone | 1/30 | 43/18805 | 0.066421659 | 0.21898024 | 0.175164428 | TLR2 | 1 |
| BP | GO:0032735 | positive regulation of interleukin-12 production | 1/30 | 44/18805 | 0.067914429 | 0.222330394 | 0.177844248 | TLR2 | 1 |
| BP | GO:0045494 | photoreceptor cell maintenance | 1/30 | 44/18805 | 0.067914429 | 0.222330394 | 0.177844248 | IQCB1 | 1 |
| BP | GO:0045687 | positive regulation of glial cell differentiation | 1/30 | 45/18805 | 0.069404892 | 0.223823365 | 0.17903849 | TLR2 | 1 |
| BP | GO:0048713 | regulation of oligodendrocyte differentiation | 1/30 | 45/18805 | 0.069404892 | 0.223823365 | 0.17903849 | TLR2 | 1 |
| BP | GO:0061001 | regulation of dendritic spine morphogenesis | 1/30 | 45/18805 | 0.069404892 | 0.223823365 | 0.17903849 | EPHA4 | 1 |
| BP | GO:0090207 | regulation of triglyceride metabolic process | 1/30 | 45/18805 | 0.069404892 | 0.223823365 | 0.17903849 | PNPLA2 | 1 |
| BP | GO:0009636 | response to toxic substance | 2/30 | 272/18805 | 0.069569963 | 0.223823365 | 0.17903849 | TLR2/MGST1 | 2 |
| BP | GO:0009225 | nucleotide-sugar metabolic process | 1/30 | 46/18805 | 0.07089305 | 0.224976924 | 0.179961234 | FUT8 | 1 |
| BP | GO:0021587 | cerebellum morphogenesis | 1/30 | 46/18805 | 0.07089305 | 0.224976924 | 0.179961234 | RORA | 1 |
| BP | GO:0031638 | zymogen activation | 1/30 | 46/18805 | 0.07089305 | 0.224976924 | 0.179961234 | C1RL | 1 |
| BP | GO:1904646 | cellular response to amyloid-beta | 1/30 | 46/18805 | 0.07089305 | 0.224976924 | 0.179961234 | EPHA4 | 1 |
| BP | GO:0003009 | skeletal muscle contraction | 1/30 | 47/18805 | 0.072378908 | 0.225602552 | 0.18046168 | TNNT1 | 1 |
| BP | GO:0006623 | protein targeting to vacuole | 1/30 | 47/18805 | 0.072378908 | 0.225602552 | 0.18046168 | SORT1 | 1 |
| BP | GO:0010824 | regulation of centrosome duplication | 1/30 | 47/18805 | 0.072378908 | 0.225602552 | 0.18046168 | XPO1 | 1 |
| BP | GO:0001881 | receptor recycling | 1/30 | 48/18805 | 0.073862469 | 0.225602552 | 0.18046168 | CTSD | 1 |
| BP | GO:0001914 | regulation of T cell mediated cytotoxicity | 1/30 | 48/18805 | 0.073862469 | 0.225602552 | 0.18046168 | IL7R | 1 |
| BP | GO:0008038 | neuron recognition | 1/30 | 48/18805 | 0.073862469 | 0.225602552 | 0.18046168 | EPHA4 | 1 |
| BP | GO:0071985 | multivesicular body sorting pathway | 1/30 | 48/18805 | 0.073862469 | 0.225602552 | 0.18046168 | SORT1 | 1 |
| BP | GO:1900271 | regulation of long-term synaptic potentiation | 1/30 | 48/18805 | 0.073862469 | 0.225602552 | 0.18046168 | EPHA4 | 1 |
| BP | GO:0014003 | oligodendrocyte development | 1/30 | 49/18805 | 0.075343736 | 0.225602552 | 0.18046168 | TLR2 | 1 |
| BP | GO:0021575 | hindbrain morphogenesis | 1/30 | 49/18805 | 0.075343736 | 0.225602552 | 0.18046168 | RORA | 1 |
| BP | GO:0036323 | vascular endothelial growth factor receptor-1 signaling pathway | 1/30 | 49/18805 | 0.075343736 | 0.225602552 | 0.18046168 | EPHA4 | 1 |
| BP | GO:0043029 | T cell homeostasis | 1/30 | 49/18805 | 0.075343736 | 0.225602552 | 0.18046168 | IL7R | 1 |
| BP | GO:1902003 | regulation of amyloid-beta formation | 1/30 | 49/18805 | 0.075343736 | 0.225602552 | 0.18046168 | EPHA4 | 1 |
| BP | GO:1902459 | positive regulation of stem cell population maintenance | 1/30 | 49/18805 | 0.075343736 | 0.225602552 | 0.18046168 | BCL7A | 1 |
| BP | GO:1904894 | positive regulation of receptor signaling pathway via STAT | 1/30 | 49/18805 | 0.075343736 | 0.225602552 | 0.18046168 | IL7R | 1 |
| BP | GO:0051962 | positive regulation of nervous system development | 2/30 | 285/18805 | 0.075436113 | 0.225602552 | 0.18046168 | TLR2/EPHA4 | 2 |
| BP | GO:0001774 | microglial cell activation | 1/30 | 50/18805 | 0.076822713 | 0.225602552 | 0.18046168 | TLR2 | 1 |
| BP | GO:0006693 | prostaglandin metabolic process | 1/30 | 50/18805 | 0.076822713 | 0.225602552 | 0.18046168 | TBXAS1 | 1 |
| BP | GO:0019884 | antigen processing and presentation of exogenous antigen | 1/30 | 50/18805 | 0.076822713 | 0.225602552 | 0.18046168 | CTSD | 1 |
| BP | GO:0006692 | prostanoid metabolic process | 1/30 | 51/18805 | 0.078299402 | 0.225602552 | 0.18046168 | TBXAS1 | 1 |
| BP | GO:0019320 | hexose catabolic process | 1/30 | 51/18805 | 0.078299402 | 0.225602552 | 0.18046168 | FUT8 | 1 |
| BP | GO:0045214 | sarcomere organization | 1/30 | 51/18805 | 0.078299402 | 0.225602552 | 0.18046168 | TNNT1 | 1 |
| BP | GO:1990090 | cellular response to nerve growth factor stimulus | 1/30 | 51/18805 | 0.078299402 | 0.225602552 | 0.18046168 | SORT1 | 1 |
| BP | GO:0006636 | unsaturated fatty acid biosynthetic process | 1/30 | 52/18805 | 0.079773809 | 0.225602552 | 0.18046168 | TBXAS1 | 1 |
| BP | GO:0006968 | cellular defense response | 1/30 | 52/18805 | 0.079773809 | 0.225602552 | 0.18046168 | KLRC3 | 1 |
| BP | GO:0032692 | negative regulation of interleukin-1 production | 1/30 | 52/18805 | 0.079773809 | 0.225602552 | 0.18046168 | IL1R2 | 1 |
| BP | GO:0034331 | cell junction maintenance | 1/30 | 52/18805 | 0.079773809 | 0.225602552 | 0.18046168 | SORT1 | 1 |
| BP | GO:0036215 | response to stem cell factor | 1/30 | 52/18805 | 0.079773809 | 0.225602552 | 0.18046168 | EPHA4 | 1 |
| BP | GO:0036216 | cellular response to stem cell factor stimulus | 1/30 | 52/18805 | 0.079773809 | 0.225602552 | 0.18046168 | EPHA4 | 1 |
| BP | GO:0038109 | Kit signaling pathway | 1/30 | 52/18805 | 0.079773809 | 0.225602552 | 0.18046168 | EPHA4 | 1 |
| BP | GO:0042572 | retinol metabolic process | 1/30 | 52/18805 | 0.079773809 | 0.225602552 | 0.18046168 | PNPLA2 | 1 |
| BP | GO:1903532 | positive regulation of secretion by cell | 2/30 | 297/18805 | 0.080988554 | 0.225602552 | 0.18046168 | TLR2/RETN | 2 |
| BP | GO:0002269 | leukocyte activation involved in inflammatory response | 1/30 | 53/18805 | 0.081245935 | 0.225602552 | 0.18046168 | TLR2 | 1 |
| BP | GO:0021695 | cerebellar cortex development | 1/30 | 53/18805 | 0.081245935 | 0.225602552 | 0.18046168 | RORA | 1 |
| BP | GO:0022602 | ovulation cycle process | 1/30 | 53/18805 | 0.081245935 | 0.225602552 | 0.18046168 | RETN | 1 |
| BP | GO:0031648 | protein destabilization | 1/30 | 53/18805 | 0.081245935 | 0.225602552 | 0.18046168 | PYHIN1 | 1 |
| BP | GO:0042269 | regulation of natural killer cell mediated cytotoxicity | 1/30 | 53/18805 | 0.081245935 | 0.225602552 | 0.18046168 | KLRC3 | 1 |
| BP | GO:0046460 | neutral lipid biosynthetic process | 1/30 | 53/18805 | 0.081245935 | 0.225602552 | 0.18046168 | PNPLA2 | 1 |
| BP | GO:0046463 | acylglycerol biosynthetic process | 1/30 | 53/18805 | 0.081245935 | 0.225602552 | 0.18046168 | PNPLA2 | 1 |
| BP | GO:0090102 | cochlea development | 1/30 | 53/18805 | 0.081245935 | 0.225602552 | 0.18046168 | EPHA4 | 1 |
| BP | GO:0140353 | lipid export from cell | 1/30 | 53/18805 | 0.081245935 | 0.225602552 | 0.18046168 | RETN | 1 |
| BP | GO:1990089 | response to nerve growth factor | 1/30 | 53/18805 | 0.081245935 | 0.225602552 | 0.18046168 | SORT1 | 1 |
| BP | GO:0043122 | regulation of canonical NF-kappaB signal transduction | 2/30 | 300/18805 | 0.082396491 | 0.225654466 | 0.180503206 | S100A12/RORA | 2 |
| BP | GO:0002455 | humoral immune response mediated by circulating immunoglobulin | 1/30 | 54/18805 | 0.082715785 | 0.225654466 | 0.180503206 | C1RL | 1 |
| BP | GO:0002548 | monocyte chemotaxis | 1/30 | 54/18805 | 0.082715785 | 0.225654466 | 0.180503206 | S100A12 | 1 |
| BP | GO:0009395 | phospholipid catabolic process | 1/30 | 54/18805 | 0.082715785 | 0.225654466 | 0.180503206 | PLBD1 | 1 |
| BP | GO:0031103 | axon regeneration | 1/30 | 54/18805 | 0.082715785 | 0.225654466 | 0.180503206 | EPHA4 | 1 |
| BP | GO:0038145 | macrophage colony-stimulating factor signaling pathway | 1/30 | 54/18805 | 0.082715785 | 0.225654466 | 0.180503206 | EPHA4 | 1 |
| BP | GO:0048511 | rhythmic process | 2/30 | 303/18805 | 0.083812157 | 0.225698495 | 0.180538425 | RORA/RETN | 2 |
| BP | GO:0010883 | regulation of lipid storage | 1/30 | 55/18805 | 0.084183361 | 0.225698495 | 0.180538425 | PNPLA2 | 1 |
| BP | GO:0031547 | brain-derived neurotrophic factor receptor signaling pathway | 1/30 | 55/18805 | 0.084183361 | 0.225698495 | 0.180538425 | EPHA4 | 1 |
| BP | GO:0035790 | platelet-derived growth factor receptor-alpha signaling pathway | 1/30 | 55/18805 | 0.084183361 | 0.225698495 | 0.180538425 | EPHA4 | 1 |
| BP | GO:0046365 | monosaccharide catabolic process | 1/30 | 55/18805 | 0.084183361 | 0.225698495 | 0.180538425 | FUT8 | 1 |
| BP | GO:0072538 | T-helper 17 type immune response | 1/30 | 55/18805 | 0.084183361 | 0.225698495 | 0.180538425 | RORA | 1 |
| BP | GO:0002758 | innate immune response-activating signaling pathway | 2/30 | 305/18805 | 0.084760178 | 0.225817173 | 0.180633356 | KLRC3/TLR2 | 2 |
| BP | GO:0010719 | negative regulation of epithelial to mesenchymal transition | 1/30 | 56/18805 | 0.085648668 | 0.225817173 | 0.180633356 | EPHA4 | 1 |
| BP | GO:0010761 | fibroblast migration | 1/30 | 56/18805 | 0.085648668 | 0.225817173 | 0.180633356 | FUT8 | 1 |
| BP | GO:0046605 | regulation of centrosome cycle | 1/30 | 56/18805 | 0.085648668 | 0.225817173 | 0.180633356 | XPO1 | 1 |
| BP | GO:0006959 | humoral immune response | 2/30 | 309/18805 | 0.086666273 | 0.225817173 | 0.180633356 | S100A12/C1RL | 2 |
| BP | GO:0001895 | retina homeostasis | 1/30 | 57/18805 | 0.087111708 | 0.225817173 | 0.180633356 | IQCB1 | 1 |
| BP | GO:0014888 | striated muscle adaptation | 1/30 | 57/18805 | 0.087111708 | 0.225817173 | 0.180633356 | TNNT1 | 1 |
| BP | GO:0034205 | amyloid-beta formation | 1/30 | 57/18805 | 0.087111708 | 0.225817173 | 0.180633356 | EPHA4 | 1 |
| BP | GO:1902991 | regulation of amyloid precursor protein catabolic process | 1/30 | 57/18805 | 0.087111708 | 0.225817173 | 0.180633356 | EPHA4 | 1 |
| BP | GO:0051146 | striated muscle cell differentiation | 2/30 | 311/18805 | 0.087624289 | 0.225817173 | 0.180633356 | SORT1/TNNT1 | 2 |
| BP | GO:0038063 | collagen-activated tyrosine kinase receptor signaling pathway | 1/30 | 58/18805 | 0.088572485 | 0.225817173 | 0.180633356 | EPHA4 | 1 |
| BP | GO:1904645 | response to amyloid-beta | 1/30 | 58/18805 | 0.088572485 | 0.225817173 | 0.180633356 | EPHA4 | 1 |
| BP | GO:0050807 | regulation of synapse organization | 2/30 | 315/18805 | 0.089550112 | 0.225817173 | 0.180633356 | TLR2/EPHA4 | 2 |
| BP | GO:0001913 | T cell mediated cytotoxicity | 1/30 | 59/18805 | 0.090031003 | 0.225817173 | 0.180633356 | IL7R | 1 |
| BP | GO:0002715 | regulation of natural killer cell mediated immunity | 1/30 | 59/18805 | 0.090031003 | 0.225817173 | 0.180633356 | KLRC3 | 1 |
| BP | GO:0019369 | arachidonate metabolic process | 1/30 | 59/18805 | 0.090031003 | 0.225817173 | 0.180633356 | TBXAS1 | 1 |
| BP | GO:0034332 | adherens junction organization | 1/30 | 59/18805 | 0.090031003 | 0.225817173 | 0.180633356 | EPHA4 | 1 |
| BP | GO:0060986 | endocrine hormone secretion | 1/30 | 59/18805 | 0.090031003 | 0.225817173 | 0.180633356 | RETN | 1 |
| BP | GO:0061462 | protein localization to lysosome | 1/30 | 59/18805 | 0.090031003 | 0.225817173 | 0.180633356 | SORT1 | 1 |
| BP | GO:0061900 | glial cell activation | 1/30 | 59/18805 | 0.090031003 | 0.225817173 | 0.180633356 | TLR2 | 1 |
| BP | GO:2000107 | negative regulation of leukocyte apoptotic process | 1/30 | 59/18805 | 0.090031003 | 0.225817173 | 0.180633356 | IL7R | 1 |
| BP | GO:0042254 | ribosome biogenesis | 2/30 | 317/18805 | 0.090517861 | 0.225817173 | 0.180633356 | SDAD1/XPO1 | 2 |
| BP | GO:0032608 | interferon-beta production | 1/30 | 60/18805 | 0.091487264 | 0.225817173 | 0.180633356 | TLR2 | 1 |
| BP | GO:0032648 | regulation of interferon-beta production | 1/30 | 60/18805 | 0.091487264 | 0.225817173 | 0.180633356 | TLR2 | 1 |
| BP | GO:0048012 | hepatocyte growth factor receptor signaling pathway | 1/30 | 60/18805 | 0.091487264 | 0.225817173 | 0.180633356 | EPHA4 | 1 |
| BP | GO:0050881 | musculoskeletal movement | 1/30 | 60/18805 | 0.091487264 | 0.225817173 | 0.180633356 | TNNT1 | 1 |
| BP | GO:0060997 | dendritic spine morphogenesis | 1/30 | 60/18805 | 0.091487264 | 0.225817173 | 0.180633356 | EPHA4 | 1 |
| BP | GO:0070613 | regulation of protein processing | 1/30 | 60/18805 | 0.091487264 | 0.225817173 | 0.180633356 | IL1R2 | 1 |
| BP | GO:0050803 | regulation of synapse structure or activity | 2/30 | 321/18805 | 0.092462892 | 0.225817173 | 0.180633356 | TLR2/EPHA4 | 2 |
| BP | GO:0051047 | positive regulation of secretion | 2/30 | 321/18805 | 0.092462892 | 0.225817173 | 0.180633356 | TLR2/RETN | 2 |
| BP | GO:0031102 | neuron projection regeneration | 1/30 | 61/18805 | 0.092941272 | 0.225817173 | 0.180633356 | EPHA4 | 1 |
| BP | GO:0045604 | regulation of epidermal cell differentiation | 1/30 | 61/18805 | 0.092941272 | 0.225817173 | 0.180633356 | MAFG | 1 |
| BP | GO:0046456 | icosanoid biosynthetic process | 1/30 | 61/18805 | 0.092941272 | 0.225817173 | 0.180633356 | TBXAS1 | 1 |
| BP | GO:0070231 | T cell apoptotic process | 1/30 | 61/18805 | 0.092941272 | 0.225817173 | 0.180633356 | IL7R | 1 |
| BP | GO:0034504 | protein localization to nucleus | 2/30 | 322/18805 | 0.092951115 | 0.225817173 | 0.180633356 | PYHIN1/XPO1 | 2 |
| BP | GO:0062197 | cellular response to chemical stress | 2/30 | 323/18805 | 0.093440117 | 0.225817173 | 0.180633356 | FUT8/MGST1 | 2 |
| BP | GO:0002823 | negative regulation of adaptive immune response based on somatic recombination of immune receptors built from immunoglobulin superfamily domains | 1/30 | 62/18805 | 0.09439303 | 0.225817173 | 0.180633356 | IL7R | 1 |
| BP | GO:0010574 | regulation of vascular endothelial growth factor production | 1/30 | 62/18805 | 0.09439303 | 0.225817173 | 0.180633356 | RORA | 1 |
| BP | GO:0031663 | lipopolysaccharide-mediated signaling pathway | 1/30 | 62/18805 | 0.09439303 | 0.225817173 | 0.180633356 | TLR2 | 1 |
| BP | GO:0038065 | collagen-activated signaling pathway | 1/30 | 62/18805 | 0.09439303 | 0.225817173 | 0.180633356 | EPHA4 | 1 |
| BP | GO:0050879 | multicellular organismal movement | 1/30 | 62/18805 | 0.09439303 | 0.225817173 | 0.180633356 | TNNT1 | 1 |
| BP | GO:0070228 | regulation of lymphocyte apoptotic process | 1/30 | 62/18805 | 0.09439303 | 0.225817173 | 0.180633356 | IL7R | 1 |
| BP | GO:0006611 | protein export from nucleus | 1/30 | 63/18805 | 0.095842543 | 0.226646917 | 0.181297077 | XPO1 | 1 |
| BP | GO:0008347 | glial cell migration | 1/30 | 63/18805 | 0.095842543 | 0.226646917 | 0.181297077 | EPHA4 | 1 |
| BP | GO:0035791 | platelet-derived growth factor receptor-beta signaling pathway | 1/30 | 63/18805 | 0.095842543 | 0.226646917 | 0.181297077 | EPHA4 | 1 |
| BP | GO:0043030 | regulation of macrophage activation | 1/30 | 63/18805 | 0.095842543 | 0.226646917 | 0.181297077 | RORA | 1 |
| BP | GO:0009311 | oligosaccharide metabolic process | 1/30 | 64/18805 | 0.097289812 | 0.226646917 | 0.181297077 | FUT8 | 1 |
| BP | GO:0051090 | regulation of DNA-binding transcription factor activity | 2/30 | 332/18805 | 0.097875614 | 0.226646917 | 0.181297077 | S100A12/TLR2 | 2 |
| BP | GO:0002707 | negative regulation of lymphocyte mediated immunity | 1/30 | 65/18805 | 0.098734842 | 0.226646917 | 0.181297077 | IL7R | 1 |
| BP | GO:0006956 | complement activation | 1/30 | 65/18805 | 0.098734842 | 0.226646917 | 0.181297077 | C1RL | 1 |
| BP | GO:0010573 | vascular endothelial growth factor production | 1/30 | 65/18805 | 0.098734842 | 0.226646917 | 0.181297077 | RORA | 1 |
| BP | GO:0032615 | interleukin-12 production | 1/30 | 65/18805 | 0.098734842 | 0.226646917 | 0.181297077 | TLR2 | 1 |
| BP | GO:0032655 | regulation of interleukin-12 production | 1/30 | 65/18805 | 0.098734842 | 0.226646917 | 0.181297077 | TLR2 | 1 |
| BP | GO:0032757 | positive regulation of interleukin-8 production | 1/30 | 65/18805 | 0.098734842 | 0.226646917 | 0.181297077 | TLR2 | 1 |
| BP | GO:0042273 | ribosomal large subunit biogenesis | 1/30 | 65/18805 | 0.098734842 | 0.226646917 | 0.181297077 | SDAD1 | 1 |
| BP | GO:0048814 | regulation of dendrite morphogenesis | 1/30 | 65/18805 | 0.098734842 | 0.226646917 | 0.181297077 | EPHA4 | 1 |
| BP | GO:0072348 | sulfur compound transport | 1/30 | 65/18805 | 0.098734842 | 0.226646917 | 0.181297077 | MGST1 | 1 |
| BP | GO:0072666 | establishment of protein localization to vacuole | 1/30 | 65/18805 | 0.098734842 | 0.226646917 | 0.181297077 | SORT1 | 1 |
| BP | GO:0051336 | regulation of hydrolase activity | 2/30 | 334/18805 | 0.098869555 | 0.226646917 | 0.181297077 | SORT1/EPHA4 | 2 |
| BP | GO:0032613 | interleukin-10 production | 1/30 | 66/18805 | 0.100177636 | 0.226858578 | 0.181466387 | TLR2 | 1 |
| BP | GO:0032653 | regulation of interleukin-10 production | 1/30 | 66/18805 | 0.100177636 | 0.226858578 | 0.181466387 | TLR2 | 1 |
| BP | GO:0052548 | regulation of endopeptidase activity | 1/30 | 66/18805 | 0.100177636 | 0.226858578 | 0.181466387 | EPHA4 | 1 |
| BP | GO:1901224 | positive regulation of non-canonical NF-kappaB signal transduction | 1/30 | 66/18805 | 0.100177636 | 0.226858578 | 0.181466387 | TLR2 | 1 |
| BP | GO:1903317 | regulation of protein maturation | 1/30 | 66/18805 | 0.100177636 | 0.226858578 | 0.181466387 | IL1R2 | 1 |
| BP | GO:0002820 | negative regulation of adaptive immune response | 1/30 | 67/18805 | 0.101618197 | 0.22737738 | 0.181881381 | IL7R | 1 |
| BP | GO:0032922 | circadian regulation of gene expression | 1/30 | 67/18805 | 0.101618197 | 0.22737738 | 0.181881381 | RORA | 1 |
| BP | GO:0050832 | defense response to fungus | 1/30 | 67/18805 | 0.101618197 | 0.22737738 | 0.181881381 | S100A12 | 1 |
| BP | GO:0050994 | regulation of lipid catabolic process | 1/30 | 67/18805 | 0.101618197 | 0.22737738 | 0.181881381 | PNPLA2 | 1 |
| BP | GO:0006913 | nucleocytoplasmic transport | 2/30 | 340/18805 | 0.101868965 | 0.22737738 | 0.181881381 | SDAD1/XPO1 | 2 |
| BP | GO:0051169 | nuclear transport | 2/30 | 340/18805 | 0.101868965 | 0.22737738 | 0.181881381 | SDAD1/XPO1 | 2 |
| BP | GO:0001912 | positive regulation of leukocyte mediated cytotoxicity | 1/30 | 68/18805 | 0.103056528 | 0.228388933 | 0.182690532 | KLRC3 | 1 |
| BP | GO:0045682 | regulation of epidermis development | 1/30 | 68/18805 | 0.103056528 | 0.228388933 | 0.182690532 | MAFG | 1 |
| BP | GO:0060760 | positive regulation of response to cytokine stimulus | 1/30 | 68/18805 | 0.103056528 | 0.228388933 | 0.182690532 | TLR2 | 1 |
| BP | GO:0019216 | regulation of lipid metabolic process | 2/30 | 343/18805 | 0.103378395 | 0.228559343 | 0.182826845 | PNPLA2/RORA | 2 |
| BP | GO:0019229 | regulation of vasoconstriction | 1/30 | 69/18805 | 0.104492633 | 0.228853584 | 0.183062212 | TBXAS1 | 1 |
| BP | GO:0045576 | mast cell activation | 1/30 | 69/18805 | 0.104492633 | 0.228853584 | 0.183062212 | S100A12 | 1 |
| BP | GO:0046503 | glycerolipid catabolic process | 1/30 | 69/18805 | 0.104492633 | 0.228853584 | 0.183062212 | PNPLA2 | 1 |
| BP | GO:1905953 | negative regulation of lipid localization | 1/30 | 69/18805 | 0.104492633 | 0.228853584 | 0.183062212 | PNPLA2 | 1 |
| BP | GO:0042063 | gliogenesis | 2/30 | 348/18805 | 0.105908204 | 0.230679215 | 0.184522551 | TLR2/EPHA4 | 2 |
| BP | GO:0008333 | endosome to lysosome transport | 1/30 | 71/18805 | 0.107358179 | 0.230679215 | 0.184522551 | SORT1 | 1 |
| BP | GO:0030225 | macrophage differentiation | 1/30 | 71/18805 | 0.107358179 | 0.230679215 | 0.184522551 | TLR2 | 1 |
| BP | GO:0031343 | positive regulation of cell killing | 1/30 | 71/18805 | 0.107358179 | 0.230679215 | 0.184522551 | KLRC3 | 1 |
| BP | GO:0042987 | amyloid precursor protein catabolic process | 1/30 | 71/18805 | 0.107358179 | 0.230679215 | 0.184522551 | EPHA4 | 1 |
| BP | GO:0043406 | positive regulation of MAP kinase activity | 1/30 | 71/18805 | 0.107358179 | 0.230679215 | 0.184522551 | S100A12 | 1 |
| BP | GO:0048002 | antigen processing and presentation of peptide antigen | 1/30 | 71/18805 | 0.107358179 | 0.230679215 | 0.184522551 | CTSD | 1 |
| BP | GO:0050435 | amyloid-beta metabolic process | 1/30 | 71/18805 | 0.107358179 | 0.230679215 | 0.184522551 | EPHA4 | 1 |
| BP | GO:0006414 | translational elongation | 1/30 | 72/18805 | 0.108787625 | 0.230679215 | 0.184522551 | EIF5A2 | 1 |
| BP | GO:0043124 | negative regulation of canonical NF-kappaB signal transduction | 1/30 | 72/18805 | 0.108787625 | 0.230679215 | 0.184522551 | RORA | 1 |
| BP | GO:0046626 | regulation of insulin receptor signaling pathway | 1/30 | 72/18805 | 0.108787625 | 0.230679215 | 0.184522551 | CTSD | 1 |
| BP | GO:0051101 | regulation of DNA binding | 1/30 | 72/18805 | 0.108787625 | 0.230679215 | 0.184522551 | PYHIN1 | 1 |
| BP | GO:0140895 | cell surface toll-like receptor signaling pathway | 1/30 | 72/18805 | 0.108787625 | 0.230679215 | 0.184522551 | TLR2 | 1 |
| BP | GO:1903008 | organelle disassembly | 1/30 | 72/18805 | 0.108787625 | 0.230679215 | 0.184522551 | PNPLA2 | 1 |
| BP | GO:0006487 | protein N-linked glycosylation | 1/30 | 73/18805 | 0.110214859 | 0.231600143 | 0.185259211 | FUT8 | 1 |
| BP | GO:0014015 | positive regulation of gliogenesis | 1/30 | 73/18805 | 0.110214859 | 0.231600143 | 0.185259211 | TLR2 | 1 |
| BP | GO:0032722 | positive regulation of chemokine production | 1/30 | 73/18805 | 0.110214859 | 0.231600143 | 0.185259211 | TLR2 | 1 |
| BP | GO:0051898 | negative regulation of phosphatidylinositol 3-kinase/protein kinase B signal transduction | 1/30 | 73/18805 | 0.110214859 | 0.231600143 | 0.185259211 | OTUD3 | 1 |
| BP | GO:0006635 | fatty acid beta-oxidation | 1/30 | 74/18805 | 0.111639884 | 0.23302016 | 0.186395096 | CRAT | 1 |
| BP | GO:1903051 | negative regulation of proteolysis involved in protein catabolic process | 1/30 | 74/18805 | 0.111639884 | 0.23302016 | 0.186395096 | EPHA4 | 1 |
| BP | GO:2000036 | regulation of stem cell population maintenance | 1/30 | 74/18805 | 0.111639884 | 0.23302016 | 0.186395096 | BCL7A | 1 |
| BP | GO:0006406 | mRNA export from nucleus | 1/30 | 75/18805 | 0.113062701 | 0.234416668 | 0.187512176 | XPO1 | 1 |
| BP | GO:0030239 | myofibril assembly | 1/30 | 75/18805 | 0.113062701 | 0.234416668 | 0.187512176 | TNNT1 | 1 |
| BP | GO:0042698 | ovulation cycle | 1/30 | 75/18805 | 0.113062701 | 0.234416668 | 0.187512176 | RETN | 1 |
| BP | GO:0002704 | negative regulation of leukocyte mediated immunity | 1/30 | 76/18805 | 0.114483316 | 0.234753702 | 0.187781773 | IL7R | 1 |
| BP | GO:0032481 | positive regulation of type I interferon production | 1/30 | 76/18805 | 0.114483316 | 0.234753702 | 0.187781773 | TLR2 | 1 |
| BP | GO:0045806 | negative regulation of endocytosis | 1/30 | 76/18805 | 0.114483316 | 0.234753702 | 0.187781773 | TLR2 | 1 |
| BP | GO:0050795 | regulation of behavior | 1/30 | 76/18805 | 0.114483316 | 0.234753702 | 0.187781773 | RETN | 1 |
| BP | GO:0051298 | centrosome duplication | 1/30 | 76/18805 | 0.114483316 | 0.234753702 | 0.187781773 | XPO1 | 1 |
| BP | GO:1900076 | regulation of cellular response to insulin stimulus | 1/30 | 77/18805 | 0.115901732 | 0.236409607 | 0.189106348 | CTSD | 1 |
| BP | GO:0045859 | regulation of protein kinase activity | 2/30 | 369/18805 | 0.116717082 | 0.236409607 | 0.189106348 | S100A12/EPHA4 | 2 |
| BP | GO:0071375 | cellular response to peptide hormone stimulus | 2/30 | 369/18805 | 0.116717082 | 0.236409607 | 0.189106348 | EPHA4/CTSD | 2 |
| BP | GO:0044282 | small molecule catabolic process | 2/30 | 370/18805 | 0.117238872 | 0.236409607 | 0.189106348 | FUT8/CRAT | 2 |
| BP | GO:0007585 | respiratory gaseous exchange by respiratory system | 1/30 | 78/18805 | 0.117317951 | 0.236409607 | 0.189106348 | FUT8 | 1 |
| BP | GO:0009620 | response to fungus | 1/30 | 78/18805 | 0.117317951 | 0.236409607 | 0.189106348 | S100A12 | 1 |
| BP | GO:0046425 | regulation of receptor signaling pathway via JAK-STAT | 1/30 | 78/18805 | 0.117317951 | 0.236409607 | 0.189106348 | IL7R | 1 |
| BP | GO:0070373 | negative regulation of ERK1 and ERK2 cascade | 1/30 | 78/18805 | 0.117317951 | 0.236409607 | 0.189106348 | EPHA4 | 1 |
| BP | GO:0050767 | regulation of neurogenesis | 2/30 | 372/18805 | 0.118284306 | 0.237843228 | 0.190253115 | TLR2/EPHA4 | 2 |
| BP | GO:0002534 | cytokine production involved in inflammatory response | 1/30 | 80/18805 | 0.120143813 | 0.240030358 | 0.192002622 | IL1R2 | 1 |
| BP | GO:0006809 | nitric oxide biosynthetic process | 1/30 | 80/18805 | 0.120143813 | 0.240030358 | 0.192002622 | RORA | 1 |
| BP | GO:1900015 | regulation of cytokine production involved in inflammatory response | 1/30 | 80/18805 | 0.120143813 | 0.240030358 | 0.192002622 | IL1R2 | 1 |
| BP | GO:0002260 | lymphocyte homeostasis | 1/30 | 81/18805 | 0.121553462 | 0.241296553 | 0.193015464 | IL7R | 1 |
| BP | GO:0002752 | cell surface pattern recognition receptor signaling pathway | 1/30 | 81/18805 | 0.121553462 | 0.241296553 | 0.193015464 | TLR2 | 1 |
| BP | GO:0043954 | cellular component maintenance | 1/30 | 81/18805 | 0.121553462 | 0.241296553 | 0.193015464 | SORT1 | 1 |
| BP | GO:0002224 | toll-like receptor signaling pathway | 1/30 | 82/18805 | 0.122960928 | 0.243572284 | 0.194835843 | TLR2 | 1 |
| BP | GO:0006289 | nucleotide-excision repair | 1/30 | 83/18805 | 0.124366214 | 0.244796789 | 0.195815336 | BCL7A | 1 |
| BP | GO:0042267 | natural killer cell mediated cytotoxicity | 1/30 | 83/18805 | 0.124366214 | 0.244796789 | 0.195815336 | KLRC3 | 1 |
| BP | GO:0046323 | D-glucose import | 1/30 | 83/18805 | 0.124366214 | 0.244796789 | 0.195815336 | SORT1 | 1 |
| BP | GO:0001523 | retinoid metabolic process | 1/30 | 84/18805 | 0.125769324 | 0.246001633 | 0.196779103 | PNPLA2 | 1 |
| BP | GO:0008344 | adult locomotory behavior | 1/30 | 84/18805 | 0.125769324 | 0.246001633 | 0.196779103 | EPHA4 | 1 |
| BP | GO:0030593 | neutrophil chemotaxis | 1/30 | 84/18805 | 0.125769324 | 0.246001633 | 0.196779103 | S100A12 | 1 |
| BP | GO:0048013 | ephrin receptor signaling pathway | 1/30 | 85/18805 | 0.127170259 | 0.24770324 | 0.198140235 | EPHA4 | 1 |
| BP | GO:0070227 | lymphocyte apoptotic process | 1/30 | 85/18805 | 0.127170259 | 0.24770324 | 0.198140235 | IL7R | 1 |
| BP | GO:0008625 | extrinsic apoptotic signaling pathway via death domain receptors | 1/30 | 86/18805 | 0.128569025 | 0.248353831 | 0.198660649 | SORT1 | 1 |
| BP | GO:0034248 | regulation of amide metabolic process | 1/30 | 86/18805 | 0.128569025 | 0.248353831 | 0.198660649 | EPHA4 | 1 |
| BP | GO:0042093 | T-helper cell differentiation | 1/30 | 86/18805 | 0.128569025 | 0.248353831 | 0.198660649 | RORA | 1 |
| BP | GO:0097061 | dendritic spine organization | 1/30 | 86/18805 | 0.128569025 | 0.248353831 | 0.198660649 | EPHA4 | 1 |
| BP | GO:0008589 | regulation of smoothened signaling pathway | 1/30 | 87/18805 | 0.129965624 | 0.248989583 | 0.199169193 | RORA | 1 |
| BP | GO:0048678 | response to axon injury | 1/30 | 87/18805 | 0.129965624 | 0.248989583 | 0.199169193 | EPHA4 | 1 |
| BP | GO:0052547 | regulation of peptidase activity | 1/30 | 87/18805 | 0.129965624 | 0.248989583 | 0.199169193 | EPHA4 | 1 |
| BP | GO:0150076 | neuroinflammatory response | 1/30 | 87/18805 | 0.129965624 | 0.248989583 | 0.199169193 | TLR2 | 1 |
| BP | GO:0002228 | natural killer cell mediated immunity | 1/30 | 88/18805 | 0.131360059 | 0.24921112 | 0.199346403 | KLRC3 | 1 |
| BP | GO:0002294 | CD4-positive, alpha-beta T cell differentiation involved in immune response | 1/30 | 88/18805 | 0.131360059 | 0.24921112 | 0.199346403 | RORA | 1 |
| BP | GO:0032370 | positive regulation of lipid transport | 1/30 | 88/18805 | 0.131360059 | 0.24921112 | 0.199346403 | RETN | 1 |
| BP | GO:0072665 | protein localization to vacuole | 1/30 | 88/18805 | 0.131360059 | 0.24921112 | 0.199346403 | SORT1 | 1 |
| BP | GO:0043549 | regulation of kinase activity | 2/30 | 397/18805 | 0.131551234 | 0.24921112 | 0.199346403 | S100A12/EPHA4 | 2 |
| BP | GO:0002287 | alpha-beta T cell activation involved in immune response | 1/30 | 89/18805 | 0.132752333 | 0.24921112 | 0.199346403 | RORA | 1 |
| BP | GO:0002293 | alpha-beta T cell differentiation involved in immune response | 1/30 | 89/18805 | 0.132752333 | 0.24921112 | 0.199346403 | RORA | 1 |
| BP | GO:0016101 | diterpenoid metabolic process | 1/30 | 89/18805 | 0.132752333 | 0.24921112 | 0.199346403 | PNPLA2 | 1 |
| BP | GO:0033077 | T cell differentiation in thymus | 1/30 | 89/18805 | 0.132752333 | 0.24921112 | 0.199346403 | IL7R | 1 |
| BP | GO:0042310 | vasoconstriction | 1/30 | 89/18805 | 0.132752333 | 0.24921112 | 0.199346403 | TBXAS1 | 1 |
| BP | GO:0061515 | myeloid cell development | 1/30 | 90/18805 | 0.13414245 | 0.250811434 | 0.20062651 | TLR2 | 1 |
| BP | GO:2000243 | positive regulation of reproductive process | 1/30 | 90/18805 | 0.13414245 | 0.250811434 | 0.20062651 | RETN | 1 |
| BP | GO:0001701 | in utero embryonic development | 2/30 | 403/18805 | 0.134787048 | 0.251512632 | 0.201187405 | FUT8/MAFG | 2 |
| BP | GO:0048708 | astrocyte differentiation | 1/30 | 91/18805 | 0.135530413 | 0.25239496 | 0.201893188 | EPHA4 | 1 |
| BP | GO:0006979 | response to oxidative stress | 2/30 | 408/18805 | 0.137497893 | 0.255512469 | 0.204386913 | FUT8/MGST1 | 2 |
| BP | GO:0030071 | regulation of mitotic metaphase/anaphase transition | 1/30 | 93/18805 | 0.138299889 | 0.255512469 | 0.204386913 | BCL7A | 1 |
| BP | GO:0042100 | B cell proliferation | 1/30 | 93/18805 | 0.138299889 | 0.255512469 | 0.204386913 | IL7R | 1 |
| BP | GO:0097194 | execution phase of apoptosis | 1/30 | 93/18805 | 0.138299889 | 0.255512469 | 0.204386913 | CTSD | 1 |
| BP | GO:0021954 | central nervous system neuron development | 1/30 | 94/18805 | 0.139681409 | 0.256036847 | 0.204806368 | EPHA4 | 1 |
| BP | GO:0050886 | endocrine process | 1/30 | 94/18805 | 0.139681409 | 0.256036847 | 0.204806368 | RETN | 1 |
| BP | GO:1900182 | positive regulation of protein localization to nucleus | 1/30 | 94/18805 | 0.139681409 | 0.256036847 | 0.204806368 | PYHIN1 | 1 |
| BP | GO:2000781 | positive regulation of double-strand break repair | 1/30 | 94/18805 | 0.139681409 | 0.256036847 | 0.204806368 | BCL7A | 1 |
| BP | GO:0006631 | fatty acid metabolic process | 2/30 | 413/18805 | 0.140221384 | 0.25605003 | 0.204816914 | CRAT/TBXAS1 | 2 |
| BP | GO:0001934 | positive regulation of protein phosphorylation | 2/30 | 414/18805 | 0.140767568 | 0.25605003 | 0.204816914 | S100A12/EPHA4 | 2 |
| BP | GO:0002697 | regulation of immune effector process | 2/30 | 414/18805 | 0.140767568 | 0.25605003 | 0.204816914 | KLRC3/IL7R | 2 |
| BP | GO:0038084 | vascular endothelial growth factor signaling pathway | 1/30 | 95/18805 | 0.141060788 | 0.25605003 | 0.204816914 | EPHA4 | 1 |
| BP | GO:2000106 | regulation of leukocyte apoptotic process | 1/30 | 95/18805 | 0.141060788 | 0.25605003 | 0.204816914 | IL7R | 1 |
| BP | GO:0006405 | RNA export from nucleus | 1/30 | 96/18805 | 0.142438029 | 0.257045316 | 0.205613053 | XPO1 | 1 |
| BP | GO:0106027 | neuron projection organization | 1/30 | 96/18805 | 0.142438029 | 0.257045316 | 0.205613053 | EPHA4 | 1 |
| BP | GO:1902099 | regulation of metaphase/anaphase transition of cell cycle | 1/30 | 96/18805 | 0.142438029 | 0.257045316 | 0.205613053 | BCL7A | 1 |
| BP | GO:0002292 | T cell differentiation involved in immune response | 1/30 | 97/18805 | 0.143813135 | 0.257045316 | 0.205613053 | RORA | 1 |
| BP | GO:0007091 | metaphase/anaphase transition of mitotic cell cycle | 1/30 | 97/18805 | 0.143813135 | 0.257045316 | 0.205613053 | BCL7A | 1 |
| BP | GO:0019915 | lipid storage | 1/30 | 97/18805 | 0.143813135 | 0.257045316 | 0.205613053 | PNPLA2 | 1 |
| BP | GO:0170062 | nutrient storage | 1/30 | 97/18805 | 0.143813135 | 0.257045316 | 0.205613053 | PNPLA2 | 1 |
| BP | GO:1904892 | regulation of receptor signaling pathway via STAT | 1/30 | 97/18805 | 0.143813135 | 0.257045316 | 0.205613053 | IL7R | 1 |
| BP | GO:0042157 | lipoprotein metabolic process | 1/30 | 98/18805 | 0.14518611 | 0.258016458 | 0.206389878 | CTSD | 1 |
| BP | GO:0043502 | regulation of muscle adaptation | 1/30 | 98/18805 | 0.14518611 | 0.258016458 | 0.206389878 | TNNT1 | 1 |
| BP | GO:0071346 | cellular response to type II interferon | 1/30 | 98/18805 | 0.14518611 | 0.258016458 | 0.206389878 | TLR2 | 1 |
| BP | GO:0042982 | amyloid precursor protein metabolic process | 1/30 | 99/18805 | 0.146556956 | 0.258972802 | 0.207154868 | EPHA4 | 1 |
| BP | GO:0060996 | dendritic spine development | 1/30 | 99/18805 | 0.146556956 | 0.258972802 | 0.207154868 | EPHA4 | 1 |
| BP | GO:0098869 | cellular oxidant detoxification | 1/30 | 99/18805 | 0.146556956 | 0.258972802 | 0.207154868 | MGST1 | 1 |
| BP | GO:0006721 | terpenoid metabolic process | 1/30 | 100/18805 | 0.147925676 | 0.259426045 | 0.207517421 | PNPLA2 | 1 |
| BP | GO:0021675 | nerve development | 1/30 | 100/18805 | 0.147925676 | 0.259426045 | 0.207517421 | EPHA4 | 1 |
| BP | GO:0044784 | metaphase/anaphase transition of cell cycle | 1/30 | 100/18805 | 0.147925676 | 0.259426045 | 0.207517421 | BCL7A | 1 |
| BP | GO:0051346 | negative regulation of hydrolase activity | 1/30 | 100/18805 | 0.147925676 | 0.259426045 | 0.207517421 | SORT1 | 1 |
| BP | GO:1903035 | negative regulation of response to wounding | 1/30 | 101/18805 | 0.149292275 | 0.261269057 | 0.208991664 | EPHA4 | 1 |
| BP | GO:0032755 | positive regulation of interleukin-6 production | 1/30 | 102/18805 | 0.150656755 | 0.261269057 | 0.208991664 | TLR2 | 1 |
| BP | GO:0048009 | insulin-like growth factor receptor signaling pathway | 1/30 | 102/18805 | 0.150656755 | 0.261269057 | 0.208991664 | EPHA4 | 1 |
| BP | GO:0048534 | hematopoietic or lymphoid organ development | 1/30 | 102/18805 | 0.150656755 | 0.261269057 | 0.208991664 | IL7R | 1 |
| BP | GO:0050764 | regulation of phagocytosis | 1/30 | 102/18805 | 0.150656755 | 0.261269057 | 0.208991664 | TLR2 | 1 |
| BP | GO:0050773 | regulation of dendrite development | 1/30 | 102/18805 | 0.150656755 | 0.261269057 | 0.208991664 | EPHA4 | 1 |
| BP | GO:0010906 | regulation of glucose metabolic process | 1/30 | 103/18805 | 0.152019119 | 0.262169757 | 0.209712142 | RORA | 1 |
| BP | GO:0030101 | natural killer cell activation | 1/30 | 103/18805 | 0.152019119 | 0.262169757 | 0.209712142 | KLRC3 | 1 |
| BP | GO:0034308 | primary alcohol metabolic process | 1/30 | 103/18805 | 0.152019119 | 0.262169757 | 0.209712142 | PNPLA2 | 1 |
| BP | GO:0001960 | negative regulation of cytokine-mediated signaling pathway | 1/30 | 104/18805 | 0.153379371 | 0.263449087 | 0.210735491 | IL1R2 | 1 |
| BP | GO:0042327 | positive regulation of phosphorylation | 2/30 | 437/18805 | 0.153459163 | 0.263449087 | 0.210735491 | S100A12/EPHA4 | 2 |
| BP | GO:0009062 | fatty acid catabolic process | 1/30 | 105/18805 | 0.154737513 | 0.263449087 | 0.210735491 | CRAT | 1 |
| BP | GO:0032602 | chemokine production | 1/30 | 105/18805 | 0.154737513 | 0.263449087 | 0.210735491 | TLR2 | 1 |
| BP | GO:0032637 | interleukin-8 production | 1/30 | 105/18805 | 0.154737513 | 0.263449087 | 0.210735491 | TLR2 | 1 |
| BP | GO:0032642 | regulation of chemokine production | 1/30 | 105/18805 | 0.154737513 | 0.263449087 | 0.210735491 | TLR2 | 1 |
| BP | GO:0032677 | regulation of interleukin-8 production | 1/30 | 105/18805 | 0.154737513 | 0.263449087 | 0.210735491 | TLR2 | 1 |
| BP | GO:0002709 | regulation of T cell mediated immunity | 1/30 | 106/18805 | 0.156093549 | 0.263546989 | 0.210813804 | IL7R | 1 |
| BP | GO:0008585 | female gonad development | 1/30 | 106/18805 | 0.156093549 | 0.263546989 | 0.210813804 | RETN | 1 |
| BP | GO:0048008 | platelet-derived growth factor receptor signaling pathway | 1/30 | 106/18805 | 0.156093549 | 0.263546989 | 0.210813804 | EPHA4 | 1 |
| BP | GO:2000060 | positive regulation of ubiquitin-dependent protein catabolic process | 1/30 | 106/18805 | 0.156093549 | 0.263546989 | 0.210813804 | PYHIN1 | 1 |
| BP | GO:0002532 | production of molecular mediator involved in inflammatory response | 1/30 | 107/18805 | 0.157447482 | 0.263546989 | 0.210813804 | IL1R2 | 1 |
| BP | GO:0019395 | fatty acid oxidation | 1/30 | 107/18805 | 0.157447482 | 0.263546989 | 0.210813804 | CRAT | 1 |
| BP | GO:0043367 | CD4-positive, alpha-beta T cell differentiation | 1/30 | 107/18805 | 0.157447482 | 0.263546989 | 0.210813804 | RORA | 1 |
| BP | GO:1901222 | regulation of non-canonical NF-kappaB signal transduction | 1/30 | 107/18805 | 0.157447482 | 0.263546989 | 0.210813804 | TLR2 | 1 |
| BP | GO:1990266 | neutrophil migration | 1/30 | 107/18805 | 0.157447482 | 0.263546989 | 0.210813804 | S100A12 | 1 |
| BP | GO:0007631 | feeding behavior | 1/30 | 108/18805 | 0.158799315 | 0.263546989 | 0.210813804 | RETN | 1 |
| BP | GO:0021549 | cerebellum development | 1/30 | 108/18805 | 0.158799315 | 0.263546989 | 0.210813804 | RORA | 1 |
| BP | GO:0033045 | regulation of sister chromatid segregation | 1/30 | 108/18805 | 0.158799315 | 0.263546989 | 0.210813804 | BCL7A | 1 |
| BP | GO:0048010 | vascular endothelial growth factor receptor signaling pathway | 1/30 | 108/18805 | 0.158799315 | 0.263546989 | 0.210813804 | EPHA4 | 1 |
| BP | GO:0032102 | negative regulation of response to external stimulus | 2/30 | 448/18805 | 0.15961079 | 0.263546989 | 0.210813804 | EPHA4/RORA | 2 |
| BP | GO:0043405 | regulation of MAP kinase activity | 1/30 | 109/18805 | 0.160149052 | 0.263546989 | 0.210813804 | S100A12 | 1 |
| BP | GO:0048709 | oligodendrocyte differentiation | 1/30 | 109/18805 | 0.160149052 | 0.263546989 | 0.210813804 | TLR2 | 1 |
| BP | GO:0060761 | negative regulation of response to cytokine stimulus | 1/30 | 109/18805 | 0.160149052 | 0.263546989 | 0.210813804 | IL1R2 | 1 |
| BP | GO:0051223 | regulation of protein transport | 2/30 | 449/18805 | 0.160172467 | 0.263546989 | 0.210813804 | TLR2/XPO1 | 2 |
| BP | GO:0006417 | regulation of translation | 2/30 | 451/18805 | 0.161297002 | 0.263546989 | 0.210813804 | EPHA4/EIF5A2 | 2 |
| BP | GO:0006641 | triglyceride metabolic process | 1/30 | 110/18805 | 0.161496694 | 0.263546989 | 0.210813804 | PNPLA2 | 1 |
| BP | GO:0032760 | positive regulation of tumor necrosis factor production | 1/30 | 110/18805 | 0.161496694 | 0.263546989 | 0.210813804 | TLR2 | 1 |
| BP | GO:0046545 | development of primary female sexual characteristics | 1/30 | 110/18805 | 0.161496694 | 0.263546989 | 0.210813804 | RETN | 1 |
| BP | GO:0009410 | response to xenobiotic stimulus | 2/30 | 453/18805 | 0.162423097 | 0.263546989 | 0.210813804 | S100A12/RORA | 2 |
| BP | GO:0001676 | long-chain fatty acid metabolic process | 1/30 | 111/18805 | 0.162842247 | 0.263546989 | 0.210813804 | TBXAS1 | 1 |
| BP | GO:0006892 | post-Golgi vesicle-mediated transport | 1/30 | 111/18805 | 0.162842247 | 0.263546989 | 0.210813804 | SORT1 | 1 |
| BP | GO:0007229 | integrin-mediated signaling pathway | 1/30 | 111/18805 | 0.162842247 | 0.263546989 | 0.210813804 | FUT8 | 1 |
| BP | GO:0042632 | cholesterol homeostasis | 1/30 | 111/18805 | 0.162842247 | 0.263546989 | 0.210813804 | RORA | 1 |
| BP | GO:0060291 | long-term synaptic potentiation | 1/30 | 111/18805 | 0.162842247 | 0.263546989 | 0.210813804 | EPHA4 | 1 |
| BP | GO:0051960 | regulation of nervous system development | 2/30 | 454/18805 | 0.162986723 | 0.263546989 | 0.210813804 | TLR2/EPHA4 | 2 |
| BP | GO:0014013 | regulation of gliogenesis | 1/30 | 112/18805 | 0.164185712 | 0.26365795 | 0.210902563 | TLR2 | 1 |
| BP | GO:0019218 | regulation of steroid metabolic process | 1/30 | 112/18805 | 0.164185712 | 0.26365795 | 0.210902563 | RORA | 1 |
| BP | GO:0055092 | sterol homeostasis | 1/30 | 112/18805 | 0.164185712 | 0.26365795 | 0.210902563 | RORA | 1 |
| BP | GO:1904659 | D-glucose transmembrane transport | 1/30 | 112/18805 | 0.164185712 | 0.26365795 | 0.210902563 | SORT1 | 1 |
| BP | GO:0042752 | regulation of circadian rhythm | 1/30 | 113/18805 | 0.165527092 | 0.264772693 | 0.211794257 | RORA | 1 |
| BP | GO:1905954 | positive regulation of lipid localization | 1/30 | 113/18805 | 0.165527092 | 0.264772693 | 0.211794257 | RETN | 1 |
| BP | GO:0001776 | leukocyte homeostasis | 1/30 | 114/18805 | 0.166866392 | 0.264772693 | 0.211794257 | IL7R | 1 |
| BP | GO:0007218 | neuropeptide signaling pathway | 1/30 | 114/18805 | 0.166866392 | 0.264772693 | 0.211794257 | SORT1 | 1 |
| BP | GO:0034440 | lipid oxidation | 1/30 | 114/18805 | 0.166866392 | 0.264772693 | 0.211794257 | CRAT | 1 |
| BP | GO:0071902 | positive regulation of protein serine/threonine kinase activity | 1/30 | 114/18805 | 0.166866392 | 0.264772693 | 0.211794257 | S100A12 | 1 |
| BP | GO:1903557 | positive regulation of tumor necrosis factor superfamily cytokine production | 1/30 | 114/18805 | 0.166866392 | 0.264772693 | 0.211794257 | TLR2 | 1 |
| BP | GO:0051338 | regulation of transferase activity | 2/30 | 463/18805 | 0.168076261 | 0.265989782 | 0.212767818 | S100A12/EPHA4 | 2 |
| BP | GO:0033559 | unsaturated fatty acid metabolic process | 1/30 | 115/18805 | 0.168203613 | 0.265989782 | 0.212767818 | TBXAS1 | 1 |
| BP | GO:0050731 | positive regulation of peptidyl-tyrosine phosphorylation | 1/30 | 116/18805 | 0.16953876 | 0.267647484 | 0.21409383 | EPHA4 | 1 |
| BP | GO:0022037 | metencephalon development | 1/30 | 118/18805 | 0.172202841 | 0.271394005 | 0.217090708 | RORA | 1 |
| BP | GO:0000045 | autophagosome assembly | 1/30 | 119/18805 | 0.173531782 | 0.271652941 | 0.217297834 | CTSD | 1 |
| BP | GO:0031640 | killing of cells of another organism | 1/30 | 119/18805 | 0.173531782 | 0.271652941 | 0.217297834 | S100A12 | 1 |
| BP | GO:0046822 | regulation of nucleocytoplasmic transport | 1/30 | 119/18805 | 0.173531782 | 0.271652941 | 0.217297834 | XPO1 | 1 |
| BP | GO:0141061 | disruption of cell in another organism | 1/30 | 119/18805 | 0.173531782 | 0.271652941 | 0.217297834 | S100A12 | 1 |
| BP | GO:0016579 | protein deubiquitination | 1/30 | 120/18805 | 0.17485866 | 0.273271574 | 0.218592595 | OTUD3 | 1 |
| BP | GO:0008645 | hexose transmembrane transport | 1/30 | 121/18805 | 0.176183479 | 0.274194244 | 0.219330647 | SORT1 | 1 |
| BP | GO:0034341 | response to type II interferon | 1/30 | 121/18805 | 0.176183479 | 0.274194244 | 0.219330647 | TLR2 | 1 |
| BP | GO:0006720 | isoprenoid metabolic process | 1/30 | 122/18805 | 0.177506242 | 0.274194244 | 0.219330647 | PNPLA2 | 1 |
| BP | GO:0019080 | viral gene expression | 1/30 | 122/18805 | 0.177506242 | 0.274194244 | 0.219330647 | FUT8 | 1 |
| BP | GO:0035924 | cellular response to vascular endothelial growth factor stimulus | 1/30 | 122/18805 | 0.177506242 | 0.274194244 | 0.219330647 | EPHA4 | 1 |
| BP | GO:0045582 | positive regulation of T cell differentiation | 1/30 | 122/18805 | 0.177506242 | 0.274194244 | 0.219330647 | IL7R | 1 |
| BP | GO:0071621 | granulocyte chemotaxis | 1/30 | 122/18805 | 0.177506242 | 0.274194244 | 0.219330647 | S100A12 | 1 |
| BP | GO:0141060 | disruption of anatomical structure in another organism | 1/30 | 123/18805 | 0.178826951 | 0.275457326 | 0.220340999 | S100A12 | 1 |
| BP | GO:0010876 | lipid localization | 2/30 | 482/18805 | 0.178914405 | 0.275457326 | 0.220340999 | PNPLA2/RETN | 2 |
| BP | GO:0015749 | monosaccharide transmembrane transport | 1/30 | 124/18805 | 0.18014561 | 0.275534187 | 0.220402481 | SORT1 | 1 |
| BP | GO:0043500 | muscle adaptation | 1/30 | 124/18805 | 0.18014561 | 0.275534187 | 0.220402481 | TNNT1 | 1 |
| BP | GO:0071887 | leukocyte apoptotic process | 1/30 | 124/18805 | 0.18014561 | 0.275534187 | 0.220402481 | IL7R | 1 |
| BP | GO:0072329 | monocarboxylic acid catabolic process | 1/30 | 124/18805 | 0.18014561 | 0.275534187 | 0.220402481 | CRAT | 1 |
| BP | GO:0046660 | female sex differentiation | 1/30 | 125/18805 | 0.181462222 | 0.276640937 | 0.221287781 | RETN | 1 |
| BP | GO:1990748 | cellular detoxification | 1/30 | 125/18805 | 0.181462222 | 0.276640937 | 0.221287781 | MGST1 | 1 |
| BP | GO:0051656 | establishment of organelle localization | 2/30 | 487/18805 | 0.181786057 | 0.276682531 | 0.221321052 | SDAD1/XPO1 | 2 |
| BP | GO:0010717 | regulation of epithelial to mesenchymal transition | 1/30 | 126/18805 | 0.18277679 | 0.277285765 | 0.221803585 | EPHA4 | 1 |
| BP | GO:1905037 | autophagosome organization | 1/30 | 126/18805 | 0.18277679 | 0.277285765 | 0.221803585 | CTSD | 1 |
| BP | GO:0010562 | positive regulation of phosphorus metabolic process | 2/30 | 493/18805 | 0.185241994 | 0.280114717 | 0.224066491 | S100A12/EPHA4 | 2 |
| BP | GO:0045937 | positive regulation of phosphate metabolic process | 2/30 | 493/18805 | 0.185241994 | 0.280114717 | 0.224066491 | S100A12/EPHA4 | 2 |
| BP | GO:0022613 | ribonucleoprotein complex biogenesis | 2/30 | 494/18805 | 0.185819007 | 0.280532578 | 0.224400742 | SDAD1/XPO1 | 2 |
| BP | GO:0032479 | regulation of type I interferon production | 1/30 | 129/18805 | 0.186708262 | 0.28096582 | 0.224747296 | TLR2 | 1 |
| BP | GO:0032606 | type I interferon production | 1/30 | 129/18805 | 0.186708262 | 0.28096582 | 0.224747296 | TLR2 | 1 |
| BP | GO:0043244 | regulation of protein-containing complex disassembly | 1/30 | 130/18805 | 0.188014684 | 0.281265446 | 0.224986971 | EIF5A2 | 1 |
| BP | GO:0045785 | positive regulation of cell adhesion | 2/30 | 499/18805 | 0.18870832 | 0.281265446 | 0.224986971 | EPHA4/IL7R | 2 |
| BP | GO:1903829 | positive regulation of protein localization | 2/30 | 499/18805 | 0.18870832 | 0.281265446 | 0.224986971 | PYHIN1/TLR2 | 2 |
| BP | GO:0007259 | cell surface receptor signaling pathway via JAK-STAT | 1/30 | 131/18805 | 0.189319079 | 0.281265446 | 0.224986971 | IL7R | 1 |
| BP | GO:0010977 | negative regulation of neuron projection development | 1/30 | 131/18805 | 0.189319079 | 0.281265446 | 0.224986971 | EPHA4 | 1 |
| BP | GO:0032612 | interleukin-1 production | 1/30 | 131/18805 | 0.189319079 | 0.281265446 | 0.224986971 | IL1R2 | 1 |
| BP | GO:0032652 | regulation of interleukin-1 production | 1/30 | 131/18805 | 0.189319079 | 0.281265446 | 0.224986971 | IL1R2 | 1 |
| BP | GO:0045471 | response to ethanol | 1/30 | 131/18805 | 0.189319079 | 0.281265446 | 0.224986971 | TBXAS1 | 1 |
| BP | GO:0014902 | myotube differentiation | 1/30 | 132/18805 | 0.190621447 | 0.282301286 | 0.225815549 | SORT1 | 1 |
| BP | GO:0032434 | regulation of proteasomal ubiquitin-dependent protein catabolic process | 1/30 | 132/18805 | 0.190621447 | 0.282301286 | 0.225815549 | XPO1 | 1 |
| BP | GO:0002708 | positive regulation of lymphocyte mediated immunity | 1/30 | 133/18805 | 0.191921793 | 0.282879988 | 0.226278459 | KLRC3 | 1 |
| BP | GO:0038061 | non-canonical NF-kappaB signal transduction | 1/30 | 133/18805 | 0.191921793 | 0.282879988 | 0.226278459 | TLR2 | 1 |
| BP | GO:0045834 | positive regulation of lipid metabolic process | 1/30 | 133/18805 | 0.191921793 | 0.282879988 | 0.226278459 | PNPLA2 | 1 |
| BP | GO:0002698 | negative regulation of immune effector process | 1/30 | 134/18805 | 0.193220119 | 0.283896647 | 0.227091694 | IL7R | 1 |
| BP | GO:0035710 | CD4-positive, alpha-beta T cell activation | 1/30 | 134/18805 | 0.193220119 | 0.283896647 | 0.227091694 | RORA | 1 |
| BP | GO:0007041 | lysosomal transport | 1/30 | 135/18805 | 0.194516428 | 0.28401225 | 0.227184166 | SORT1 | 1 |
| BP | GO:0008543 | fibroblast growth factor receptor signaling pathway | 1/30 | 135/18805 | 0.194516428 | 0.28401225 | 0.227184166 | EPHA4 | 1 |
| BP | GO:0021782 | glial cell development | 1/30 | 135/18805 | 0.194516428 | 0.28401225 | 0.227184166 | TLR2 | 1 |
| BP | GO:0097237 | cellular response to toxic substance | 1/30 | 135/18805 | 0.194516428 | 0.28401225 | 0.227184166 | MGST1 | 1 |
| BP | GO:0034219 | carbohydrate transmembrane transport | 1/30 | 136/18805 | 0.195810725 | 0.284566053 | 0.227627158 | SORT1 | 1 |
| BP | GO:0051028 | mRNA transport | 1/30 | 136/18805 | 0.195810725 | 0.284566053 | 0.227627158 | XPO1 | 1 |
| BP | GO:0061844 | antimicrobial humoral immune response mediated by antimicrobial peptide | 1/30 | 136/18805 | 0.195810725 | 0.284566053 | 0.227627158 | S100A12 | 1 |
| BP | GO:0006639 | acylglycerol metabolic process | 1/30 | 137/18805 | 0.19710301 | 0.285111796 | 0.228063703 | PNPLA2 | 1 |
| BP | GO:0045621 | positive regulation of lymphocyte differentiation | 1/30 | 137/18805 | 0.19710301 | 0.285111796 | 0.228063703 | IL7R | 1 |
| BP | GO:0051983 | regulation of chromosome segregation | 1/30 | 137/18805 | 0.19710301 | 0.285111796 | 0.228063703 | BCL7A | 1 |
| BP | GO:0006638 | neutral lipid metabolic process | 1/30 | 138/18805 | 0.198393288 | 0.285209457 | 0.228141823 | PNPLA2 | 1 |
| BP | GO:0045739 | positive regulation of DNA repair | 1/30 | 138/18805 | 0.198393288 | 0.285209457 | 0.228141823 | BCL7A | 1 |
| BP | GO:0046632 | alpha-beta T cell differentiation | 1/30 | 138/18805 | 0.198393288 | 0.285209457 | 0.228141823 | RORA | 1 |
| BP | GO:1903052 | positive regulation of proteolysis involved in protein catabolic process | 1/30 | 138/18805 | 0.198393288 | 0.285209457 | 0.228141823 | PYHIN1 | 1 |
| BP | GO:0070646 | protein modification by small protein removal | 1/30 | 139/18805 | 0.199681562 | 0.286619841 | 0.229270003 | OTUD3 | 1 |
| BP | GO:0002286 | T cell activation involved in immune response | 1/30 | 140/18805 | 0.200967834 | 0.287581271 | 0.23003906 | RORA | 1 |
| BP | GO:0045727 | positive regulation of translation | 1/30 | 140/18805 | 0.200967834 | 0.287581271 | 0.23003906 | EIF5A2 | 1 |
| BP | GO:0008643 | carbohydrate transport | 1/30 | 141/18805 | 0.202252107 | 0.288533969 | 0.230801133 | SORT1 | 1 |
| BP | GO:0016482 | cytosolic transport | 1/30 | 141/18805 | 0.202252107 | 0.288533969 | 0.230801133 | SORT1 | 1 |
| BP | GO:0000018 | regulation of DNA recombination | 1/30 | 142/18805 | 0.203534385 | 0.289919971 | 0.23190981 | IL7R | 1 |
| BP | GO:0007098 | centrosome cycle | 1/30 | 143/18805 | 0.204814671 | 0.290855537 | 0.232658177 | XPO1 | 1 |
| BP | GO:0050770 | regulation of axonogenesis | 1/30 | 143/18805 | 0.204814671 | 0.290855537 | 0.232658177 | EPHA4 | 1 |
| BP | GO:0048813 | dendrite morphogenesis | 1/30 | 144/18805 | 0.206092967 | 0.292226045 | 0.233754461 | EPHA4 | 1 |
| BP | GO:2000779 | regulation of double-strand break repair | 1/30 | 145/18805 | 0.207369276 | 0.293589582 | 0.234845167 | BCL7A | 1 |
| BP | GO:0046887 | positive regulation of hormone secretion | 1/30 | 146/18805 | 0.208643602 | 0.294499971 | 0.235573396 | RETN | 1 |
| BP | GO:1900180 | regulation of protein localization to nucleus | 1/30 | 146/18805 | 0.208643602 | 0.294499971 | 0.235573396 | PYHIN1 | 1 |
| BP | GO:0051961 | negative regulation of nervous system development | 1/30 | 147/18805 | 0.209915947 | 0.295848306 | 0.236651942 | TLR2 | 1 |
| BP | GO:0007224 | smoothened signaling pathway | 1/30 | 148/18805 | 0.211186315 | 0.296742217 | 0.23736699 | RORA | 1 |
| BP | GO:0099175 | regulation of postsynapse organization | 1/30 | 148/18805 | 0.211186315 | 0.296742217 | 0.23736699 | EPHA4 | 1 |
| BP | GO:0097530 | granulocyte migration | 1/30 | 149/18805 | 0.212454708 | 0.298075553 | 0.238433539 | S100A12 | 1 |
| BP | GO:0050714 | positive regulation of protein secretion | 1/30 | 150/18805 | 0.21372113 | 0.299402123 | 0.239494675 | TLR2 | 1 |
| BP | GO:0050852 | T cell receptor signaling pathway | 1/30 | 151/18805 | 0.214985582 | 0.300721962 | 0.240550427 | CD8A | 1 |
| BP | GO:0030177 | positive regulation of Wnt signaling pathway | 1/30 | 152/18805 | 0.216248069 | 0.30158363 | 0.241239684 | TLR2 | 1 |
| BP | GO:0033157 | regulation of intracellular protein transport | 1/30 | 152/18805 | 0.216248069 | 0.30158363 | 0.241239684 | XPO1 | 1 |
| BP | GO:0002705 | positive regulation of leukocyte mediated immunity | 1/30 | 155/18805 | 0.220023765 | 0.305855948 | 0.244657152 | KLRC3 | 1 |
| BP | GO:0072073 | kidney epithelium development | 1/30 | 155/18805 | 0.220023765 | 0.305855948 | 0.244657152 | EPHA4 | 1 |
| BP | GO:0031023 | microtubule organizing center organization | 1/30 | 156/18805 | 0.221278419 | 0.305855948 | 0.244657152 | XPO1 | 1 |
| BP | GO:0035296 | regulation of tube diameter | 1/30 | 156/18805 | 0.221278419 | 0.305855948 | 0.244657152 | TBXAS1 | 1 |
| BP | GO:0050730 | regulation of peptidyl-tyrosine phosphorylation | 1/30 | 156/18805 | 0.221278419 | 0.305855948 | 0.244657152 | EPHA4 | 1 |
| BP | GO:0097746 | blood vessel diameter maintenance | 1/30 | 156/18805 | 0.221278419 | 0.305855948 | 0.244657152 | TBXAS1 | 1 |
| BP | GO:0032368 | regulation of lipid transport | 1/30 | 157/18805 | 0.222531121 | 0.306678783 | 0.245315346 | RETN | 1 |
| BP | GO:0035150 | regulation of tube size | 1/30 | 157/18805 | 0.222531121 | 0.306678783 | 0.245315346 | TBXAS1 | 1 |
| BP | GO:0007173 | epidermal growth factor receptor signaling pathway | 1/30 | 158/18805 | 0.223781876 | 0.307041897 | 0.245605805 | EPHA4 | 1 |
| BP | GO:0008037 | cell recognition | 1/30 | 158/18805 | 0.223781876 | 0.307041897 | 0.245605805 | EPHA4 | 1 |
| BP | GO:0042552 | myelination | 1/30 | 158/18805 | 0.223781876 | 0.307041897 | 0.245605805 | TLR2 | 1 |
| BP | GO:0097696 | cell surface receptor signaling pathway via STAT | 1/30 | 159/18805 | 0.225030685 | 0.308199935 | 0.246532131 | IL7R | 1 |
| BP | GO:0007272 | ensheathment of neurons | 1/30 | 160/18805 | 0.226277552 | 0.308199935 | 0.246532131 | TLR2 | 1 |
| BP | GO:0007612 | learning | 1/30 | 160/18805 | 0.226277552 | 0.308199935 | 0.246532131 | TLR2 | 1 |
| BP | GO:0008366 | axon ensheathment | 1/30 | 160/18805 | 0.226277552 | 0.308199935 | 0.246532131 | TLR2 | 1 |
| BP | GO:0061351 | neural precursor cell proliferation | 1/30 | 160/18805 | 0.226277552 | 0.308199935 | 0.246532131 | RORA | 1 |
| BP | GO:0030902 | hindbrain development | 1/30 | 161/18805 | 0.227522479 | 0.309443838 | 0.247527141 | RORA | 1 |
| BP | GO:0044344 | cellular response to fibroblast growth factor stimulus | 1/30 | 165/18805 | 0.232482854 | 0.314813502 | 0.251822387 | EPHA4 | 1 |
| BP | GO:0050657 | nucleic acid transport | 1/30 | 165/18805 | 0.232482854 | 0.314813502 | 0.251822387 | XPO1 | 1 |
| BP | GO:0050658 | RNA transport | 1/30 | 165/18805 | 0.232482854 | 0.314813502 | 0.251822387 | XPO1 | 1 |
| BP | GO:0051236 | establishment of RNA localization | 1/30 | 168/18805 | 0.236182914 | 0.318898204 | 0.25508978 | XPO1 | 1 |
| BP | GO:0098754 | detoxification | 1/30 | 168/18805 | 0.236182914 | 0.318898204 | 0.25508978 | MGST1 | 1 |
| BP | GO:0051963 | regulation of synapse assembly | 1/30 | 169/18805 | 0.237412431 | 0.320095084 | 0.256047176 | TLR2 | 1 |
| BP | GO:0006633 | fatty acid biosynthetic process | 1/30 | 170/18805 | 0.238640035 | 0.321285935 | 0.256999749 | TBXAS1 | 1 |
| BP | GO:0043409 | negative regulation of MAPK cascade | 1/30 | 171/18805 | 0.239865729 | 0.322006798 | 0.257576375 | EPHA4 | 1 |
| BP | GO:0043524 | negative regulation of neuron apoptotic process | 1/30 | 171/18805 | 0.239865729 | 0.322006798 | 0.257576375 | EPHA4 | 1 |
| BP | GO:0007034 | vacuolar transport | 1/30 | 172/18805 | 0.241089515 | 0.322720972 | 0.258147649 | SORT1 | 1 |
| BP | GO:0043087 | regulation of GTPase activity | 1/30 | 172/18805 | 0.241089515 | 0.322720972 | 0.258147649 | EPHA4 | 1 |
| BP | GO:0046777 | protein autophosphorylation | 1/30 | 173/18805 | 0.242311396 | 0.323428516 | 0.258713621 | EPHA4 | 1 |
| BP | GO:0071774 | response to fibroblast growth factor | 1/30 | 173/18805 | 0.242311396 | 0.323428516 | 0.258713621 | EPHA4 | 1 |
| BP | GO:0030856 | regulation of epithelial cell differentiation | 1/30 | 174/18805 | 0.243531376 | 0.323667768 | 0.258905001 | MAFG | 1 |
| BP | GO:0048284 | organelle fusion | 1/30 | 174/18805 | 0.243531376 | 0.323667768 | 0.258905001 | PNPLA2 | 1 |
| BP | GO:0055002 | striated muscle cell development | 1/30 | 174/18805 | 0.243531376 | 0.323667768 | 0.258905001 | TNNT1 | 1 |
| BP | GO:0006937 | regulation of muscle contraction | 1/30 | 175/18805 | 0.244749456 | 0.324823958 | 0.259829849 | TNNT1 | 1 |
| BP | GO:0050905 | neuromuscular process | 1/30 | 176/18805 | 0.245965641 | 0.325511975 | 0.2603802 | TNNT1 | 1 |
| BP | GO:2000045 | regulation of G1/S transition of mitotic cell cycle | 1/30 | 176/18805 | 0.245965641 | 0.325511975 | 0.2603802 | BCL7A | 1 |
| BP | GO:0032635 | interleukin-6 production | 1/30 | 177/18805 | 0.247179932 | 0.326193602 | 0.26092544 | TLR2 | 1 |
| BP | GO:0032675 | regulation of interleukin-6 production | 1/30 | 177/18805 | 0.247179932 | 0.326193602 | 0.26092544 | TLR2 | 1 |
| BP | GO:0008361 | regulation of cell size | 1/30 | 179/18805 | 0.249602846 | 0.328461855 | 0.262739838 | IL7R | 1 |
| BP | GO:0016052 | carbohydrate catabolic process | 1/30 | 179/18805 | 0.249602846 | 0.328461855 | 0.262739838 | FUT8 | 1 |
| BP | GO:0038127 | ERBB signaling pathway | 1/30 | 180/18805 | 0.250811474 | 0.329587473 | 0.26364023 | EPHA4 | 1 |
| BP | GO:1903034 | regulation of response to wounding | 1/30 | 181/18805 | 0.252018221 | 0.330707454 | 0.264536115 | EPHA4 | 1 |
| BP | GO:0006109 | regulation of carbohydrate metabolic process | 1/30 | 184/18805 | 0.255627198 | 0.33450235 | 0.267571688 | RORA | 1 |
| BP | GO:0006575 | modified amino acid metabolic process | 1/30 | 184/18805 | 0.255627198 | 0.33450235 | 0.267571688 | CRAT | 1 |
| BP | GO:0019730 | antimicrobial humoral response | 1/30 | 185/18805 | 0.256826445 | 0.335600943 | 0.268450464 | S100A12 | 1 |
| BP | GO:0019827 | stem cell population maintenance | 1/30 | 187/18805 | 0.259219339 | 0.338254047 | 0.270572707 | BCL7A | 1 |
| BP | GO:0031345 | negative regulation of cell projection organization | 1/30 | 188/18805 | 0.260412991 | 0.338863767 | 0.271060429 | EPHA4 | 1 |
| BP | GO:0051345 | positive regulation of hydrolase activity | 1/30 | 188/18805 | 0.260412991 | 0.338863767 | 0.271060429 | EPHA4 | 1 |
| BP | GO:0001959 | regulation of cytokine-mediated signaling pathway | 1/30 | 189/18805 | 0.261604784 | 0.339940479 | 0.271921701 | IL1R2 | 1 |
| BP | GO:0032640 | tumor necrosis factor production | 1/30 | 191/18805 | 0.263982803 | 0.341603267 | 0.273251781 | TLR2 | 1 |
| BP | GO:0032680 | regulation of tumor necrosis factor production | 1/30 | 191/18805 | 0.263982803 | 0.341603267 | 0.273251781 | TLR2 | 1 |
| BP | GO:0098727 | maintenance of cell number | 1/30 | 191/18805 | 0.263982803 | 0.341603267 | 0.273251781 | BCL7A | 1 |
| BP | GO:0001837 | epithelial to mesenchymal transition | 1/30 | 192/18805 | 0.265169035 | 0.342189087 | 0.273720384 | EPHA4 | 1 |
| BP | GO:0045580 | regulation of T cell differentiation | 1/30 | 192/18805 | 0.265169035 | 0.342189087 | 0.273720384 | IL7R | 1 |
| BP | GO:0006006 | glucose metabolic process | 1/30 | 193/18805 | 0.266353419 | 0.343242734 | 0.274563207 | RORA | 1 |
| BP | GO:0006941 | striated muscle contraction | 1/30 | 194/18805 | 0.267535957 | 0.34381687 | 0.275022464 | TNNT1 | 1 |
| BP | GO:0030258 | lipid modification | 1/30 | 194/18805 | 0.267535957 | 0.34381687 | 0.275022464 | CRAT | 1 |
| BP | GO:1902107 | positive regulation of leukocyte differentiation | 1/30 | 195/18805 | 0.268716652 | 0.344385489 | 0.275477308 | IL7R | 1 |
| BP | GO:1903708 | positive regulation of hemopoiesis | 1/30 | 195/18805 | 0.268716652 | 0.344385489 | 0.275477308 | IL7R | 1 |
| BP | GO:0071706 | tumor necrosis factor superfamily cytokine production | 1/30 | 196/18805 | 0.269895507 | 0.344948642 | 0.275927779 | TLR2 | 1 |
| BP | GO:1903555 | regulation of tumor necrosis factor superfamily cytokine production | 1/30 | 196/18805 | 0.269895507 | 0.344948642 | 0.275927779 | TLR2 | 1 |
| BP | GO:0018108 | peptidyl-tyrosine phosphorylation | 1/30 | 198/18805 | 0.27224771 | 0.347478951 | 0.277951798 | EPHA4 | 1 |
| BP | GO:0018212 | peptidyl-tyrosine modification | 1/30 | 199/18805 | 0.273421062 | 0.348499797 | 0.278768382 | EPHA4 | 1 |
| BP | GO:0061136 | regulation of proteasomal protein catabolic process | 1/30 | 200/18805 | 0.274592586 | 0.349515529 | 0.279580877 | XPO1 | 1 |
| BP | GO:0046631 | alpha-beta T cell activation | 1/30 | 201/18805 | 0.275762283 | 0.350526172 | 0.2803893 | RORA | 1 |
| BP | GO:0002520 | immune system development | 1/30 | 202/18805 | 0.276930158 | 0.351054126 | 0.280811616 | IL7R | 1 |
| BP | GO:0055001 | muscle cell development | 1/30 | 202/18805 | 0.276930158 | 0.351054126 | 0.280811616 | TNNT1 | 1 |
| BP | GO:0031099 | regeneration | 1/30 | 203/18805 | 0.278096211 | 0.352053955 | 0.281611389 | EPHA4 | 1 |
| BP | GO:0002822 | regulation of adaptive immune response based on somatic recombination of immune receptors built from immunoglobulin superfamily domains | 1/30 | 204/18805 | 0.279260447 | 0.352607191 | 0.282053927 | IL7R | 1 |
| BP | GO:0016064 | immunoglobulin mediated immune response | 1/30 | 205/18805 | 0.280422868 | 0.352607191 | 0.282053927 | C1RL | 1 |
| BP | GO:0048839 | inner ear development | 1/30 | 205/18805 | 0.280422868 | 0.352607191 | 0.282053927 | EPHA4 | 1 |
| BP | GO:1902806 | regulation of cell cycle G1/S phase transition | 1/30 | 205/18805 | 0.280422868 | 0.352607191 | 0.282053927 | BCL7A | 1 |
| BP | GO:2000241 | regulation of reproductive process | 1/30 | 205/18805 | 0.280422868 | 0.352607191 | 0.282053927 | RETN | 1 |
| BP | GO:0006403 | RNA localization | 1/30 | 206/18805 | 0.281583476 | 0.353590017 | 0.2828401 | XPO1 | 1 |
| BP | GO:0007623 | circadian rhythm | 1/30 | 207/18805 | 0.282742274 | 0.353617349 | 0.282861962 | RORA | 1 |
| BP | GO:0016051 | carbohydrate biosynthetic process | 1/30 | 207/18805 | 0.282742274 | 0.353617349 | 0.282861962 | FUT8 | 1 |
| BP | GO:0050728 | negative regulation of inflammatory response | 1/30 | 207/18805 | 0.282742274 | 0.353617349 | 0.282861962 | RORA | 1 |
| BP | GO:0019724 | B cell mediated immunity | 1/30 | 208/18805 | 0.283899266 | 0.354589043 | 0.28363923 | C1RL | 1 |
| BP | GO:0017148 | negative regulation of translation | 1/30 | 213/18805 | 0.289657216 | 0.360814663 | 0.288619164 | EPHA4 | 1 |
| BP | GO:0050851 | antigen receptor-mediated signaling pathway | 1/30 | 213/18805 | 0.289657216 | 0.360814663 | 0.288619164 | CD8A | 1 |
| BP | GO:0043123 | positive regulation of canonical NF-kappaB signal transduction | 1/30 | 214/18805 | 0.290803423 | 0.361277755 | 0.288989596 | S100A12 | 1 |
| BP | GO:0050777 | negative regulation of immune response | 1/30 | 214/18805 | 0.290803423 | 0.361277755 | 0.288989596 | IL7R | 1 |
| BP | GO:0007626 | locomotory behavior | 1/30 | 215/18805 | 0.291947842 | 0.361736171 | 0.289356287 | EPHA4 | 1 |
| BP | GO:0031032 | actomyosin structure organization | 1/30 | 215/18805 | 0.291947842 | 0.361736171 | 0.289356287 | TNNT1 | 1 |
| BP | GO:0071900 | regulation of protein serine/threonine kinase activity | 1/30 | 217/18805 | 0.294231328 | 0.364082001 | 0.291232739 | S100A12 | 1 |
| BP | GO:0002819 | regulation of adaptive immune response | 1/30 | 219/18805 | 0.296507693 | 0.365928145 | 0.292709488 | IL7R | 1 |
| BP | GO:0071356 | cellular response to tumor necrosis factor | 1/30 | 219/18805 | 0.296507693 | 0.365928145 | 0.292709488 | RORA | 1 |
| BP | GO:0006486 | protein glycosylation | 1/30 | 221/18805 | 0.298776961 | 0.367755811 | 0.294171456 | FUT8 | 1 |
| BP | GO:0043413 | macromolecule glycosylation | 1/30 | 221/18805 | 0.298776961 | 0.367755811 | 0.294171456 | FUT8 | 1 |
| BP | GO:0045216 | cell-cell junction organization | 1/30 | 222/18805 | 0.299908939 | 0.368662767 | 0.294896939 | EPHA4 | 1 |
| BP | GO:0009612 | response to mechanical stimulus | 1/30 | 223/18805 | 0.301039152 | 0.369079538 | 0.295230318 | RETN | 1 |
| BP | GO:0048167 | regulation of synaptic plasticity | 1/30 | 223/18805 | 0.301039152 | 0.369079538 | 0.295230318 | EPHA4 | 1 |
| BP | GO:0045619 | regulation of lymphocyte differentiation | 1/30 | 225/18805 | 0.303294287 | 0.370869685 | 0.296662274 | IL7R | 1 |
| BP | GO:1901654 | response to ketone | 1/30 | 225/18805 | 0.303294287 | 0.370869685 | 0.296662274 | TLR2 | 1 |
| BP | GO:0006282 | regulation of DNA repair | 1/30 | 227/18805 | 0.305542388 | 0.373129644 | 0.298470037 | BCL7A | 1 |
| BP | GO:0072330 | monocarboxylic acid biosynthetic process | 1/30 | 229/18805 | 0.307783476 | 0.375375141 | 0.300266233 | TBXAS1 | 1 |
| BP | GO:0071674 | mononuclear cell migration | 1/30 | 230/18805 | 0.308901397 | 0.37624674 | 0.300963433 | S100A12 | 1 |
| BP | GO:0002285 | lymphocyte activation involved in immune response | 1/30 | 231/18805 | 0.310017572 | 0.377113944 | 0.301657118 | RORA | 1 |
| BP | GO:0043583 | ear development | 1/30 | 232/18805 | 0.311132005 | 0.377485254 | 0.301954132 | EPHA4 | 1 |
| BP | GO:0071222 | cellular response to lipopolysaccharide | 1/30 | 232/18805 | 0.311132005 | 0.377485254 | 0.301954132 | TLR2 | 1 |
| BP | GO:0000819 | sister chromatid segregation | 1/30 | 234/18805 | 0.313355653 | 0.379196918 | 0.303323309 | BCL7A | 1 |
| BP | GO:0030595 | leukocyte chemotaxis | 1/30 | 234/18805 | 0.313355653 | 0.379196918 | 0.303323309 | S100A12 | 1 |
| BP | GO:0097191 | extrinsic apoptotic signaling pathway | 1/30 | 237/18805 | 0.31667812 | 0.381950353 | 0.305525809 | SORT1 | 1 |
| BP | GO:0097529 | myeloid leukocyte migration | 1/30 | 237/18805 | 0.31667812 | 0.381950353 | 0.305525809 | S100A12 | 1 |
| BP | GO:0006909 | phagocytosis | 1/30 | 238/18805 | 0.317782151 | 0.381950353 | 0.305525809 | TLR2 | 1 |
| BP | GO:0007033 | vacuole organization | 1/30 | 238/18805 | 0.317782151 | 0.381950353 | 0.305525809 | CTSD | 1 |
| BP | GO:0007411 | axon guidance | 1/30 | 238/18805 | 0.317782151 | 0.381950353 | 0.305525809 | EPHA4 | 1 |
| BP | GO:0097485 | neuron projection guidance | 1/30 | 239/18805 | 0.318884458 | 0.381950353 | 0.305525809 | EPHA4 | 1 |
| BP | GO:0000082 | G1/S transition of mitotic cell cycle | 1/30 | 240/18805 | 0.319985043 | 0.381950353 | 0.305525809 | BCL7A | 1 |
| BP | GO:0008406 | gonad development | 1/30 | 240/18805 | 0.319985043 | 0.381950353 | 0.305525809 | RETN | 1 |
| BP | GO:0015931 | nucleobase-containing compound transport | 1/30 | 240/18805 | 0.319985043 | 0.381950353 | 0.305525809 | XPO1 | 1 |
| BP | GO:0042445 | hormone metabolic process | 1/30 | 240/18805 | 0.319985043 | 0.381950353 | 0.305525809 | PNPLA2 | 1 |
| BP | GO:0034612 | response to tumor necrosis factor | 1/30 | 241/18805 | 0.321083909 | 0.381950353 | 0.305525809 | RORA | 1 |
| BP | GO:0070085 | glycosylation | 1/30 | 241/18805 | 0.321083909 | 0.381950353 | 0.305525809 | FUT8 | 1 |
| BP | GO:0016054 | organic acid catabolic process | 1/30 | 242/18805 | 0.322181059 | 0.381950353 | 0.305525809 | CRAT | 1 |
| BP | GO:0016358 | dendrite development | 1/30 | 242/18805 | 0.322181059 | 0.381950353 | 0.305525809 | EPHA4 | 1 |
| BP | GO:0046395 | carboxylic acid catabolic process | 1/30 | 242/18805 | 0.322181059 | 0.381950353 | 0.305525809 | CRAT | 1 |
| BP | GO:0046434 | organophosphate catabolic process | 1/30 | 242/18805 | 0.322181059 | 0.381950353 | 0.305525809 | PLBD1 | 1 |
| BP | GO:0001667 | ameboidal-type cell migration | 1/30 | 243/18805 | 0.323276494 | 0.382277528 | 0.305787518 | FUT8 | 1 |
| BP | GO:0099173 | postsynapse organization | 1/30 | 243/18805 | 0.323276494 | 0.382277528 | 0.305787518 | EPHA4 | 1 |
| BP | GO:0033044 | regulation of chromosome organization | 1/30 | 244/18805 | 0.324370218 | 0.383085334 | 0.306433691 | BCL7A | 1 |
| BP | GO:0045137 | development of primary sexual characteristics | 1/30 | 245/18805 | 0.325462234 | 0.383889082 | 0.307076617 | RETN | 1 |
| BP | GO:0045017 | glycerolipid biosynthetic process | 1/30 | 246/18805 | 0.326552542 | 0.384203685 | 0.30732827 | PNPLA2 | 1 |
| BP | GO:0071219 | cellular response to molecule of bacterial origin | 1/30 | 246/18805 | 0.326552542 | 0.384203685 | 0.30732827 | TLR2 | 1 |
| BP | GO:0022604 | regulation of cell morphogenesis | 1/30 | 247/18805 | 0.327641148 | 0.384998981 | 0.307964435 | EPHA4 | 1 |
| BP | GO:0002573 | myeloid leukocyte differentiation | 1/30 | 250/18805 | 0.330896767 | 0.388335451 | 0.31063331 | TLR2 | 1 |
| BP | GO:0009913 | epidermal cell differentiation | 1/30 | 251/18805 | 0.331978583 | 0.3891156 | 0.311257359 | MAFG | 1 |
| BP | GO:0007179 | transforming growth factor beta receptor signaling pathway | 1/30 | 252/18805 | 0.333058708 | 0.389891812 | 0.311878259 | FUT8 | 1 |
| BP | GO:0043523 | regulation of neuron apoptotic process | 1/30 | 255/18805 | 0.336288964 | 0.393179954 | 0.314508475 | EPHA4 | 1 |
| BP | GO:0090257 | regulation of muscle system process | 1/30 | 256/18805 | 0.337362351 | 0.393941268 | 0.315117458 | TNNT1 | 1 |
| BP | GO:0051222 | positive regulation of protein transport | 1/30 | 257/18805 | 0.33843406 | 0.394698722 | 0.315723353 | TLR2 | 1 |
| BP | GO:0031669 | cellular response to nutrient levels | 1/30 | 258/18805 | 0.339504093 | 0.395452333 | 0.316326174 | OTUD3 | 1 |
| BP | GO:0032984 | protein-containing complex disassembly | 1/30 | 261/18805 | 0.342704165 | 0.398185536 | 0.31851249 | EIF5A2 | 1 |
| BP | GO:0050870 | positive regulation of T cell activation | 1/30 | 261/18805 | 0.342704165 | 0.398185536 | 0.31851249 | IL7R | 1 |
| BP | GO:0046883 | regulation of hormone secretion | 1/30 | 262/18805 | 0.343767521 | 0.39892425 | 0.319103394 | RETN | 1 |
| BP | GO:0032886 | regulation of microtubule-based process | 1/30 | 269/18805 | 0.351164589 | 0.406496975 | 0.32516089 | XPO1 | 1 |
| BP | GO:1903522 | regulation of blood circulation | 1/30 | 269/18805 | 0.351164589 | 0.406496975 | 0.32516089 | TBXAS1 | 1 |
| BP | GO:0002699 | positive regulation of immune effector process | 1/30 | 270/18805 | 0.352214711 | 0.406703373 | 0.32532599 | KLRC3 | 1 |
| BP | GO:1901888 | regulation of cell junction assembly | 1/30 | 270/18805 | 0.352214711 | 0.406703373 | 0.32532599 | TLR2 | 1 |
| BP | GO:0044843 | cell cycle G1/S phase transition | 1/30 | 271/18805 | 0.35326319 | 0.406805434 | 0.32540763 | BCL7A | 1 |
| BP | GO:0001894 | tissue homeostasis | 1/30 | 272/18805 | 0.354310029 | 0.406805434 | 0.32540763 | IQCB1 | 1 |
| BP | GO:0060249 | anatomical structure homeostasis | 1/30 | 272/18805 | 0.354310029 | 0.406805434 | 0.32540763 | IQCB1 | 1 |
| BP | GO:0007416 | synapse assembly | 1/30 | 273/18805 | 0.355355229 | 0.406805434 | 0.32540763 | TLR2 | 1 |
| BP | GO:0032386 | regulation of intracellular transport | 1/30 | 273/18805 | 0.355355229 | 0.406805434 | 0.32540763 | XPO1 | 1 |
| BP | GO:0050708 | regulation of protein secretion | 1/30 | 273/18805 | 0.355355229 | 0.406805434 | 0.32540763 | TLR2 | 1 |
| BP | GO:0071216 | cellular response to biotic stimulus | 1/30 | 273/18805 | 0.355355229 | 0.406805434 | 0.32540763 | TLR2 | 1 |
| BP | GO:0048762 | mesenchymal cell differentiation | 1/30 | 275/18805 | 0.357440725 | 0.408691418 | 0.326916247 | EPHA4 | 1 |
| BP | GO:0097305 | response to alcohol | 1/30 | 276/18805 | 0.358481027 | 0.409379189 | 0.327466402 | TBXAS1 | 1 |
| BP | GO:0003018 | vascular process in circulatory system | 1/30 | 278/18805 | 0.360556746 | 0.410744132 | 0.328558233 | TBXAS1 | 1 |
| BP | GO:0051896 | regulation of phosphatidylinositol 3-kinase/protein kinase B signal transduction | 1/30 | 278/18805 | 0.360556746 | 0.410744132 | 0.328558233 | OTUD3 | 1 |
| BP | GO:0007611 | learning or memory | 1/30 | 282/18805 | 0.364688727 | 0.414944612 | 0.331918238 | TLR2 | 1 |
| BP | GO:0015833 | peptide transport | 1/30 | 283/18805 | 0.365717682 | 0.415608523 | 0.332449306 | MGST1 | 1 |
| BP | GO:1903039 | positive regulation of leukocyte cell-cell adhesion | 1/30 | 285/18805 | 0.367770762 | 0.417433237 | 0.333908913 | IL7R | 1 |
| BP | GO:0042113 | B cell activation | 1/30 | 287/18805 | 0.369817417 | 0.419246233 | 0.335359146 | IL7R | 1 |
| BP | GO:0002221 | pattern recognition receptor signaling pathway | 1/30 | 288/18805 | 0.370838341 | 0.419893413 | 0.335876832 | TLR2 | 1 |
| BP | GO:0016197 | endosomal transport | 1/30 | 293/18805 | 0.375919028 | 0.42410212 | 0.339243417 | SORT1 | 1 |
| BP | GO:0043542 | endothelial cell migration | 1/30 | 293/18805 | 0.375919028 | 0.42410212 | 0.339243417 | S100A12 | 1 |
| BP | GO:0051054 | positive regulation of DNA metabolic process | 1/30 | 293/18805 | 0.375919028 | 0.42410212 | 0.339243417 | BCL7A | 1 |
| BP | GO:0007548 | sex differentiation | 1/30 | 296/18805 | 0.378948379 | 0.426488344 | 0.341152181 | RETN | 1 |
| BP | GO:0070372 | regulation of ERK1 and ERK2 cascade | 1/30 | 296/18805 | 0.378948379 | 0.426488344 | 0.341152181 | EPHA4 | 1 |
| BP | GO:0046651 | lymphocyte proliferation | 1/30 | 300/18805 | 0.382965409 | 0.430490032 | 0.34435317 | IL7R | 1 |
| BP | GO:0043086 | negative regulation of catalytic activity | 1/30 | 306/18805 | 0.388943898 | 0.436159443 | 0.348888188 | SORT1 | 1 |
| BP | GO:0048193 | Golgi vesicle transport | 1/30 | 306/18805 | 0.388943898 | 0.436159443 | 0.348888188 | SORT1 | 1 |
| BP | GO:0031348 | negative regulation of defense response | 1/30 | 308/18805 | 0.390924255 | 0.436805185 | 0.349404723 | RORA | 1 |
| BP | GO:0032943 | mononuclear cell proliferation | 1/30 | 308/18805 | 0.390924255 | 0.436805185 | 0.349404723 | IL7R | 1 |
| BP | GO:0071560 | cellular response to transforming growth factor beta stimulus | 1/30 | 308/18805 | 0.390924255 | 0.436805185 | 0.349404723 | FUT8 | 1 |
| BP | GO:0030100 | regulation of endocytosis | 1/30 | 309/18805 | 0.391912105 | 0.436862598 | 0.349450648 | TLR2 | 1 |
| BP | GO:0051402 | neuron apoptotic process | 1/30 | 309/18805 | 0.391912105 | 0.436862598 | 0.349450648 | EPHA4 | 1 |
| BP | GO:0007162 | negative regulation of cell adhesion | 1/30 | 312/18805 | 0.394866373 | 0.439630461 | 0.351664689 | EPHA4 | 1 |
| BP | GO:0048608 | reproductive structure development | 1/30 | 314/18805 | 0.396828172 | 0.441288063 | 0.352990621 | RETN | 1 |
| BP | GO:0071559 | response to transforming growth factor beta | 1/30 | 315/18805 | 0.397806765 | 0.441849657 | 0.353439845 | FUT8 | 1 |
| BP | GO:0043491 | phosphatidylinositol 3-kinase/protein kinase B signal transduction | 1/30 | 316/18805 | 0.398783823 | 0.442408212 | 0.353886639 | OTUD3 | 1 |
| BP | GO:0061458 | reproductive system development | 1/30 | 318/18805 | 0.400733343 | 0.444043003 | 0.355194324 | RETN | 1 |
| BP | GO:0002366 | leukocyte activation involved in immune response | 1/30 | 319/18805 | 0.40170581 | 0.444065783 | 0.355212546 | RORA | 1 |
| BP | GO:0070371 | ERK1 and ERK2 cascade | 1/30 | 319/18805 | 0.40170581 | 0.444065783 | 0.355212546 | EPHA4 | 1 |
| BP | GO:0046879 | hormone secretion | 1/30 | 320/18805 | 0.402676751 | 0.444612318 | 0.355649725 | RETN | 1 |
| BP | GO:0051607 | defense response to virus | 1/30 | 321/18805 | 0.40364617 | 0.445155882 | 0.356084527 | TLR2 | 1 |
| BP | GO:0098813 | nuclear chromosome segregation | 1/30 | 322/18805 | 0.404614067 | 0.445184183 | 0.356107165 | BCL7A | 1 |
| BP | GO:0002263 | cell activation involved in immune response | 1/30 | 323/18805 | 0.405580446 | 0.445184183 | 0.356107165 | RORA | 1 |
| BP | GO:0006302 | double-strand break repair | 1/30 | 323/18805 | 0.405580446 | 0.445184183 | 0.356107165 | BCL7A | 1 |
| BP | GO:0044248 | cellular catabolic process | 1/30 | 323/18805 | 0.405580446 | 0.445184183 | 0.356107165 | CRAT | 1 |
| BP | GO:0001822 | kidney development | 1/30 | 325/18805 | 0.407508657 | 0.446250677 | 0.356960264 | EPHA4 | 1 |
| BP | GO:0060326 | cell chemotaxis | 1/30 | 325/18805 | 0.407508657 | 0.446250677 | 0.356960264 | S100A12 | 1 |
| BP | GO:0009101 | glycoprotein biosynthetic process | 1/30 | 326/18805 | 0.408470493 | 0.446779566 | 0.357383328 | FUT8 | 1 |
| BP | GO:0042886 | amide transport | 1/30 | 328/18805 | 0.41038964 | 0.448353085 | 0.358642 | MGST1 | 1 |
| BP | GO:0050890 | cognition | 1/30 | 329/18805 | 0.411346955 | 0.448873344 | 0.359058161 | TLR2 | 1 |
| BP | GO:0009914 | hormone transport | 1/30 | 331/18805 | 0.41325708 | 0.449905316 | 0.359883645 | RETN | 1 |
| BP | GO:1904951 | positive regulation of establishment of protein localization | 1/30 | 331/18805 | 0.41325708 | 0.449905316 | 0.359883645 | TLR2 | 1 |
| BP | GO:0046394 | carboxylic acid biosynthetic process | 1/30 | 334/18805 | 0.416111037 | 0.452409511 | 0.361886775 | TBXAS1 | 1 |
| BP | GO:0008202 | steroid metabolic process | 1/30 | 335/18805 | 0.417059371 | 0.452409511 | 0.361886775 | RORA | 1 |
| BP | GO:0072001 | renal system development | 1/30 | 336/18805 | 0.418006215 | 0.452409511 | 0.361886775 | EPHA4 | 1 |
| BP | GO:0016053 | organic acid biosynthetic process | 1/30 | 337/18805 | 0.418951573 | 0.452409511 | 0.361886775 | TBXAS1 | 1 |
| BP | GO:0022409 | positive regulation of cell-cell adhesion | 1/30 | 337/18805 | 0.418951573 | 0.452409511 | 0.361886775 | IL7R | 1 |
| BP | GO:0051251 | positive regulation of lymphocyte activation | 1/30 | 337/18805 | 0.418951573 | 0.452409511 | 0.361886775 | IL7R | 1 |
| BP | GO:0060562 | epithelial tube morphogenesis | 1/30 | 337/18805 | 0.418951573 | 0.452409511 | 0.361886775 | EPHA4 | 1 |
| BP | GO:0048545 | response to steroid hormone | 1/30 | 338/18805 | 0.419895446 | 0.452873936 | 0.362258273 | TLR2 | 1 |
| BP | GO:0006605 | protein targeting | 1/30 | 339/18805 | 0.420837838 | 0.452873936 | 0.362258273 | SORT1 | 1 |
| BP | GO:0048872 | homeostasis of number of cells | 1/30 | 339/18805 | 0.420837838 | 0.452873936 | 0.362258273 | IL7R | 1 |
| BP | GO:1902105 | regulation of leukocyte differentiation | 1/30 | 340/18805 | 0.421778749 | 0.453363563 | 0.362649931 | IL7R | 1 |
| BP | GO:0060485 | mesenchyme development | 1/30 | 342/18805 | 0.42365614 | 0.454857513 | 0.363844956 | EPHA4 | 1 |
| BP | GO:0070661 | leukocyte proliferation | 1/30 | 344/18805 | 0.425527638 | 0.456341709 | 0.365032178 | IL7R | 1 |
| BP | GO:0062012 | regulation of small molecule metabolic process | 1/30 | 347/18805 | 0.428323875 | 0.45881306 | 0.367009037 | RORA | 1 |
| BP | GO:0009150 | purine ribonucleotide metabolic process | 1/30 | 350/18805 | 0.431106953 | 0.460736297 | 0.368547452 | RORA | 1 |
| BP | GO:0030111 | regulation of Wnt signaling pathway | 1/30 | 350/18805 | 0.431106953 | 0.460736297 | 0.368547452 | TLR2 | 1 |
| BP | GO:0006310 | DNA recombination | 1/30 | 352/18805 | 0.432955057 | 0.462182 | 0.369703885 | IL7R | 1 |
| BP | GO:0032535 | regulation of cellular component size | 1/30 | 353/18805 | 0.433876932 | 0.462636774 | 0.370067663 | IL7R | 1 |
| BP | GO:0032496 | response to lipopolysaccharide | 1/30 | 357/18805 | 0.437549963 | 0.466020679 | 0.372774481 | TLR2 | 1 |
| BP | GO:1901990 | regulation of mitotic cell cycle phase transition | 1/30 | 358/18805 | 0.438464615 | 0.466462355 | 0.373127782 | BCL7A | 1 |
| BP | GO:0006936 | muscle contraction | 1/30 | 359/18805 | 0.439377829 | 0.466901497 | 0.373479056 | TNNT1 | 1 |
| BP | GO:0031346 | positive regulation of cell projection organization | 1/30 | 360/18805 | 0.440289607 | 0.467338116 | 0.373828312 | EPHA4 | 1 |
| BP | GO:0006066 | alcohol metabolic process | 1/30 | 368/18805 | 0.44753241 | 0.474486066 | 0.379546027 | PNPLA2 | 1 |
| BP | GO:0009259 | ribonucleotide metabolic process | 1/30 | 371/18805 | 0.450225034 | 0.476664473 | 0.381288556 | RORA | 1 |
| BP | GO:0002696 | positive regulation of leukocyte activation | 1/30 | 372/18805 | 0.451119753 | 0.476664473 | 0.381288556 | IL7R | 1 |
| BP | GO:0009306 | protein secretion | 1/30 | 372/18805 | 0.451119753 | 0.476664473 | 0.381288556 | TLR2 | 1 |
| BP | GO:0016236 | macroautophagy | 1/30 | 374/18805 | 0.45290497 | 0.477868144 | 0.382251384 | CTSD | 1 |
| BP | GO:0035592 | establishment of protein localization to extracellular region | 1/30 | 375/18805 | 0.453795472 | 0.477868144 | 0.382251384 | TLR2 | 1 |
| BP | GO:0042176 | regulation of protein catabolic process | 1/30 | 375/18805 | 0.453795472 | 0.477868144 | 0.382251384 | XPO1 | 1 |
| BP | GO:0002237 | response to molecule of bacterial origin | 1/30 | 379/18805 | 0.457343491 | 0.480519682 | 0.384372375 | TLR2 | 1 |
| BP | GO:0019693 | ribose phosphate metabolic process | 1/30 | 379/18805 | 0.457343491 | 0.480519682 | 0.384372375 | RORA | 1 |
| BP | GO:0006644 | phospholipid metabolic process | 1/30 | 383/18805 | 0.460869227 | 0.483135942 | 0.386465147 | PLBD1 | 1 |
| BP | GO:0071692 | protein localization to extracellular region | 1/30 | 383/18805 | 0.460869227 | 0.483135942 | 0.386465147 | TLR2 | 1 |
| BP | GO:0030336 | negative regulation of cell migration | 1/30 | 388/18805 | 0.465245265 | 0.486995014 | 0.389552056 | EPHA4 | 1 |
| BP | GO:0001818 | negative regulation of cytokine production | 1/30 | 389/18805 | 0.466116343 | 0.486995014 | 0.389552056 | IL1R2 | 1 |
| BP | GO:0016050 | vesicle organization | 1/30 | 389/18805 | 0.466116343 | 0.486995014 | 0.389552056 | SORT1 | 1 |
| BP | GO:0050867 | positive regulation of cell activation | 1/30 | 392/18805 | 0.468721355 | 0.488053312 | 0.390398599 | IL7R | 1 |
| BP | GO:0030522 | intracellular receptor signaling pathway | 1/30 | 393/18805 | 0.469586958 | 0.488053312 | 0.390398599 | RORA | 1 |
| BP | GO:0008544 | epidermis development | 1/30 | 395/18805 | 0.471314077 | 0.488053312 | 0.390398599 | MAFG | 1 |
| BP | GO:0046486 | glycerolipid metabolic process | 1/30 | 395/18805 | 0.471314077 | 0.488053312 | 0.390398599 | PNPLA2 | 1 |
| BP | GO:0050863 | regulation of T cell activation | 1/30 | 395/18805 | 0.471314077 | 0.488053312 | 0.390398599 | IL7R | 1 |
| BP | GO:0050900 | leukocyte migration | 1/30 | 395/18805 | 0.471314077 | 0.488053312 | 0.390398599 | S100A12 | 1 |
| BP | GO:0060271 | cilium assembly | 1/30 | 395/18805 | 0.471314077 | 0.488053312 | 0.390398599 | IQCB1 | 1 |
| BP | GO:1903037 | regulation of leukocyte cell-cell adhesion | 1/30 | 395/18805 | 0.471314077 | 0.488053312 | 0.390398599 | IL7R | 1 |
| BP | GO:0141091 | transforming growth factor beta receptor superfamily signaling pathway | 1/30 | 399/18805 | 0.474752022 | 0.491068333 | 0.392810343 | FUT8 | 1 |
| BP | GO:0009100 | glycoprotein metabolic process | 1/30 | 400/18805 | 0.475608125 | 0.491409059 | 0.393082894 | FUT8 | 1 |
| BP | GO:2000146 | negative regulation of cell motility | 1/30 | 403/18805 | 0.47816835 | 0.493507821 | 0.394761714 | EPHA4 | 1 |
| BP | GO:0016032 | viral process | 1/30 | 421/18805 | 0.493277833 | 0.507978166 | 0.406336684 | FUT8 | 1 |
| BP | GO:0040013 | negative regulation of locomotion | 1/30 | 421/18805 | 0.493277833 | 0.507978166 | 0.406336684 | EPHA4 | 1 |
| BP | GO:0009615 | response to virus | 1/30 | 425/18805 | 0.4965776 | 0.510249891 | 0.408153859 | TLR2 | 1 |
| BP | GO:0044782 | cilium organization | 1/30 | 425/18805 | 0.4965776 | 0.510249891 | 0.408153859 | IQCB1 | 1 |
| BP | GO:0007178 | cell surface receptor protein serine/threonine kinase signaling pathway | 1/30 | 427/18805 | 0.498219685 | 0.51081205 | 0.408603535 | FUT8 | 1 |
| BP | GO:0140694 | membraneless organelle assembly | 1/30 | 427/18805 | 0.498219685 | 0.51081205 | 0.408603535 | TNNT1 | 1 |
| BP | GO:0022412 | cellular process involved in reproduction in multicellular organism | 1/30 | 429/18805 | 0.499856591 | 0.511927771 | 0.409496011 | RETN | 1 |
| BP | GO:0007059 | chromosome segregation | 1/30 | 431/18805 | 0.501488335 | 0.513035763 | 0.410382305 | BCL7A | 1 |
| BP | GO:0007159 | leukocyte cell-cell adhesion | 1/30 | 433/18805 | 0.503114932 | 0.514136069 | 0.411262451 | IL7R | 1 |
| BP | GO:1903706 | regulation of hemopoiesis | 1/30 | 436/18805 | 0.505545211 | 0.516054357 | 0.412796908 | IL7R | 1 |
| BP | GO:0006163 | purine nucleotide metabolic process | 1/30 | 448/18805 | 0.515151932 | 0.524712612 | 0.41972273 | RORA | 1 |
| BP | GO:0010975 | regulation of neuron projection development | 1/30 | 448/18805 | 0.515151932 | 0.524712612 | 0.41972273 | EPHA4 | 1 |
| BP | GO:0030099 | myeloid cell differentiation | 1/30 | 455/18805 | 0.520672264 | 0.529757058 | 0.423757832 | TLR2 | 1 |
| BP | GO:0044772 | mitotic cell cycle phase transition | 1/30 | 457/18805 | 0.52223831 | 0.530771615 | 0.424569386 | BCL7A | 1 |
| BP | GO:0001503 | ossification | 1/30 | 458/18805 | 0.523019477 | 0.530987129 | 0.424741778 | SORT1 | 1 |
| BP | GO:0007409 | axonogenesis | 1/30 | 459/18805 | 0.523799409 | 0.531200922 | 0.424912793 | EPHA4 | 1 |
| BP | GO:0006869 | lipid transport | 1/30 | 460/18805 | 0.524578108 | 0.531413002 | 0.425082438 | RETN | 1 |
| BP | GO:0043410 | positive regulation of MAPK cascade | 1/30 | 464/18805 | 0.527680614 | 0.533048856 | 0.426390973 | S100A12 | 1 |
| BP | GO:0006935 | chemotaxis | 1/30 | 466/18805 | 0.529224519 | 0.533048856 | 0.426390973 | S100A12 | 1 |
| BP | GO:0003012 | muscle system process | 1/30 | 467/18805 | 0.529994641 | 0.533048856 | 0.426390973 | TNNT1 | 1 |
| BP | GO:0043161 | proteasome-mediated ubiquitin-dependent protein catabolic process | 1/30 | 467/18805 | 0.529994641 | 0.533048856 | 0.426390973 | XPO1 | 1 |
| BP | GO:0051051 | negative regulation of transport | 1/30 | 467/18805 | 0.529994641 | 0.533048856 | 0.426390973 | TLR2 | 1 |
| BP | GO:0072594 | establishment of protein localization to organelle | 1/30 | 467/18805 | 0.529994641 | 0.533048856 | 0.426390973 | SORT1 | 1 |
| BP | GO:0042330 | taxis | 1/30 | 468/18805 | 0.530763545 | 0.533048856 | 0.426390973 | S100A12 | 1 |
| BP | GO:1901987 | regulation of cell cycle phase transition | 1/30 | 468/18805 | 0.530763545 | 0.533048856 | 0.426390973 | BCL7A | 1 |
| BP | GO:0015711 | organic anion transport | 1/30 | 469/18805 | 0.531531233 | 0.53324585 | 0.42654855 | MGST1 | 1 |
| BP | GO:0016055 | Wnt signaling pathway | 1/30 | 477/18805 | 0.537629182 | 0.538784132 | 0.430978676 | TLR2 | 1 |
| BP | GO:0043603 | amide metabolic process | 1/30 | 480/18805 | 0.539896072 | 0.540475359 | 0.432331505 | EPHA4 | 1 |
| BP | GO:0023061 | signal release | 1/30 | 497/18805 | 0.552540255 | 0.552540255 | 0.441982333 | RETN | 1 |
| CC | GO:0031904 | endosome lumen | 2/32 | 38/19880 | 0.001701931 | 0.178234626 | 0.136056966 | PDLIM4/CTSD | 2 |
| CC | GO:0035580 | specific granule lumen | 2/32 | 62/19880 | 0.004469207 | 0.178234626 | 0.136056966 | RETN/CTSD | 2 |
| CC | GO:0032580 | Golgi cisterna membrane | 2/32 | 94/19880 | 0.010005181 | 0.178234626 | 0.136056966 | FUT8/SORT1 | 2 |
| CC | GO:0045121 | membrane raft | 3/32 | 291/19880 | 0.011252517 | 0.178234626 | 0.136056966 | TLR2/CD8A/CTSD | 3 |
| CC | GO:0098857 | membrane microdomain | 3/32 | 293/19880 | 0.011462083 | 0.178234626 | 0.136056966 | TLR2/CD8A/CTSD | 3 |
| CC | GO:0034774 | secretory granule lumen | 3/32 | 324/19880 | 0.015002087 | 0.178234626 | 0.136056966 | S100A12/RETN/CTSD | 3 |
| CC | GO:0031985 | Golgi cisterna | 2/32 | 118/19880 | 0.015427233 | 0.178234626 | 0.136056966 | FUT8/SORT1 | 2 |
| CC | GO:0060205 | cytoplasmic vesicle lumen | 3/32 | 329/19880 | 0.015624901 | 0.178234626 | 0.136056966 | S100A12/RETN/CTSD | 3 |
| CC | GO:0031983 | vesicle lumen | 3/32 | 330/19880 | 0.015751207 | 0.178234626 | 0.136056966 | S100A12/RETN/CTSD | 3 |
| CC | GO:0008303 | caspase complex | 1/32 | 10/19880 | 0.015984076 | 0.178234626 | 0.136056966 | CAPNS2 | 1 |
| CC | GO:0140288 | GBAF complex | 1/32 | 14/19880 | 0.02230816 | 0.178234626 | 0.136056966 | BCL7A | 1 |
| CC | GO:0005777 | peroxisome | 2/32 | 147/19880 | 0.023300345 | 0.178234626 | 0.136056966 | CRAT/MGST1 | 2 |
| CC | GO:0042579 | microbody | 2/32 | 147/19880 | 0.023300345 | 0.178234626 | 0.136056966 | CRAT/MGST1 | 2 |
| CC | GO:0005795 | Golgi stack | 2/32 | 152/19880 | 0.024794549 | 0.178234626 | 0.136056966 | FUT8/SORT1 | 2 |
| CC | GO:0098985 | asymmetric, glutamatergic, excitatory synapse | 1/32 | 16/19880 | 0.02545542 | 0.178234626 | 0.136056966 | SORT1 | 1 |
| CC | GO:0005766 | primary lysosome | 2/32 | 155/19880 | 0.025709563 | 0.178234626 | 0.136056966 | RETN/MGST1 | 2 |
| CC | GO:0042582 | azurophil granule | 2/32 | 155/19880 | 0.025709563 | 0.178234626 | 0.136056966 | RETN/MGST1 | 2 |
| CC | GO:0009897 | external side of plasma membrane | 3/32 | 401/19880 | 0.026219 | 0.178234626 | 0.136056966 | KLRC3/CD8A/IL7R | 3 |
| CC | GO:0042581 | specific granule | 2/32 | 160/19880 | 0.02726494 | 0.178234626 | 0.136056966 | RETN/CTSD | 2 |
| CC | GO:0005775 | vacuolar lumen | 2/32 | 178/19880 | 0.033168968 | 0.178234626 | 0.136056966 | RETN/CTSD | 2 |
| CC | GO:0043197 | dendritic spine | 2/32 | 178/19880 | 0.033168968 | 0.178234626 | 0.136056966 | PDLIM4/EPHA4 | 2 |
| CC | GO:0005769 | early endosome | 3/32 | 440/19880 | 0.033234487 | 0.178234626 | 0.136056966 | PDLIM4/SORT1/EPHA4 | 3 |
| CC | GO:0044309 | neuron spine | 2/32 | 180/19880 | 0.03385354 | 0.178234626 | 0.136056966 | PDLIM4/EPHA4 | 2 |
| CC | GO:0005865 | striated muscle thin filament | 1/32 | 22/19880 | 0.034838423 | 0.178234626 | 0.136056966 | TNNT1 | 1 |
| CC | GO:0005765 | lysosomal membrane | 3/32 | 451/19880 | 0.035374811 | 0.178234626 | 0.136056966 | SORT1/MGST1/CTSD | 3 |
| CC | GO:0098852 | lytic vacuole membrane | 3/32 | 451/19880 | 0.035374811 | 0.178234626 | 0.136056966 | SORT1/MGST1/CTSD | 3 |
| CC | GO:0005912 | adherens junction | 2/32 | 189/19880 | 0.037002383 | 0.179530079 | 0.137045862 | PDLIM4/EPHA4 | 2 |
| CC | GO:0031901 | early endosome membrane | 2/32 | 200/19880 | 0.040998651 | 0.179957058 | 0.1373718 | PDLIM4/EPHA4 | 2 |
| CC | GO:0036379 | myofilament | 1/32 | 26/19880 | 0.041045073 | 0.179957058 | 0.1373718 | TNNT1 | 1 |
| CC | GO:0030666 | endocytic vesicle membrane | 2/32 | 207/19880 | 0.043623534 | 0.179957058 | 0.1373718 | TLR2/IL7R | 2 |
| CC | GO:0044295 | axonal growth cone | 1/32 | 28/19880 | 0.044133882 | 0.179957058 | 0.1373718 | EPHA4 | 1 |
| CC | GO:0005774 | vacuolar membrane | 3/32 | 495/19880 | 0.044638447 | 0.179957058 | 0.1373718 | SORT1/MGST1/CTSD | 3 |
| CC | GO:0031941 | filamentous actin | 1/32 | 29/19880 | 0.045674669 | 0.179957058 | 0.1373718 | PDLIM4 | 1 |
| CC | GO:0016514 | SWI/SNF complex | 1/32 | 30/19880 | 0.047213051 | 0.179957058 | 0.1373718 | BCL7A | 1 |
| CC | GO:0030017 | sarcomere | 2/32 | 222/19880 | 0.049453848 | 0.179957058 | 0.1373718 | PDLIM4/TNNT1 | 2 |
| CC | GO:0030136 | clathrin-coated vesicle | 2/32 | 222/19880 | 0.049453848 | 0.179957058 | 0.1373718 | SORT1/IL7R | 2 |
| CC | GO:0005741 | mitochondrial outer membrane | 2/32 | 228/19880 | 0.051861715 | 0.183618503 | 0.140166796 | EPHA4/MGST1 | 2 |
| CC | GO:0030016 | myofibril | 2/32 | 244/19880 | 0.0584844 | 0.194973455 | 0.148834699 | PDLIM4/TNNT1 | 2 |
| CC | GO:0030140 | trans-Golgi network transport vesicle | 1/32 | 38/19880 | 0.059433917 | 0.194973455 | 0.148834699 | SORT1 | 1 |
| CC | GO:0043198 | dendritic shaft | 1/32 | 40/19880 | 0.062465325 | 0.194973455 | 0.148834699 | EPHA4 | 1 |
| CC | GO:0043292 | contractile muscle fiber | 2/32 | 254/19880 | 0.062766428 | 0.194973455 | 0.148834699 | PDLIM4/TNNT1 | 2 |
| CC | GO:0031968 | organelle outer membrane | 2/32 | 257/19880 | 0.064071646 | 0.194973455 | 0.148834699 | EPHA4/MGST1 | 2 |
| CC | GO:0019867 | outer membrane | 2/32 | 259/19880 | 0.06494697 | 0.194973455 | 0.148834699 | EPHA4/MGST1 | 2 |
| CC | GO:0032391 | photoreceptor connecting cilium | 1/32 | 42/19880 | 0.065487267 | 0.194973455 | 0.148834699 | IQCB1 | 1 |
| CC | GO:0005782 | peroxisomal matrix | 1/32 | 52/19880 | 0.080455992 | 0.229124673 | 0.174904331 | CRAT | 1 |
| CC | GO:0031907 | microbody lumen | 1/32 | 52/19880 | 0.080455992 | 0.229124673 | 0.174904331 | CRAT | 1 |
| CC | GO:1904724 | tertiary granule lumen | 1/32 | 55/19880 | 0.084901134 | 0.236639331 | 0.180640711 | CTSD | 1 |
| CC | GO:0035577 | azurophil granule membrane | 1/32 | 58/19880 | 0.089325456 | 0.237153915 | 0.181033523 | MGST1 | 1 |
| CC | GO:0031965 | nuclear membrane | 2/32 | 312/19880 | 0.089537325 | 0.237153915 | 0.181033523 | SORT1/XPO1 | 2 |
| CC | GO:0045211 | postsynaptic membrane | 2/32 | 321/19880 | 0.093956584 | 0.237153915 | 0.181033523 | PDLIM4/EPHA4 | 2 |
| CC | GO:0030135 | coated vesicle | 2/32 | 322/19880 | 0.094451581 | 0.237153915 | 0.181033523 | SORT1/IL7R | 2 |
| CC | GO:0030667 | secretory granule membrane | 2/32 | 322/19880 | 0.094451581 | 0.237153915 | 0.181033523 | TLR2/MGST1 | 2 |
| CC | GO:0098802 | plasma membrane signaling receptor complex | 2/32 | 326/19880 | 0.096439338 | 0.237153915 | 0.181033523 | TLR2/CD8A | 2 |
| CC | GO:0005778 | peroxisomal membrane | 1/32 | 65/19880 | 0.099568438 | 0.237153915 | 0.181033523 | MGST1 | 1 |
| CC | GO:0031903 | microbody membrane | 1/32 | 65/19880 | 0.099568438 | 0.237153915 | 0.181033523 | MGST1 | 1 |
| CC | GO:0060076 | excitatory synapse | 1/32 | 70/19880 | 0.106816428 | 0.242718874 | 0.185281583 | SORT1 | 1 |
| CC | GO:0035869 | ciliary transition zone | 1/32 | 74/19880 | 0.112574096 | 0.242718874 | 0.185281583 | IQCB1 | 1 |
| CC | GO:0030139 | endocytic vesicle | 2/32 | 358/19880 | 0.112763858 | 0.242718874 | 0.185281583 | TLR2/IL7R | 2 |
| CC | GO:0030669 | clathrin-coated endocytic vesicle membrane | 1/32 | 75/19880 | 0.114007885 | 0.242718874 | 0.185281583 | IL7R | 1 |
| CC | GO:0005905 | clathrin-coated pit | 1/32 | 78/19880 | 0.1182958 | 0.242718874 | 0.185281583 | SORT1 | 1 |
| CC | GO:0031594 | neuromuscular junction | 1/32 | 78/19880 | 0.1182958 | 0.242718874 | 0.185281583 | EPHA4 | 1 |
| CC | GO:0032279 | asymmetric synapse | 2/32 | 376/19880 | 0.122248922 | 0.242718874 | 0.185281583 | SORT1/EPHA4 | 2 |
| CC | GO:0015030 | Cajal body | 1/32 | 82/19880 | 0.12398176 | 0.242718874 | 0.185281583 | XPO1 | 1 |
| CC | GO:0030670 | phagocytic vesicle membrane | 1/32 | 82/19880 | 0.12398176 | 0.242718874 | 0.185281583 | TLR2 | 1 |
| CC | GO:0001725 | stress fiber | 1/32 | 85/19880 | 0.128222902 | 0.242718874 | 0.185281583 | PDLIM4 | 1 |
| CC | GO:0097517 | contractile actin filament bundle | 1/32 | 85/19880 | 0.128222902 | 0.242718874 | 0.185281583 | PDLIM4 | 1 |
| CC | GO:0098791 | Golgi apparatus subcompartment | 2/32 | 388/19880 | 0.128681176 | 0.242718874 | 0.185281583 | FUT8/SORT1 | 2 |
| CC | GO:0035578 | azurophil granule lumen | 1/32 | 91/19880 | 0.136645593 | 0.242718874 | 0.185281583 | RETN | 1 |
| CC | GO:0042641 | actomyosin | 1/32 | 91/19880 | 0.136645593 | 0.242718874 | 0.185281583 | PDLIM4 | 1 |
| CC | GO:0045171 | intercellular bridge | 1/32 | 92/19880 | 0.138041689 | 0.242718874 | 0.185281583 | IQCB1 | 1 |
| CC | GO:0045334 | clathrin-coated endocytic vesicle | 1/32 | 93/19880 | 0.139435598 | 0.242718874 | 0.185281583 | IL7R | 1 |
| CC | GO:0001750 | photoreceptor outer segment | 1/32 | 94/19880 | 0.140827323 | 0.242718874 | 0.185281583 | IQCB1 | 1 |
| CC | GO:0005798 | Golgi-associated vesicle | 1/32 | 95/19880 | 0.142216867 | 0.242718874 | 0.185281583 | SORT1 | 1 |
| CC | GO:0032432 | actin filament bundle | 1/32 | 95/19880 | 0.142216867 | 0.242718874 | 0.185281583 | PDLIM4 | 1 |
| CC | GO:0070603 | SWI/SNF superfamily-type complex | 1/32 | 95/19880 | 0.142216867 | 0.242718874 | 0.185281583 | BCL7A | 1 |
| CC | GO:0098984 | neuron to neuron synapse | 2/32 | 414/19880 | 0.142888106 | 0.242718874 | 0.185281583 | SORT1/EPHA4 | 2 |
| CC | GO:0043202 | lysosomal lumen | 1/32 | 98/19880 | 0.14637245 | 0.242718874 | 0.185281583 | CTSD | 1 |
| CC | GO:0098685 | Schaffer collateral - CA1 synapse | 1/32 | 98/19880 | 0.14637245 | 0.242718874 | 0.185281583 | EPHA4 | 1 |
| CC | GO:1905369 | endopeptidase complex | 1/32 | 98/19880 | 0.14637245 | 0.242718874 | 0.185281583 | CAPNS2 | 1 |
| CC | GO:0005811 | lipid droplet | 1/32 | 106/19880 | 0.157358885 | 0.253104608 | 0.193209624 | PNPLA2 | 1 |
| CC | GO:0030175 | filopodium | 1/32 | 107/19880 | 0.158722519 | 0.253104608 | 0.193209624 | EPHA4 | 1 |
| CC | GO:0055038 | recycling endosome membrane | 1/32 | 109/19880 | 0.161443379 | 0.253104608 | 0.193209624 | PDLIM4 | 1 |
| CC | GO:0042470 | melanosome | 1/32 | 111/19880 | 0.164155712 | 0.253104608 | 0.193209624 | CTSD | 1 |
| CC | GO:0048770 | pigment granule | 1/32 | 111/19880 | 0.164155712 | 0.253104608 | 0.193209624 | CTSD | 1 |
| CC | GO:0097060 | synaptic membrane | 2/32 | 452/19880 | 0.164228181 | 0.253104608 | 0.193209624 | PDLIM4/EPHA4 | 2 |
| CC | GO:0043679 | axon terminus | 1/32 | 115/19880 | 0.169554906 | 0.254407085 | 0.194203882 | EPHA4 | 1 |
| CC | GO:0044853 | plasma membrane raft | 1/32 | 115/19880 | 0.169554906 | 0.254407085 | 0.194203882 | CD8A | 1 |
| CC | GO:0005884 | actin filament | 1/32 | 116/19880 | 0.170899416 | 0.254407085 | 0.194203882 | PDLIM4 | 1 |
| CC | GO:0097733 | photoreceptor cell cilium | 1/32 | 120/19880 | 0.1762564 | 0.259433577 | 0.198040899 | IQCB1 | 1 |
| CC | GO:1904813 | ficolin-1-rich granule lumen | 1/32 | 124/19880 | 0.181579846 | 0.262173216 | 0.200132226 | CTSD | 1 |
| CC | GO:0098839 | postsynaptic density membrane | 1/32 | 126/19880 | 0.184229056 | 0.262173216 | 0.200132226 | EPHA4 | 1 |
| CC | GO:1904949 | ATPase complex | 1/32 | 127/19880 | 0.185550544 | 0.262173216 | 0.200132226 | BCL7A | 1 |
| CC | GO:1905368 | peptidase complex | 1/32 | 129/19880 | 0.188187301 | 0.262173216 | 0.200132226 | CAPNS2 | 1 |
| CC | GO:0005635 | nuclear envelope | 2/32 | 496/19880 | 0.189617933 | 0.262173216 | 0.200132226 | SORT1/XPO1 | 2 |
| CC | GO:0044306 | neuron projection terminus | 1/32 | 131/19880 | 0.190815787 | 0.262173216 | 0.200132226 | EPHA4 | 1 |
| CC | GO:0097731 | 9+0 non-motile cilium | 1/32 | 132/19880 | 0.192126937 | 0.262173216 | 0.200132226 | IQCB1 | 1 |
| CC | GO:0030018 | Z disc | 1/32 | 134/19880 | 0.194743065 | 0.26300352 | 0.200766046 | PDLIM4 | 1 |
| CC | GO:0030665 | clathrin-coated vesicle membrane | 1/32 | 138/19880 | 0.199950722 | 0.267281067 | 0.204031349 | IL7R | 1 |
| CC | GO:0045335 | phagocytic vesicle | 1/32 | 145/19880 | 0.208985719 | 0.273771291 | 0.208985719 | TLR2 | 1 |
| CC | GO:0072562 | blood microparticle | 1/32 | 145/19880 | 0.208985719 | 0.273771291 | 0.208985719 | C1RL | 1 |
| CC | GO:0031674 | I band | 1/32 | 150/19880 | 0.215378688 | 0.276613806 | 0.211155577 | PDLIM4 | 1 |
| CC | GO:0042101 | T cell receptor complex | 1/32 | 150/19880 | 0.215378688 | 0.276613806 | 0.211155577 | CD8A | 1 |
| CC | GO:0043204 | perikaryon | 1/32 | 152/19880 | 0.217921836 | 0.277162724 | 0.211574598 | EPHA4 | 1 |
| CC | GO:0099634 | postsynaptic specialization membrane | 1/32 | 156/19880 | 0.222984198 | 0.280874327 | 0.214407883 | EPHA4 | 1 |
| CC | GO:0005814 | centriole | 1/32 | 164/19880 | 0.233013862 | 0.287969962 | 0.219824398 | IQCB1 | 1 |
| CC | GO:0070820 | tertiary granule | 1/32 | 164/19880 | 0.233013862 | 0.287969962 | 0.219824398 | CTSD | 1 |
| CC | GO:0030426 | growth cone | 1/32 | 166/19880 | 0.235501614 | 0.288324406 | 0.220094967 | EPHA4 | 1 |
| CC | GO:0097730 | non-motile cilium | 1/32 | 169/19880 | 0.239218592 | 0.290163292 | 0.221498696 | IQCB1 | 1 |
| CC | GO:0030427 | site of polarized growth | 1/32 | 171/19880 | 0.241686844 | 0.290467675 | 0.22173105 | EPHA4 | 1 |
| CC | GO:0000776 | kinetochore | 1/32 | 174/19880 | 0.245374683 | 0.29221894 | 0.223067893 | XPO1 | 1 |
| CC | GO:0042734 | presynaptic membrane | 1/32 | 181/19880 | 0.2539122 | 0.298404175 | 0.227789447 | EPHA4 | 1 |
| CC | GO:0072686 | mitotic spindle | 1/32 | 182/19880 | 0.25512418 | 0.298404175 | 0.227789447 | IQCB1 | 1 |
| CC | GO:0101002 | ficolin-1-rich granule | 1/32 | 185/19880 | 0.25874869 | 0.298717989 | 0.228028999 | CTSD | 1 |
| CC | GO:0000779 | condensed chromosome, centromeric region | 1/32 | 186/19880 | 0.259953059 | 0.298717989 | 0.228028999 | XPO1 | 1 |
| CC | GO:0030027 | lamellipodium | 1/32 | 203/19880 | 0.280139617 | 0.319006613 | 0.243516499 | PDLIM4 | 1 |
| CC | GO:0030662 | coated vesicle membrane | 1/32 | 205/19880 | 0.282479139 | 0.319006613 | 0.243516499 | IL7R | 1 |
| CC | GO:0055037 | recycling endosome | 1/32 | 211/19880 | 0.289453595 | 0.324089068 | 0.247396235 | PDLIM4 | 1 |
| CC | GO:0098858 | actin-based cell projection | 1/32 | 235/19880 | 0.316700437 | 0.351591163 | 0.268390201 | EPHA4 | 1 |
| CC | GO:0090575 | RNA polymerase II transcription regulator complex | 1/32 | 254/19880 | 0.337550343 | 0.371589033 | 0.28365575 | MAFG | 1 |
| CC | GO:0000775 | chromosome, centromeric region | 1/32 | 265/19880 | 0.349338227 | 0.381360897 | 0.291115189 | XPO1 | 1 |
| CC | GO:0150034 | distal axon | 1/32 | 277/19880 | 0.361965972 | 0.391880516 | 0.299145432 | EPHA4 | 1 |
| CC | GO:0000793 | condensed chromosome | 1/32 | 285/19880 | 0.370252224 | 0.397565912 | 0.303485429 | XPO1 | 1 |
| CC | GO:0005788 | endoplasmic reticulum lumen | 1/32 | 315/19880 | 0.400407116 | 0.426449855 | 0.325534241 | PNPLA2 | 1 |
| CC | GO:0014069 | postsynaptic density | 1/32 | 360/19880 | 0.443032549 | 0.468042451 | 0.357284314 | EPHA4 | 1 |
| CC | GO:0099572 | postsynaptic specialization | 1/32 | 396/19880 | 0.475004904 | 0.49780514 | 0.380003923 | EPHA4 | 1 |
| CC | GO:0098687 | chromosomal region | 1/32 | 408/19880 | 0.485261674 | 0.50451809 | 0.385128313 | XPO1 | 1 |
| CC | GO:0016607 | nuclear speck | 1/32 | 415/19880 | 0.491154859 | 0.506624303 | 0.386736109 | PYHIN1 | 1 |
| CC | GO:0062023 | collagen-containing extracellular matrix | 1/32 | 432/19880 | 0.505196051 | 0.511539512 | 0.390488177 | CTSD | 1 |
| CC | GO:0031252 | cell leading edge | 1/32 | 434/19880 | 0.506823067 | 0.511539512 | 0.390488177 | PDLIM4 | 1 |
| CC | GO:0005819 | spindle | 1/32 | 435/19880 | 0.507634631 | 0.511539512 | 0.390488177 | IQCB1 | 1 |
| CC | GO:0030133 | transport vesicle | 1/32 | 444/19880 | 0.514880718 | 0.514880718 | 0.393038716 | SORT1 | 1 |
| MF | GO:0005030 | neurotrophin receptor activity | 2/30 | 51/18639 | 0.00304037 | 0.117124751 | 0.088063723 | SORT1/EPHA4 | 2 |
| MF | GO:0008234 | cysteine-type peptidase activity | 3/30 | 187/18639 | 0.003305415 | 0.117124751 | 0.088063723 | OTUD3/CAPNS2/CTSD | 3 |
| MF | GO:0008374 | O-acyltransferase activity | 2/30 | 55/18639 | 0.003527033 | 0.117124751 | 0.088063723 | PNPLA2/CRAT | 2 |
| MF | GO:0004175 | endopeptidase activity | 4/30 | 405/18639 | 0.003848763 | 0.117124751 | 0.088063723 | C1RL/CAPNS2/CTSD/GZMK | 4 |
| MF | GO:0016627 | oxidoreductase activity, acting on the CH-CH group of donors | 2/30 | 60/18639 | 0.004183027 | 0.117124751 | 0.088063723 | CRAT/TBXAS1 | 2 |
| MF | GO:0001540 | amyloid-beta binding | 2/30 | 83/18639 | 0.007860127 | 0.139863826 | 0.105160772 | TLR2/EPHA4 | 2 |
| MF | GO:0004197 | cysteine-type endopeptidase activity | 2/30 | 88/18639 | 0.008797904 | 0.139863826 | 0.105160772 | CAPNS2/CTSD | 2 |
| MF | GO:0004896 | cytokine receptor activity | 2/30 | 96/18639 | 0.01039723 | 0.139863826 | 0.105160772 | IL1R2/IL7R | 2 |
| MF | GO:0004620 | phospholipase activity | 2/30 | 109/18639 | 0.013248444 | 0.139863826 | 0.105160772 | PNPLA2/PLBD1 | 2 |
| MF | GO:0016634 | oxidoreductase activity, acting on the CH-CH group of donors, oxygen as acceptor | 1/30 | 10/18639 | 0.015983038 | 0.139863826 | 0.105160772 | CRAT | 1 |
| MF | GO:0043121 | neurotrophin binding | 1/30 | 10/18639 | 0.015983038 | 0.139863826 | 0.105160772 | SORT1 | 1 |
| MF | GO:0071723 | lipopeptide binding | 1/30 | 10/18639 | 0.015983038 | 0.139863826 | 0.105160772 | TLR2 | 1 |
| MF | GO:1905394 | retromer complex binding | 1/30 | 10/18639 | 0.015983038 | 0.139863826 | 0.105160772 | SORT1 | 1 |
| MF | GO:0050786 | RAGE receptor binding | 1/30 | 11/18639 | 0.017567692 | 0.139863826 | 0.105160772 | S100A12 | 1 |
| MF | GO:0016298 | lipase activity | 2/30 | 130/18639 | 0.018484271 | 0.139863826 | 0.105160772 | PNPLA2/PLBD1 | 2 |
| MF | GO:0005049 | nuclear export signal receptor activity | 1/30 | 12/18639 | 0.019149878 | 0.139863826 | 0.105160772 | XPO1 | 1 |
| MF | GO:0019838 | growth factor binding | 2/30 | 136/18639 | 0.020116885 | 0.139863826 | 0.105160772 | SORT1/IL1R2 | 2 |
| MF | GO:0004465 | lipoprotein lipase activity | 1/30 | 13/18639 | 0.020729601 | 0.139863826 | 0.105160772 | PNPLA2 | 1 |
| MF | GO:0008417 | fucosyltransferase activity | 1/30 | 13/18639 | 0.020729601 | 0.139863826 | 0.105160772 | FUT8 | 1 |
| MF | GO:0035325 | Toll-like receptor binding | 1/30 | 13/18639 | 0.020729601 | 0.139863826 | 0.105160772 | TLR2 | 1 |
| MF | GO:0005523 | tropomyosin binding | 1/30 | 15/18639 | 0.023881673 | 0.139863826 | 0.105160772 | TNNT1 | 1 |
| MF | GO:0051371 | muscle alpha-actinin binding | 1/30 | 15/18639 | 0.023881673 | 0.139863826 | 0.105160772 | PDLIM4 | 1 |
| MF | GO:0004198 | calcium-dependent cysteine-type endopeptidase activity | 1/30 | 16/18639 | 0.025454028 | 0.139863826 | 0.105160772 | CAPNS2 | 1 |
| MF | GO:0008142 | oxysterol binding | 1/30 | 16/18639 | 0.025454028 | 0.139863826 | 0.105160772 | RORA | 1 |
| MF | GO:0061809 | NAD+ nucleosidase activity, cyclic ADP-ribose generating | 1/30 | 16/18639 | 0.025454028 | 0.139863826 | 0.105160772 | TLR2 | 1 |
| MF | GO:0140375 | immune receptor activity | 2/30 | 156/18639 | 0.025974711 | 0.139863826 | 0.105160772 | IL1R2/IL7R | 2 |
| MF | GO:0003746 | translation elongation factor activity | 1/30 | 18/18639 | 0.028591397 | 0.14825169 | 0.111467436 | EIF5A2 | 1 |
| MF | GO:0042834 | peptidoglycan binding | 1/30 | 19/18639 | 0.030156418 | 0.148982835 | 0.112017169 | TLR2 | 1 |
| MF | GO:0042288 | MHC class I protein binding | 1/30 | 20/18639 | 0.031719002 | 0.148982835 | 0.112017169 | CD8A | 1 |
| MF | GO:0004252 | serine-type endopeptidase activity | 2/30 | 176/18639 | 0.032439006 | 0.148982835 | 0.112017169 | C1RL/GZMK | 2 |
| MF | GO:0004602 | glutathione peroxidase activity | 1/30 | 23/18639 | 0.036392165 | 0.148982835 | 0.112017169 | MGST1 | 1 |
| MF | GO:0140103 | catalytic activity, acting on a glycoprotein | 1/30 | 23/18639 | 0.036392165 | 0.148982835 | 0.112017169 | FUT8 | 1 |
| MF | GO:0004806 | triacylglycerol lipase activity | 1/30 | 24/18639 | 0.037945035 | 0.148982835 | 0.112017169 | PNPLA2 | 1 |
| MF | GO:0008236 | serine-type peptidase activity | 2/30 | 194/18639 | 0.038740916 | 0.148982835 | 0.112017169 | C1RL/GZMK | 2 |
| MF | GO:0017171 | serine hydrolase activity | 2/30 | 198/18639 | 0.040200515 | 0.148982835 | 0.112017169 | C1RL/GZMK | 2 |
| MF | GO:0004364 | glutathione transferase activity | 1/30 | 27/18639 | 0.042589146 | 0.148982835 | 0.112017169 | MGST1 | 1 |
| MF | GO:0046875 | ephrin receptor binding | 1/30 | 28/18639 | 0.044132361 | 0.148982835 | 0.112017169 | EPHA4 | 1 |
| MF | GO:0051393 | alpha-actinin binding | 1/30 | 28/18639 | 0.044132361 | 0.148982835 | 0.112017169 | PDLIM4 | 1 |
| MF | GO:0016411 | acylglycerol O-acyltransferase activity | 1/30 | 31/18639 | 0.048747594 | 0.148982835 | 0.112017169 | PNPLA2 | 1 |
| MF | GO:0047499 | calcium-independent phospholipase A2 activity | 1/30 | 31/18639 | 0.048747594 | 0.148982835 | 0.112017169 | PNPLA2 | 1 |
| MF | GO:0047498 | calcium-dependent phospholipase A2 activity | 1/30 | 32/18639 | 0.050281212 | 0.148982835 | 0.112017169 | PNPLA2 | 1 |
| MF | GO:0140142 | nucleocytoplasmic carrier activity | 1/30 | 32/18639 | 0.050281212 | 0.148982835 | 0.112017169 | XPO1 | 1 |
| MF | GO:0016628 | oxidoreductase activity, acting on the CH-CH group of donors, NAD or NADP as acceptor | 1/30 | 33/18639 | 0.051812441 | 0.148982835 | 0.112017169 | TBXAS1 | 1 |
| MF | GO:0016747 | acyltransferase activity, transferring groups other than amino-acyl groups | 1/30 | 229/18639 | 0.052195772 | 0.148982835 | 0.112017169 | PNPLA2/CRAT | 2 |
| MF | GO:0004623 | phospholipase A2 activity | 1/30 | 35/18639 | 0.054867741 | 0.148982835 | 0.112017169 | PNPLA2 | 1 |
| MF | GO:0004190 | aspartic-type endopeptidase activity | 1/30 | 36/18639 | 0.05639182 | 0.148982835 | 0.112017169 | CTSD | 1 |
| MF | GO:0023023 | MHC protein complex binding | 1/30 | 37/18639 | 0.057913523 | 0.148982835 | 0.112017169 | CD8A | 1 |
| MF | GO:0042805 | actinin binding | 1/30 | 37/18639 | 0.057913523 | 0.148982835 | 0.112017169 | PDLIM4 | 1 |
| MF | GO:0070001 | aspartic-type peptidase activity | 1/30 | 37/18639 | 0.057913523 | 0.148982835 | 0.112017169 | CTSD | 1 |
| MF | GO:0033218 | amide binding | 1/30 | 247/18639 | 0.059680216 | 0.148982835 | 0.112017169 | TLR2/EPHA4 | 2 |
| MF | GO:0042277 | peptide binding | 1/30 | 249/18639 | 0.060533856 | 0.148982835 | 0.112017169 | TLR2/EPHA4 | 2 |
| MF | GO:0016799 | hydrolase activity, hydrolyzing N-glycosyl compounds | 1/30 | 39/18639 | 0.060949816 | 0.148982835 | 0.112017169 | TLR2 | 1 |
| MF | GO:0001530 | lipopolysaccharide binding | 1/30 | 41/18639 | 0.063976649 | 0.148982835 | 0.112017169 | TLR2 | 1 |
| MF | GO:0042287 | MHC protein binding | 1/30 | 41/18639 | 0.063976649 | 0.148982835 | 0.112017169 | CD8A | 1 |
| MF | GO:0001223 | transcription coactivator binding | 1/30 | 44/18639 | 0.068499221 | 0.148982835 | 0.112017169 | RORA | 1 |
| MF | GO:0038187 | pattern recognition receptor activity | 1/30 | 48/18639 | 0.074496471 | 0.148982835 | 0.112017169 | TLR2 | 1 |
| MF | GO:0001222 | transcription corepressor binding | 1/30 | 49/18639 | 0.075989941 | 0.148982835 | 0.112017169 | RORA | 1 |
| MF | GO:0005004 | GPI-linked ephrin receptor activity | 1/30 | 49/18639 | 0.075989941 | 0.148982835 | 0.112017169 | EPHA4 | 1 |
| MF | GO:0005008 | hepatocyte growth factor receptor activity | 1/30 | 49/18639 | 0.075989941 | 0.148982835 | 0.112017169 | EPHA4 | 1 |
| MF | GO:0005009 | insulin receptor activity | 1/30 | 49/18639 | 0.075989941 | 0.148982835 | 0.112017169 | EPHA4 | 1 |
| MF | GO:0005011 | macrophage colony-stimulating factor receptor activity | 1/30 | 49/18639 | 0.075989941 | 0.148982835 | 0.112017169 | EPHA4 | 1 |
| MF | GO:0005018 | platelet-derived growth factor alpha-receptor activity | 1/30 | 49/18639 | 0.075989941 | 0.148982835 | 0.112017169 | EPHA4 | 1 |
| MF | GO:0005020 | stem cell factor receptor activity | 1/30 | 49/18639 | 0.075989941 | 0.148982835 | 0.112017169 | EPHA4 | 1 |
| MF | GO:0008288 | boss receptor activity | 1/30 | 49/18639 | 0.075989941 | 0.148982835 | 0.112017169 | EPHA4 | 1 |
| MF | GO:0036332 | placental growth factor receptor activity | 1/30 | 49/18639 | 0.075989941 | 0.148982835 | 0.112017169 | EPHA4 | 1 |
| MF | GO:0038062 | protein tyrosine kinase collagen receptor activity | 1/30 | 49/18639 | 0.075989941 | 0.148982835 | 0.112017169 | EPHA4 | 1 |
| MF | GO:0060175 | brain-derived neurotrophic factor receptor activity | 1/30 | 49/18639 | 0.075989941 | 0.148982835 | 0.112017169 | EPHA4 | 1 |
| MF | GO:0005006 | epidermal growth factor receptor activity | 1/30 | 50/18639 | 0.077481081 | 0.148982835 | 0.112017169 | EPHA4 | 1 |
| MF | GO:0005007 | fibroblast growth factor receptor activity | 1/30 | 50/18639 | 0.077481081 | 0.148982835 | 0.112017169 | EPHA4 | 1 |
| MF | GO:0005017 | platelet-derived growth factor receptor activity | 1/30 | 50/18639 | 0.077481081 | 0.148982835 | 0.112017169 | EPHA4 | 1 |
| MF | GO:0005019 | platelet-derived growth factor beta-receptor activity | 1/30 | 50/18639 | 0.077481081 | 0.148982835 | 0.112017169 | EPHA4 | 1 |
| MF | GO:0005010 | insulin-like growth factor receptor activity | 1/30 | 51/18639 | 0.078969896 | 0.148982835 | 0.112017169 | EPHA4 | 1 |
| MF | GO:0005021 | vascular endothelial growth factor receptor activity | 1/30 | 51/18639 | 0.078969896 | 0.148982835 | 0.112017169 | EPHA4 | 1 |
| MF | GO:0005003 | ephrin receptor activity | 1/30 | 53/18639 | 0.081940559 | 0.148982835 | 0.112017169 | EPHA4 | 1 |
| MF | GO:0005005 | transmembrane-ephrin receptor activity | 1/30 | 53/18639 | 0.081940559 | 0.148982835 | 0.112017169 | EPHA4 | 1 |
| MF | GO:0016860 | intramolecular oxidoreductase activity | 1/30 | 53/18639 | 0.081940559 | 0.148982835 | 0.112017169 | TBXAS1 | 1 |
| MF | GO:0038064 | collagen receptor activity | 1/30 | 53/18639 | 0.081940559 | 0.148982835 | 0.112017169 | EPHA4 | 1 |
| MF | GO:0004601 | peroxidase activity | 1/30 | 57/18639 | 0.087854125 | 0.154380242 | 0.11607537 | MGST1 | 1 |
| MF | GO:0004879 | nuclear receptor activity | 1/30 | 59/18639 | 0.090797083 | 0.154380242 | 0.11607537 | RORA | 1 |
| MF | GO:0016684 | oxidoreductase activity, acting on peroxide as acceptor | 1/30 | 59/18639 | 0.090797083 | 0.154380242 | 0.11607537 | MGST1 | 1 |
| MF | GO:0004714 | transmembrane receptor protein tyrosine kinase activity | 1/30 | 60/18639 | 0.092265118 | 0.154380242 | 0.11607537 | EPHA4 | 1 |
| MF | GO:0098531 | ligand-modulated transcription factor activity | 1/30 | 60/18639 | 0.092265118 | 0.154380242 | 0.11607537 | RORA | 1 |
| MF | GO:0015026 | coreceptor activity | 1/30 | 61/18639 | 0.093730861 | 0.154380242 | 0.11607537 | CD8A | 1 |
| MF | GO:0016765 | transferase activity, transferring alkyl or aryl (other than methyl) groups | 1/30 | 61/18639 | 0.093730861 | 0.154380242 | 0.11607537 | MGST1 | 1 |
| MF | GO:0016836 | hydro-lyase activity | 1/30 | 61/18639 | 0.093730861 | 0.154380242 | 0.11607537 | TBXAS1 | 1 |
| MF | GO:0005507 | copper ion binding | 1/30 | 63/18639 | 0.096655487 | 0.157346142 | 0.11830537 | S100A12 | 1 |
| MF | GO:0016500 | protein-hormone receptor activity | 1/30 | 67/18639 | 0.102477394 | 0.164906151 | 0.123989587 | EPHA4 | 1 |
| MF | GO:0032934 | sterol binding | 1/30 | 68/18639 | 0.103927194 | 0.165338717 | 0.124314825 | RORA | 1 |
| MF | GO:0019199 | transmembrane receptor protein kinase activity | 1/30 | 78/18639 | 0.118301233 | 0.186091827 | 0.139918667 | EPHA4 | 1 |
| MF | GO:0016835 | carbon-oxygen lyase activity | 1/30 | 79/18639 | 0.119726316 | 0.186240936 | 0.140030779 | TBXAS1 | 1 |
| MF | GO:0048306 | calcium-dependent protein binding | 1/30 | 80/18639 | 0.121149172 | 0.186383341 | 0.140137851 | S100A12 | 1 |
| MF | GO:0008135 | translation factor activity, RNA binding | 1/30 | 83/18639 | 0.125404414 | 0.190832804 | 0.143483311 | EIF5A2 | 1 |
| MF | GO:0180051 | translation factor activity | 1/30 | 84/18639 | 0.126818397 | 0.190909414 | 0.143540913 | EIF5A2 | 1 |
| MF | GO:0016209 | antioxidant activity | 1/30 | 86/18639 | 0.129639736 | 0.193080458 | 0.145173277 | MGST1 | 1 |
| MF | GO:0052689 | carboxylic ester hydrolase activity | 1/30 | 92/18639 | 0.138050982 | 0.203443552 | 0.152965077 | PNPLA2 | 1 |
| MF | GO:0016407 | acetyltransferase activity | 1/30 | 96/18639 | 0.143614767 | 0.209438202 | 0.157472332 | CRAT | 1 |
| MF | GO:0004497 | monooxygenase activity | 1/30 | 106/18639 | 0.157372769 | 0.223963157 | 0.168393351 | TBXAS1 | 1 |
| MF | GO:0008013 | beta-catenin binding | 1/30 | 106/18639 | 0.157372769 | 0.223963157 | 0.168393351 | RORA | 1 |
| MF | GO:0004843 | cysteine-type deubiquitinase activity | 1/30 | 108/18639 | 0.160098614 | 0.223963157 | 0.168393351 | OTUD3 | 1 |
| MF | GO:0005496 | steroid binding | 1/30 | 108/18639 | 0.160098614 | 0.223963157 | 0.168393351 | RORA | 1 |
| MF | GO:0043022 | ribosome binding | 1/30 | 112/18639 | 0.165524754 | 0.223963157 | 0.168393351 | EIF5A2 | 1 |
| MF | GO:0001221 | transcription coregulator binding | 1/30 | 115/18639 | 0.169572105 | 0.223963157 | 0.168393351 | RORA | 1 |
| MF | GO:0017124 | SH3 domain binding | 1/30 | 115/18639 | 0.169572105 | 0.223963157 | 0.168393351 | FUT8 | 1 |
| MF | GO:0035401 | histone H3Y41 kinase activity | 1/30 | 115/18639 | 0.169572105 | 0.223963157 | 0.168393351 | EPHA4 | 1 |
| MF | GO:0101005 | deubiquitinase activity | 1/30 | 115/18639 | 0.169572105 | 0.223963157 | 0.168393351 | OTUD3 | 1 |
| MF | GO:0140801 | histone H2AXY142 kinase activity | 1/30 | 115/18639 | 0.169572105 | 0.223963157 | 0.168393351 | EPHA4 | 1 |
| MF | GO:1990782 | protein tyrosine kinase binding | 1/30 | 117/18639 | 0.172259789 | 0.22538664 | 0.169463639 | EPHA4 | 1 |
| MF | GO:0019783 | ubiquitin-like protein peptidase activity | 1/30 | 127/18639 | 0.185572575 | 0.240557041 | 0.180869956 | OTUD3 | 1 |
| MF | GO:0016798 | hydrolase activity, acting on glycosyl bonds | 1/30 | 130/18639 | 0.189525886 | 0.243427743 | 0.183028378 | TLR2 | 1 |
| MF | GO:0005179 | hormone activity | 1/30 | 132/18639 | 0.192151114 | 0.244555963 | 0.183876664 | RETN | 1 |
| MF | GO:0004713 | protein tyrosine kinase activity | 1/30 | 140/18639 | 0.202570066 | 0.255107892 | 0.191810445 | EPHA4 | 1 |
| MF | GO:0020037 | heme binding | 1/30 | 142/18639 | 0.205154438 | 0.255107892 | 0.191810445 | TBXAS1 | 1 |
| MF | GO:0019903 | protein phosphatase binding | 1/30 | 144/18639 | 0.207730712 | 0.255107892 | 0.191810445 | PDLIM4 | 1 |
| MF | GO:0019955 | cytokine binding | 1/30 | 144/18639 | 0.207730712 | 0.255107892 | 0.191810445 | IL1R2 | 1 |
| MF | GO:0046906 | tetrapyrrole binding | 1/30 | 152/18639 | 0.217955325 | 0.265336918 | 0.199501442 | TBXAS1 | 1 |
| MF | GO:0005506 | iron ion binding | 1/30 | 154/18639 | 0.220491479 | 0.266110406 | 0.200083012 | TBXAS1 | 1 |
| MF | GO:0003823 | antigen binding | 1/30 | 174/18639 | 0.245419639 | 0.293664525 | 0.220800395 | IL7R | 1 |
| MF | GO:0043021 | ribonucleoprotein complex binding | 1/30 | 177/18639 | 0.249091755 | 0.295532591 | 0.222204956 | EIF5A2 | 1 |
| MF | GO:0016853 | isomerase activity | 1/30 | 180/18639 | 0.252746594 | 0.296287889 | 0.222772849 | TBXAS1 | 1 |
| MF | GO:0016705 | oxidoreductase activity, acting on paired donors, with incorporation or reduction of molecular oxygen | 1/30 | 181/18639 | 0.253961048 | 0.296287889 | 0.222772849 | TBXAS1 | 1 |
| MF | GO:0019902 | phosphatase binding | 1/30 | 190/18639 | 0.264805615 | 0.306386662 | 0.230365911 | PDLIM4 | 1 |
| MF | GO:0016758 | hexosyltransferase activity | 1/30 | 197/18639 | 0.273134769 | 0.313433341 | 0.235664166 | FUT8 | 1 |
| MF | GO:0016829 | lyase activity | 1/30 | 200/18639 | 0.276676415 | 0.314916245 | 0.236779132 | TBXAS1 | 1 |
| MF | GO:0005516 | calmodulin binding | 1/30 | 207/18639 | 0.284875506 | 0.321633636 | 0.241829801 | IQCB1 | 1 |
| MF | GO:0005539 | glycosaminoglycan binding | 1/30 | 242/18639 | 0.324541717 | 0.363486723 | 0.273298288 | TLR2 | 1 |
| MF | GO:0140104 | molecular carrier activity | 1/30 | 249/18639 | 0.332215637 | 0.369128486 | 0.277540215 | XPO1 | 1 |
| MF | GO:0016757 | glycosyltransferase activity | 1/30 | 267/18639 | 0.351563587 | 0.385680785 | 0.289985552 | FUT8 | 1 |
| MF | GO:0001227 | DNA-binding transcription repressor activity, RNA polymerase II-specific | 1/30 | 268/18639 | 0.352622432 | 0.385680785 | 0.289985552 | KLF12 | 1 |
| MF | GO:0001217 | DNA-binding transcription repressor activity | 1/30 | 278/18639 | 0.363119366 | 0.393291143 | 0.295707626 | KLF12 | 1 |
| MF | GO:0030246 | carbohydrate binding | 1/30 | 280/18639 | 0.365198918 | 0.393291143 | 0.295707626 | KLRC3 | 1 |
| MF | GO:0031267 | small GTPase binding | 1/30 | 283/18639 | 0.368305944 | 0.393609406 | 0.295946922 | XPO1 | 1 |
| MF | GO:0031625 | ubiquitin protein ligase binding | 1/30 | 302/18639 | 0.387645105 | 0.411138748 | 0.309126878 | PYHIN1 | 1 |
| MF | GO:0051020 | GTPase binding | 1/30 | 315/18639 | 0.400546058 | 0.42162743 | 0.317013105 | XPO1 | 1 |
| MF | GO:0044389 | ubiquitin-like protein ligase binding | 1/30 | 322/18639 | 0.407383492 | 0.425624544 | 0.320018454 | PYHIN1 | 1 |
| MF | GO:0141003 | histone H2AX kinase activity | 1/30 | 354/18639 | 0.437692011 | 0.452910773 | 0.340534416 | EPHA4 | 1 |
| MF | GO:0140996 | histone H3 kinase activity | 1/30 | 357/18639 | 0.440455341 | 0.452910773 | 0.340534416 | EPHA4 | 1 |
| MF | GO:0035173 | histone kinase activity | 1/30 | 360/18639 | 0.443205542 | 0.452910773 | 0.340534416 | EPHA4 | 1 |
| MF | GO:0003779 | actin binding | 1/30 | 443/18639 | 0.514317373 | 0.521771248 | 0.392309209 | PDLIM4 | 1 |
| MF | GO:0001228 | DNA-binding transcription activator activity, RNA polymerase II-specific | 1/30 | 484/18639 | 0.546122296 | 0.550051234 | 0.413572356 | MAFG | 1 |
| MF | GO:0001216 | DNA-binding transcription activator activity | 1/30 | 491/18639 | 0.551347232 | 0.551347232 | 0.414546791 | MAFG | 1 |
