# Supplementary material for: Integrated bioinformatics and machine learning for constructing a diagnostic model of major depressive disorder leveraging shared signatures from hemodialysis: A cross-sectional study
Source: Medicine (Baltimore). 2026 Jun 5;105(23):e49113. doi: 10.1097/MD.0000000000049113 (PMC13246050; doi:10.1097/MD.0000000000049113)
Supplement: Supplementary file 4 [file medi-105-e49113-s004.docx]

# ****Supplementary Table 4. KEGG function enrichment analysis****

| **ID** | **Description** | **GeneRatio** | **BgRatio** | **pvalue** | **p.adjust** | **qvalue** | **geneID** | **Count** |
| --- | --- | --- | --- | --- | --- | --- | --- | --- |
| hsa04640 | Hematopoietic cell lineage | 3/19 | 100/9564 | 0.000951700486415225 | 0.0704258359947267 | 0.0661181390562156 | CD8A/IL1R2/IL7R | 3 |
| hsa05340 | Primary immunodeficiency | 2/19 | 38/9564 | 0.00251900392283349 | 0.093203145144839 | 0.0875022415300053 | CD8A/IL7R | 2 |
| hsa05321 | Inflammatory bowel disease | 2/19 | 66/9564 | 0.00743585163863565 | 0.183417673753013 | 0.172198669526299 | TLR2/RORA | 2 |
| hsa04612 | Antigen processing and presentation | 2/19 | 82/9564 | 0.0112972915425226 | 0.208999893536667 | 0.196216116264866 | KLRC3/CD8A | 2 |
| hsa05146 | Amoebiasis | 2/19 | 103/9564 | 0.0174328468077021 | 0.258006132753992 | 0.242224818801756 | TLR2/IL1R2 | 2 |
| hsa00533 | Glycosaminoglycan biosynthesis - keratan sulfate | 1/19 | 14/9564 | 0.0274747615531495 | 0.335478819174309 | 0.314958777603192 | FUT8 | 1 |
| hsa05418 | Fluid shear stress and atherosclerosis | 2/19 | 142/9564 | 0.031734482894867 | 0.335478819174309 | 0.314958777603192 | MGST1/IL1R2 | 2 |
| hsa05152 | Tuberculosis | 2/19 | 182/9564 | 0.0498221516309805 | 0.35262998905531 | 0.331060871659323 | TLR2/CTSD | 2 |
| hsa05202 | Transcriptional misregulation in cancer | 2/19 | 201/9564 | 0.0594642269938029 | 0.35262998905531 | 0.331060871659323 | FUT8/IL1R2 | 2 |
| hsa04710 | Circadian rhythm | 1/19 | 34/9564 | 0.0654864736065021 | 0.35262998905531 | 0.331060871659323 | RORA | 1 |
| hsa05166 | Human T-cell leukemia virus 1 infection | 2/19 | 224/9564 | 0.0719334868517573 | 0.35262998905531 | 0.331060871659323 | XPO1/IL1R2 | 2 |
| hsa04820 | Cytoskeleton in muscle cells | 2/19 | 233/9564 | 0.0770316725311965 | 0.35262998905531 | 0.331060871659323 | PDLIM4/TNNT1 | 2 |
| hsa00513 | Various types of N-glycan biosynthesis | 1/19 | 44/9564 | 0.0839602853081992 | 0.35262998905531 | 0.331060871659323 | FUT8 | 1 |
| hsa04142 | Lysosome | 2/19 | 249/9564 | 0.0863757212392988 | 0.35262998905531 | 0.331060871659323 | SORT1/CTSD | 2 |
| hsa05144 | Malaria | 1/19 | 50/9564 | 0.0948779352616842 | 0.35262998905531 | 0.331060871659323 | TLR2 | 1 |
| hsa04979 | Cholesterol metabolism | 1/19 | 52/9564 | 0.0984896757067086 | 0.35262998905531 | 0.331060871659323 | SORT1 | 1 |
| hsa00510 | N-Glycan biosynthesis | 1/19 | 56/9564 | 0.105672236190598 | 0.35262998905531 | 0.331060871659323 | FUT8 | 1 |
| hsa05134 | Legionellosis | 1/19 | 56/9564 | 0.105672236190598 | 0.35262998905531 | 0.331060871659323 | TLR2 | 1 |
| hsa00480 | Glutathione metabolism | 1/19 | 59/9564 | 0.111023542857687 | 0.35262998905531 | 0.331060871659323 | MGST1 | 1 |
| hsa04923 | Regulation of lipolysis in adipocytes | 1/19 | 59/9564 | 0.111023542857687 | 0.35262998905531 | 0.331060871659323 | PNPLA2 | 1 |
| hsa04060 | Cytokine-cytokine receptor interaction | 2/19 | 298/9564 | 0.116957496239591 | 0.35262998905531 | 0.331060871659323 | IL1R2/IL7R | 2 |
| hsa00590 | Arachidonic acid metabolism | 1/19 | 63/9564 | 0.118111444115367 | 0.35262998905531 | 0.331060871659323 | TBXAS1 | 1 |
| hsa00561 | Glycerolipid metabolism | 1/19 | 65/9564 | 0.121635285514757 | 0.35262998905531 | 0.331060871659323 | PNPLA2 | 1 |
| hsa05204 | Chemical carcinogenesis - DNA adducts | 1/19 | 71/9564 | 0.132126946899931 | 0.35262998905531 | 0.331060871659323 | MGST1 | 1 |
| hsa00982 | Drug metabolism - cytochrome P450 | 1/19 | 73/9564 | 0.135597704959075 | 0.35262998905531 | 0.331060871659323 | MGST1 | 1 |
| hsa03250 | Viral life cycle - HIV-1 | 1/19 | 73/9564 | 0.135597704959075 | 0.35262998905531 | 0.331060871659323 | XPO1 | 1 |
| hsa01524 | Platinum drug resistance | 1/19 | 75/9564 | 0.139055310684301 | 0.35262998905531 | 0.331060871659323 | MGST1 | 1 |
| hsa00980 | Metabolism of xenobiotics by cytochrome P450 | 1/19 | 79/9564 | 0.145931253299864 | 0.35262998905531 | 0.331060871659323 | MGST1 | 1 |
| hsa05140 | Leishmaniasis | 1/19 | 79/9564 | 0.145931253299864 | 0.35262998905531 | 0.331060871659323 | TLR2 | 1 |
| hsa00983 | Drug metabolism - other enzymes | 1/19 | 81/9564 | 0.149349683877494 | 0.35262998905531 | 0.331060871659323 | MGST1 | 1 |
| hsa04146 | Peroxisome | 1/19 | 83/9564 | 0.152755149496794 | 0.35262998905531 | 0.331060871659323 | CRAT | 1 |
| hsa04151 | PI3K-Akt signaling pathway | 2/19 | 362/9564 | 0.160399973858694 | 0.35262998905531 | 0.331060871659323 | TLR2/IL7R | 2 |
| hsa05235 | PD-L1 expression and PD-1 checkpoint pathway in cancer | 1/19 | 90/9564 | 0.164572849323581 | 0.35262998905531 | 0.331060871659323 | TLR2 | 1 |
| hsa05323 | Rheumatoid arthritis | 1/19 | 95/9564 | 0.172918269335523 | 0.35262998905531 | 0.331060871659323 | TLR2 | 1 |
| hsa05142 | Chagas disease | 1/19 | 103/9564 | 0.18610687477238 | 0.35262998905531 | 0.331060871659323 | TLR2 | 1 |
| hsa05215 | Prostate cancer | 1/19 | 106/9564 | 0.191001039119712 | 0.35262998905531 | 0.331060871659323 | IL1R2 | 1 |
| hsa03013 | Nucleocytoplasmic transport | 1/19 | 108/9564 | 0.194248311706645 | 0.35262998905531 | 0.331060871659323 | XPO1 | 1 |
| hsa04620 | Toll-like receptor signaling pathway | 1/19 | 109/9564 | 0.195867313618402 | 0.35262998905531 | 0.331060871659323 | TLR2 | 1 |
| hsa04659 | Th17 cell differentiation | 1/19 | 109/9564 | 0.195867313618402 | 0.35262998905531 | 0.331060871659323 | RORA | 1 |
| hsa05145 | Toxoplasmosis | 1/19 | 112/9564 | 0.200705848419289 | 0.35262998905531 | 0.331060871659323 | TLR2 | 1 |
| hsa03082 | ATP-dependent chromatin remodeling | 1/19 | 117/9564 | 0.208708834721576 | 0.35262998905531 | 0.331060871659323 | BCL7A | 1 |
| hsa04722 | Neurotrophin signaling pathway | 1/19 | 120/9564 | 0.213474125289407 | 0.35262998905531 | 0.331060871659323 | SORT1 | 1 |
| hsa04660 | T cell receptor signaling pathway | 1/19 | 122/9564 | 0.216635867918847 | 0.35262998905531 | 0.331060871659323 | CD8A | 1 |
| hsa04071 | Sphingolipid signaling pathway | 1/19 | 125/9564 | 0.221355913946262 | 0.35262998905531 | 0.331060871659323 | CTSD | 1 |
| hsa04611 | Platelet activation | 1/19 | 126/9564 | 0.222923266169487 | 0.35262998905531 | 0.331060871659323 | TBXAS1 | 1 |
| hsa04068 | FoxO signaling pathway | 1/19 | 133/9564 | 0.233811334243407 | 0.35262998905531 | 0.331060871659323 | IL7R | 1 |
| hsa04650 | Natural killer cell mediated cytotoxicity | 1/19 | 134/9564 | 0.23535492290308 | 0.35262998905531 | 0.331060871659323 | KLRC3 | 1 |
| hsa05162 | Measles | 1/19 | 136/9564 | 0.238433266320683 | 0.35262998905531 | 0.331060871659323 | TLR2 | 1 |
| hsa04210 | Apoptosis | 1/19 | 137/9564 | 0.239968031694029 | 0.35262998905531 | 0.331060871659323 | CTSD | 1 |
| hsa05135 | Yersinia infection | 1/19 | 138/9564 | 0.241499866572338 | 0.35262998905531 | 0.331060871659323 | CD8A | 1 |
| hsa04915 | Estrogen signaling pathway | 1/19 | 139/9564 | 0.243028776240821 | 0.35262998905531 | 0.331060871659323 | CTSD | 1 |
| hsa05017 | Spinocerebellar ataxia | 1/19 | 144/9564 | 0.250629630791054 | 0.356665243818039 | 0.334849304295741 | RORA | 1 |
| hsa04145 | Phagosome | 1/19 | 159/9564 | 0.273000735804119 | 0.367878275946812 | 0.345376475284347 | TLR2 | 1 |
| hsa04514 | Cell adhesion molecule (CAM) interaction | 1/19 | 160/9564 | 0.274469421186333 | 0.367878275946812 | 0.345376475284347 | CD8A | 1 |
| hsa05161 | Hepatitis B | 1/19 | 163/9564 | 0.278858630573931 | 0.367878275946812 | 0.345376475284347 | TLR2 | 1 |
| hsa04630 | JAK-STAT signaling pathway | 1/19 | 168/9564 | 0.286118127599243 | 0.367878275946812 | 0.345376475284347 | IL7R | 1 |
| hsa04140 | Autophagy - animal | 1/19 | 169/9564 | 0.287561694603885 | 0.367878275946812 | 0.345376475284347 | CTSD | 1 |
| hsa05225 | Hepatocellular carcinoma | 1/19 | 170/9564 | 0.289002495860141 | 0.367878275946812 | 0.345376475284347 | MGST1 | 1 |
| hsa05164 | Influenza A | 1/19 | 173/9564 | 0.293308355146782 | 0.367878275946812 | 0.345376475284347 | XPO1 | 1 |
| hsa05168 | Herpes simplex virus 1 infection | 1/19 | 180/9564 | 0.30325948916969 | 0.373901727834361 | 0.351031494126142 | TLR2 | 1 |
| hsa04360 | Axon guidance | 1/19 | 184/9564 | 0.308886097254751 | 0.373901727834361 | 0.351031494126142 | EPHA4 | 1 |
| hsa04613 | Neutrophil extracellular trap formation | 1/19 | 196/9564 | 0.325508774250103 | 0.373901727834361 | 0.351031494126142 | TLR2 | 1 |
| hsa04814 | Motor proteins | 1/19 | 197/9564 | 0.32687676456706 | 0.373901727834361 | 0.351031494126142 | TNNT1 | 1 |
| hsa05205 | Proteoglycans in cancer | 1/19 | 204/9564 | 0.336379357446811 | 0.373901727834361 | 0.351031494126142 | TLR2 | 1 |
| hsa05169 | Epstein-Barr virus infection | 1/19 | 205/9564 | 0.337726450631481 | 0.373901727834361 | 0.351031494126142 | TLR2 | 1 |
| hsa05415 | Diabetic cardiomyopathy | 1/19 | 205/9564 | 0.337726450631481 | 0.373901727834361 | 0.351031494126142 | CTSD | 1 |
| hsa05170 | Human immunodeficiency virus 1 infection | 1/19 | 214/9564 | 0.349734264570028 | 0.373901727834361 | 0.351031494126142 | TLR2 | 1 |
| hsa05417 | Lipid and atherosclerosis | 1/19 | 216/9564 | 0.352374511658925 | 0.373901727834361 | 0.351031494126142 | TLR2 | 1 |
| hsa04382 | Cornified envelope formation | 1/19 | 217/9564 | 0.353690823627098 | 0.373901727834361 | 0.351031494126142 | S100A12 | 1 |
| hsa05207 | Chemical carcinogenesis - receptor activation | 1/19 | 217/9564 | 0.353690823627098 | 0.373901727834361 | 0.351031494126142 | MGST1 | 1 |
| hsa05208 | Chemical carcinogenesis - reactive oxygen species | 1/19 | 227/9564 | 0.366715282560181 | 0.382210294499344 | 0.358831855433239 | MGST1 | 1 |
| hsa04714 | Thermogenesis | 1/19 | 235/9564 | 0.376955410364202 | 0.38597067869943 | 0.362362230357929 | PNPLA2 | 1 |
| hsa05171 | Coronavirus disease - COVID-19 | 1/19 | 238/9564 | 0.380754858717005 | 0.38597067869943 | 0.362362230357929 | TLR2 | 1 |
| hsa05132 | Salmonella infection | 1/19 | 251/9564 | 0.396966939912957 | 0.396966939912957 | 0.372685889534212 | TLR2 | 1 |
