# Supplementary material for: Integrated bioinformatics and machine learning for constructing a diagnostic model of major depressive disorder leveraging shared signatures from hemodialysis: A cross-sectional study
Source: Medicine (Baltimore). 2026 Jun 5;105(23):e49113. doi: 10.1097/MD.0000000000049113 (PMC13246050; doi:10.1097/MD.0000000000049113)
Supplement: Supplementary file 5 [file medi-105-e49113-s005.docx]

**Supplementary Table 5. Model and gene ID**

| **No** | **Model** | **Gene ID** | **Train (AUC)** | **GSE52790 (AUC)** | **GSE76826(AUC)** |
| --- | --- | --- | --- | --- | --- |
| 1 | Lasso+Stepglm[both] | MGST1,BCL7A,CRAT,FUT8,MAFG | 0.756835938 | 0.558333333333333 | 0.866666666666667 |
| 2 | SVM | IL1R2,MGST1,BCL7A,CRAT,FUT8,EPHA4 | 0.86328125 | 0.558333333333333 | 0.883333333333333 |
| 3 | glmBoost+SVM | MGST1,BCL7A,CRAT,FUT8,MAFG,SDAD1 | 0.851074219 | 0.533333333333333 | 0.875 |
| 4 | Ridge | IL1R2,MGST1,BCL7A,CRAT,FUT8,EPHA4 | 0.739257813 | 0.533333333333333 | 0.875 |
| 5 | Lasso+SVM | MGST1,BCL7A,CRAT,FUT8,MAFG,SDAD1 | 0.851074219 | 0.533333333333333 | 0.875 |
| 6 | glmBoost+Ridge | MGST1,BCL7A,CRAT,FUT8,MAFG,SDAD1 | 0.752685547 | 0.666666666666667 | 0.891666666666667 |
| 7 | Enet[alpha=0.1] | IL1R2,MGST1,BCL7A,CRAT,FUT8,EPHA4 | 0.737792969 | 0.516666666666667 | 0.875 |
| 8 | glmBoost+Enet[alpha=0.1] | MGST1,BCL7A,CRAT,FUT8,MAFG,SDAD1 | 0.754638672 | 0.666666666666667 | 0.891666666666667 |
| 9 | Enet[alpha=0.2] | IL1R2,MGST1,BCL7A,CRAT,FUT8,EPHA4 | 0.738769531 | 0.491666666666667 | 0.875 |
| 10 | Enet[alpha=0.3] | IL1R2,MGST1,BCL7A,CRAT,FUT8,EPHA4 | 0.739990234 | 0.475 | 0.875 |
| 11 | glmBoost+Enet[alpha=0.3] | MGST1,BCL7A,CRAT,FUT8,MAFG,SDAD1 | 0.755126953 | 0.65 | 0.891666666666667 |
| 12 | glmBoost+Enet[alpha=0.2] | MGST1,BCL7A,CRAT,FUT8,MAFG,SDAD1 | 0.755371094 | 0.658333333333333 | 0.891666666666667 |
| 13 | Enet[alpha=0.4] | IL1R2,MGST1,BCL7A,CRAT,FUT8,EPHA4 | 0.738769531 | 0.5 | 0.875 |
| 14 | glmBoost+Enet[alpha=0.4] | MGST1,BCL7A,CRAT,FUT8,MAFG,SDAD1 | 0.754882813 | 0.65 | 0.891666666666667 |
| 15 | Lasso+glmBoost | MGST1,BCL7A,CRAT,FUT8,MAFG,SDAD1 | 0.754638672 | 0.65 | 0.891666666666667 |
| 16 | Enet[alpha=0.5] | IL1R2,MGST1,BCL7A,CRAT,FUT8,EPHA4 | 0.738037109 | 0.466666666666667 | 0.866666666666667 |
| 17 | glmBoost | IL1R2,MGST1,BCL7A,CRAT,FUT8,EPHA4 | 0.741210938 | 0.525 | 0.883333333333333 |
| 18 | glmBoost+Enet[alpha=0.5] | MGST1,BCL7A,CRAT,FUT8,MAFG,SDAD1 | 0.756347656 | 0.641666666666667 | 0.891666666666667 |
| 19 | Enet[alpha=0.6] | IL1R2,MGST1,BCL7A,CRAT,FUT8,EPHA4 | 0.740234375 | 0.525 | 0.883333333333333 |
| 20 | glmBoost+Enet[alpha=0.6] | MGST1,BCL7A,CRAT,FUT8,MAFG,SDAD1 | 0.756835938 | 0.641666666666667 | 0.891666666666667 |
| 21 | glmBoost+Enet[alpha=0.7] | MGST1,BCL7A,CRAT,FUT8,MAFG,SDAD1 | 0.755859375 | 0.65 | 0.891666666666667 |
| 22 | glmBoost+Enet[alpha=0.8] | MGST1,BCL7A,CRAT,FUT8,MAFG,SDAD1 | 0.756835938 | 0.641666666666667 | 0.891666666666667 |
| 23 | Enet[alpha=0.8] | IL1R2,MGST1,BCL7A,CRAT,FUT8,EPHA4 | 0.740722656 | 0.491666666666667 | 0.875 |
| 24 | Enet[alpha=0.9] | IL1R2,MGST1,BCL7A,CRAT,FUT8,EPHA4 | 0.738525391 | 0.466666666666667 | 0.866666666666667 |
| 25 | Lasso | MGST1,BCL7A,CRAT,FUT8 | 0.736328125 | 0.458333333333333 | 0.866666666666667 |
| 26 | Enet[alpha=0.7] | IL1R2,MGST1,BCL7A,CRAT,FUT8,EPHA4 | 0.739257813 | 0.466666666666667 | 0.866666666666667 |
| 27 | glmBoost+Enet[alpha=0.9] | MGST1,BCL7A,CRAT,FUT8,MAFG,SDAD1 | 0.756591797 | 0.641666666666667 | 0.891666666666667 |
| 28 | glmBoost+Lasso | MGST1,BCL7A,CRAT,FUT8,MAFG,SDAD1 | 0.755371094 | 0.65 | 0.891666666666667 |
| 29 | Lasso+plsRglm | MGST1,BCL7A,CRAT,FUT8,MAFG,SDAD1 | 0.75390625 | 0.658333333333333 | 0.891666666666667 |
| 30 | glmBoost+plsRglm | MGST1,BCL7A,CRAT,FUT8,MAFG,SDAD1 | 0.75390625 | 0.658333333333333 | 0.891666666666667 |
| 31 | glmBoost+Stepglm[forward] | MGST1,BCL7A,CRAT,FUT8,MAFG,SDAD1 | 0.755615234 | 0.633333333333333 | 0.891666666666667 |
| 32 | Lasso+Stepglm[forward] | MGST1,BCL7A,CRAT,FUT8,MAFG,SDAD1 | 0.755615234 | 0.633333333333333 | 0.891666666666667 |
| 33 | RF+SVM | MGST1,BCL7A,FUT8,SORT1,PDLIM4,S100A12 | 0.903564453 | 0.55 | 0.791666666666667 |
| 34 | Stepglm[forward] | IL1R2,MGST1,BCL7A,CRAT,FUT8,EPHA4 | 0.741455078 | 0.541666666666667 | 0.883333333333333 |
| 35 | plsRglm | IL1R2,MGST1,BCL7A,CRAT,FUT8,EPHA4 | 0.741455078 | 0.541666666666667 | 0.875 |
| 36 | RF+Ridge | MGST1,BCL7A,FUT8,SORT1,PDLIM4,S100A12 | 0.737792969 | 0.6 | 0.825 |
| 37 | RF+Enet[alpha=0.1] | MGST1,BCL7A,FUT8,SORT1,PDLIM4,S100A12 | 0.737060547 | 0.583333333333333 | 0.816666666666667 |
| 38 | RF+plsRglm | BCL7A,FUT8,SORT1,PDLIM4 | 0.716552734 | 0.666666666666667 | 0.766666666666667 |
| 39 | RF+Stepglm[forward] | MGST1,BCL7A,FUT8,SORT1,PDLIM4,S100A12 | 0.733398438 | 0.575 | 0.825 |
| 40 | RF+Enet[alpha=0.2] | MGST1,BCL7A,FUT8,SORT1,PDLIM4 | 0.734375 | 0.575 | 0.808333333333333 |
| 41 | RF+Enet[alpha=0.3] | MGST1,BCL7A,FUT8,SORT1,PDLIM4 | 0.733886719 | 0.575 | 0.808333333333333 |
| 42 | RF+Enet[alpha=0.6] | MGST1,BCL7A,FUT8,SORT1,PDLIM4 | 0.732910156 | 0.575 | 0.808333333333333 |
| 43 | RF+Lasso | MGST1,BCL7A,FUT8,SORT1,PDLIM4 | 0.732421875 | 0.575 | 0.816666666666667 |
| 44 | RF+Enet[alpha=0.7] | MGST1,BCL7A,FUT8,SORT1,PDLIM4 | 0.731933594 | 0.575 | 0.816666666666667 |
| 45 | RF+Enet[alpha=0.5] | MGST1,BCL7A,FUT8,SORT1,PDLIM4 | 0.732910156 | 0.583333333333333 | 0.808333333333333 |
| 46 | RF+glmBoost | MGST1,BCL7A,FUT8,SORT1,PDLIM4 | 0.733886719 | 0.558333333333333 | 0.825 |
| 47 | RF+Enet[alpha=0.9] | MGST1,BCL7A,FUT8,SORT1,PDLIM4 | 0.732177734 | 0.575 | 0.816666666666667 |
| 48 | RF+Enet[alpha=0.4] | MGST1,BCL7A,FUT8,SORT1,PDLIM4 | 0.733154297 | 0.575 | 0.808333333333333 |
| 49 | RF+Enet[alpha=0.8] | MGST1,BCL7A,FUT8,SORT1,PDLIM4 | 0.732177734 | 0.575 | 0.816666666666667 |
| 50 | RF+Stepglm[both] | MGST1,BCL7A,FUT8,SORT1 | 0.729492188 | 0.55 | 0.833333333333333 |
| 51 | RF+Stepglm[backward] | MGST1,BCL7A,FUT8,SORT1 | 0.729492188 | 0.55 | 0.833333333333333 |
| 52 | Stepglm[both]+Ridge | MGST1,BCL7A,CRAT,FUT8,MAFG,SORT1 | 0.761230469 | 0.516666666666667 | 0.866666666666667 |
| 53 | Stepglm[backward]+Ridge | MGST1,BCL7A,CRAT,FUT8,MAFG,SORT1 | 0.761230469 | 0.516666666666667 | 0.866666666666667 |
| 54 | Stepglm[both]+plsRglm | MGST1,BCL7A,CRAT,FUT8,MAFG,SORT1 | 0.760498047 | 0.508333333333333 | 0.858333333333333 |
| 55 | Stepglm[backward]+plsRglm | MGST1,BCL7A,CRAT,FUT8,MAFG,SORT1 | 0.760498047 | 0.508333333333333 | 0.858333333333333 |
| 56 | Stepglm[both]+Enet[alpha=0.9] | MGST1,BCL7A,CRAT,FUT8,MAFG,SORT1 | 0.763671875 | 0.508333333333333 | 0.866666666666667 |
| 57 | Stepglm[backward]+Enet[alpha=0.9] | MGST1,BCL7A,CRAT,FUT8,MAFG,SORT1 | 0.761962891 | 0.508333333333333 | 0.866666666666667 |
| 58 | Stepglm[both]+Enet[alpha=0.1] | MGST1,BCL7A,CRAT,FUT8,MAFG,SORT1 | 0.761474609 | 0.508333333333333 | 0.866666666666667 |
| 59 | Stepglm[backward]+Enet[alpha=0.1] | MGST1,BCL7A,CRAT,FUT8,MAFG,SORT1 | 0.76171875 | 0.508333333333333 | 0.866666666666667 |
| 60 | Stepglm[both]+Enet[alpha=0.8] | MGST1,BCL7A,CRAT,FUT8,MAFG,SORT1 | 0.763183594 | 0.508333333333333 | 0.866666666666667 |
| 61 | Stepglm[backward]+Enet[alpha=0.8] | MGST1,BCL7A,CRAT,FUT8,MAFG,SORT1 | 0.763671875 | 0.5 | 0.866666666666667 |
| 62 | Stepglm[both]+Enet[alpha=0.2] | MGST1,BCL7A,CRAT,FUT8,MAFG,SORT1 | 0.761230469 | 0.5 | 0.866666666666667 |
| 63 | Stepglm[backward]+Enet[alpha=0.2] | MGST1,BCL7A,CRAT,FUT8,MAFG,SORT1 | 0.761474609 | 0.508333333333333 | 0.866666666666667 |
| 64 | Stepglm[both]+Lasso | MGST1,BCL7A,CRAT,FUT8,MAFG,SORT1 | 0.764160156 | 0.508333333333333 | 0.866666666666667 |
| 65 | Stepglm[backward]+Lasso | MGST1,BCL7A,CRAT,FUT8,MAFG,SORT1 | 0.763671875 | 0.508333333333333 | 0.866666666666667 |
| 66 | Stepglm[both]+Enet[alpha=0.6] | MGST1,BCL7A,CRAT,FUT8,MAFG,SORT1 | 0.761962891 | 0.508333333333333 | 0.866666666666667 |
| 67 | Stepglm[backward]+Enet[alpha=0.6] | MGST1,BCL7A,CRAT,FUT8,MAFG,SORT1 | 0.763183594 | 0.5 | 0.866666666666667 |
| 68 | glmBoost+GBM | BCL7A,FUT8,CRAT,MGST1,SDAD1,MAFG | 0.885498047 | 0.841666666666667 | 0.85 |
| 69 | Stepglm[both]+Enet[alpha=0.7] | MGST1,BCL7A,CRAT,FUT8,MAFG,SORT1 | 0.762207031 | 0.508333333333333 | 0.866666666666667 |
| 70 | Stepglm[backward]+Enet[alpha=0.7] | MGST1,BCL7A,CRAT,FUT8,MAFG,SORT1 | 0.763183594 | 0.508333333333333 | 0.866666666666667 |
| 71 | Lasso+Stepglm[backward] | MGST1,BCL7A,CRAT,FUT8,MAFG | 0.756835938 | 0.558333333333333 | 0.866666666666667 |
| 72 | Stepglm[both] | MGST1,BCL7A,CRAT,FUT8 | 0.739746094 | 0.483333333333333 | 0.866666666666667 |
| 73 | Stepglm[backward] | MGST1,BCL7A,CRAT,FUT8 | 0.739746094 | 0.483333333333333 | 0.866666666666667 |
| 74 | glmBoost+Stepglm[both] | MGST1,BCL7A,CRAT,FUT8,MAFG | 0.756835938 | 0.558333333333333 | 0.866666666666667 |
| 75 | glmBoost+Stepglm[backward] | MGST1,BCL7A,CRAT,FUT8,MAFG | 0.756835938 | 0.558333333333333 | 0.866666666666667 |
| 76 | Stepglm[both]+Enet[alpha=0.4] | MGST1,BCL7A,CRAT,FUT8,MAFG,SORT1 | 0.76171875 | 0.516666666666667 | 0.866666666666667 |
| 77 | Stepglm[backward]+Enet[alpha=0.4] | MGST1,BCL7A,CRAT,FUT8,MAFG,SORT1 | 0.762451172 | 0.508333333333333 | 0.866666666666667 |
| 78 | Stepglm[both]+Enet[alpha=0.3] | MGST1,BCL7A,CRAT,FUT8,MAFG,SORT1 | 0.761962891 | 0.508333333333333 | 0.866666666666667 |
| 79 | Stepglm[backward]+Enet[alpha=0.3] | MGST1,BCL7A,CRAT,FUT8,MAFG,SORT1 | 0.762207031 | 0.5 | 0.866666666666667 |
| 80 | Stepglm[both]+glmBoost | MGST1,BCL7A,CRAT,FUT8,MAFG,SORT1 | 0.761474609 | 0.516666666666667 | 0.866666666666667 |
| 81 | Stepglm[backward]+glmBoost | MGST1,BCL7A,CRAT,FUT8,MAFG,SORT1 | 0.761474609 | 0.516666666666667 | 0.866666666666667 |
| 82 | Stepglm[both]+Enet[alpha=0.5] | MGST1,BCL7A,CRAT,FUT8,MAFG,SORT1 | 0.762451172 | 0.508333333333333 | 0.866666666666667 |
| 83 | Stepglm[backward]+Enet[alpha=0.5] | MGST1,BCL7A,CRAT,FUT8,MAFG,SORT1 | 0.762207031 | 0.508333333333333 | 0.866666666666667 |
| 84 | glmBoost+RF | MGST1,BCL7A,CRAT,FUT8 | 0.996337891 | 0.708333333333333 | 0.758333333333333 |
| 85 | RF | IL1R2,MGST1,BCL7A,CRAT,FUT8 | 0.997314453 | 0.616666666666667 | 0.616666666666667 |
| 86 | Lasso+GBM | BCL7A,FUT8,CRAT,MGST1,SDAD1,MAFG | 0.890625 | 0.858333333333333 | 0.858333333333333 |
| 87 | RF+GBM | BCL7A,FUT8,SORT1,MGST1,PDLIM4,S100A12 | 0.891357422 | 0.816666666666667 | 0.658333333333333 |
| 88 | GBM | BCL7A,FUT8,CRAT,MGST1,EPHA4,IL1R2 | 0.911865234 | 0.633333333333333 | 0.708333333333333 |
| 89 | Stepglm[both]+SVM | MGST1,BCL7A,CRAT,FUT8,MAFG,SORT1 | 0.873046875 | 0.491666666666667 | 0.841666666666667 |
| 90 | Stepglm[backward]+SVM | MGST1,BCL7A,CRAT,FUT8,MAFG,SORT1 | 0.873046875 | 0.491666666666667 | 0.841666666666667 |
| 91 | Lasso+RF | MGST1,BCL7A,CRAT,FUT8 | 0.997070313 | 0.758333333333333 | 0.766666666666667 |
| 92 | Stepglm[both]+GBM | BCL7A,FUT8,SORT1,CRAT,MGST1,MAFG | 0.905029297 | 0.75 | 0.708333333333333 |
| 93 | Stepglm[backward]+GBM | BCL7A,FUT8,SORT1,CRAT,MGST1,MAFG | 0.911865234 | 0.75 | 0.7 |
| 94 | Stepglm[both]+RF | MGST1,BCL7A,CRAT,FUT8,SORT1 | 0.996826172 | 0.708333333333333 | 0.633333333333333 |
| 95 | LDA | IL1R2,MGST1,BCL7A,CRAT,FUT8,EPHA4 | 0.740722656 | 0.541666666666667 | 0.875 |
| 96 | glmBoost+LDA | MGST1,BCL7A,CRAT,FUT8,MAFG,SDAD1 | 0.757080078 | 0.641666666666667 | 0.891666666666667 |
| 97 | RF+LDA | MGST1,BCL7A,FUT8,SORT1,PDLIM4,S100A12 | 0.733398438 | 0.558333333333333 | 0.825 |
| 98 | Stepglm[both]+LDA | MGST1,BCL7A,CRAT,FUT8,MAFG,SORT1 | 0.761230469 | 0.508333333333333 | 0.858333333333333 |
| 99 | Stepglm[backward]+LDA | MGST1,BCL7A,CRAT,FUT8,MAFG,SORT1 | 0.761230469 | 0.508333333333333 | 0.858333333333333 |
| 100 | Lasso+LDA | MGST1,BCL7A,CRAT,FUT8,MAFG,SDAD1 | 0.757080078 | 0.641666666666667 | 0.891666666666667 |
| 101 | Stepglm[backward]+RF | MGST1,BCL7A,CRAT,FUT8,SORT1 | 0.996826172 | 0.675 | 0.633333333333333 |
| 102 | XGBoost | IL1R2,MGST1,BCL7A,CRAT,FUT8,EPHA4 | 0.9388427734375 | 0.708333333333333 | 0.55 |
| 103 | Lasso+XGBoost | MGST1,BCL7A,CRAT,FUT8,MAFG,SDAD1 | 0.9193115234375 | 0.8125 | 0.504166666666667 |
| 104 | glmBoost+XGBoost | MGST1,BCL7A,CRAT,FUT8,MAFG,SDAD1 | 0.9193115234375 | 0.8125 | 0.504166666666667 |
| 105 | RF+XGBoost | MGST1,BCL7A,FUT8,SORT1,PDLIM4,S100A12 | 0.930908203 | 0.8375 | 0.4875 |
| 106 | Stepglm[both]+XGBoost | MGST1,BCL7A,CRAT,FUT8,MAFG,SORT1 | 0.956787109 | 0.55 | 0.533333333333333 |
| 107 | Stepglm[backward]+XGBoost | MGST1,BCL7A,CRAT,FUT8,MAFG,SORT1 | 0.956787109 | 0.55 | 0.533333333333333 |
| 108 | NaiveBayes | IL1R2,MGST1,BCL7A,CRAT,FUT8,EPHA4 | 0.74609375 | 0.475 | 0.883333333333333 |
| 109 | Lasso+NaiveBayes | MGST1,BCL7A,CRAT,FUT8,MAFG,SDAD1 | 0.754638672 | 0.725 | 0.883333333333333 |
| 110 | glmBoost+NaiveBayes | MGST1,BCL7A,CRAT,FUT8,MAFG,SDAD1 | 0.754638672 | 0.725 | 0.883333333333333 |
| 111 | RF+NaiveBayes | MGST1,BCL7A,FUT8,SORT1,PDLIM4,S100A12 | 0.784667969 | 0.675 | 0.808333333333333 |
| 112 | Stepglm[both]+NaiveBayes | MGST1,BCL7A,CRAT,FUT8,MAFG,SORT1 | 0.773681641 | 0.541666666666667 | 0.858333333333333 |
| 113 | Stepglm[backward]+NaiveBayes | MGST1,BCL7A,CRAT,FUT8,MAFG,SORT1 | 0.773681641 | 0.541666666666667 | 0.858333333333333 |
| 114 | Stepglm[both]+RF+NaiveBayes | MGST1,BCL7A,CRAT,FUT8,SORT1 | 0.997070313 | 0.666666666666667 | 0.641666666666667 |
| 115 | Lasso+GBM+RF | BCL7A,FUT8,CRAT,MGST1,SDAD1,MAFG | 0.891357422 | 0.841666666666667 | 0.841666666666667 |
| 116 | Stepglm[both]+Enet[alpha=0.8]+XGBoost | MGST1,BCL7A,CRAT,FUT8,MAFG,SORT1 | 0.762695313 | 0.508333333333333 | 0.866666666666667 |
| 117 | Stepglm[backward]+Enet[alpha=0.8]+glmBoost | MGST1,BCL7A,CRAT,FUT8,MAFG,SORT1 | 0.762207031 | 0.508333333333333 | 0.866666666666667 |
| 118 | Stepglm[both]+Enet[alpha=0.2]+GBM | MGST1,BCL7A,CRAT,FUT8,MAFG,SORT1 | 0.761474609 | 0.508333333333333 | 0.866666666666667 |
| 119 | Stepglm[backward]+Enet[alpha=0.2]+XGBoost | MGST1,BCL7A,CRAT,FUT8,MAFG,SORT1 | 0.761474609 | 0.508333333333333 | 0.866666666666667 |
| 120 | Stepglm[both]+Lasso+GBM | MGST1,BCL7A,CRAT,FUT8,MAFG,SORT1 | 0.762451172 | 0.508333333333333 | 0.866666666666667 |
| 121 | Stepglm[backward]+Lasso+RF | MGST1,BCL7A,CRAT,FUT8,MAFG,SORT1 | 0.761962891 | 0.508333333333333 | 0.866666666666667 |
| 122 | Stepglm[both]+Enet[alpha=0.6]+GBM | MGST1,BCL7A,CRAT,FUT8,MAFG,SORT1 | 0.762451172 | 0.508333333333333 | 0.866666666666667 |
| 123 | Stepglm[backward]+Enet[alpha=0.6]+GBM | MGST1,BCL7A,CRAT,FUT8,MAFG,SORT1 | 0.761962891 | 0.508333333333333 | 0.866666666666667 |
| 124 | glmBoost+GBM+Lasso | BCL7A,FUT8,CRAT,MGST1,SDAD1,MAFG | 0.885742188 | 0.833333333333333 | 0.841666666666667 |
| 125 | Stepglm[both]+Enet[alpha=0.7]+GBM | MGST1,BCL7A,CRAT,FUT8,MAFG,SORT1 | 0.763427734 | 0.508333333333333 | 0.866666666666667 |
| 126 | Stepglm[backward]+Enet[alpha=0.7]+NaiveBayes | MGST1,BCL7A,CRAT,FUT8,MAFG,SORT1 | 0.763183594 | 0.508333333333333 | 0.866666666666667 |
| 127 | Lasso+Stepglm[backward]+Enet[alpha=0.5] | MGST1,BCL7A,CRAT,FUT8,MAFG | 0.756835938 | 0.558333333333333 | 0.866666666666667 |
| 128 | Lasso+glmBoost+Stepglm[both] | MGST1,BCL7A,CRAT,FUT8,MAFG,SDAD1 | 0.754638672 | 0.65 | 0.891666666666667 |
| 129 | glmBoost+Stepglm[backward]+GBM | MGST1,BCL7A,CRAT,FUT8,MAFG | 0.756835938 | 0.558333333333333 | 0.866666666666667 |
| 130 | Stepglm[both]+Enet[alpha=0.4]+glmBoost | MGST1,BCL7A,CRAT,FUT8,MAFG,SORT1 | 0.762207031 | 0.508333333333333 | 0.866666666666667 |
