# Supplementary material for: Integrated bioinformatics and machine learning for constructing a diagnostic model of major depressive disorder leveraging shared signatures from hemodialysis: A cross-sectional study
Source: Medicine (Baltimore). 2026 Jun 5;105(23):e49113. doi: 10.1097/MD.0000000000049113 (PMC13246050; doi:10.1097/MD.0000000000049113)
Supplement: Supplementary file 6 [file medi-105-e49113-s006.docx]

**Supplementary Table 6. Gene Set Enrichment Analysis (GSEA) Results of Core Genes**

| **Gene** | **Gene Set Name** | **Enrichment Score** | **Normalized Enrichment Score** | **P - value** | **False Discovery Rate** |
| --- | --- | --- | --- | --- | --- |
| BCL7A | PRIMARY_IMMUNODEFICIENCY | 0.5873 | 1.8071 | 0.004 | 0.0511 |
|  | SPLICEOSOME | 0.4016 | 1.8084 | 0.004 | 0.0762 |
|  | B_CELL_RECEPTOR_SIGNALING_PATHWAY | 0.3796 | 1.8282 | 0.0061 | 0.1223 |
|  | HOMOLOGOUS_RECOMBINATION | 0.5398 | 1.6924 | 0.01 | 0.124 |
|  | RIBOSOME | 0.524 | 1.634 | 0.0478 | 0.1446 |
|  | DNA_REPLICATION | 0.6325 | 1.6518 | 0.0078 | 0.1454 |
|  | TOLL_LIKE_RECEPTOR_SIGNALING_PATHWAY | -0.4528 | -1.8581 | 0 | 0.1748 |
|  | NUCLEOTIDE_EXCISION_REPAIR | 0.4909 | 1.5717 | 0.0431 | 0.2104 |
|  | FC_EPSILON_RI_SIGNALING_PATHWAY | -0.3581 | -1.6908 | 0.0038 | 0.2107 |
|  | NOD_LIKE_RECEPTOR_SIGNALING_PATHWAY | -0.4281 | -1.6459 | 0.0139 | 0.2156 |
|  | FOLATE_BIOSYNTHESIS | -0.6062 | -1.626 | 0.0179 | 0.2186 |
|  | BASE_EXCISION_REPAIR | 0.4546 | 1.519 | 0.0742 | 0.2221 |
|  | RNA_DEGRADATION | 0.3889 | 1.4782 | 0.0553 | 0.2259 |
|  | PHENYLALANINE_METABOLISM | -0.6099 | -1.6062 | 0.0225 | 0.2296 |
|  | MISMATCH_REPAIR | 0.601 | 1.4833 | 0.0698 | 0.2355 |
|  | ONE_CARBON_POOL_BY_FOLATE | 0.4827 | 1.5248 | 0.0503 | 0.2365 |
|  | CHEMOKINE_SIGNALING_PATHWAY | -0.317 | -1.5418 | 0.0156 | 0.2398 |
|  | ADIPOCYTOKINE_SIGNALING_PATHWAY | -0.3574 | -1.5509 | 0.0223 | 0.2406 |
|  | RNA_POLYMERASE | 0.4803 | 1.5364 | 0.0364 | 0.2434 |
|  | APOPTOSIS | -0.3541 | -1.6495 | 0.0116 | 0.2485 |
|  | FC_GAMMA_R_MEDIATED_PHAGOCYTOSIS | -0.3372 | -1.5798 | 0.0402 | 0.2526 |
| BCL7A | VALINE_LEUCINE_AND_ISOLEUCINE_DEGRADATION | 0.4922 | 1.4852 | 0.03 | 0.2535 |
|  | RENIN_ANGIOTENSIN_SYSTEM | -0.5925 | -1.5651 | 0.0346 | 0.2569 |
|  | ENDOCYTOSIS | -0.3172 | -1.5519 | 0.0142 | 0.2603 |
|  | EPITHELIAL_CELL_SIGNALING_IN_HELICOBACTER_PYLORI_INFECTION | -0.3792 | -1.693 | 0.0145 | 0.2784 |
|  | PPAR_SIGNALING_PATHWAY | -0.3585 | -1.3378 | 0.0522 | 0.314 |
|  | RENAL_CELL_CARCINOMA | -0.3141 | -1.3448 | 0.1087 | 0.3183 |
|  | TAURINE_AND_HYPOTAURINE_METABOLISM | -0.5559 | -1.3302 | 0.1352 | 0.3188 |
|  | ALPHA_LINOLENIC_ACID_METABOLISM | -0.4608 | -1.3395 | 0.1172 | 0.3192 |
|  | FOCAL_ADHESION | -0.2752 | -1.3237 | 0.0672 | 0.3232 |
|  | HISTIDINE_METABOLISM | -0.4316 | -1.3449 | 0.0982 | 0.3271 |
|  | CHRONIC_MYELOID_LEUKEMIA | -0.2756 | -1.3139 | 0.1284 | 0.3324 |
|  | GLYCEROPHOSPHOLIPID_METABOLISM | -0.3221 | -1.3462 | 0.0856 | 0.3343 |
|  | GNRH_SIGNALING_PATHWAY | -0.2909 | -1.3474 | 0.0784 | 0.3423 |
|  | LEISHMANIA_INFECTION | -0.3855 | -1.4792 | 0.0626 | 0.3465 |
|  | MELANOMA | -0.3425 | -1.3561 | 0.057 | 0.3465 |
|  | MTOR_SIGNALING_PATHWAY | -0.2948 | -1.3489 | 0.0798 | 0.3497 |
|  | TYPE_II_DIABETES_MELLITUS | -0.3551 | -1.3821 | 0.0902 | 0.3533 |
|  | HYPERTROPHIC_CARDIOMYOPATHY_HCM | -0.31 | -1.2969 | 0.0883 | 0.3536 |
|  | O_GLYCAN_BIOSYNTHESIS | -0.4195 | -1.3565 | 0.0994 | 0.3567 |
|  | ARACHIDONIC_ACID_METABOLISM | -0.4031 | -1.3727 | 0.0545 | 0.3602 |
|  | GLUTATHIONE_METABOLISM | -0.4057 | -1.3587 | 0.0842 | 0.3638 |
|  | INSULIN_SIGNALING_PATHWAY | -0.2619 | -1.3835 | 0.0553 | 0.3642 |
|  | CYSTEINE_AND_METHIONINE_METABOLISM | 0.3992 | 1.3941 | 0.0648 | 0.3659 |
|  | DILATED_CARDIOMYOPATHY | -0.3294 | -1.3877 | 0.0432 | 0.3707 |
|  | LYSOSOME | -0.3209 | -1.3614 | 0.1242 | 0.3716 |
| BCL7A | MAPK_SIGNALING_PATHWAY | -0.3231 | -1.7037 | 0 | 0.3764 |
|  | GLIOMA | -0.3198 | -1.2766 | 0.1845 | 0.3839 |
|  | ETHER_LIPID_METABOLISM | -0.4166 | -1.389 | 0.0675 | 0.3843 |
|  | RIG_I_LIKE_RECEPTOR_SIGNALING_PATHWAY | -0.3944 | -1.4008 | 0.0692 | 0.3905 |
|  | COMPLEMENT_AND_COAGULATION_CASCADES | -0.4264 | -1.3922 | 0.0489 | 0.3929 |
|  | PANCREATIC_CANCER | -0.3112 | -1.4419 | 0.0521 | 0.396 |
|  | NOTCH_SIGNALING_PATHWAY | -0.3474 | -1.4508 | 0.0862 | 0.3967 |
|  | P53_SIGNALING_PATHWAY | -0.3457 | -1.2659 | 0.129 | 0.3967 |
|  | REGULATION_OF_ACTIN_CYTOSKELETON | -0.2619 | -1.2602 | 0.0911 | 0.3978 |
|  | BLADDER_CANCER | -0.419 | -1.4011 | 0.0981 | 0.4091 |
|  | STEROID_BIOSYNTHESIS | 0.4891 | 1.3629 | 0.1039 | 0.4116 |
|  | AMYOTROPHIC_LATERAL_SCLEROSIS_ALS | -0.3344 | -1.4081 | 0.0569 | 0.412 |
|  | BUTANOATE_METABOLISM | 0.3814 | 1.2534 | 0.1882 | 0.4166 |
|  | MATURITY_ONSET_DIABETES_OF_THE_YOUNG | -0.4439 | -1.2409 | 0.1708 | 0.4181 |
|  | PROTEIN_EXPORT | 0.3681 | 1.245 | 0.2604 | 0.4181 |
|  | FRUCTOSE_AND_MANNOSE_METABOLISM | -0.4279 | -1.2445 | 0.236 | 0.4197 |
|  | GLYCOSAMINOGLYCAN_DEGRADATION | -0.429 | -1.4128 | 0.1053 | 0.4214 |
|  | N_GLYCAN_BIOSYNTHESIS | 0.3039 | 1.2342 | 0.1865 | 0.426 |
|  | AMINOACYL_TRNA_BIOSYNTHESIS | 0.5603 | 1.2258 | 0.2825 | 0.4283 |
|  | GLYOXYLATE_AND_DICARBOXYLATE_METABOLISM | 0.4312 | 1.2549 | 0.182 | 0.4285 |
|  | INTESTINAL_IMMUNE_NETWORK_FOR_IGA_PRODUCTION | 0.3894 | 1.2186 | 0.1786 | 0.4291 |
|  | PYRUVATE_METABOLISM | 0.341 | 1.2601 | 0.1537 | 0.4342 |
|  | GALACTOSE_METABOLISM | -0.3857 | -1.4136 | 0.0964 | 0.4439 |
|  | NON_HOMOLOGOUS_END_JOINING | 0.4783 | 1.2625 | 0.1434 | 0.4469 |
|  | PATHWAYS_IN_CANCER | -0.2401 | -1.2217 | 0.0881 | 0.4495 |
| BCL7A | STARCH_AND_SUCROSE_METABOLISM | -0.3828 | -1.2038 | 0.1896 | 0.4505 |
|  | REGULATION_OF_AUTOPHAGY | -0.3854 | -1.1974 | 0.2068 | 0.4556 |
|  | LEUKOCYTE_TRANSENDOTHELIAL_MIGRATION | -0.2645 | -1.2044 | 0.1936 | 0.4585 |
|  | HEMATOPOIETIC_CELL_LINEAGE | 0.3518 | 1.2909 | 0.0934 | 0.459 |
|  | PURINE_METABOLISM | 0.2926 | 1.2654 | 0.1149 | 0.4596 |
|  | GLYCOSYLPHOSPHATIDYLINOSITOL_GPI_ANCHOR_BIOSYNTHESIS | 0.4351 | 1.2811 | 0.2295 | 0.4627 |
|  | ACUTE_MYELOID_LEUKEMIA | -0.2948 | -1.2061 | 0.219 | 0.4643 |
|  | GLYCOSPHINGOLIPID_BIOSYNTHESIS_GLOBO_SERIES | -0.4433 | -1.1865 | 0.2625 | 0.4691 |
|  | GLYCOSAMINOGLYCAN_BIOSYNTHESIS_CHONDROITIN_SULFATE | -0.3679 | -1.1769 | 0.2552 | 0.4719 |
|  | NEUROTROPHIN_SIGNALING_PATHWAY | -0.2199 | -1.2067 | 0.1895 | 0.4725 |
|  | BASAL_TRANSCRIPTION_FACTORS | 0.3539 | 1.2941 | 0.1906 | 0.4738 |
|  | LIMONENE_AND_PINENE_DEGRADATION | 0.5872 | 1.267 | 0.2165 | 0.4762 |
|  | GLYCINE_SERINE_AND_THREONINE_METABOLISM | -0.4207 | -1.1791 | 0.2172 | 0.4765 |
|  | CYTOKINE_CYTOKINE_RECEPTOR_INTERACTION | -0.2733 | -1.1576 | 0.1653 | 0.4778 |
|  | ERBB_SIGNALING_PATHWAY | -0.264 | -1.1643 | 0.1943 | 0.4805 |
|  | NON_SMALL_CELL_LUNG_CANCER | -0.2742 | -1.1681 | 0.2391 | 0.4807 |
|  | VEGF_SIGNALING_PATHWAY | -0.2449 | -1.1599 | 0.2242 | 0.4812 |
|  | CIRCADIAN_RHYTHM_MAMMAL | 0.512 | 1.3111 | 0.1542 | 0.4864 |
|  | SELENOAMINO_ACID_METABOLISM | 0.3872 | 1.2978 | 0.1369 | 0.4905 |
|  | TERPENOID_BACKBONE_BIOSYNTHESIS | 0.3676 | 1.1791 | 0.2654 | 0.4969 |
|  | PROPANOATE_METABOLISM | 0.3929 | 1.3143 | 0.124 | 0.5075 |
|  | NICOTINATE_AND_NICOTINAMIDE_METABOLISM | -0.3246 | -1.1207 | 0.2983 | 0.5308 |
|  | VASCULAR_SMOOTH_MUSCLE_CONTRACTION | -0.252 | -1.123 | 0.2545 | 0.5347 |
|  | CITRATE_CYCLE_TCA_CYCLE | 0.3173 | 1.1522 | 0.2955 | 0.5408 |
|  | GLYCOSAMINOGLYCAN_BIOSYNTHESIS_KERATAN_SULFATE | 0.404 | 1.1443 | 0.3066 | 0.5427 |
| BCL7A | OTHER_GLYCAN_DEGRADATION | -0.3631 | -1.1232 | 0.343 | 0.5432 |
|  | ARRHYTHMOGENIC_RIGHT_VENTRICULAR_CARDIOMYOPATHY_ARVC | -0.29 | -1.1104 | 0.2625 | 0.5442 |
|  | GLYCOLYSIS_GLUCONEOGENESIS | -0.2813 | -1.0955 | 0.3093 | 0.5691 |
|  | BIOSYNTHESIS_OF_UNSATURATED_FATTY_ACIDS | -0.3554 | -1.0904 | 0.3198 | 0.5719 |
|  | CYTOSOLIC_DNA_SENSING_PATHWAY | -0.3342 | -1.0851 | 0.3527 | 0.5746 |
|  | BETA_ALANINE_METABOLISM | -0.4254 | -1.0708 | 0.3941 | 0.5884 |
|  | PROSTATE_CANCER | -0.221 | -1.0642 | 0.3659 | 0.5939 |
|  | GLYCOSAMINOGLYCAN_BIOSYNTHESIS_HEPARAN_SULFATE | -0.3524 | -1.0722 | 0.3452 | 0.594 |
|  | VIBRIO_CHOLERAE_INFECTION | -0.2177 | -1.0457 | 0.3783 | 0.6189 |
|  | NEUROACTIVE_LIGAND_RECEPTOR_INTERACTION | -0.2781 | -1.0471 | 0.3757 | 0.6242 |
|  | RIBOFLAVIN_METABOLISM | -0.4021 | -1.012 | 0.4568 | 0.6252 |
|  | PYRIMIDINE_METABOLISM | 0.2743 | 1.1002 | 0.3405 | 0.6269 |
|  | TRYPTOPHAN_METABOLISM | -0.3143 | -1.034 | 0.4085 | 0.6278 |
|  | FATTY_ACID_METABOLISM | -0.259 | -1.0131 | 0.4447 | 0.631 |
|  | TYROSINE_METABOLISM | -0.3079 | -1.0347 | 0.3971 | 0.6345 |
|  | METABOLISM_OF_XENOBIOTICS_BY_CYTOCHROME_P450 | -0.3112 | -1.0032 | 0.4491 | 0.6355 |
|  | NATURAL_KILLER_CELL_MEDIATED_CYTOTOXICITY | -0.251 | -1.0137 | 0.4142 | 0.6381 |
|  | PRION_DISEASES | -0.2812 | -0.9979 | 0.4665 | 0.6385 |
|  | PANTOTHENATE_AND_COA_BIOSYNTHESIS | -0.4426 | -1.0244 | 0.4376 | 0.6403 |
|  | DORSO_VENTRAL_AXIS_FORMATION | -0.3406 | -1.0137 | 0.4412 | 0.6463 |
|  | LONG_TERM_POTENTIATION | -0.2228 | -1.0173 | 0.4261 | 0.6474 |
|  | JAK_STAT_SIGNALING_PATHWAY | -0.2286 | -0.9811 | 0.4969 | 0.6504 |
|  | PORPHYRIN_AND_CHLOROPHYLL_METABOLISM | -0.3249 | -0.987 | 0.4429 | 0.6538 |
|  | PROXIMAL_TUBULE_BICARBONATE_RECLAMATION | -0.3184 | -0.9713 | 0.506 | 0.6556 |
|  | LINOLEIC_ACID_METABOLISM | -0.3244 | -0.9816 | 0.5179 | 0.6574 |
| BCL7A | ALZHEIMERS_DISEASE | -0.1894 | -0.9733 | 0.4727 | 0.6588 |
|  | AXON_GUIDANCE | -0.2054 | -0.9512 | 0.5567 | 0.6894 |
|  | INOSITOL_PHOSPHATE_METABOLISM | -0.2465 | -0.9405 | 0.5828 | 0.6959 |
|  | SMALL_CELL_LUNG_CANCER | 0.2399 | 1.0638 | 0.3249 | 0.6978 |
|  | GLYCEROLIPID_METABOLISM | -0.2677 | -0.9343 | 0.5633 | 0.7003 |
|  | AMINO_SUGAR_AND_NUCLEOTIDE_SUGAR_METABOLISM | -0.2841 | -0.9416 | 0.569 | 0.7018 |
|  | PROGESTERONE_MEDIATED_OOCYTE_MATURATION | -0.2057 | -0.9109 | 0.6579 | 0.7414 |
|  | GLYCOSPHINGOLIPID_BIOSYNTHESIS_LACTO_AND_NEOLACTO_SERIES | -0.2861 | -0.8915 | 0.63 | 0.7418 |
|  | PHOSPHATIDYLINOSITOL_SIGNALING_SYSTEM | -0.2001 | -0.9029 | 0.6586 | 0.7422 |
|  | ABC_TRANSPORTERS | -0.2645 | -0.8943 | 0.676 | 0.7435 |
|  | ENDOMETRIAL_CANCER | -0.2026 | -0.8865 | 0.6127 | 0.7441 |
|  | ADHERENS_JUNCTION | -0.2103 | -0.9049 | 0.5764 | 0.7459 |
|  | NITROGEN_METABOLISM | -0.3148 | -0.8956 | 0.5993 | 0.7483 |
|  | WNT_SIGNALING_PATHWAY | 0.2105 | 1.0361 | 0.3673 | 0.7494 |
|  | ALDOSTERONE_REGULATED_SODIUM_REABSORPTION | 0.27 | 1.014 | 0.4105 | 0.7496 |
|  | CELL_ADHESION_MOLECULES_CAMS | 0.2437 | 1.0268 | 0.3838 | 0.7537 |
|  | VIRAL_MYOCARDITIS | 0.2616 | 1.0153 | 0.447 | 0.7651 |
|  | ASTHMA | -0.3276 | -0.857 | 0.6804 | 0.7869 |
|  | DRUG_METABOLISM_OTHER_ENZYMES | -0.245 | -0.8517 | 0.7996 | 0.7887 |
|  | GAP_JUNCTION | -0.2124 | -0.8597 | 0.7168 | 0.7895 |
|  | DRUG_METABOLISM_CYTOCHROME_P450 | -0.265 | -0.8343 | 0.738 | 0.805 |
|  | ARGININE_AND_PROLINE_METABOLISM | -0.2294 | -0.8348 | 0.7072 | 0.8121 |
|  | ANTIGEN_PROCESSING_AND_PRESENTATION | 0.2971 | 0.8869 | 0.5673 | 0.8291 |
|  | ECM_RECEPTOR_INTERACTION | -0.2251 | -0.8157 | 0.8321 | 0.8304 |
|  | HUNTINGTONS_DISEASE | 0.1775 | 0.8718 | 0.5922 | 0.8315 |
| BCL7A | OOCYTE_MEIOSIS | -0.169 | -0.8091 | 0.833 | 0.8335 |
|  | PENTOSE_PHOSPHATE_PATHWAY | -0.2461 | -0.804 | 0.7263 | 0.8341 |
|  | CALCIUM_SIGNALING_PATHWAY | -0.1785 | -0.7983 | 0.846 | 0.8361 |
|  | CARDIAC_MUSCLE_CONTRACTION | -0.1874 | -0.793 | 0.8403 | 0.8377 |
|  | PROTEASOME | 0.2434 | 0.9408 | 0.521 | 0.8392 |
|  | MELANOGENESIS | 0.2075 | 0.933 | 0.5853 | 0.8402 |
|  | TGF_BETA_SIGNALING_PATHWAY | 0.225 | 0.9243 | 0.5892 | 0.8426 |
|  | PRIMARY_BILE_ACID_BIOSYNTHESIS | 0.3396 | 0.873 | 0.6725 | 0.8439 |
|  | PENTOSE_AND_GLUCURONATE_INTERCONVERSIONS | 0.3416 | 0.8869 | 0.6468 | 0.8449 |
|  | BASAL_CELL_CARCINOMA | 0.2422 | 0.8985 | 0.6072 | 0.8514 |
|  | LYSINE_DEGRADATION | 0.2701 | 0.9653 | 0.5298 | 0.8549 |
|  | T_CELL_RECEPTOR_SIGNALING_PATHWAY | 0.191 | 0.9411 | 0.5599 | 0.8577 |
|  | PARKINSONS_DISEASE | 0.1978 | 0.8883 | 0.5822 | 0.8582 |
|  | CELL_CYCLE | 0.2225 | 0.9479 | 0.5277 | 0.8602 |
|  | THYROID_CANCER | 0.2362 | 0.9004 | 0.6068 | 0.8638 |
|  | OLFACTORY_TRANSDUCTION | -0.2604 | -0.768 | 0.8239 | 0.8673 |
|  | LONG_TERM_DEPRESSION | -0.1945 | -0.7602 | 0.8672 | 0.8706 |
|  | TIGHT_JUNCTION | 0.1793 | 0.8427 | 0.8206 | 0.8769 |
|  | COLORECTAL_CANCER | 0.2212 | 0.9492 | 0.5255 | 0.8769 |
|  | STEROID_HORMONE_BIOSYNTHESIS | 0.2906 | 0.9021 | 0.6612 | 0.8775 |
|  | UBIQUITIN_MEDIATED_PROTEOLYSIS | 0.1872 | 0.8338 | 0.6998 | 0.8793 |
|  | ALLOGRAFT_REJECTION | 0.2986 | 0.8104 | 0.75 | 0.8931 |
|  | SYSTEMIC_LUPUS_ERYTHEMATOSUS | -0.2325 | -0.7317 | 0.8672 | 0.8934 |
|  | SPHINGOLIPID_METABOLISM | 0.2619 | 0.8162 | 0.725 | 0.8981 |
|  | RETINOL_METABOLISM | -0.2428 | -0.7341 | 0.8665 | 0.8984 |
| BCL7A | ALANINE_ASPARTATE_AND_GLUTAMATE_METABOLISM | -0.2071 | -0.718 | 0.8974 | 0.9014 |
|  | OXIDATIVE_PHOSPHORYLATION | 0.1852 | 0.789 | 0.6472 | 0.9159 |
|  | PEROXISOME | 0.2265 | 0.7699 | 0.778 | 0.9169 |
|  | ASCORBATE_AND_ALDARATE_METABOLISM | 0.3063 | 0.7786 | 0.8285 | 0.9178 |
|  | GLYCOSPHINGOLIPID_BIOSYNTHESIS_GANGLIO_SERIES | -0.2269 | -0.6603 | 0.8902 | 0.9318 |
|  | PATHOGENIC_ESCHERICHIA_COLI_INFECTION | -0.1729 | -0.6649 | 0.9074 | 0.9362 |
|  | GRAFT_VERSUS_HOST_DISEASE | 0.2685 | 0.6816 | 0.8481 | 0.9394 |
|  | SNARE_INTERACTIONS_IN_VESICULAR_TRANSPORT | -0.1775 | -0.6673 | 0.8568 | 0.9426 |
|  | TASTE_TRANSDUCTION | 0.2328 | 0.7057 | 0.9277 | 0.9436 |
|  | AUTOIMMUNE_THYROID_DISEASE | 0.2199 | 0.69 | 0.948 | 0.9456 |
|  | HEDGEHOG_SIGNALING_PATHWAY | 0.194 | 0.7263 | 0.9063 | 0.9487 |
|  | VASOPRESSIN_REGULATED_WATER_REABSORPTION | 0.1691 | 0.7127 | 0.9211 | 0.9501 |
|  | TYPE_I_DIABETES_MELLITUS | 0.2373 | 0.7295 | 0.905 | 0.9594 |
|  | SULFUR_METABOLISM | 0.1505 | 0.3959 | 1 | 0.9993 |
| CRAT | LYSOSOME | 0.4502 | 1.8926 | 0.0021 | 0.1344 |
|  | OTHER_GLYCAN_DEGRADATION | 0.5571 | 1.8046 | 0.0107 | 0.1652 |
|  | PATHOGENIC_ESCHERICHIA_COLI_INFECTION | 0.4378 | 1.6918 | 0.0159 | 0.3374 |
|  | LEUKOCYTE_TRANSENDOTHELIAL_MIGRATION | 0.3132 | 1.4561 | 0.0411 | 0.3901 |
|  | GALACTOSE_METABOLISM | 0.4003 | 1.4582 | 0.0688 | 0.4078 |
|  | ACUTE_MYELOID_LEUKEMIA | 0.3459 | 1.4609 | 0.0672 | 0.4267 |
|  | BASAL_TRANSCRIPTION_FACTORS | -0.4143 | -1.5243 | 0.0585 | 0.4338 |
|  | ARACHIDONIC_ACID_METABOLISM | 0.4409 | 1.4906 | 0.018 | 0.4403 |
|  | BLADDER_CANCER | 0.4396 | 1.4783 | 0.0737 | 0.4405 |
|  | LIMONENE_AND_PINENE_DEGRADATION | 0.6894 | 1.4987 | 0.035 | 0.4508 |
|  | GLYOXYLATE_AND_DICARBOXYLATE_METABOLISM | 0.4992 | 1.4614 | 0.0637 | 0.456 |
| CRAT | ARGININE_AND_PROLINE_METABOLISM | 0.3907 | 1.4022 | 0.087 | 0.4673 |
|  | TYROSINE_METABOLISM | 0.4203 | 1.4184 | 0.0353 | 0.4693 |
|  | FRUCTOSE_AND_MANNOSE_METABOLISM | 0.5287 | 1.5063 | 0.061 | 0.4719 |
|  | TERPENOID_BACKBONE_BIOSYNTHESIS | -0.4555 | -1.4774 | 0.0648 | 0.4854 |
|  | ENDOMETRIAL_CANCER | 0.3216 | 1.4041 | 0.0727 | 0.4861 |
|  | PARKINSONS_DISEASE | 0.305 | 1.386 | 0.1569 | 0.489 |
|  | HEMATOPOIETIC_CELL_LINEAGE | 0.4115 | 1.5139 | 0.0163 | 0.4933 |
|  | CHRONIC_MYELOID_LEUKEMIA | 0.3063 | 1.5195 | 0.0487 | 0.5348 |
|  | UBIQUITIN_MEDIATED_PROTEOLYSIS | -0.3523 | -1.5839 | 0.0262 | 0.5457 |
|  | RNA_DEGRADATION | -0.3854 | -1.5301 | 0.0624 | 0.5526 |
|  | ENDOCYTOSIS | 0.3143 | 1.5238 | 0.0192 | 0.5901 |
|  | CHEMOKINE_SIGNALING_PATHWAY | 0.2737 | 1.3354 | 0.0654 | 0.5943 |
|  | GLYCOLYSIS_GLUCONEOGENESIS | 0.3335 | 1.3195 | 0.1365 | 0.5945 |
|  | ALZHEIMERS_DISEASE | 0.2624 | 1.298 | 0.166 | 0.5969 |
|  | BASE_EXCISION_REPAIR | 0.3752 | 1.2684 | 0.2173 | 0.6062 |
|  | SYSTEMIC_LUPUS_ERYTHEMATOSUS | 0.4146 | 1.3236 | 0.0928 | 0.607 |
|  | GLYCINE_SERINE_AND_THREONINE_METABOLISM | 0.4804 | 1.3392 | 0.0731 | 0.6093 |
|  | EPITHELIAL_CELL_SIGNALING_IN_HELICOBACTER_PYLORI_INFECTION | 0.2711 | 1.2416 | 0.1717 | 0.612 |
|  | COMPLEMENT_AND_COAGULATION_CASCADES | 0.4003 | 1.3066 | 0.0917 | 0.6125 |
|  | CYTOSOLIC_DNA_SENSING_PATHWAY | 0.3918 | 1.2477 | 0.1847 | 0.6128 |
|  | GLUTATHIONE_METABOLISM | 0.3946 | 1.2988 | 0.1149 | 0.6165 |
|  | GLYCEROPHOSPHOLIPID_METABOLISM | 0.3102 | 1.2692 | 0.1417 | 0.6236 |
|  | TAURINE_AND_HYPOTAURINE_METABOLISM | 0.4937 | 1.1869 | 0.2709 | 0.6273 |
|  | GLYCEROLIPID_METABOLISM | 0.3702 | 1.2745 | 0.1414 | 0.6278 |
|  | PENTOSE_PHOSPHATE_PATHWAY | 0.3838 | 1.2548 | 0.1957 | 0.6283 |
| CRAT | ALPHA_LINOLENIC_ACID_METABOLISM | 0.4374 | 1.2478 | 0.1833 | 0.6307 |
|  | VEGF_SIGNALING_PATHWAY | 0.2509 | 1.1965 | 0.1914 | 0.6308 |
|  | LEISHMANIA_INFECTION | 0.3166 | 1.2122 | 0.2273 | 0.6309 |
|  | NON_SMALL_CELL_LUNG_CANCER | 0.2824 | 1.2288 | 0.1882 | 0.6322 |
|  | PORPHYRIN_AND_CHLOROPHYLL_METABOLISM | 0.4263 | 1.2796 | 0.1363 | 0.6333 |
|  | TIGHT_JUNCTION | 0.257 | 1.2219 | 0.1232 | 0.635 |
|  | SNARE_INTERACTIONS_IN_VESICULAR_TRANSPORT | 0.3116 | 1.1886 | 0.2465 | 0.6365 |
|  | PPAR_SIGNALING_PATHWAY | 0.3289 | 1.2154 | 0.1429 | 0.6373 |
|  | NATURAL_KILLER_CELL_MEDIATED_CYTOTOXICITY | 0.2997 | 1.1986 | 0.2617 | 0.6398 |
|  | GLYCOSAMINOGLYCAN_DEGRADATION | 0.3592 | 1.2032 | 0.2485 | 0.6411 |
|  | P53_SIGNALING_PATHWAY | -0.3561 | -1.2968 | 0.0907 | 0.6446 |
|  | HUNTINGTONS_DISEASE | 0.308 | 1.531 | 0.048 | 0.6472 |
|  | OXIDATIVE_PHOSPHORYLATION | 0.3613 | 1.5757 | 0.0847 | 0.6771 |
|  | PHENYLALANINE_METABOLISM | 0.5909 | 1.5448 | 0.0248 | 0.6885 |
|  | TGF_BETA_SIGNALING_PATHWAY | -0.3203 | -1.3012 | 0.0835 | 0.6922 |
|  | CELL_CYCLE | -0.312 | -1.3173 | 0.1208 | 0.7027 |
|  | ASTHMA | 0.429 | 1.1544 | 0.2638 | 0.7039 |
|  | AMINO_SUGAR_AND_NUCLEOTIDE_SUGAR_METABOLISM | 0.3409 | 1.1232 | 0.3153 | 0.7081 |
|  | PHOSPHATIDYLINOSITOL_SIGNALING_SYSTEM | 0.2501 | 1.1156 | 0.2337 | 0.7147 |
|  | FC_GAMMA_R_MEDIATED_PHAGOCYTOSIS | 0.2402 | 1.1257 | 0.2881 | 0.7147 |
|  | APOPTOSIS | 0.2382 | 1.1273 | 0.2682 | 0.7242 |
|  | FOCAL_ADHESION | 0.2388 | 1.1412 | 0.2051 | 0.7271 |
|  | THYROID_CANCER | 0.3086 | 1.13 | 0.2908 | 0.7304 |
|  | PROSTATE_CANCER | 0.219 | 1.0507 | 0.3716 | 0.7331 |
|  | PANCREATIC_CANCER | 0.2383 | 1.1041 | 0.2988 | 0.7341 |
| CRAT | DRUG_METABOLISM_OTHER_ENZYMES | 0.3122 | 1.0538 | 0.3636 | 0.736 |
|  | NEUROTROPHIN_SIGNALING_PATHWAY | 0.2044 | 1.1327 | 0.2695 | 0.7372 |
|  | NOTCH_SIGNALING_PATHWAY | 0.2548 | 1.0914 | 0.3693 | 0.7426 |
|  | PATHWAYS_IN_CANCER | 0.2049 | 1.0548 | 0.3114 | 0.7444 |
|  | ECM_RECEPTOR_INTERACTION | 0.3055 | 1.0953 | 0.2883 | 0.7446 |
|  | ADHERENS_JUNCTION | 0.2481 | 1.0627 | 0.3507 | 0.7463 |
|  | MAPK_SIGNALING_PATHWAY | 0.1973 | 1.0314 | 0.3694 | 0.7509 |
|  | PYRUVATE_METABOLISM | 0.2764 | 1.0555 | 0.3803 | 0.7545 |
|  | INSULIN_SIGNALING_PATHWAY | 0.1973 | 1.0341 | 0.3934 | 0.7546 |
|  | LONG_TERM_POTENTIATION | -0.2857 | -1.3262 | 0.1189 | 0.755 |
|  | VIBRIO_CHOLERAE_INFECTION | 0.2244 | 1.0634 | 0.374 | 0.7563 |
|  | BETA_ALANINE_METABOLISM | 0.4316 | 1.0815 | 0.3852 | 0.7565 |
|  | BIOSYNTHESIS_OF_UNSATURATED_FATTY_ACIDS | 0.3366 | 1.0362 | 0.3952 | 0.7602 |
|  | TYPE_II_DIABETES_MELLITUS | 0.2708 | 1.0664 | 0.3353 | 0.7617 |
|  | FOLATE_BIOSYNTHESIS | -0.472 | -1.2157 | 0.2453 | 0.7634 |
|  | PURINE_METABOLISM | 0.242 | 1.069 | 0.3033 | 0.7674 |
|  | CARDIAC_MUSCLE_CONTRACTION | 0.2591 | 1.0728 | 0.3261 | 0.769 |
|  | PENTOSE_AND_GLUCURONATE_INTERCONVERSIONS | -0.479 | -1.2443 | 0.1531 | 0.7775 |
|  | PROPANOATE_METABOLISM | -0.3692 | -1.2264 | 0.2004 | 0.7837 |
|  | CITRATE_CYCLE_TCA_CYCLE | 0.278 | 1.0127 | 0.4363 | 0.7905 |
|  | GAP_JUNCTION | 0.252 | 0.994 | 0.4508 | 0.8074 |
|  | OOCYTE_MEIOSIS | -0.2875 | -1.3658 | 0.0747 | 0.8091 |
|  | HISTIDINE_METABOLISM | 0.3233 | 0.995 | 0.4492 | 0.8156 |
|  | PROXIMAL_TUBULE_BICARBONATE_RECLAMATION | 0.325 | 0.9991 | 0.4576 | 0.8161 |
|  | SULFUR_METABOLISM | 0.3767 | 0.9811 | 0.485 | 0.819 |
| CRAT | DILATED_CARDIOMYOPATHY | 0.2388 | 0.9838 | 0.4951 | 0.823 |
|  | PROTEIN_EXPORT | -0.3784 | -1.3322 | 0.2016 | 0.8369 |
|  | GLYCOSYLPHOSPHATIDYLINOSITOL_GPI_ANCHOR_BIOSYNTHESIS | -0.5289 | -1.6166 | 0.0514 | 0.8391 |
|  | ONE_CARBON_POOL_BY_FOLATE | -0.289 | -0.9218 | 0.5755 | 0.8396 |
|  | CIRCADIAN_RHYTHM_MAMMAL | 0.3713 | 0.9392 | 0.5545 | 0.8548 |
|  | GLYCOSAMINOGLYCAN_BIOSYNTHESIS_KERATAN_SULFATE | -0.3225 | -0.9245 | 0.5565 | 0.8557 |
|  | GRAFT_VERSUS_HOST_DISEASE | 0.376 | 0.9325 | 0.5714 | 0.861 |
|  | VASCULAR_SMOOTH_MUSCLE_CONTRACTION | 0.2114 | 0.9392 | 0.5511 | 0.8653 |
|  | TOLL_LIKE_RECEPTOR_SIGNALING_PATHWAY | 0.2282 | 0.9429 | 0.5339 | 0.866 |
|  | VALINE_LEUCINE_AND_ISOLEUCINE_BIOSYNTHESIS | 0.4756 | 0.9585 | 0.5833 | 0.8689 |
|  | GLIOMA | 0.2312 | 0.9454 | 0.5301 | 0.8701 |
|  | FATTY_ACID_METABOLISM | 0.2416 | 0.9496 | 0.5604 | 0.8703 |
|  | INOSITOL_PHOSPHATE_METABOLISM | -0.2321 | -0.8991 | 0.6573 | 0.8704 |
|  | BUTANOATE_METABOLISM | -0.2469 | -0.8138 | 0.7278 | 0.8713 |
|  | FC_EPSILON_RI_SIGNALING_PATHWAY | 0.1961 | 0.9201 | 0.5985 | 0.8722 |
|  | CYTOKINE_CYTOKINE_RECEPTOR_INTERACTION | 0.2016 | 0.8554 | 0.7988 | 0.8727 |
|  | NITROGEN_METABOLISM | 0.3076 | 0.8709 | 0.6234 | 0.8732 |
|  | PROTEASOME | -0.2594 | -1.0073 | 0.4382 | 0.8736 |
|  | CYSTEINE_AND_METHIONINE_METABOLISM | -0.2841 | -0.9961 | 0.4638 | 0.8743 |
|  | GNRH_SIGNALING_PATHWAY | 0.1857 | 0.8582 | 0.7078 | 0.8752 |
|  | STEROID_BIOSYNTHESIS | -0.3368 | -0.9258 | 0.5663 | 0.8762 |
|  | MTOR_SIGNALING_PATHWAY | 0.2148 | 0.951 | 0.5166 | 0.8777 |
|  | TASTE_TRANSDUCTION | -0.2692 | -0.8291 | 0.7554 | 0.8793 |
|  | SPHINGOLIPID_METABOLISM | -0.2714 | -0.8385 | 0.702 | 0.8794 |
|  | HYPERTROPHIC_CARDIOMYOPATHY_HCM | 0.2068 | 0.8602 | 0.7192 | 0.8797 |
| CRAT | PRION_DISEASES | 0.2405 | 0.8713 | 0.6497 | 0.8815 |
|  | ANTIGEN_PROCESSING_AND_PRESENTATION | 0.311 | 0.9204 | 0.5366 | 0.8817 |
|  | PYRIMIDINE_METABOLISM | -0.2045 | -0.8467 | 0.7185 | 0.8821 |
|  | NOD_LIKE_RECEPTOR_SIGNALING_PATHWAY | 0.2233 | 0.8625 | 0.649 | 0.8837 |
|  | STARCH_AND_SUCROSE_METABOLISM | -0.2626 | -0.8167 | 0.773 | 0.8848 |
|  | ABC_TRANSPORTERS | -0.2635 | -0.8829 | 0.6887 | 0.886 |
|  | PEROXISOME | -0.2517 | -0.8639 | 0.6541 | 0.8861 |
|  | REGULATION_OF_ACTIN_CYTOSKELETON | 0.1831 | 0.8816 | 0.6871 | 0.8863 |
|  | TRYPTOPHAN_METABOLISM | 0.2716 | 0.8769 | 0.7018 | 0.888 |
|  | LINOLEIC_ACID_METABOLISM | 0.2969 | 0.8844 | 0.6667 | 0.8892 |
|  | RIG_I_LIKE_RECEPTOR_SIGNALING_PATHWAY | 0.2491 | 0.8717 | 0.6653 | 0.8898 |
|  | AXON_GUIDANCE | 0.1933 | 0.9034 | 0.6641 | 0.8923 |
|  | ADIPOCYTOKINE_SIGNALING_PATHWAY | 0.2001 | 0.8861 | 0.6458 | 0.8947 |
|  | GLYCOSAMINOGLYCAN_BIOSYNTHESIS_HEPARAN_SULFATE | -0.321 | -0.9684 | 0.5051 | 0.8951 |
|  | GLYCOSPHINGOLIPID_BIOSYNTHESIS_LACTO_AND_NEOLACTO_SERIES | -0.2773 | -0.8696 | 0.688 | 0.8951 |
|  | O_GLYCAN_BIOSYNTHESIS | -0.2607 | -0.8493 | 0.6972 | 0.8972 |
|  | GLYCOSPHINGOLIPID_BIOSYNTHESIS_GANGLIO_SERIES | -0.3299 | -0.927 | 0.567 | 0.8979 |
|  | SPLICEOSOME | -0.201 | -0.9372 | 0.5306 | 0.898 |
|  | AMYOTROPHIC_LATERAL_SCLEROSIS_ALS | 0.1938 | 0.8333 | 0.7722 | 0.9009 |
|  | VASOPRESSIN_REGULATED_WATER_REABSORPTION | 0.2222 | 0.9035 | 0.6433 | 0.9023 |
|  | DORSO_VENTRAL_AXIS_FORMATION | -0.3304 | -1.0085 | 0.4622 | 0.9026 |
|  | LONG_TERM_DEPRESSION | 0.2257 | 0.8869 | 0.6468 | 0.9027 |
|  | DNA_REPLICATION | 0.3337 | 0.8942 | 0.5816 | 0.9054 |
|  | MELANOGENESIS | -0.1729 | -0.7833 | 0.8595 | 0.9068 |
|  | RNA_POLYMERASE | 0.2512 | 0.8248 | 0.674 | 0.9093 |
| CRAT | BASAL_CELL_CARCINOMA | 0.2221 | 0.8334 | 0.6848 | 0.9095 |
|  | NUCLEOTIDE_EXCISION_REPAIR | 0.2753 | 0.8882 | 0.5842 | 0.9098 |
|  | ALANINE_ASPARTATE_AND_GLUTAMATE_METABOLISM | 0.2379 | 0.8194 | 0.7106 | 0.9112 |
|  | JAK_STAT_SIGNALING_PATHWAY | 0.1841 | 0.8029 | 0.9201 | 0.9174 |
|  | HOMOLOGOUS_RECOMBINATION | -0.3112 | -0.971 | 0.5 | 0.9175 |
|  | RENIN_ANGIOTENSIN_SYSTEM | 0.2996 | 0.8045 | 0.7085 | 0.9226 |
|  | MELANOMA | -0.2368 | -0.9378 | 0.5825 | 0.9237 |
|  | NICOTINATE_AND_NICOTINAMIDE_METABOLISM | -0.216 | -0.7591 | 0.8105 | 0.9259 |
|  | LYSINE_DEGRADATION | -0.1919 | -0.6836 | 0.9156 | 0.9283 |
|  | VALINE_LEUCINE_AND_ISOLEUCINE_DEGRADATION | -0.3727 | -1.1557 | 0.2715 | 0.9287 |
|  | WNT_SIGNALING_PATHWAY | -0.1925 | -0.9458 | 0.5225 | 0.9292 |
|  | ETHER_LIPID_METABOLISM | 0.2452 | 0.8049 | 0.7827 | 0.9308 |
|  | SMALL_CELL_LUNG_CANCER | -0.2332 | -1.0096 | 0.4494 | 0.9338 |
|  | PANTOTHENATE_AND_COA_BIOSYNTHESIS | 0.3355 | 0.7625 | 0.7746 | 0.9357 |
|  | GLYCOSAMINOGLYCAN_BIOSYNTHESIS_CHONDROITIN_SULFATE | -0.2147 | -0.6886 | 0.8627 | 0.941 |
|  | AMINOACYL_TRNA_BIOSYNTHESIS | 0.3397 | 0.7429 | 0.7737 | 0.9415 |
|  | ALDOSTERONE_REGULATED_SODIUM_REABSORPTION | 0.2051 | 0.7637 | 0.8274 | 0.9419 |
|  | PRIMARY_IMMUNODEFICIENCY | 0.2334 | 0.7473 | 0.7665 | 0.9429 |
|  | ARRHYTHMOGENIC_RIGHT_VENTRICULAR_CARDIOMYOPATHY_ARVC | 0.2003 | 0.7516 | 0.9023 | 0.9442 |
|  | REGULATION_OF_AUTOPHAGY | -0.2327 | -0.7178 | 0.8948 | 0.9449 |
|  | OLFACTORY_TRANSDUCTION | -0.2483 | -0.7312 | 0.8905 | 0.9464 |
|  | NON_HOMOLOGOUS_END_JOINING | -0.387 | -1.0409 | 0.4142 | 0.9468 |
|  | HEDGEHOG_SIGNALING_PATHWAY | -0.1836 | -0.6984 | 0.9163 | 0.9482 |
|  | SELENOAMINO_ACID_METABOLISM | 0.2258 | 0.7646 | 0.8 | 0.949 |
|  | RIBOFLAVIN_METABOLISM | 0.3085 | 0.7749 | 0.8113 | 0.9494 |
| CRAT | GLYCOSPHINGOLIPID_BIOSYNTHESIS_GLOBO_SERIES | 0.2909 | 0.7674 | 0.7789 | 0.9532 |
| FUT8 | RNA_DEGRADATION | -0.5004 | -1.9071 | 0 | 0.0311 |
|  | SPLICEOSOME | -0.4286 | -1.9382 | 0.0019 | 0.0411 |
|  | RIBOSOME | -0.5585 | -1.766 | 0.0284 | 0.0606 |
|  | VALINE_LEUCINE_AND_ISOLEUCINE_DEGRADATION | -0.5688 | -1.7051 | 0 | 0.0648 |
|  | N_GLYCAN_BIOSYNTHESIS | -0.433 | -1.7074 | 0.0097 | 0.0704 |
|  | BASAL_TRANSCRIPTION_FACTORS | -0.4846 | -1.7685 | 0.0077 | 0.0718 |
|  | PEROXISOME | -0.5058 | -1.7137 | 0.0019 | 0.0759 |
|  | NUCLEOTIDE_EXCISION_REPAIR | -0.5356 | -1.7196 | 0.0058 | 0.0825 |
|  | UBIQUITIN_MEDIATED_PROTEOLYSIS | -0.4084 | -1.7707 | 0 | 0.0866 |
|  | PROTEIN_EXPORT | -0.5291 | -1.7823 | 0.0155 | 0.099 |
|  | CELL_CYCLE | -0.3873 | -1.6501 | 0.0076 | 0.0996 |
|  | NOTCH_SIGNALING_PATHWAY | 0.4404 | 1.8516 | 0.0056 | 0.1179 |
|  | MISMATCH_REPAIR | -0.6461 | -1.5984 | 0.0173 | 0.141 |
|  | DNA_REPLICATION | -0.5891 | -1.5117 | 0.0314 | 0.1981 |
|  | AMINOACYL_TRNA_BIOSYNTHESIS | -0.7048 | -1.5172 | 0.021 | 0.2014 |
|  | PANTOTHENATE_AND_COA_BIOSYNTHESIS | -0.6787 | -1.5396 | 0.0297 | 0.2055 |
|  | T_CELL_RECEPTOR_SIGNALING_PATHWAY | -0.3131 | -1.5203 | 0.0295 | 0.2096 |
|  | PYRIMIDINE_METABOLISM | -0.3789 | -1.5267 | 0.0216 | 0.2124 |
|  | PROPANOATE_METABOLISM | -0.4398 | -1.4597 | 0.0593 | 0.2406 |
|  | GLYCOSYLPHOSPHATIDYLINOSITOL_GPI_ANCHOR_BIOSYNTHESIS | -0.4995 | -1.4661 | 0.0611 | 0.2422 |
|  | BETA_ALANINE_METABOLISM | -0.5954 | -1.4709 | 0.0361 | 0.2483 |
|  | COLORECTAL_CANCER | -0.3391 | -1.4322 | 0.0518 | 0.2778 |
|  | GLYCOSAMINOGLYCAN_BIOSYNTHESIS_KERATAN_SULFATE | -0.4964 | -1.3907 | 0.0875 | 0.3435 |
|  | TERPENOID_BACKBONE_BIOSYNTHESIS | -0.419 | -1.3821 | 0.1117 | 0.3462 |
| FUT8 | CITRATE_CYCLE_TCA_CYCLE | -0.3816 | -1.3474 | 0.1479 | 0.4073 |
|  | LYSINE_DEGRADATION | -0.3691 | -1.3179 | 0.1203 | 0.4443 |
|  | CYSTEINE_AND_METHIONINE_METABOLISM | -0.3768 | -1.3237 | 0.1078 | 0.4475 |
|  | PURINE_METABOLISM | -0.2996 | -1.2935 | 0.0841 | 0.4546 |
|  | SELENOAMINO_ACID_METABOLISM | -0.386 | -1.2936 | 0.1207 | 0.4707 |
|  | PROTEASOME | -0.3403 | -1.2959 | 0.1629 | 0.4817 |
|  | NICOTINATE_AND_NICOTINAMIDE_METABOLISM | -0.3675 | -1.2656 | 0.1564 | 0.5091 |
|  | AMINO_SUGAR_AND_NUCLEOTIDE_SUGAR_METABOLISM | -0.3913 | -1.2576 | 0.17 | 0.5134 |
|  | ONE_CARBON_POOL_BY_FOLATE | -0.3802 | -1.2295 | 0.2222 | 0.5534 |
|  | SMALL_CELL_LUNG_CANCER | -0.2844 | -1.231 | 0.1178 | 0.5662 |
|  | SPHINGOLIPID_METABOLISM | -0.3794 | -1.1752 | 0.2617 | 0.5983 |
|  | HOMOLOGOUS_RECOMBINATION | -0.3809 | -1.1835 | 0.2275 | 0.6077 |
|  | OOCYTE_MEIOSIS | -0.2484 | -1.1763 | 0.2107 | 0.6108 |
|  | BUTANOATE_METABOLISM | -0.3642 | -1.1847 | 0.2279 | 0.6215 |
|  | P53_SIGNALING_PATHWAY | -0.3313 | -1.196 | 0.1874 | 0.6248 |
|  | NON_HOMOLOGOUS_END_JOINING | -0.4484 | -1.1877 | 0.2243 | 0.6312 |
|  | CIRCADIAN_RHYTHM_MAMMAL | -0.4559 | -1.1509 | 0.2959 | 0.6515 |
|  | LIMONENE_AND_PINENE_DEGRADATION | -0.5327 | -1.1448 | 0.3211 | 0.6522 |
|  | ABC_TRANSPORTERS | -0.34 | -1.1371 | 0.2147 | 0.6571 |
|  | PRIMARY_IMMUNODEFICIENCY | -0.3509 | -1.076 | 0.3876 | 0.6774 |
|  | TGF_BETA_SIGNALING_PATHWAY | -0.2722 | -1.1077 | 0.2824 | 0.6777 |
|  | GLYCOSAMINOGLYCAN_BIOSYNTHESIS_HEPARAN_SULFATE | -0.3668 | -1.118 | 0.2939 | 0.6794 |
|  | FATTY_ACID_METABOLISM | -0.2886 | -1.1114 | 0.2597 | 0.6824 |
|  | GLYCOSPHINGOLIPID_BIOSYNTHESIS_GANGLIO_SERIES | -0.3707 | -1.0772 | 0.3644 | 0.6877 |
|  | INOSITOL_PHOSPHATE_METABOLISM | -0.2881 | -1.1205 | 0.2615 | 0.6881 |
| FUT8 | JAK_STAT_SIGNALING_PATHWAY | -0.2457 | -1.0782 | 0.2749 | 0.6989 |
|  | RNA_POLYMERASE | -0.3394 | -1.0816 | 0.3726 | 0.704 |
|  | RIBOFLAVIN_METABOLISM | -0.4453 | -1.0911 | 0.3592 | 0.7084 |
|  | REGULATION_OF_AUTOPHAGY | -0.3488 | -1.0818 | 0.3307 | 0.7179 |
|  | GLYCOSPHINGOLIPID_BIOSYNTHESIS_LACTO_AND_NEOLACTO_SERIES | -0.3362 | -1.0175 | 0.4535 | 0.778 |
|  | NOD_LIKE_RECEPTOR_SIGNALING_PATHWAY | -0.2643 | -1.0226 | 0.4297 | 0.7785 |
|  | PENTOSE_AND_GLUCURONATE_INTERCONVERSIONS | -0.4078 | -1.0266 | 0.415 | 0.7819 |
|  | PROGESTERONE_MEDIATED_OOCYTE_MATURATION | -0.2301 | -1.0306 | 0.3865 | 0.7857 |
|  | DRUG_METABOLISM_OTHER_ENZYMES | -0.291 | -0.9963 | 0.4558 | 0.8081 |
|  | STARCH_AND_SUCROSE_METABOLISM | -0.3163 | -1.0002 | 0.4544 | 0.8108 |
|  | VALINE_LEUCINE_AND_ISOLEUCINE_BIOSYNTHESIS | -0.4876 | -0.9659 | 0.5697 | 0.8481 |
|  | ENDOCYTOSIS | -0.2012 | -0.9754 | 0.499 | 0.8504 |
|  | GLYOXYLATE_AND_DICARBOXYLATE_METABOLISM | -0.3252 | -0.9584 | 0.5305 | 0.8549 |
|  | RIG_I_LIKE_RECEPTOR_SIGNALING_PATHWAY | -0.2752 | -0.9686 | 0.521 | 0.8552 |
|  | ALANINE_ASPARTATE_AND_GLUTAMATE_METABOLISM | -0.2719 | -0.9416 | 0.5481 | 0.8712 |
|  | ERBB_SIGNALING_PATHWAY | -0.2152 | -0.9442 | 0.532 | 0.8782 |
|  | B_CELL_RECEPTOR_SIGNALING_PATHWAY | -0.1959 | -0.9321 | 0.5369 | 0.8825 |
|  | ASCORBATE_AND_ALDARATE_METABOLISM | -0.3648 | -0.916 | 0.6019 | 0.8956 |
|  | O_GLYCAN_BIOSYNTHESIS | -0.281 | -0.9212 | 0.558 | 0.896 |
|  | VASOPRESSIN_REGULATED_WATER_REABSORPTION | -0.2165 | -0.9039 | 0.6375 | 0.9129 |
|  | APOPTOSIS | -0.1906 | -0.8967 | 0.6564 | 0.9169 |
|  | INTESTINAL_IMMUNE_NETWORK_FOR_IGA_PRODUCTION | -0.2834 | -0.8761 | 0.6602 | 0.9274 |
|  | AMYOTROPHIC_LATERAL_SCLEROSIS_ALS | -0.2012 | -0.8698 | 0.7132 | 0.9286 |
|  | VIRAL_MYOCARDITIS | -0.2305 | -0.8853 | 0.6424 | 0.9322 |
|  | ADHERENS_JUNCTION | -0.1974 | -0.8789 | 0.6505 | 0.9336 |
| FUT8 | SNARE_INTERACTIONS_IN_VESICULAR_TRANSPORT | -0.2337 | -0.8612 | 0.645 | 0.9357 |
|  | ETHER_LIPID_METABOLISM | -0.2589 | -0.8541 | 0.7166 | 0.9386 |
|  | ARRHYTHMOGENIC_RIGHT_VENTRICULAR_CARDIOMYOPATHY_ARVC | -0.2196 | -0.848 | 0.7686 | 0.9394 |
|  | NITROGEN_METABOLISM | -0.2874 | -0.8321 | 0.7349 | 0.9612 |
|  | AUTOIMMUNE_THYROID_DISEASE | 0.216 | 0.6761 | 0.9527 | 0.9649 |
|  | BIOSYNTHESIS_OF_UNSATURATED_FATTY_ACIDS | -0.2714 | -0.8114 | 0.7375 | 0.9682 |
|  | ARACHIDONIC_ACID_METABOLISM | 0.1946 | 0.6521 | 0.9766 | 0.972 |
|  | TOLL_LIKE_RECEPTOR_SIGNALING_PATHWAY | 0.1685 | 0.6814 | 0.9422 | 0.9725 |
|  | ASTHMA | 0.242 | 0.6297 | 0.9442 | 0.9752 |
|  | ALPHA_LINOLENIC_ACID_METABOLISM | -0.2244 | -0.6513 | 0.908 | 0.976 |
|  | MELANOMA | 0.1755 | 0.6902 | 0.9345 | 0.9768 |
|  | GAP_JUNCTION | -0.2034 | -0.8009 | 0.8228 | 0.977 |
|  | SYSTEMIC_LUPUS_ERYTHEMATOSUS | 0.2255 | 0.7029 | 0.8908 | 0.977 |
|  | FOLATE_BIOSYNTHESIS | -0.306 | -0.8129 | 0.701 | 0.9773 |
|  | TYPE_I_DIABETES_MELLITUS | -0.203 | -0.6141 | 0.9822 | 0.9798 |
|  | DORSO_VENTRAL_AXIS_FORMATION | 0.2467 | 0.7426 | 0.7845 | 0.9802 |
|  | FOCAL_ADHESION | -0.1598 | -0.7801 | 0.9387 | 0.9805 |
|  | GLYCINE_SERINE_AND_THREONINE_METABOLISM | 0.2554 | 0.7105 | 0.9324 | 0.9813 |
|  | HISTIDINE_METABOLISM | -0.2631 | -0.8167 | 0.7716 | 0.9819 |
|  | PROSTATE_CANCER | 0.1525 | 0.7317 | 0.8958 | 0.9822 |
|  | PORPHYRIN_AND_CHLOROPHYLL_METABOLISM | -0.2072 | -0.6236 | 0.9669 | 0.9847 |
|  | ALZHEIMERS_DISEASE | -0.1341 | -0.6527 | 0.9089 | 0.9848 |
|  | TRYPTOPHAN_METABOLISM | -0.1776 | -0.5687 | 1 | 0.988 |
|  | GRAFT_VERSUS_HOST_DISEASE | -0.261 | -0.6603 | 0.8674 | 0.9895 |
|  | PATHWAYS_IN_CANCER | -0.1507 | -0.781 | 0.9619 | 0.9909 |
| FUT8 | EPITHELIAL_CELL_SIGNALING_IN_HELICOBACTER_PYLORI_INFECTION | 0.1665 | 0.7442 | 0.8201 | 0.9918 |
|  | OXIDATIVE_PHOSPHORYLATION | -0.1701 | -0.7103 | 0.7325 | 0.9925 |
|  | PHOSPHATIDYLINOSITOL_SIGNALING_SYSTEM | 0.1569 | 0.7119 | 0.9581 | 0.9931 |
|  | ECM_RECEPTOR_INTERACTION | -0.1825 | -0.6671 | 0.9876 | 0.9944 |
|  | HEMATOPOIETIC_CELL_LINEAGE | -0.1931 | -0.7141 | 0.9601 | 0.9989 |
|  | STEROID_HORMONE_BIOSYNTHESIS | 0.1319 | 0.4004 | 1 | 0.999 |
|  | LONG_TERM_DEPRESSION | -0.1976 | -0.7828 | 0.8546 | 0.9993 |
|  | NATURAL_KILLER_CELL_MEDIATED_CYTOTOXICITY | -0.0983 | -0.3936 | 1 | 0.9993 |
|  | CYTOKINE_CYTOKINE_RECEPTOR_INTERACTION | -0.1747 | -0.7568 | 0.9665 | 1 |
|  | CYTOSOLIC_DNA_SENSING_PATHWAY | -0.2409 | -0.7476 | 0.8196 | 1 |
|  | ANTIGEN_PROCESSING_AND_PRESENTATION | -0.2478 | -0.74 | 0.7544 | 1 |
|  | FRUCTOSE_AND_MANNOSE_METABOLISM | -0.2665 | -0.7326 | 0.7652 | 1 |
|  | ALLOGRAFT_REJECTION | -0.2717 | -0.7267 | 0.8611 | 1 |
|  | PENTOSE_PHOSPHATE_PATHWAY | -0.2225 | -0.7165 | 0.8495 | 1 |
|  | ACUTE_MYELOID_LEUKEMIA | -0.1657 | -0.6896 | 0.9 | 1 |
|  | PATHOGENIC_ESCHERICHIA_COLI_INFECTION | -0.1685 | -0.6724 | 0.9263 | 1 |
|  | BASE_EXCISION_REPAIR | -0.2064 | -0.6679 | 0.8437 | 1 |
|  | LINOLEIC_ACID_METABOLISM | 0.1676 | 0.5052 | 1 | 1 |
|  | GLYCOSAMINOGLYCAN_DEGRADATION | 0.2212 | 0.7453 | 0.8038 | 1 |
|  | THYROID_CANCER | 0.2007 | 0.7476 | 0.832 | 1 |
|  | CHRONIC_MYELOID_LEUKEMIA | 0.152 | 0.7488 | 0.8 | 1 |
|  | STEROID_BIOSYNTHESIS | 0.2782 | 0.7677 | 0.7917 | 1 |
|  | LEISHMANIA_INFECTION | 0.208 | 0.7809 | 0.7476 | 1 |
|  | GLYCEROLIPID_METABOLISM | 0.2296 | 0.7818 | 0.8048 | 1 |
|  | GLYCOSPHINGOLIPID_BIOSYNTHESIS_GLOBO_SERIES | 0.2974 | 0.7852 | 0.75 | 1 |
| FUT8 | GLYCEROPHOSPHOLIPID_METABOLISM | 0.1921 | 0.786 | 0.8078 | 1 |
|  | RENIN_ANGIOTENSIN_SYSTEM | 0.2914 | 0.7934 | 0.7551 | 1 |
|  | ENDOMETRIAL_CANCER | 0.1802 | 0.7948 | 0.7803 | 1 |
|  | RETINOL_METABOLISM | 0.265 | 0.7955 | 0.8202 | 1 |
|  | CELL_ADHESION_MOLECULES_CAMS | 0.1928 | 0.8081 | 0.8776 | 1 |
|  | PARKINSONS_DISEASE | 0.1843 | 0.8128 | 0.6821 | 1 |
|  | FC_GAMMA_R_MEDIATED_PHAGOCYTOSIS | 0.1736 | 0.8211 | 0.7146 | 1 |
|  | ARGININE_AND_PROLINE_METABOLISM | 0.2284 | 0.8265 | 0.7265 | 1 |
|  | INSULIN_SIGNALING_PATHWAY | 0.1621 | 0.8516 | 0.7572 | 1 |
|  | HUNTINGTONS_DISEASE | 0.1743 | 0.8535 | 0.6389 | 1 |
|  | TAURINE_AND_HYPOTAURINE_METABOLISM | 0.3592 | 0.8596 | 0.628 | 1 |
|  | PYRUVATE_METABOLISM | 0.2294 | 0.8767 | 0.6444 | 1 |
|  | GLYCOSAMINOGLYCAN_BIOSYNTHESIS_CHONDROITIN_SULFATE | 0.2677 | 0.8786 | 0.5942 | 1 |
|  | METABOLISM_OF_XENOBIOTICS_BY_CYTOCHROME_P450 | 0.2695 | 0.8856 | 0.6667 | 1 |
|  | MTOR_SIGNALING_PATHWAY | 0.196 | 0.8872 | 0.64 | 1 |
|  | HYPERTROPHIC_CARDIOMYOPATHY_HCM | 0.2107 | 0.8935 | 0.6756 | 1 |
|  | GLUTATHIONE_METABOLISM | 0.267 | 0.8938 | 0.5965 | 1 |
|  | DILATED_CARDIOMYOPATHY | 0.2128 | 0.8952 | 0.6693 | 1 |
|  | PROXIMAL_TUBULE_BICARBONATE_RECLAMATION | 0.2888 | 0.9008 | 0.5854 | 1 |
|  | MAPK_SIGNALING_PATHWAY | 0.1741 | 0.9075 | 0.6157 | 1 |
|  | CARDIAC_MUSCLE_CONTRACTION | 0.2144 | 0.9139 | 0.616 | 1 |
|  | AXON_GUIDANCE | 0.1964 | 0.9185 | 0.6215 | 1 |
|  | TASTE_TRANSDUCTION | 0.3063 | 0.9231 | 0.592 | 1 |
|  | BLADDER_CANCER | 0.279 | 0.9279 | 0.5385 | 1 |
|  | DRUG_METABOLISM_CYTOCHROME_P450 | 0.2946 | 0.9335 | 0.5853 | 1 |
| FUT8 | COMPLEMENT_AND_COAGULATION_CASCADES | 0.2852 | 0.9355 | 0.5393 | 1 |
|  | REGULATION_OF_ACTIN_CYTOSKELETON | 0.1954 | 0.9399 | 0.5465 | 1 |
|  | FC_EPSILON_RI_SIGNALING_PATHWAY | 0.2098 | 0.9842 | 0.4776 | 1 |
|  | GLYCOLYSIS_GLUCONEOGENESIS | 0.2462 | 0.9879 | 0.4623 | 1 |
|  | WNT_SIGNALING_PATHWAY | 0.2043 | 0.991 | 0.4554 | 1 |
|  | PRION_DISEASES | 0.2811 | 1.0027 | 0.4503 | 1 |
|  | PPAR_SIGNALING_PATHWAY | 0.2714 | 1.0216 | 0.3878 | 1 |
|  | LONG_TERM_POTENTIATION | 0.2187 | 1.0235 | 0.4136 | 1 |
|  | ADIPOCYTOKINE_SIGNALING_PATHWAY | 0.2358 | 1.0242 | 0.4294 | 1 |
|  | VASCULAR_SMOOTH_MUSCLE_CONTRACTION | 0.2303 | 1.026 | 0.4056 | 1 |
|  | NON_SMALL_CELL_LUNG_CANCER | 0.2361 | 1.031 | 0.4101 | 1 |
|  | LYSOSOME | 0.2518 | 1.0332 | 0.4385 | 1 |
|  | BASAL_CELL_CARCINOMA | 0.2823 | 1.0347 | 0.4183 | 1 |
|  | PRIMARY_BILE_ACID_BIOSYNTHESIS | 0.4104 | 1.0525 | 0.4004 | 1 |
|  | CHEMOKINE_SIGNALING_PATHWAY | 0.2162 | 1.0551 | 0.3418 | 1 |
|  | ALDOSTERONE_REGULATED_SODIUM_REABSORPTION | 0.2869 | 1.0676 | 0.3819 | 1 |
|  | PANCREATIC_CANCER | 0.2274 | 1.0696 | 0.379 | 1 |
|  | NEUROTROPHIN_SIGNALING_PATHWAY | 0.2035 | 1.1047 | 0.3046 | 1 |
|  | TYROSINE_METABOLISM | 0.3259 | 1.1126 | 0.2471 | 1 |
|  | TIGHT_JUNCTION | 0.2414 | 1.1328 | 0.213 | 1 |
|  | MELANOGENESIS | 0.2536 | 1.134 | 0.2281 | 1 |
|  | SULFUR_METABOLISM | 0.4307 | 1.1417 | 0.2981 | 1 |
|  | RENAL_CELL_CARCINOMA | 0.2719 | 1.1567 | 0.2811 | 1 |
|  | CALCIUM_SIGNALING_PATHWAY | 0.2633 | 1.1574 | 0.2165 | 1 |
|  | TYPE_II_DIABETES_MELLITUS | 0.2986 | 1.1586 | 0.2449 | 1 |
| FUT8 | OLFACTORY_TRANSDUCTION | 0.3942 | 1.1675 | 0.26 | 1 |
|  | VIBRIO_CHOLERAE_INFECTION | 0.2554 | 1.1806 | 0.2409 | 1 |
|  | PHENYLALANINE_METABOLISM | 0.4698 | 1.2046 | 0.2288 | 1 |
|  | GLIOMA | 0.3026 | 1.2119 | 0.2109 | 1 |
|  | VEGF_SIGNALING_PATHWAY | 0.2619 | 1.2527 | 0.1252 | 1 |
|  | GALACTOSE_METABOLISM | 0.3505 | 1.2614 | 0.1929 | 1 |
|  | HEDGEHOG_SIGNALING_PATHWAY | 0.3419 | 1.2622 | 0.1534 | 1 |
|  | GNRH_SIGNALING_PATHWAY | 0.2782 | 1.271 | 0.1262 | 1 |
|  | NEUROACTIVE_LIGAND_RECEPTOR_INTERACTION | 0.3395 | 1.2777 | 0.0979 | 1 |
|  | MATURITY_ONSET_DIABETES_OF_THE_YOUNG | 0.4735 | 1.3386 | 0.1235 | 1 |
|  | LEUKOCYTE_TRANSENDOTHELIAL_MIGRATION | 0.2949 | 1.3492 | 0.0848 | 1 |
|  | OTHER_GLYCAN_DEGRADATION | 0.4329 | 1.3644 | 0.1487 | 1 |
| MAFG | OXIDATIVE_PHOSPHORYLATION | -0.3669 | -1.6207 | 0.0587 | 0.2 |
|  | CYTOSOLIC_DNA_SENSING_PATHWAY | -0.5096 | -1.5989 | 0.004 | 0.2102 |
|  | AMINOACYL_TRNA_BIOSYNTHESIS | -0.7274 | -1.5821 | 0.0124 | 0.2121 |
|  | ALLOGRAFT_REJECTION | -0.5949 | -1.6273 | 0.0195 | 0.2162 |
|  | MISMATCH_REPAIR | -0.6523 | -1.6397 | 0.0063 | 0.23 |
|  | RNA_POLYMERASE | -0.4938 | -1.6577 | 0.0261 | 0.2332 |
|  | CITRATE_CYCLE_TCA_CYCLE | -0.4312 | -1.5546 | 0.0651 | 0.2411 |
|  | PEROXISOME | -0.4593 | -1.5412 | 0.0142 | 0.2465 |
|  | PANTOTHENATE_AND_COA_BIOSYNTHESIS | -0.6365 | -1.4459 | 0.0592 | 0.2701 |
|  | GRAFT_VERSUS_HOST_DISEASE | -0.567 | -1.4305 | 0.1111 | 0.2748 |
|  | AMINO_SUGAR_AND_NUCLEOTIDE_SUGAR_METABOLISM | -0.4711 | -1.5179 | 0.012 | 0.2761 |
|  | BUTANOATE_METABOLISM | -0.4408 | -1.4461 | 0.0664 | 0.2828 |
|  | FRUCTOSE_AND_MANNOSE_METABOLISM | -0.4931 | -1.4306 | 0.1016 | 0.2867 |
| MAFG | DNA_REPLICATION | -0.6167 | -1.6594 | 0.0042 | 0.2877 |
|  | VALINE_LEUCINE_AND_ISOLEUCINE_DEGRADATION | -0.4986 | -1.4736 | 0.0474 | 0.2885 |
|  | PROTEASOME | -0.3712 | -1.4482 | 0.0935 | 0.2927 |
|  | VALINE_LEUCINE_AND_ISOLEUCINE_BIOSYNTHESIS | -0.7018 | -1.4103 | 0.0594 | 0.2958 |
|  | PROTEIN_EXPORT | -0.4475 | -1.4968 | 0.1218 | 0.2976 |
|  | BETA_ALANINE_METABOLISM | -0.5645 | -1.3966 | 0.0769 | 0.2977 |
|  | ALZHEIMERS_DISEASE | -0.2961 | -1.4775 | 0.0546 | 0.2989 |
|  | PYRIMIDINE_METABOLISM | -0.3418 | -1.4011 | 0.0558 | 0.3006 |
|  | ANTIGEN_PROCESSING_AND_PRESENTATION | -0.4867 | -1.4501 | 0.0933 | 0.3044 |
|  | HUNTINGTONS_DISEASE | -0.2752 | -1.386 | 0.126 | 0.3045 |
|  | PRIMARY_IMMUNODEFICIENCY | -0.458 | -1.4546 | 0.0867 | 0.312 |
|  | PARKINSONS_DISEASE | -0.3193 | -1.4789 | 0.101 | 0.3156 |
|  | LIMONENE_AND_PINENE_DEGRADATION | -0.6295 | -1.3686 | 0.114 | 0.3257 |
|  | NUCLEOTIDE_EXCISION_REPAIR | -0.5171 | -1.6694 | 0.0107 | 0.3453 |
|  | T_CELL_RECEPTOR_SIGNALING_PATHWAY | -0.2719 | -1.3208 | 0.1039 | 0.3767 |
|  | GLYOXYLATE_AND_DICARBOXYLATE_METABOLISM | -0.4287 | -1.3034 | 0.1732 | 0.3792 |
|  | SNARE_INTERACTIONS_IN_VESICULAR_TRANSPORT | -0.3518 | -1.2909 | 0.1565 | 0.3836 |
|  | PURINE_METABOLISM | -0.3006 | -1.3222 | 0.0646 | 0.3857 |
|  | INTESTINAL_IMMUNE_NETWORK_FOR_IGA_PRODUCTION | -0.4139 | -1.2943 | 0.1082 | 0.3862 |
|  | SELENOAMINO_ACID_METABOLISM | -0.3943 | -1.3042 | 0.1266 | 0.3884 |
|  | AUTOIMMUNE_THYROID_DISEASE | -0.4143 | -1.2828 | 0.1043 | 0.3889 |
|  | LYSINE_DEGRADATION | -0.3693 | -1.3267 | 0.1059 | 0.3897 |
|  | BASE_EXCISION_REPAIR | -0.3844 | -1.3317 | 0.1877 | 0.3914 |
|  | RIBOFLAVIN_METABOLISM | -0.5047 | -1.2752 | 0.1622 | 0.3948 |
|  | TYPE_I_DIABETES_MELLITUS | -0.4267 | -1.3054 | 0.134 | 0.397 |
| MAFG | ONE_CARBON_POOL_BY_FOLATE | -0.3916 | -1.2609 | 0.1667 | 0.4143 |
|  | CIRCADIAN_RHYTHM_MAMMAL | -0.4869 | -1.2444 | 0.2174 | 0.4386 |
|  | N_GLYCAN_BIOSYNTHESIS | -0.3059 | -1.2256 | 0.2163 | 0.4581 |
|  | HISTIDINE_METABOLISM | -0.3888 | -1.2268 | 0.1686 | 0.4665 |
|  | PROPANOATE_METABOLISM | -0.3633 | -1.1813 | 0.251 | 0.4821 |
|  | GLYCEROLIPID_METABOLISM | -0.3416 | -1.1934 | 0.2286 | 0.4852 |
|  | NATURAL_KILLER_CELL_MEDIATED_CYTOTOXICITY | -0.2966 | -1.1748 | 0.2767 | 0.486 |
|  | PENTOSE_PHOSPHATE_PATHWAY | -0.3698 | -1.2023 | 0.2471 | 0.4868 |
|  | COLORECTAL_CANCER | -0.2897 | -1.2057 | 0.2229 | 0.4898 |
|  | SPHINGOLIPID_METABOLISM | -0.3878 | -1.1818 | 0.248 | 0.4904 |
|  | VIRAL_MYOCARDITIS | -0.306 | -1.1935 | 0.1849 | 0.4952 |
|  | FATTY_ACID_METABOLISM | -0.3108 | -1.1836 | 0.172 | 0.4963 |
|  | RIBOSOME | -0.524 | -1.6721 | 0.0363 | 0.5035 |
|  | RNA_DEGRADATION | -0.3062 | -1.1538 | 0.3029 | 0.5213 |
|  | RIG_I_LIKE_RECEPTOR_SIGNALING_PATHWAY | -0.3306 | -1.1378 | 0.2573 | 0.5381 |
|  | STARCH_AND_SUCROSE_METABOLISM | -0.3696 | -1.14 | 0.2422 | 0.5428 |
|  | REGULATION_OF_AUTOPHAGY | -0.3587 | -1.1082 | 0.2901 | 0.5659 |
|  | ASTHMA | -0.4206 | -1.1095 | 0.3252 | 0.5729 |
|  | GLYCINE_SERINE_AND_THREONINE_METABOLISM | -0.3993 | -1.1125 | 0.286 | 0.5751 |
|  | UBIQUITIN_MEDIATED_PROTEOLYSIS | -0.2579 | -1.1137 | 0.3221 | 0.5832 |
|  | PYRUVATE_METABOLISM | -0.2819 | -1.0831 | 0.3378 | 0.6132 |
|  | ALANINE_ASPARTATE_AND_GLUTAMATE_METABOLISM | -0.3105 | -1.0773 | 0.3439 | 0.617 |
|  | PATHOGENIC_ESCHERICHIA_COLI_INFECTION | -0.2758 | -1.0656 | 0.3626 | 0.6344 |
|  | PHOSPHATIDYLINOSITOL_SIGNALING_SYSTEM | -0.2299 | -1.0591 | 0.3482 | 0.6398 |
|  | BIOSYNTHESIS_OF_UNSATURATED_FATTY_ACIDS | -0.3349 | -1.0535 | 0.3854 | 0.6432 |
| MAFG | TERPENOID_BACKBONE_BIOSYNTHESIS | -0.3139 | -1.0328 | 0.4324 | 0.6817 |
|  | HEMATOPOIETIC_CELL_LINEAGE | -0.2797 | -1.0262 | 0.4232 | 0.687 |
|  | B_CELL_RECEPTOR_SIGNALING_PATHWAY | -0.2123 | -1.0121 | 0.4331 | 0.6977 |
|  | ENDOCYTOSIS | -0.2075 | -1.0072 | 0.4366 | 0.6993 |
|  | GLYCOSYLPHOSPHATIDYLINOSITOL_GPI_ANCHOR_BIOSYNTHESIS | -0.3556 | -1.0141 | 0.4641 | 0.704 |
|  | INOSITOL_PHOSPHATE_METABOLISM | -0.2566 | -0.9911 | 0.476 | 0.7259 |
|  | CARDIAC_MUSCLE_CONTRACTION | -0.2347 | -0.9862 | 0.4814 | 0.7268 |
|  | ABC_TRANSPORTERS | -0.2929 | -0.9676 | 0.5207 | 0.7496 |
|  | LEISHMANIA_INFECTION | -0.2547 | -0.9625 | 0.4805 | 0.7517 |
|  | ARGININE_AND_PROLINE_METABOLISM | -0.2747 | -0.9693 | 0.4849 | 0.7559 |
|  | CELL_CYCLE | -0.2275 | -0.9544 | 0.501 | 0.76 |
|  | JAK_STAT_SIGNALING_PATHWAY | -0.2204 | -0.9469 | 0.5623 | 0.7671 |
|  | TRYPTOPHAN_METABOLISM | -0.2828 | -0.906 | 0.6353 | 0.7839 |
|  | GLUTATHIONE_METABOLISM | -0.2773 | -0.926 | 0.557 | 0.7856 |
|  | SYSTEMIC_LUPUS_ERYTHEMATOSUS | -0.285 | -0.9003 | 0.6015 | 0.787 |
|  | GLYCEROPHOSPHOLIPID_METABOLISM | -0.2232 | -0.9168 | 0.592 | 0.7882 |
|  | GLYCOLYSIS_GLUCONEOGENESIS | -0.2292 | -0.9121 | 0.5625 | 0.7892 |
|  | EPITHELIAL_CELL_SIGNALING_IN_HELICOBACTER_PYLORI_INFECTION | -0.2073 | -0.9076 | 0.5598 | 0.7896 |
|  | GLYCOSPHINGOLIPID_BIOSYNTHESIS_GANGLIO_SERIES | -0.3181 | -0.9322 | 0.536 | 0.7912 |
|  | TOLL_LIKE_RECEPTOR_SIGNALING_PATHWAY | -0.2247 | -0.9195 | 0.5606 | 0.7919 |
|  | AMYOTROPHIC_LATERAL_SCLEROSIS_ALS | -0.219 | -0.9261 | 0.5903 | 0.7956 |
|  | DILATED_CARDIOMYOPATHY | 0.2391 | 0.9803 | 0.496 | 0.7967 |
|  | APOPTOSIS | -0.1931 | -0.8817 | 0.6632 | 0.7988 |
|  | PHENYLALANINE_METABOLISM | -0.3356 | -0.8823 | 0.6248 | 0.8065 |
|  | GLYCOSAMINOGLYCAN_BIOSYNTHESIS_HEPARAN_SULFATE | -0.2921 | -0.8861 | 0.6278 | 0.808 |
| MAFG | PATHWAYS_IN_CANCER | 0.1926 | 0.9806 | 0.4912 | 0.8144 |
|  | NON_HOMOLOGOUS_END_JOINING | -0.3304 | -0.8704 | 0.6756 | 0.8149 |
|  | CYTOKINE_CYTOKINE_RECEPTOR_INTERACTION | -0.2021 | -0.8658 | 0.7958 | 0.815 |
|  | VASCULAR_SMOOTH_MUSCLE_CONTRACTION | 0.2292 | 1.0161 | 0.4061 | 0.817 |
|  | AXON_GUIDANCE | 0.2148 | 0.9916 | 0.4475 | 0.8229 |
|  | TIGHT_JUNCTION | 0.218 | 1.0214 | 0.4094 | 0.8233 |
|  | PROSTATE_CANCER | 0.2096 | 0.9986 | 0.4538 | 0.8242 |
|  | PROXIMAL_TUBULE_BICARBONATE_RECLAMATION | 0.2733 | 0.8551 | 0.6687 | 0.8244 |
|  | GALACTOSE_METABOLISM | 0.2645 | 0.9832 | 0.4675 | 0.8264 |
|  | SMALL_CELL_LUNG_CANCER | 0.1985 | 0.856 | 0.742 | 0.8362 |
|  | PROGESTERONE_MEDIATED_OOCYTE_MATURATION | -0.1926 | -0.8452 | 0.7316 | 0.8372 |
|  | STEROID_HORMONE_BIOSYNTHESIS | 0.328 | 1.0009 | 0.4617 | 0.8379 |
|  | TAURINE_AND_HYPOTAURINE_METABOLISM | -0.3522 | -0.8391 | 0.6714 | 0.8402 |
|  | CYSTEINE_AND_METHIONINE_METABOLISM | -0.245 | -0.8482 | 0.7078 | 0.8408 |
|  | GLYCOSPHINGOLIPID_BIOSYNTHESIS_GLOBO_SERIES | 0.3284 | 0.8597 | 0.6572 | 0.843 |
|  | PRION_DISEASES | 0.2833 | 1.022 | 0.4051 | 0.8434 |
|  | P53_SIGNALING_PATHWAY | 0.289 | 1.0299 | 0.4028 | 0.8443 |
|  | TYROSINE_METABOLISM | -0.2452 | -0.8317 | 0.7906 | 0.8454 |
|  | SPLICEOSOME | -0.3665 | -1.6894 | 0.0146 | 0.8479 |
|  | HOMOLOGOUS_RECOMBINATION | 0.2666 | 0.8297 | 0.7113 | 0.848 |
|  | ENDOMETRIAL_CANCER | 0.2194 | 0.9532 | 0.5049 | 0.8486 |
|  | DRUG_METABOLISM_CYTOCHROME_P450 | 0.3269 | 1.0361 | 0.3875 | 0.8497 |
|  | BLADDER_CANCER | 0.2529 | 0.8628 | 0.6351 | 0.8506 |
|  | ERBB_SIGNALING_PATHWAY | 0.2023 | 0.8805 | 0.6414 | 0.8569 |
|  | CELL_ADHESION_MOLECULES_CAMS | 0.1973 | 0.832 | 0.8327 | 0.8571 |
| MAFG | PPAR_SIGNALING_PATHWAY | 0.2313 | 0.872 | 0.7181 | 0.8604 |
|  | METABOLISM_OF_XENOBIOTICS_BY_CYTOCHROME_P450 | 0.2637 | 0.8637 | 0.7095 | 0.8632 |
|  | HYPERTROPHIC_CARDIOMYOPATHY_HCM | 0.2228 | 0.9332 | 0.5988 | 0.8636 |
|  | FC_GAMMA_R_MEDIATED_PHAGOCYTOSIS | 0.1949 | 0.9046 | 0.5762 | 0.8646 |
|  | SULFUR_METABOLISM | 0.3311 | 0.8805 | 0.5973 | 0.8724 |
|  | FOCAL_ADHESION | 0.215 | 1.0365 | 0.3628 | 0.8733 |
|  | GLYCOSPHINGOLIPID_BIOSYNTHESIS_LACTO_AND_NEOLACTO_SERIES | -0.2619 | -0.8112 | 0.8083 | 0.8747 |
|  | STEROID_BIOSYNTHESIS | 0.3289 | 0.9072 | 0.5951 | 0.8751 |
|  | NON_SMALL_CELL_LUNG_CANCER | 0.2197 | 0.9346 | 0.5469 | 0.878 |
|  | BASAL_CELL_CARCINOMA | 0.2422 | 0.8922 | 0.6398 | 0.8782 |
|  | MAPK_SIGNALING_PATHWAY | 0.17 | 0.8843 | 0.7097 | 0.8801 |
|  | LYSOSOME | -0.1944 | -0.8029 | 0.7134 | 0.881 |
|  | VASOPRESSIN_REGULATED_WATER_REABSORPTION | 0.2269 | 0.9168 | 0.6118 | 0.8868 |
|  | GAP_JUNCTION | 0.2292 | 0.908 | 0.603 | 0.8906 |
|  | GLYCOSAMINOGLYCAN_BIOSYNTHESIS_CHONDROITIN_SULFATE | 0.3247 | 1.0367 | 0.3826 | 0.8993 |
|  | INSULIN_SIGNALING_PATHWAY | 0.2009 | 1.0581 | 0.364 | 0.9132 |
|  | ARACHIDONIC_ACID_METABOLISM | -0.2318 | -0.7772 | 0.8693 | 0.9158 |
|  | TYPE_II_DIABETES_MELLITUS | 0.2679 | 1.0378 | 0.404 | 0.9238 |
|  | MELANOMA | 0.2701 | 1.0635 | 0.3757 | 0.9249 |
|  | NOD_LIKE_RECEPTOR_SIGNALING_PATHWAY | -0.203 | -0.7612 | 0.7596 | 0.9319 |
|  | ADHERENS_JUNCTION | 0.1811 | 0.7714 | 0.8264 | 0.9342 |
|  | ECM_RECEPTOR_INTERACTION | 0.286 | 1.0436 | 0.3672 | 0.9342 |
|  | GLYCOSAMINOGLYCAN_BIOSYNTHESIS_KERATAN_SULFATE | -0.2621 | -0.7444 | 0.8519 | 0.9375 |
|  | ACUTE_MYELOID_LEUKEMIA | -0.1809 | -0.7475 | 0.8369 | 0.9427 |
|  | NICOTINATE_AND_NICOTINAMIDE_METABOLISM | -0.2099 | -0.7308 | 0.858 | 0.9462 |
| MAFG | LEUKOCYTE_TRANSENDOTHELIAL_MIGRATION | 0.2639 | 1.2142 | 0.1813 | 0.9563 |
|  | ADIPOCYTOKINE_SIGNALING_PATHWAY | 0.247 | 1.0638 | 0.364 | 0.9571 |
|  | ETHER_LIPID_METABOLISM | -0.2079 | -0.6886 | 0.9302 | 0.9572 |
|  | ALPHA_LINOLENIC_ACID_METABOLISM | -0.2399 | -0.6963 | 0.901 | 0.9591 |
|  | MTOR_SIGNALING_PATHWAY | 0.164 | 0.7318 | 0.8926 | 0.9627 |
|  | ARRHYTHMOGENIC_RIGHT_VENTRICULAR_CARDIOMYOPATHY_ARVC | 0.1852 | 0.7086 | 0.9724 | 0.9631 |
|  | PORPHYRIN_AND_CHLOROPHYLL_METABOLISM | -0.2268 | -0.6713 | 0.9387 | 0.9644 |
|  | DRUG_METABOLISM_OTHER_ENZYMES | -0.1921 | -0.6601 | 0.9841 | 0.9647 |
|  | CHRONIC_MYELOID_LEUKEMIA | 0.2391 | 1.1717 | 0.2624 | 0.9659 |
|  | O_GLYCAN_BIOSYNTHESIS | -0.1886 | -0.5955 | 0.9667 | 0.9675 |
|  | PRIMARY_BILE_ACID_BIOSYNTHESIS | 0.2635 | 0.6778 | 0.9152 | 0.9678 |
|  | PANCREATIC_CANCER | 0.2309 | 1.0707 | 0.3797 | 0.968 |
|  | OOCYTE_MEIOSIS | -0.1487 | -0.6964 | 0.9575 | 0.9683 |
|  | TGF_BETA_SIGNALING_PATHWAY | -0.1771 | -0.7046 | 0.9443 | 0.9687 |
|  | THYROID_CANCER | 0.1967 | 0.7357 | 0.8385 | 0.9719 |
|  | ASCORBATE_AND_ALDARATE_METABOLISM | 0.2818 | 0.713 | 0.9194 | 0.9721 |
|  | BASAL_TRANSCRIPTION_FACTORS | -0.165 | -0.5985 | 0.9391 | 0.9751 |
|  | LONG_TERM_DEPRESSION | -0.1541 | -0.6154 | 0.9881 | 0.9759 |
|  | NITROGEN_METABOLISM | -0.2247 | -0.6313 | 0.9544 | 0.9762 |
|  | COMPLEMENT_AND_COAGULATION_CASCADES | 0.1973 | 0.6466 | 0.9659 | 0.9792 |
|  | FOLATE_BIOSYNTHESIS | 0.2521 | 0.6781 | 0.8922 | 0.9814 |
|  | TASTE_TRANSDUCTION | 0.4002 | 1.2202 | 0.167 | 0.996 |
|  | CHEMOKINE_SIGNALING_PATHWAY | 0.2193 | 1.0723 | 0.32 | 0.9985 |
|  | RENIN_ANGIOTENSIN_SYSTEM | 0.1421 | 0.3801 | 1 | 0.9996 |
|  | PENTOSE_AND_GLUCURONATE_INTERCONVERSIONS | 0.1886 | 0.487 | 1 | 1 |
| MAFG | GLYCOSAMINOGLYCAN_DEGRADATION | 0.1568 | 0.5257 | 0.9917 | 1 |
|  | WNT_SIGNALING_PATHWAY | 0.2214 | 1.0731 | 0.348 | 1 |
|  | OTHER_GLYCAN_DEGRADATION | 0.342 | 1.0872 | 0.377 | 1 |
|  | LINOLEIC_ACID_METABOLISM | 0.3591 | 1.0883 | 0.3069 | 1 |
|  | HEDGEHOG_SIGNALING_PATHWAY | 0.2955 | 1.0895 | 0.32 | 1 |
|  | CALCIUM_SIGNALING_PATHWAY | 0.2499 | 1.1005 | 0.2996 | 1 |
|  | VIBRIO_CHOLERAE_INFECTION | 0.2407 | 1.1316 | 0.2945 | 1 |
|  | RETINOL_METABOLISM | 0.3758 | 1.1379 | 0.2557 | 1 |
|  | MELANOGENESIS | 0.2593 | 1.1742 | 0.1756 | 1 |
|  | LONG_TERM_POTENTIATION | 0.2512 | 1.1745 | 0.2371 | 1 |
|  | GNRH_SIGNALING_PATHWAY | 0.2683 | 1.2296 | 0.1776 | 1 |
|  | ALDOSTERONE_REGULATED_SODIUM_REABSORPTION | 0.3263 | 1.2311 | 0.204 | 1 |
|  | RENAL_CELL_CARCINOMA | 0.2944 | 1.2504 | 0.1703 | 1 |
|  | REGULATION_OF_ACTIN_CYTOSKELETON | 0.2541 | 1.2505 | 0.099 | 1 |
|  | FC_EPSILON_RI_SIGNALING_PATHWAY | 0.2731 | 1.2629 | 0.1275 | 1 |
|  | VEGF_SIGNALING_PATHWAY | 0.27 | 1.2735 | 0.1225 | 1 |
|  | NOTCH_SIGNALING_PATHWAY | 0.312 | 1.2861 | 0.177 | 1 |
|  | MATURITY_ONSET_DIABETES_OF_THE_YOUNG | 0.4583 | 1.3019 | 0.1401 | 1 |
|  | NEUROACTIVE_LIGAND_RECEPTOR_INTERACTION | 0.3525 | 1.3058 | 0.0898 | 1 |
|  | NEUROTROPHIN_SIGNALING_PATHWAY | 0.2436 | 1.3371 | 0.1149 | 1 |
|  | DORSO_VENTRAL_AXIS_FORMATION | 0.4754 | 1.4468 | 0.0862 | 1 |
|  | OLFACTORY_TRANSDUCTION | 0.4899 | 1.4691 | 0.0421 | 1 |
|  | GLIOMA | 0.3966 | 1.6157 | 0.0257 | 1 |
| MGST1 | NOD_LIKE_RECEPTOR_SIGNALING_PATHWAY | -0.4504 | -1.7044 | 0.01 | 0.1639 |
|  | BIOSYNTHESIS_OF_UNSATURATED_FATTY_ACIDS | -0.5591 | -1.7155 | 0.006 | 0.1878 |
| MGST1 | LEISHMANIA_INFECTION | -0.4481 | -1.7192 | 0.0179 | 0.2418 |
|  | TOLL_LIKE_RECEPTOR_SIGNALING_PATHWAY | -0.423 | -1.7516 | 0.0038 | 0.2643 |
|  | FC_GAMMA_R_MEDIATED_PHAGOCYTOSIS | -0.3431 | -1.5606 | 0.0449 | 0.275 |
|  | CITRATE_CYCLE_TCA_CYCLE | -0.4356 | -1.5684 | 0.05 | 0.2865 |
|  | SNARE_INTERACTIONS_IN_VESICULAR_TRANSPORT | -0.4187 | -1.5786 | 0.045 | 0.2914 |
|  | SPHINGOLIPID_METABOLISM | -0.5182 | -1.5872 | 0.0081 | 0.3045 |
|  | GLYCEROPHOSPHOLIPID_METABOLISM | -0.3918 | -1.6026 | 0.0057 | 0.3072 |
|  | LYSOSOME | -0.3795 | -1.6057 | 0.0238 | 0.3476 |
|  | ETHER_LIPID_METABOLISM | -0.5384 | -1.785 | 0.0039 | 0.3786 |
|  | PRIMARY_IMMUNODEFICIENCY | 0.5379 | 1.6949 | 0.0212 | 0.3849 |
|  | PANTOTHENATE_AND_COA_BIOSYNTHESIS | -0.6565 | -1.4858 | 0.0275 | 0.4004 |
|  | RIG_I_LIKE_RECEPTOR_SIGNALING_PATHWAY | -0.4241 | -1.4964 | 0.0193 | 0.4015 |
|  | BETA_ALANINE_METABOLISM | -0.5716 | -1.4247 | 0.0588 | 0.4633 |
|  | PEROXISOME | -0.4208 | -1.4262 | 0.0463 | 0.487 |
|  | GLUTATHIONE_METABOLISM | -0.4271 | -1.432 | 0.0547 | 0.501 |
|  | ENDOCYTOSIS | -0.2991 | -1.4423 | 0.0424 | 0.5037 |
|  | P53_SIGNALING_PATHWAY | -0.3763 | -1.3644 | 0.0592 | 0.6007 |
|  | UBIQUITIN_MEDIATED_PROTEOLYSIS | -0.3149 | -1.3713 | 0.119 | 0.6062 |
|  | INOSITOL_PHOSPHATE_METABOLISM | -0.3411 | -1.3064 | 0.0603 | 0.7185 |
|  | ERBB_SIGNALING_PATHWAY | -0.2943 | -1.2957 | 0.1209 | 0.7277 |
|  | O_GLYCAN_BIOSYNTHESIS | -0.4042 | -1.3084 | 0.1328 | 0.745 |
|  | CELL_CYCLE | -0.309 | -1.3161 | 0.1075 | 0.7504 |
|  | BLADDER_CANCER | -0.3818 | -1.2643 | 0.2012 | 0.7554 |
|  | DRUG_METABOLISM_OTHER_ENZYMES | -0.3553 | -1.2169 | 0.1557 | 0.7735 |
|  | ALZHEIMERS_DISEASE | -0.2527 | -1.2754 | 0.1805 | 0.7741 |
| MGST1 | GRAFT_VERSUS_HOST_DISEASE | -0.473 | -1.2084 | 0.2713 | 0.7796 |
|  | PATHOGENIC_ESCHERICHIA_COLI_INFECTION | -0.3073 | -1.1705 | 0.2782 | 0.7815 |
|  | GLYCOSAMINOGLYCAN_BIOSYNTHESIS_HEPARAN_SULFATE | -0.3968 | -1.2011 | 0.1972 | 0.7816 |
|  | VALINE_LEUCINE_AND_ISOLEUCINE_DEGRADATION | -0.424 | -1.2653 | 0.1742 | 0.7818 |
|  | TGF_BETA_SIGNALING_PATHWAY | -0.3065 | -1.2347 | 0.159 | 0.7826 |
|  | LIMONENE_AND_PINENE_DEGRADATION | -0.5538 | -1.2207 | 0.2469 | 0.7845 |
|  | CYTOKINE_CYTOKINE_RECEPTOR_INTERACTION | -0.287 | -1.2409 | 0.0996 | 0.7854 |
|  | SYSTEMIC_LUPUS_ERYTHEMATOSUS | -0.3939 | -1.2259 | 0.1885 | 0.7897 |
|  | N_GLYCAN_BIOSYNTHESIS | -0.2907 | -1.1622 | 0.2636 | 0.7903 |
|  | EPITHELIAL_CELL_SIGNALING_IN_HELICOBACTER_PYLORI_INFECTION | -0.2737 | -1.2471 | 0.1657 | 0.7904 |
|  | FRUCTOSE_AND_MANNOSE_METABOLISM | -0.407 | -1.1737 | 0.3253 | 0.7909 |
|  | ALLOGRAFT_REJECTION | -0.4339 | -1.1853 | 0.244 | 0.7937 |
|  | ACUTE_MYELOID_LEUKEMIA | -0.2817 | -1.1783 | 0.2394 | 0.796 |
|  | PPAR_SIGNALING_PATHWAY | -0.3212 | -1.1898 | 0.1756 | 0.8004 |
|  | MAPK_SIGNALING_PATHWAY | -0.2171 | -1.1144 | 0.2286 | 0.8036 |
|  | GLYCOSYLPHOSPHATIDYLINOSITOL_GPI_ANCHOR_BIOSYNTHESIS | -0.3986 | -1.1272 | 0.3503 | 0.8121 |
|  | JAK_STAT_SIGNALING_PATHWAY | -0.2648 | -1.1499 | 0.178 | 0.8126 |
|  | INTESTINAL_IMMUNE_NETWORK_FOR_IGA_PRODUCTION | -0.3561 | -1.1154 | 0.2968 | 0.8172 |
|  | SMALL_CELL_LUNG_CANCER | -0.2558 | -1.1196 | 0.2481 | 0.821 |
|  | ALPHA_LINOLENIC_ACID_METABOLISM | -0.3987 | -1.142 | 0.2773 | 0.8213 |
|  | ABC_TRANSPORTERS | -0.3215 | -1.0975 | 0.2782 | 0.8241 |
|  | AMINO_SUGAR_AND_NUCLEOTIDE_SUGAR_METABOLISM | -0.3358 | -1.1024 | 0.3593 | 0.8262 |
|  | AUTOIMMUNE_THYROID_DISEASE | -0.3577 | -1.1278 | 0.2718 | 0.8288 |
|  | APOPTOSIS | -0.2434 | -1.1324 | 0.2398 | 0.8343 |
|  | GLYCOSAMINOGLYCAN_DEGRADATION | -0.3008 | -0.9814 | 0.4565 | 0.8397 |
| MGST1 | LINOLEIC_ACID_METABOLISM | -0.332 | -0.9762 | 0.5031 | 0.843 |
|  | GLYCOSPHINGOLIPID_BIOSYNTHESIS_LACTO_AND_NEOLACTO_SERIES | -0.3226 | -0.9828 | 0.4848 | 0.8477 |
|  | REGULATION_OF_AUTOPHAGY | -0.3158 | -0.9865 | 0.472 | 0.8494 |
|  | COLORECTAL_CANCER | -0.2355 | -0.999 | 0.4686 | 0.8507 |
|  | ARRHYTHMOGENIC_RIGHT_VENTRICULAR_CARDIOMYOPATHY_ARVC | -0.2542 | -0.969 | 0.5168 | 0.8511 |
|  | LYSINE_DEGRADATION | -0.2782 | -0.9933 | 0.499 | 0.8541 |
|  | PHOSPHATIDYLINOSITOL_SIGNALING_SYSTEM | -0.2184 | -1.002 | 0.4349 | 0.855 |
|  | VALINE_LEUCINE_AND_ISOLEUCINE_BIOSYNTHESIS | -0.5195 | -1.0614 | 0.4545 | 0.8558 |
|  | ADHERENS_JUNCTION | -0.2303 | -0.9883 | 0.4568 | 0.8562 |
|  | GLYCEROLIPID_METABOLISM | -0.2797 | -0.9617 | 0.5184 | 0.8592 |
|  | CHEMOKINE_SIGNALING_PATHWAY | -0.2115 | -1.0229 | 0.3969 | 0.8597 |
|  | RENIN_ANGIOTENSIN_SYSTEM | -0.3813 | -1.0039 | 0.466 | 0.8627 |
|  | HISTIDINE_METABOLISM | -0.3332 | -1.0451 | 0.369 | 0.8629 |
|  | NUCLEOTIDE_EXCISION_REPAIR | -0.2684 | -0.8717 | 0.6113 | 0.8636 |
|  | CELL_ADHESION_MOLECULES_CAMS | -0.2065 | -0.8639 | 0.75 | 0.8638 |
|  | MTOR_SIGNALING_PATHWAY | -0.1929 | -0.8597 | 0.6823 | 0.8643 |
|  | COMPLEMENT_AND_COAGULATION_CASCADES | -0.3296 | -1.0636 | 0.3475 | 0.8644 |
|  | METABOLISM_OF_XENOBIOTICS_BY_CYTOCHROME_P450 | -0.2778 | -0.8988 | 0.6475 | 0.8648 |
|  | HEMATOPOIETIC_CELL_LINEAGE | -0.2789 | -1.0395 | 0.3963 | 0.8655 |
|  | PHENYLALANINE_METABOLISM | -0.4066 | -1.0483 | 0.4081 | 0.8668 |
|  | FC_EPSILON_RI_SIGNALING_PATHWAY | -0.2019 | -0.9418 | 0.534 | 0.8676 |
|  | ARACHIDONIC_ACID_METABOLISM | -0.2872 | -0.9459 | 0.5462 | 0.868 |
|  | MELANOMA | -0.2648 | -1.0336 | 0.3969 | 0.8686 |
|  | SELENOAMINO_ACID_METABOLISM | -0.3016 | -1.0062 | 0.4659 | 0.8686 |
|  | DORSO_VENTRAL_AXIS_FORMATION | -0.2807 | -0.8657 | 0.6296 | 0.8687 |
| MGST1 | CHRONIC_MYELOID_LEUKEMIA | -0.1884 | -0.9286 | 0.5521 | 0.8703 |
|  | HUNTINGTONS_DISEASE | -0.1715 | -0.8724 | 0.6213 | 0.8707 |
|  | PYRIMIDINE_METABOLISM | -0.2197 | -0.8995 | 0.624 | 0.8726 |
|  | AMYOTROPHIC_LATERAL_SCLEROSIS_ALS | -0.2142 | -0.9031 | 0.6238 | 0.873 |
|  | TYPE_I_DIABETES_MELLITUS | -0.336 | -1.023 | 0.4395 | 0.8733 |
|  | PENTOSE_PHOSPHATE_PATHWAY | -0.2834 | -0.9314 | 0.5346 | 0.8735 |
|  | PROTEIN_EXPORT | -0.2656 | -0.8828 | 0.5845 | 0.8741 |
|  | B_CELL_RECEPTOR_SIGNALING_PATHWAY | -0.1726 | -0.8309 | 0.7055 | 0.8748 |
|  | LONG_TERM_DEPRESSION | -0.2377 | -0.9344 | 0.5489 | 0.8762 |
|  | RENAL_CELL_CARCINOMA | -0.2533 | -1.0758 | 0.3745 | 0.8766 |
|  | AMINOACYL_TRNA_BIOSYNTHESIS | -0.4192 | -0.9179 | 0.6008 | 0.877 |
|  | PROPANOATE_METABOLISM | -0.2667 | -0.8777 | 0.6343 | 0.877 |
|  | PROGESTERONE_MEDIATED_OOCYTE_MATURATION | -0.2009 | -0.8854 | 0.6627 | 0.8772 |
|  | MISMATCH_REPAIR | -0.4153 | -1.0499 | 0.439 | 0.8774 |
|  | FOLATE_BIOSYNTHESIS | -0.3881 | -1.026 | 0.4235 | 0.8774 |
|  | GNRH_SIGNALING_PATHWAY | -0.1783 | -0.8336 | 0.7568 | 0.8774 |
|  | PANCREATIC_CANCER | -0.1888 | -0.8734 | 0.6613 | 0.8776 |
|  | PROSTATE_CANCER | -0.1975 | -0.9501 | 0.5227 | 0.8786 |
|  | OOCYTE_MEIOSIS | -0.1872 | -0.8888 | 0.6468 | 0.8787 |
|  | PATHWAYS_IN_CANCER | -0.2063 | -1.0642 | 0.3035 | 0.8788 |
|  | ASTHMA | -0.387 | -1.0117 | 0.4532 | 0.8788 |
|  | OXIDATIVE_PHOSPHORYLATION | -0.2143 | -0.9459 | 0.5122 | 0.8789 |
|  | CYTOSOLIC_DNA_SENSING_PATHWAY | -0.3137 | -1.0067 | 0.4325 | 0.8805 |
|  | GLYCOSPHINGOLIPID_BIOSYNTHESIS_GLOBO_SERIES | -0.3134 | -0.8232 | 0.7171 | 0.8816 |
|  | LEUKOCYTE_TRANSENDOTHELIAL_MIGRATION | -0.181 | -0.847 | 0.6979 | 0.8826 |
| MGST1 | CIRCADIAN_RHYTHM_MAMMAL | -0.3185 | -0.8352 | 0.6855 | 0.8826 |
|  | INSULIN_SIGNALING_PATHWAY | -0.1612 | -0.8429 | 0.756 | 0.8827 |
|  | GAP_JUNCTION | -0.269 | -1.0683 | 0.3113 | 0.8828 |
|  | REGULATION_OF_ACTIN_CYTOSKELETON | -0.1891 | -0.9031 | 0.6504 | 0.8828 |
|  | PORPHYRIN_AND_CHLOROPHYLL_METABOLISM | -0.309 | -0.9113 | 0.5782 | 0.8835 |
|  | STARCH_AND_SUCROSE_METABOLISM | -0.2601 | -0.8123 | 0.7917 | 0.8859 |
|  | RNA_DEGRADATION | -0.241 | -0.918 | 0.574 | 0.8871 |
|  | ADIPOCYTOKINE_SIGNALING_PATHWAY | -0.1889 | -0.8157 | 0.7647 | 0.8878 |
|  | RIBOFLAVIN_METABOLISM | -0.3619 | -0.9051 | 0.5907 | 0.888 |
|  | T_CELL_RECEPTOR_SIGNALING_PATHWAY | -0.1721 | -0.836 | 0.7296 | 0.8894 |
|  | BUTANOATE_METABOLISM | -0.2423 | -0.8056 | 0.7469 | 0.8902 |
|  | FOCAL_ADHESION | -0.1646 | -0.7913 | 0.8988 | 0.8911 |
|  | PROXIMAL_TUBULE_BICARBONATE_RECLAMATION | -0.2567 | -0.7958 | 0.773 | 0.8914 |
|  | TAURINE_AND_HYPOTAURINE_METABOLISM | -0.3344 | -0.7962 | 0.7036 | 0.8983 |
|  | VIRAL_MYOCARDITIS | -0.1953 | -0.769 | 0.8629 | 0.9116 |
|  | PARKINSONS_DISEASE | -0.168 | -0.7631 | 0.7291 | 0.9128 |
|  | VEGF_SIGNALING_PATHWAY | -0.164 | -0.7725 | 0.8682 | 0.9138 |
|  | NICOTINATE_AND_NICOTINAMIDE_METABOLISM | -0.2064 | -0.715 | 0.876 | 0.938 |
|  | GLYCINE_SERINE_AND_THREONINE_METABOLISM | -0.2625 | -0.7191 | 0.9002 | 0.941 |
|  | DNA_REPLICATION | -0.2712 | -0.7248 | 0.8088 | 0.9413 |
|  | NATURAL_KILLER_CELL_MEDIATED_CYTOTOXICITY | -0.1801 | -0.7253 | 0.7786 | 0.9485 |
|  | GALACTOSE_METABOLISM | -0.1966 | -0.7295 | 0.8363 | 0.951 |
|  | RIBOSOME | 0.1743 | 0.5556 | 0.8363 | 0.9817 |
|  | CYSTEINE_AND_METHIONINE_METABOLISM | -0.1657 | -0.5842 | 0.9811 | 0.9877 |
|  | CARDIAC_MUSCLE_CONTRACTION | -0.157 | -0.6529 | 0.9838 | 0.9892 |
| MGST1 | ANTIGEN_PROCESSING_AND_PRESENTATION | -0.2076 | -0.6284 | 0.8953 | 0.9897 |
|  | GLYCOSPHINGOLIPID_BIOSYNTHESIS_GANGLIO_SERIES | -0.206 | -0.5958 | 0.9609 | 0.9907 |
|  | NITROGEN_METABOLISM | -0.2263 | -0.6383 | 0.9601 | 0.9918 |
|  | GLYOXYLATE_AND_DICARBOXYLATE_METABOLISM | -0.2018 | -0.6083 | 0.9344 | 0.9929 |
|  | PRION_DISEASES | 0.1575 | 0.5631 | 0.9902 | 0.9976 |
|  | SPLICEOSOME | -0.0905 | -0.4196 | 0.9958 | 0.9986 |
|  | PROTEASOME | -0.1267 | -0.4839 | 0.9841 | 1 |
|  | PURINE_METABOLISM | 0.1345 | 0.5891 | 1 | 1 |
|  | BASAL_TRANSCRIPTION_FACTORS | 0.1751 | 0.6119 | 0.931 | 1 |
|  | NOTCH_SIGNALING_PATHWAY | 0.1499 | 0.6358 | 0.8885 | 1 |
|  | RNA_POLYMERASE | 0.2017 | 0.6504 | 0.88 | 1 |
|  | ALDOSTERONE_REGULATED_SODIUM_REABSORPTION | 0.181 | 0.6693 | 0.9293 | 1 |
|  | SULFUR_METABOLISM | 0.2639 | 0.6915 | 0.8389 | 1 |
|  | THYROID_CANCER | 0.1876 | 0.6919 | 0.8873 | 1 |
|  | ENDOMETRIAL_CANCER | 0.1617 | 0.7095 | 0.8876 | 1 |
|  | HYPERTROPHIC_CARDIOMYOPATHY_HCM | 0.1737 | 0.7235 | 0.9456 | 1 |
|  | GLYCOLYSIS_GLUCONEOGENESIS | 0.1802 | 0.7238 | 0.8765 | 1 |
|  | NON_SMALL_CELL_LUNG_CANCER | 0.1715 | 0.7301 | 0.8704 | 1 |
|  | TERPENOID_BACKBONE_BIOSYNTHESIS | 0.2243 | 0.7311 | 0.8185 | 1 |
|  | VIBRIO_CHOLERAE_INFECTION | 0.1562 | 0.7343 | 0.8414 | 1 |
|  | PRIMARY_BILE_ACID_BIOSYNTHESIS | 0.2954 | 0.7462 | 0.8517 | 1 |
|  | PENTOSE_AND_GLUCURONATE_INTERCONVERSIONS | 0.2993 | 0.7652 | 0.8107 | 1 |
|  | WNT_SIGNALING_PATHWAY | 0.1553 | 0.7723 | 0.9031 | 1 |
|  | VASCULAR_SMOOTH_MUSCLE_CONTRACTION | 0.1772 | 0.7762 | 0.8864 | 1 |
|  | TIGHT_JUNCTION | 0.1666 | 0.793 | 0.905 | 1 |
| MGST1 | NEUROTROPHIN_SIGNALING_PATHWAY | 0.1438 | 0.7961 | 0.7791 | 1 |
|  | DILATED_CARDIOMYOPATHY | 0.1911 | 0.7977 | 0.8728 | 1 |
|  | TYPE_II_DIABETES_MELLITUS | 0.2077 | 0.7992 | 0.7955 | 1 |
|  | ALANINE_ASPARTATE_AND_GLUTAMATE_METABOLISM | 0.2329 | 0.8017 | 0.75 | 1 |
|  | GLIOMA | 0.2058 | 0.825 | 0.6793 | 1 |
|  | AXON_GUIDANCE | 0.1757 | 0.8314 | 0.8263 | 1 |
|  | MELANOGENESIS | 0.1875 | 0.8478 | 0.7585 | 1 |
|  | GLYCOSAMINOGLYCAN_BIOSYNTHESIS_KERATAN_SULFATE | 0.2974 | 0.8637 | 0.6506 | 1 |
|  | VASOPRESSIN_REGULATED_WATER_REABSORPTION | 0.209 | 0.8672 | 0.6994 | 1 |
|  | ECM_RECEPTOR_INTERACTION | 0.2485 | 0.8983 | 0.6581 | 1 |
|  | OTHER_GLYCAN_DEGRADATION | 0.2862 | 0.9025 | 0.5656 | 1 |
|  | MATURITY_ONSET_DIABETES_OF_THE_YOUNG | 0.3406 | 0.9697 | 0.4851 | 1 |
|  | PYRUVATE_METABOLISM | 0.2532 | 0.9719 | 0.5151 | 1 |
|  | LONG_TERM_POTENTIATION | 0.2085 | 0.9749 | 0.4654 | 1 |
|  | DRUG_METABOLISM_CYTOCHROME_P450 | 0.3074 | 0.9808 | 0.4902 | 1 |
|  | STEROID_HORMONE_BIOSYNTHESIS | 0.3233 | 0.9822 | 0.493 | 1 |
|  | BASAL_CELL_CARCINOMA | 0.2654 | 1.0083 | 0.4311 | 1 |
|  | TYROSINE_METABOLISM | 0.2946 | 1.0093 | 0.448 | 1 |
|  | FATTY_ACID_METABOLISM | 0.2617 | 1.0177 | 0.4052 | 1 |
|  | NON_HOMOLOGOUS_END_JOINING | 0.3784 | 1.0341 | 0.4145 | 1 |
|  | ASCORBATE_AND_ALDARATE_METABOLISM | 0.4175 | 1.0355 | 0.3794 | 1 |
|  | ARGININE_AND_PROLINE_METABOLISM | 0.2928 | 1.0425 | 0.3886 | 1 |
|  | TASTE_TRANSDUCTION | 0.3461 | 1.0519 | 0.3789 | 1 |
|  | TRYPTOPHAN_METABOLISM | 0.3242 | 1.0618 | 0.3577 | 1 |
|  | NEUROACTIVE_LIGAND_RECEPTOR_INTERACTION | 0.2891 | 1.0778 | 0.3395 | 1 |
| MGST1 | BASE_EXCISION_REPAIR | 0.3321 | 1.0897 | 0.4088 | 1 |
|  | GLYCOSAMINOGLYCAN_BIOSYNTHESIS_CHONDROITIN_SULFATE | 0.3405 | 1.095 | 0.3326 | 1 |
|  | ONE_CARBON_POOL_BY_FOLATE | 0.3518 | 1.0951 | 0.355 | 1 |
|  | HOMOLOGOUS_RECOMBINATION | 0.3592 | 1.1035 | 0.3155 | 1 |
|  | CALCIUM_SIGNALING_PATHWAY | 0.2768 | 1.2339 | 0.1373 | 1 |
|  | RETINOL_METABOLISM | 0.4129 | 1.2572 | 0.1455 | 1 |
|  | STEROID_BIOSYNTHESIS | 0.4667 | 1.2676 | 0.1594 | 1 |
|  | HEDGEHOG_SIGNALING_PATHWAY | 0.3366 | 1.2874 | 0.1367 | 1 |
|  | OLFACTORY_TRANSDUCTION | 0.4778 | 1.4135 | 0.0242 | 1 |
| SDAD1 | SPLICEOSOME | -0.4704 | -2.1956 | 0 | 0.0016 |
|  | RIBOSOME | -0.6429 | -2.0795 | 0 | 0.004 |
|  | NUCLEOTIDE_EXCISION_REPAIR | -0.5729 | -1.8626 | 0 | 0.034 |
|  | PROTEIN_EXPORT | -0.5338 | -1.7707 | 0.0126 | 0.0736 |
|  | DNA_REPLICATION | -0.6513 | -1.747 | 0 | 0.0756 |
|  | RNA_DEGRADATION | -0.4401 | -1.7059 | 0.0142 | 0.0966 |
|  | MISMATCH_REPAIR | -0.6674 | -1.6828 | 0.004 | 0.1029 |
|  | N_GLYCAN_BIOSYNTHESIS | -0.4163 | -1.6399 | 0.0142 | 0.1036 |
|  | PROTEASOME | -0.4315 | -1.6647 | 0.0239 | 0.105 |
|  | PRIMARY_IMMUNODEFICIENCY | -0.5173 | -1.64 | 0.0403 | 0.1149 |
|  | T_CELL_RECEPTOR_SIGNALING_PATHWAY | -0.3154 | -1.5341 | 0.0327 | 0.1391 |
|  | PYRIMIDINE_METABOLISM | -0.3783 | -1.536 | 0.0102 | 0.1443 |
|  | UBIQUITIN_MEDIATED_PROTEOLYSIS | -0.3492 | -1.5392 | 0.0495 | 0.15 |
|  | RNA_POLYMERASE | -0.4785 | -1.5637 | 0.0342 | 0.1517 |
|  | VALINE_LEUCINE_AND_ISOLEUCINE_DEGRADATION | -0.5178 | -1.5441 | 0.0062 | 0.1541 |
|  | AMINOACYL_TRNA_BIOSYNTHESIS | -0.7154 | -1.5673 | 0.012 | 0.1599 |
| SDAD1 | PURINE_METABOLISM | -0.3513 | -1.5447 | 0.006 | 0.1646 |
|  | CELL_CYCLE | -0.3707 | -1.5722 | 0.0299 | 0.1673 |
|  | ONE_CARBON_POOL_BY_FOLATE | -0.4668 | -1.4702 | 0.0756 | 0.1992 |
|  | COLORECTAL_CANCER | -0.3422 | -1.4617 | 0.0655 | 0.2011 |
|  | BASAL_TRANSCRIPTION_FACTORS | -0.3982 | -1.4743 | 0.0794 | 0.2044 |
|  | TERPENOID_BACKBONE_BIOSYNTHESIS | -0.4398 | -1.4465 | 0.0862 | 0.211 |
|  | HOMOLOGOUS_RECOMBINATION | -0.4565 | -1.419 | 0.084 | 0.2418 |
|  | BLADDER_CANCER | 0.4984 | 1.6884 | 0.0295 | 0.2758 |
|  | PROPANOATE_METABOLISM | -0.4142 | -1.3757 | 0.0825 | 0.2788 |
|  | BUTANOATE_METABOLISM | -0.4122 | -1.3664 | 0.0821 | 0.2836 |
|  | CITRATE_CYCLE_TCA_CYCLE | -0.3767 | -1.3765 | 0.1369 | 0.2883 |
|  | BASE_EXCISION_REPAIR | -0.4109 | -1.3831 | 0.144 | 0.2894 |
|  | GLYCOSYLPHOSPHATIDYLINOSITOL_GPI_ANCHOR_BIOSYNTHESIS | -0.4642 | -1.3366 | 0.1694 | 0.2931 |
|  | VALINE_LEUCINE_AND_ISOLEUCINE_BIOSYNTHESIS | -0.6738 | -1.3462 | 0.1271 | 0.2977 |
|  | PEROXISOME | -0.3927 | -1.337 | 0.1138 | 0.3021 |
|  | LYSINE_DEGRADATION | -0.3718 | -1.3494 | 0.1085 | 0.3029 |
|  | ALLOGRAFT_REJECTION | -0.4732 | -1.3118 | 0.1295 | 0.3257 |
|  | GLIOMA | 0.4239 | 1.7088 | 0.0123 | 0.3359 |
|  | NOTCH_SIGNALING_PATHWAY | 0.4236 | 1.7726 | 0.0181 | 0.3465 |
|  | CIRCADIAN_RHYTHM_MAMMAL | -0.5099 | -1.2917 | 0.1554 | 0.3515 |
|  | SELENOAMINO_ACID_METABOLISM | -0.3813 | -1.2685 | 0.1578 | 0.3751 |
|  | PARKINSONS_DISEASE | -0.2747 | -1.2729 | 0.2224 | 0.3773 |
|  | OXIDATIVE_PHOSPHORYLATION | -0.2891 | -1.2606 | 0.2441 | 0.3795 |
|  | PANTOTHENATE_AND_COA_BIOSYNTHESIS | -0.5449 | -1.2432 | 0.2187 | 0.3921 |
|  | RIBOFLAVIN_METABOLISM | -0.4971 | -1.2481 | 0.1957 | 0.3932 |
| SDAD1 | AMINO_SUGAR_AND_NUCLEOTIDE_SUGAR_METABOLISM | -0.3827 | -1.2253 | 0.2285 | 0.4153 |
|  | OOCYTE_MEIOSIS | -0.2522 | -1.1919 | 0.1941 | 0.4581 |
|  | ANTIGEN_PROCESSING_AND_PRESENTATION | -0.4067 | -1.1926 | 0.2792 | 0.4683 |
|  | CHRONIC_MYELOID_LEUKEMIA | 0.3036 | 1.4925 | 0.0872 | 0.4764 |
|  | VIRAL_MYOCARDITIS | -0.2958 | -1.1613 | 0.2132 | 0.4978 |
|  | INTESTINAL_IMMUNE_NETWORK_FOR_IGA_PRODUCTION | -0.3714 | -1.1625 | 0.242 | 0.5069 |
|  | NEUROACTIVE_LIGAND_RECEPTOR_INTERACTION | 0.3883 | 1.4627 | 0.004 | 0.5155 |
|  | NEUROTROPHIN_SIGNALING_PATHWAY | 0.2416 | 1.3417 | 0.0867 | 0.5185 |
|  | SULFUR_METABOLISM | 0.5139 | 1.3477 | 0.132 | 0.5272 |
|  | PANCREATIC_CANCER | 0.3141 | 1.4953 | 0.0364 | 0.5353 |
|  | ADIPOCYTOKINE_SIGNALING_PATHWAY | 0.3104 | 1.3646 | 0.0871 | 0.5381 |
|  | DORSO_VENTRAL_AXIS_FORMATION | 0.4618 | 1.3749 | 0.088 | 0.5397 |
|  | NON_SMALL_CELL_LUNG_CANCER | 0.3146 | 1.3531 | 0.1211 | 0.5416 |
|  | TYPE_II_DIABETES_MELLITUS | 0.3635 | 1.3782 | 0.1045 | 0.5674 |
|  | CHEMOKINE_SIGNALING_PATHWAY | 0.2593 | 1.2442 | 0.1294 | 0.5815 |
|  | MELANOMA | 0.3507 | 1.3856 | 0.0739 | 0.5826 |
|  | GLYCEROPHOSPHOLIPID_METABOLISM | 0.3068 | 1.2514 | 0.1426 | 0.5829 |
|  | REGULATION_OF_ACTIN_CYTOSKELETON | 0.2619 | 1.2645 | 0.1047 | 0.5917 |
|  | CYSTEINE_AND_METHIONINE_METABOLISM | -0.3173 | -1.1141 | 0.2804 | 0.5918 |
|  | CALCIUM_SIGNALING_PATHWAY | 0.2932 | 1.2985 | 0.0734 | 0.5926 |
|  | MATURITY_ONSET_DIABETES_OF_THE_YOUNG | 0.4387 | 1.2556 | 0.1744 | 0.5929 |
|  | VASCULAR_SMOOTH_MUSCLE_CONTRACTION | 0.2782 | 1.2313 | 0.1247 | 0.5956 |
|  | OTHER_GLYCAN_DEGRADATION | 0.4102 | 1.2872 | 0.2044 | 0.6009 |
|  | FC_EPSILON_RI_SIGNALING_PATHWAY | 0.2744 | 1.2771 | 0.1451 | 0.6032 |
|  | FC_GAMMA_R_MEDIATED_PHAGOCYTOSIS | 0.3006 | 1.3915 | 0.0882 | 0.6116 |
| SDAD1 | INSULIN_SIGNALING_PATHWAY | 0.2392 | 1.265 | 0.1274 | 0.6151 |
|  | MAPK_SIGNALING_PATHWAY | 0.2517 | 1.3006 | 0.0853 | 0.6161 |
|  | VEGF_SIGNALING_PATHWAY | 0.3186 | 1.4958 | 0.0265 | 0.6224 |
|  | SPHINGOLIPID_METABOLISM | -0.3564 | -1.0879 | 0.3702 | 0.6263 |
|  | LINOLEIC_ACID_METABOLISM | 0.4005 | 1.198 | 0.191 | 0.6291 |
|  | ARACHIDONIC_ACID_METABOLISM | 0.3577 | 1.2123 | 0.1647 | 0.6306 |
|  | PYRUVATE_METABOLISM | -0.2744 | -1.0622 | 0.362 | 0.634 |
|  | ALANINE_ASPARTATE_AND_GLUTAMATE_METABOLISM | -0.3176 | -1.0896 | 0.292 | 0.6365 |
|  | GRAFT_VERSUS_HOST_DISEASE | -0.4287 | -1.0652 | 0.4128 | 0.6391 |
|  | LYSOSOME | 0.2882 | 1.2011 | 0.2432 | 0.6409 |
|  | RENAL_CELL_CARCINOMA | 0.3288 | 1.3976 | 0.0885 | 0.6422 |
|  | HUNTINGTONS_DISEASE | -0.2123 | -1.0694 | 0.3677 | 0.6425 |
|  | SMALL_CELL_LUNG_CANCER | -0.2445 | -1.0514 | 0.3674 | 0.645 |
|  | GALACTOSE_METABOLISM | 0.3205 | 1.1628 | 0.2608 | 0.6525 |
|  | GLYOXYLATE_AND_DICARBOXYLATE_METABOLISM | -0.3683 | -1.0695 | 0.3791 | 0.6558 |
|  | PROSTATE_CANCER | 0.2359 | 1.1411 | 0.2526 | 0.6593 |
|  | AXON_GUIDANCE | 0.2552 | 1.1802 | 0.1677 | 0.6607 |
|  | PHENYLALANINE_METABOLISM | 0.4452 | 1.164 | 0.2606 | 0.6678 |
|  | LIMONENE_AND_PINENE_DEGRADATION | -0.4625 | -1.0189 | 0.4667 | 0.6699 |
|  | REGULATION_OF_AUTOPHAGY | -0.3247 | -1.0079 | 0.4431 | 0.6701 |
|  | ALZHEIMERS_DISEASE | -0.2005 | -1.0297 | 0.4127 | 0.6704 |
|  | PROGESTERONE_MEDIATED_OOCYTE_MATURATION | -0.2336 | -1.0237 | 0.4128 | 0.6712 |
|  | EPITHELIAL_CELL_SIGNALING_IN_HELICOBACTER_PYLORI_INFECTION | 0.2546 | 1.1418 | 0.2629 | 0.6749 |
|  | FATTY_ACID_METABOLISM | -0.2668 | -1.0332 | 0.4064 | 0.6753 |
|  | LEUKOCYTE_TRANSENDOTHELIAL_MIGRATION | 0.333 | 1.5411 | 0.025 | 0.6772 |
| SDAD1 | BETA_ALANINE_METABOLISM | -0.4039 | -1.0095 | 0.4748 | 0.6784 |
|  | DILATED_CARDIOMYOPATHY | 0.2655 | 1.1045 | 0.2759 | 0.6791 |
|  | BIOSYNTHESIS_OF_UNSATURATED_FATTY_ACIDS | -0.3306 | -0.9983 | 0.4699 | 0.6801 |
|  | VIBRIO_CHOLERAE_INFECTION | 0.2907 | 1.4037 | 0.0785 | 0.6823 |
|  | FOCAL_ADHESION | 0.2382 | 1.1454 | 0.2077 | 0.6831 |
|  | ALDOSTERONE_REGULATED_SODIUM_REABSORPTION | 0.2852 | 1.0625 | 0.351 | 0.6848 |
|  | GLYCOSAMINOGLYCAN_BIOSYNTHESIS_KERATAN_SULFATE | -0.3474 | -0.9851 | 0.4871 | 0.6857 |
|  | JAK_STAT_SIGNALING_PATHWAY | -0.229 | -0.99 | 0.496 | 0.6868 |
|  | PATHWAYS_IN_CANCER | 0.2251 | 1.1642 | 0.1644 | 0.6869 |
|  | PRION_DISEASES | 0.3048 | 1.089 | 0.3268 | 0.6902 |
|  | HEDGEHOG_SIGNALING_PATHWAY | 0.2801 | 1.0445 | 0.3845 | 0.6903 |
|  | GLYCOSAMINOGLYCAN_BIOSYNTHESIS_CHONDROITIN_SULFATE | 0.3526 | 1.105 | 0.3112 | 0.6938 |
|  | HYPERTROPHIC_CARDIOMYOPATHY_HCM | 0.2531 | 1.0635 | 0.348 | 0.696 |
|  | ENDOMETRIAL_CANCER | 0.2439 | 1.0915 | 0.336 | 0.6982 |
|  | BASAL_CELL_CARCINOMA | 0.2804 | 1.0454 | 0.3728 | 0.7012 |
|  | TIGHT_JUNCTION | 0.2326 | 1.1082 | 0.2387 | 0.702 |
|  | ACUTE_MYELOID_LEUKEMIA | 0.2478 | 1.0348 | 0.4289 | 0.7025 |
|  | MELANOGENESIS | 0.235 | 1.0664 | 0.3235 | 0.7029 |
|  | GNRH_SIGNALING_PATHWAY | 0.3231 | 1.5044 | 0.0196 | 0.7034 |
|  | TASTE_TRANSDUCTION | 0.333 | 1.0196 | 0.4361 | 0.7039 |
|  | PHOSPHATIDYLINOSITOL_SIGNALING_SYSTEM | 0.2232 | 1.0237 | 0.3921 | 0.7054 |
|  | ENDOCYTOSIS | 0.2217 | 1.0707 | 0.332 | 0.7065 |
|  | COMPLEMENT_AND_COAGULATION_CASCADES | 0.344 | 1.1176 | 0.2802 | 0.7089 |
|  | TYROSINE_METABOLISM | 0.3055 | 1.0475 | 0.3659 | 0.7093 |
|  | NOD_LIKE_RECEPTOR_SIGNALING_PATHWAY | 0.2758 | 1.027 | 0.4148 | 0.7095 |
| SDAD1 | PPAR_SIGNALING_PATHWAY | 0.2966 | 1.1108 | 0.2808 | 0.7112 |
|  | OLFACTORY_TRANSDUCTION | 0.3623 | 1.0736 | 0.3547 | 0.7138 |
|  | PRIMARY_BILE_ACID_BIOSYNTHESIS | 0.393 | 1.0023 | 0.4728 | 0.722 |
|  | DRUG_METABOLISM_CYTOCHROME_P450 | 0.3147 | 0.9928 | 0.4789 | 0.7323 |
|  | TAURINE_AND_HYPOTAURINE_METABOLISM | 0.4176 | 1.0028 | 0.4682 | 0.733 |
|  | ECM_RECEPTOR_INTERACTION | 0.2715 | 0.9873 | 0.4783 | 0.7344 |
|  | APOPTOSIS | 0.2093 | 0.9814 | 0.4819 | 0.7362 |
|  | NON_HOMOLOGOUS_END_JOINING | -0.3571 | -0.9539 | 0.5281 | 0.7428 |
|  | LONG_TERM_POTENTIATION | 0.2104 | 0.9662 | 0.5086 | 0.7606 |
|  | GAP_JUNCTION | 0.2428 | 0.96 | 0.504 | 0.7637 |
|  | ASCORBATE_AND_ALDARATE_METABOLISM | -0.379 | -0.9365 | 0.5431 | 0.7701 |
|  | GLYCOSPHINGOLIPID_BIOSYNTHESIS_GLOBO_SERIES | 0.3355 | 0.8993 | 0.5861 | 0.7709 |
|  | ADHERENS_JUNCTION | 0.2102 | 0.903 | 0.6325 | 0.7731 |
|  | O_GLYCAN_BIOSYNTHESIS | 0.284 | 0.9109 | 0.5827 | 0.7753 |
|  | ERBB_SIGNALING_PATHWAY | 0.2071 | 0.9055 | 0.6235 | 0.7777 |
|  | RENIN_ANGIOTENSIN_SYSTEM | 0.3413 | 0.9114 | 0.5583 | 0.7851 |
|  | GLYCOSAMINOGLYCAN_DEGRADATION | 0.2767 | 0.9115 | 0.5462 | 0.7957 |
|  | TYPE_I_DIABETES_MELLITUS | -0.2799 | -0.8583 | 0.6911 | 0.7995 |
|  | P53_SIGNALING_PATHWAY | -0.2406 | -0.8622 | 0.6964 | 0.8025 |
|  | TRYPTOPHAN_METABOLISM | 0.2771 | 0.9125 | 0.6409 | 0.8048 |
|  | HEMATOPOIETIC_CELL_LINEAGE | 0.2472 | 0.9157 | 0.5902 | 0.8091 |
|  | B_CELL_RECEPTOR_SIGNALING_PATHWAY | -0.1801 | -0.8694 | 0.6593 | 0.8111 |
|  | ABC_TRANSPORTERS | -0.2583 | -0.8632 | 0.7099 | 0.8117 |
|  | AUTOIMMUNE_THYROID_DISEASE | -0.2821 | -0.9012 | 0.6541 | 0.8141 |
|  | MTOR_SIGNALING_PATHWAY | 0.2054 | 0.9173 | 0.5693 | 0.8172 |
| SDAD1 | ALPHA_LINOLENIC_ACID_METABOLISM | 0.3183 | 0.9328 | 0.5385 | 0.8174 |
|  | PENTOSE_AND_GLUCURONATE_INTERCONVERSIONS | -0.3549 | -0.905 | 0.5866 | 0.8179 |
|  | AMYOTROPHIC_LATERAL_SCLEROSIS_ALS | -0.2082 | -0.871 | 0.6736 | 0.8197 |
|  | TOLL_LIKE_RECEPTOR_SIGNALING_PATHWAY | 0.2158 | 0.8713 | 0.6761 | 0.8208 |
|  | CELL_ADHESION_MOLECULES_CAMS | 0.2166 | 0.9247 | 0.6438 | 0.8244 |
|  | CYTOSOLIC_DNA_SENSING_PATHWAY | -0.2612 | -0.8393 | 0.6759 | 0.8255 |
|  | RETINOL_METABOLISM | 0.3038 | 0.9185 | 0.5897 | 0.8266 |
|  | FOLATE_BIOSYNTHESIS | -0.34 | -0.9069 | 0.5784 | 0.8267 |
|  | METABOLISM_OF_XENOBIOTICS_BY_CYTOCHROME_P450 | 0.2622 | 0.8472 | 0.7541 | 0.8279 |
|  | GLYCINE_SERINE_AND_THREONINE_METABOLISM | -0.3155 | -0.8725 | 0.6759 | 0.8282 |
|  | HISTIDINE_METABOLISM | 0.2689 | 0.8518 | 0.7103 | 0.8283 |
|  | GLYCOSAMINOGLYCAN_BIOSYNTHESIS_HEPARAN_SULFATE | -0.2907 | -0.8765 | 0.6528 | 0.832 |
|  | ETHER_LIPID_METABOLISM | 0.2584 | 0.8547 | 0.7134 | 0.833 |
|  | WNT_SIGNALING_PATHWAY | 0.1725 | 0.8593 | 0.7364 | 0.8349 |
|  | STARCH_AND_SUCROSE_METABOLISM | -0.2795 | -0.8855 | 0.6283 | 0.8366 |
|  | PENTOSE_PHOSPHATE_PATHWAY | -0.2662 | -0.8788 | 0.625 | 0.8392 |
|  | DRUG_METABOLISM_OTHER_ENZYMES | 0.2459 | 0.8228 | 0.8364 | 0.8556 |
|  | THYROID_CANCER | 0.2137 | 0.8098 | 0.714 | 0.859 |
|  | LEISHMANIA_INFECTION | 0.218 | 0.8262 | 0.699 | 0.8591 |
|  | LONG_TERM_DEPRESSION | 0.2059 | 0.8108 | 0.8283 | 0.8672 |
|  | TGF_BETA_SIGNALING_PATHWAY | -0.1991 | -0.8065 | 0.8291 | 0.8759 |
|  | ARGININE_AND_PROLINE_METABOLISM | 0.2177 | 0.7831 | 0.8184 | 0.8851 |
|  | CARDIAC_MUSCLE_CONTRACTION | 0.1874 | 0.7842 | 0.8397 | 0.8937 |
|  | INOSITOL_PHOSPHATE_METABOLISM | 0.1981 | 0.7601 | 0.9071 | 0.9005 |
|  | PROXIMAL_TUBULE_BICARBONATE_RECLAMATION | 0.2542 | 0.766 | 0.818 | 0.9019 |
| SDAD1 | SNARE_INTERACTIONS_IN_VESICULAR_TRANSPORT | -0.2083 | -0.7775 | 0.7495 | 0.9025 |
|  | GLYCOSPHINGOLIPID_BIOSYNTHESIS_GANGLIO_SERIES | -0.2634 | -0.7641 | 0.8064 | 0.9125 |
|  | NICOTINATE_AND_NICOTINAMIDE_METABOLISM | -0.226 | -0.7777 | 0.8168 | 0.9137 |
|  | GLYCOLYSIS_GLUCONEOGENESIS | 0.1862 | 0.7424 | 0.8286 | 0.9145 |
|  | GLYCEROLIPID_METABOLISM | 0.21 | 0.7262 | 0.841 | 0.9252 |
|  | ARRHYTHMOGENIC_RIGHT_VENTRICULAR_CARDIOMYOPATHY_ARVC | 0.1841 | 0.7018 | 0.9638 | 0.9332 |
|  | VASOPRESSIN_REGULATED_WATER_REABSORPTION | 0.1708 | 0.7107 | 0.9243 | 0.9337 |
|  | PATHOGENIC_ESCHERICHIA_COLI_INFECTION | 0.1737 | 0.6712 | 0.8821 | 0.9515 |
|  | PORPHYRIN_AND_CHLOROPHYLL_METABOLISM | 0.2191 | 0.643 | 0.9667 | 0.9629 |
|  | STEROID_BIOSYNTHESIS | -0.2294 | -0.6276 | 0.9583 | 0.9646 |
|  | GLUTATHIONE_METABOLISM | 0.1807 | 0.6205 | 0.9757 | 0.9666 |
|  | FRUCTOSE_AND_MANNOSE_METABOLISM | -0.2522 | -0.7146 | 0.8039 | 0.9701 |
|  | RIG_I_LIKE_RECEPTOR_SIGNALING_PATHWAY | -0.1821 | -0.6327 | 0.9861 | 0.973 |
|  | NATURAL_KILLER_CELL_MEDIATED_CYTOTOXICITY | -0.1652 | -0.6495 | 0.9053 | 0.9733 |
|  | STEROID_HORMONE_BIOSYNTHESIS | -0.1914 | -0.5784 | 0.9917 | 0.9755 |
|  | GLYCOSPHINGOLIPID_BIOSYNTHESIS_LACTO_AND_NEOLACTO_SERIES | -0.2174 | -0.6557 | 0.973 | 0.9803 |
|  | SYSTEMIC_LUPUS_ERYTHEMATOSUS | 0.1814 | 0.5635 | 0.9938 | 0.9805 |
|  | CYTOKINE_CYTOKINE_RECEPTOR_INTERACTION | -0.1607 | -0.6852 | 0.9859 | 0.9895 |
|  | ASTHMA | -0.2437 | -0.6566 | 0.9414 | 0.9912 |
|  | NITROGEN_METABOLISM | -0.2318 | -0.6614 | 0.9332 | 0.9992 |
