# Supplementary material for: Integrated bioinformatics and machine learning for constructing a diagnostic model of major depressive disorder leveraging shared signatures from hemodialysis: A cross-sectional study
Source: Medicine (Baltimore). 2026 Jun 5;105(23):e49113. doi: 10.1097/MD.0000000000049113 (PMC13246050; doi:10.1097/MD.0000000000049113)
Supplement: Supplementary file 7 [file medi-105-e49113-s007.docx]

**Supplementary Table 7. Correlation between immune cells and core genes**

| **Data set** | **Gene name** | **Immune cell** | **Correlation coefficient** |
| --- | --- | --- | --- |
| hemodialysis | FUT8 | B.cells.naive | +0.55 |
| hemodialysis | FUT8 | Plasma.cells | -0.66 |
| hemodialysis | FUT8 | Monocytes | -0.73 |
| hemodialysis | CRAT | Plasma.cells | +0.57 |
| hemodialysis | CRAT | Monocytes | +0.53 |
| hemodialysis | SDAD1 | Plasma.cells | -0.55 |
| hemodialysis | MAFG | Plasma.cells | +0.54 |
| hemodialysis | MAFG | Monocytes | +0.63 |
| MDD | CRAT | T.cells.CD4.naive | -0.51 |

MDD: major depressive disorder
