# Supplementary material for: Integrated bioinformatics and machine learning for constructing a diagnostic model of major depressive disorder leveraging shared signatures from hemodialysis: A cross-sectional study
Source: Medicine (Baltimore). 2026 Jun 5;105(23):e49113. doi: 10.1097/MD.0000000000049113 (PMC13246050; doi:10.1097/MD.0000000000049113)
Supplement: Supplementary file 8 [file medi-105-e49113-s008.docx]

****Supplementary Table 8.** miRNAs associated with 6 core genes**

| **Gene ID** | **miRNA ID** | **Gene ID** | **miRNA ID** | **Gene ID** | **miRNA ID** |
| --- | --- | --- | --- | --- | --- |
| BCL7A | hsa-let-7a-5p | BCL7A | hsa-miR-92a-3p | BCL7A | hsa-miR-183-5p |
|  | hsa-let-7b-5p |  | hsa-miR-93-5p |  | hsa-miR-203a-3p |
|  | hsa-let-7c-5p |  | hsa-miR-96-5p |  | hsa-miR-204-5p |
|  | hsa-let-7d-5p |  | hsa-miR-98-5p |  | hsa-miR-210-3p |
|  | hsa-let-7e-5p |  | hsa-miR-29b-3p |  | hsa-miR-211-5p |
|  | hsa-let-7f-5p |  | hsa-miR-103a-3p |  | hsa-miR-212-3p |
|  | hsa-miR-15a-5p |  | hsa-miR-106a-5p |  | hsa-miR-214-3p |
|  | hsa-miR-16-5p |  | hsa-miR-107 |  | hsa-miR-221-3p |
|  | hsa-miR-17-5p |  | hsa-miR-196a-5p |  | hsa-miR-222-3p |
|  | hsa-miR-18a-5p |  | hsa-miR-197-3p |  | hsa-miR-224-5p |
|  | hsa-miR-19a-3p |  | hsa-miR-199a-3p |  | hsa-let-7g-5p |
|  | hsa-miR-19b-3p |  | hsa-miR-129-5p |  | hsa-let-7i-5p |
|  | hsa-miR-20a-5p |  | hsa-miR-148a-3p |  | hsa-miR-1-3p |
|  | hsa-miR-21-5p |  | hsa-miR-30c-5p |  | hsa-miR-15b-5p |
|  | hsa-miR-23a-3p |  | hsa-miR-30d-5p |  | hsa-miR-27b-3p |
|  | hsa-miR-24-3p |  | hsa-miR-139-5p |  | hsa-miR-30b-5p |
|  | hsa-miR-25-3p |  | hsa-miR-7-5p |  | hsa-miR-122-5p |
|  | hsa-miR-27a-3p |  | hsa-miR-10a-5p |  | hsa-miR-124-3p |
|  | hsa-miR-28-5p |  | hsa-miR-10b-5p |  | hsa-miR-128-3p |
|  | hsa-miR-29a-3p |  | hsa-miR-34a-5p |  | hsa-miR-130a-3p |
|  | hsa-miR-30a-5p |  | hsa-miR-181a-5p |  | hsa-miR-133a-3p |
|  | hsa-miR-31-5p |  | hsa-miR-181b-5p |  | hsa-miR-152-3p |
|  | hsa-miR-33a-5p |  | hsa-miR-182-5p |  | hsa-miR-153-3p |
| BCL7A | hsa-miR-191-5p | BCL7A | hsa-miR-335-5p | BCL7A | hsa-miR-623 |
|  | hsa-miR-9-5p |  | hsa-miR-196b-5p |  | hsa-miR-625-5p |
|  | hsa-miR-125a-5p |  | hsa-miR-424-5p |  | hsa-miR-629-3p |
|  | hsa-miR-126-3p |  | hsa-miR-18b-5p |  | hsa-miR-33b-5p |
|  | hsa-miR-146a-5p |  | hsa-miR-20b-5p |  | hsa-miR-660-5p |
|  | hsa-miR-194-5p |  | hsa-miR-452-5p |  | hsa-miR-542-5p |
|  | hsa-miR-195-5p |  | hsa-miR-489-3p |  | hsa-miR-363-5p |
|  | hsa-miR-106b-5p |  | hsa-miR-491-5p |  | hsa-miR-542-3p |
|  | hsa-miR-302a-3p |  | hsa-miR-146b-5p |  | hsa-miR-671-5p |
|  | hsa-miR-301a-3p |  | hsa-miR-493-5p |  | hsa-miR-454-3p |
|  | hsa-miR-99b-5p |  | hsa-miR-519d-3p |  | hsa-miR-765 |
|  | hsa-miR-130b-3p |  | hsa-miR-500a-3p |  | hsa-miR-16-1-3p |
|  | hsa-miR-30e-5p |  | hsa-miR-507 |  | hsa-miR-19b-1-5p |
|  | hsa-miR-30e-3p |  | hsa-miR-18a-3p |  | hsa-miR-22-5p |
|  | hsa-miR-302b-3p |  | hsa-miR-539-5p |  | hsa-miR-29a-5p |
|  | hsa-miR-302c-3p |  | hsa-miR-544a |  | hsa-miR-92a-2-5p |
|  | hsa-miR-302d-3p |  | hsa-miR-92b-3p |  | hsa-miR-30d-3p |
|  | hsa-miR-375 |  | hsa-miR-557 |  | hsa-miR-183-3p |
|  | hsa-miR-378a-5p |  | hsa-miR-579-3p |  | hsa-miR-15b-3p |
|  | hsa-miR-380-5p |  | hsa-miR-548b-3p |  | hsa-miR-30b-3p |
|  | hsa-miR-135b-5p |  | hsa-miR-589-3p |  | hsa-miR-125b-1-3p |
|  | hsa-miR-148b-3p |  | hsa-miR-611 |  | hsa-miR-140-3p |
|  | hsa-miR-324-3p |  | hsa-miR-618 |  | hsa-miR-141-5p |
|  | hsa-miR-6516-5p |  | hsa-miR-7109-5p |  | hsa-miR-6885-5p |
|  | hsa-miR-203a-5p |  | hsa-miR-7113-5p |  | hsa-miR-6887-5p |
| BCL7A | hsa-miR-144-5p | BCL7A | hsa-miR-876-3p | BCL7A | hsa-miR-151b |
|  | hsa-miR-125a-3p |  | hsa-miR-708-5p |  | hsa-miR-2115-5p |
|  | hsa-miR-193a-5p |  | hsa-miR-885-3p |  | hsa-miR-2277-3p |
|  | hsa-miR-106b-3p |  | hsa-miR-877-5p |  | hsa-miR-3125 |
|  | hsa-miR-296-3p |  | hsa-miR-301b-3p |  | hsa-miR-3131 |
|  | hsa-miR-130b-5p |  | hsa-miR-935 |  | hsa-miR-3158-3p |
|  | hsa-miR-362-3p |  | hsa-miR-939-5p |  | hsa-miR-3184-5p |
|  | hsa-miR-330-5p |  | hsa-miR-941 |  | hsa-miR-4326 |
|  | hsa-miR-151a-5p |  | hsa-miR-942-5p |  | hsa-miR-3613-5p |
|  | hsa-miR-135b-3p |  | hsa-miR-944 |  | hsa-miR-3659 |
|  | hsa-miR-148b-5p |  | hsa-miR-1180-3p |  | hsa-miR-3680-3p |
|  | hsa-miR-423-5p |  | hsa-miR-1290 |  | hsa-miR-3691-5p |
|  | hsa-miR-483-5p |  | hsa-miR-548k |  | hsa-miR-3916 |
|  | hsa-miR-486-3p |  | hsa-miR-1293 |  | hsa-miR-4447 |
|  | hsa-miR-193b-5p |  | hsa-miR-1295a |  | hsa-miR-4472 |
|  | hsa-miR-505-5p |  | hsa-miR-1270 |  | hsa-miR-4483 |
|  | hsa-miR-455-3p |  | hsa-miR-1275 |  | hsa-miR-4488 |
|  | hsa-miR-545-5p |  | hsa-miR-302e |  | hsa-miR-4533 |
|  | hsa-miR-576-3p |  | hsa-miR-1292-5p |  | hsa-miR-3117-5p |
|  | hsa-miR-589-5p |  | hsa-miR-1252-5p |  | hsa-miR-4446-5p |
|  | hsa-miR-550a-5p |  | hsa-miR-205-3p |  | hsa-miR-3976 |
|  | hsa-miR-629-5p |  | hsa-miR-224-3p |  | hsa-miR-1343-3p |
|  | hsa-miR-450b-5p |  | hsa-miR-2110 |  | hsa-miR-4697-5p |
|  | hsa-miR-9985 |  | hsa-miR-320a-3p |  | hsa-miR-9500 |
|  | hsa-miR-6758-5p |  | hsa-miR-375-3p |  | hsa-let-7b |
| BCL7A | hsa-miR-203b-5p | BCL7A | hsa-miR-6758-5p | CRAT | hsa-let-7b-5p |
|  | hsa-miR-4738-3p |  | hsa-miR-6766-5p |  | hsa-let-7c-5p |
|  | hsa-miR-4747-5p |  | hsa-miR-6769a-5p |  | hsa-let-7d-5p |
|  | hsa-miR-4755-5p |  | hsa-miR-6795-5p |  | hsa-let-7e-5p |
|  | hsa-miR-1273g-5p |  | hsa-miR-6803-5p |  | hsa-miR-15a-5p |
|  | hsa-miR-5096 |  | hsa-miR-6828-5p |  | hsa-miR-16-5p |
|  | hsa-miR-5006-3p |  | hsa-miR-6846-5p |  | hsa-miR-17-5p |
|  | hsa-miR-5196-5p |  | hsa-miR-6848-5p |  | hsa-miR-24-3p |
|  | hsa-miR-664b-3p |  | hsa-miR-6856-5p |  | hsa-miR-28-5p |
|  | hsa-miR-5581-5p |  | hsa-miR-6859-5p |  | hsa-miR-33a-5p |
|  | hsa-miR-548ax |  | hsa-miR-6769b-5p |  | hsa-miR-96-5p |
|  | hsa-miR-1271-3p | CRAT | hsa-miR-148b-5p |  | hsa-miR-98-5p |
|  | hsa-miR-766-5p |  | hsa-miR-338-5p |  | hsa-miR-103a-3p |
|  | hsa-miR-873-3p |  | hsa-miR-423-5p |  | hsa-miR-106a-5p |
|  | hsa-miR-3184-3p |  | hsa-miR-486-3p |  | hsa-miR-107 |
|  | hsa-miR-758-5p |  | hsa-miR-301b-3p |  | hsa-miR-196a-5p |
|  | hsa-miR-1237-5p |  | hsa-miR-939-5p |  | hsa-miR-147a |
|  | hsa-let-7c-3p |  | hsa-miR-942-5p |  | hsa-miR-7-5p |
|  | hsa-miR-328-5p |  | hsa-miR-151b |  | hsa-miR-34a-5p |
|  | hsa-miR-1251-3p |  | hsa-miR-3619-5p |  | hsa-miR-182-5p |
|  | hsa-miR-6745 |  | hsa-miR-4677-3p |  | hsa-miR-182-3p |
|  | hsa-miR-6748-5p |  | hsa-miR-4793-3p |  | hsa-miR-183-5p |
|  | hsa-miR-6756-5p |  | hsa-miR-151a-5p |  | hsa-let-7g-5p |
|  | hsa-miR-203b-5p |  | hsa-miR-6766-5p |  | hsa-let-7i-5p |
|  | hsa-miR-4738-3p |  | hsa-let-7a-5p |  | hsa-miR-1-3p |
| CRAT | hsa-miR-122-5p | SDAD1 | hsa-miR-548au-5p | SDAD1 | hsa-miR-26a-5p |
|  | hsa-miR-130a-3p |  | hsa-miR-5590-3p |  | hsa-miR-26b-5p |
|  | hsa-miR-9-5p |  | hsa-miR-548av-5p |  | hsa-miR-27a-3p |
|  | hsa-miR-195-5p |  | hsa-miR-766-5p |  | hsa-miR-29a-3p |
|  | hsa-miR-155-5p |  | hsa-miR-1304-3p |  | hsa-miR-30a-5p |
|  | hsa-miR-106b-5p |  | hsa-miR-1277-5p |  | hsa-miR-32-5p |
|  | hsa-miR-130b-3p |  | hsa-miR-1307-5p |  | hsa-miR-33a-5p |
|  | hsa-miR-328-3p |  | hsa-miR-548o-5p |  | hsa-miR-92a-3p |
|  | hsa-miR-326 |  | hsa-miR-548am-5p |  | hsa-miR-93-5p |
|  | hsa-miR-148b-3p |  | hsa-miR-548ay-5p |  | hsa-miR-101-3p |
|  | hsa-miR-335-5p |  | hsa-miR-7161-3p |  | hsa-miR-103a-3p |
|  | hsa-miR-196b-5p |  | hsa-let-7a-5p |  | hsa-miR-106a-5p |
|  | hsa-miR-424-5p |  | hsa-let-7b-5p |  | hsa-miR-107 |
|  | hsa-miR-20b-5p |  | hsa-let-7d-5p |  | hsa-miR-197-3p |
|  | hsa-miR-485-3p |  | hsa-let-7f-5p |  | hsa-miR-30c-5p |
|  | hsa-miR-146b-5p |  | hsa-miR-15a-5p |  | hsa-miR-30d-5p |
|  | hsa-miR-512-3p |  | hsa-miR-16-5p |  | hsa-miR-139-5p |
|  | hsa-miR-532-5p |  | hsa-miR-17-5p |  | hsa-miR-7-5p |
|  | hsa-miR-625-5p |  | hsa-miR-18a-5p |  | hsa-miR-10a-5p |
|  | hsa-miR-454-3p |  | hsa-miR-19a-3p |  | hsa-miR-10b-5p |
|  | hsa-let-7a-3p |  | hsa-miR-19b-3p |  | hsa-miR-34a-5p |
|  | hsa-miR-25-5p |  | hsa-miR-20a-5p |  | hsa-miR-181a-5p |
|  | hsa-miR-221-5p |  | hsa-miR-21-5p |  | hsa-miR-181b-5p |
|  | hsa-miR-140-3p |  | hsa-miR-23a-3p |  | hsa-miR-181c-5p |
| SDAD1 | hsa-miR-8054 |  | hsa-miR-25-3p |  | hsa-miR-182-5p |
| SDAD1 | hsa-miR-182-3p | SDAD1 | hsa-miR-194-5p | SDAD1 | hsa-miR-548c-3p |
|  | hsa-miR-183-5p |  | hsa-miR-106b-5p |  | hsa-miR-33b-5p |
|  | hsa-miR-210-3p |  | hsa-miR-29c-3p |  | hsa-miR-376a-5p |
|  | hsa-miR-181a-3p |  | hsa-miR-30e-5p |  | hsa-miR-671-5p |
|  | hsa-miR-214-3p |  | hsa-miR-30e-3p |  | hsa-let-7f-2-3p |
|  | hsa-miR-216a-5p |  | hsa-miR-361-5p |  | hsa-miR-22-5p |
|  | hsa-miR-221-3p |  | hsa-miR-378a-3p |  | hsa-miR-100-3p |
|  | hsa-let-7g-5p |  | hsa-miR-330-3p |  | hsa-miR-30d-3p |
|  | hsa-let-7i-5p |  | hsa-miR-151a-3p |  | hsa-let-7g-3p |
|  | hsa-miR-1-3p |  | hsa-miR-148b-3p |  | hsa-miR-141-5p |
|  | hsa-miR-15b-5p |  | hsa-miR-324-5p |  | hsa-miR-145-3p |
|  | hsa-miR-23b-3p |  | hsa-miR-422a |  | hsa-miR-129-2-3p |
|  | hsa-miR-27b-3p |  | hsa-miR-424-5p |  | hsa-miR-136-3p |
|  | hsa-miR-30b-5p |  | hsa-miR-18b-5p |  | hsa-miR-30c-1-3p |
|  | hsa-miR-124-3p |  | hsa-miR-20b-5p |  | hsa-miR-130b-5p |
|  | hsa-miR-125b-5p |  | hsa-miR-191-3p |  | hsa-miR-340-5p |
|  | hsa-miR-140-5p |  | hsa-miR-200a-5p |  | hsa-miR-148b-5p |
|  | hsa-miR-142-5p |  | hsa-miR-484 |  | hsa-miR-335-3p |
|  | hsa-miR-142-3p |  | hsa-miR-489-3p |  | hsa-miR-423-5p |
|  | hsa-miR-143-3p |  | hsa-miR-146b-5p |  | hsa-miR-499a-3p |
|  | hsa-miR-191-5p |  | hsa-miR-497-5p |  | hsa-miR-548b-5p |
|  | hsa-miR-125a-5p |  | hsa-miR-181d-5p |  | hsa-miR-589-5p |
|  | hsa-miR-149-5p |  | hsa-miR-520h |  | hsa-miR-548a-5p |
|  | hsa-miR-185-5p |  | hsa-miR-92b-3p |  | hsa-miR-548c-5p |
|  | hsa-miR-186-5p |  | hsa-miR-559 |  | hsa-miR-548d-5p |
| SDAD1 | hsa-miR-450b-5p | SDAD1 | hsa-miR-548ap-5p | MGST1 | hsa-miR-26a-5p |
|  | hsa-miR-876-3p |  | hsa-miR-548aq-5p |  | hsa-miR-26b-5p |
|  | hsa-miR-877-3p |  | hsa-miR-548ar-5p |  | hsa-miR-27a-3p |
|  | hsa-miR-374b-3p |  | hsa-miR-548as-5p |  | hsa-miR-93-5p |
|  | hsa-miR-320b | MGST1 | hsa-miR-561-5p |  | hsa-miR-96-5p |
|  | hsa-miR-320c |  | hsa-miR-6507-5p |  | hsa-miR-103a-3p |
|  | hsa-miR-1271-5p |  | hsa-miR-147b-3p |  | hsa-miR-106a-5p |
|  | hsa-miR-1301-3p |  | hsa-miR-320a-3p |  | hsa-miR-107 |
|  | hsa-miR-548j-5p |  | hsa-miR-151b |  | hsa-miR-148a-3p |
|  | hsa-miR-548k |  | hsa-miR-3605-3p |  | hsa-miR-139-5p |
|  | hsa-miR-548l |  | hsa-miR-3619-5p |  | hsa-miR-7-5p |
|  | hsa-miR-1305 |  | hsa-miR-3662 |  | hsa-miR-34a-5p |
|  | hsa-miR-548h-5p |  | hsa-miR-3934-5p |  | hsa-miR-182-5p |
|  | hsa-miR-548i |  | hsa-miR-4429 |  | hsa-miR-182-3p |
|  | hsa-miR-2115-5p |  | hsa-miR-3074-5p |  | hsa-miR-210-3p |
|  | hsa-miR-3156-5p |  | hsa-miR-197-5p |  | hsa-miR-218-5p |
|  | hsa-miR-548w |  | hsa-let-7a-5p |  | hsa-miR-221-3p |
|  | hsa-miR-378c |  | hsa-let-7b-5p |  | hsa-miR-222-3p |
|  | hsa-miR-3613-5p |  | hsa-let-7c-5p |  | hsa-let-7i-5p |
|  | hsa-miR-23c |  | hsa-miR-16-5p |  | hsa-miR-1-3p |
|  | hsa-miR-548y |  | hsa-miR-17-5p |  | hsa-miR-15b-5p |
|  | hsa-miR-378d |  | hsa-miR-18a-5p |  | hsa-miR-27b-3p |
|  | hsa-miR-548ab |  | hsa-miR-19a-3p |  | hsa-miR-124-3p |
|  | hsa-miR-548ak |  | hsa-miR-19b-3p |  | hsa-miR-128-3p |
|  | hsa-miR-4705 |  | hsa-miR-20a-5p |  | hsa-miR-130a-3p |
| MGST1 | hsa-miR-191-5p | MGST1 | hsa-let-7d-3p | MAFG | hsa-miR-17-5p |
|  | hsa-miR-126-3p |  | hsa-miR-93-3p |  | hsa-miR-17-3p |
|  | hsa-miR-186-5p |  | hsa-miR-30b-3p |  | hsa-miR-18a-5p |
|  | hsa-miR-200c-3p |  | hsa-miR-340-5p |  | hsa-miR-19a-3p |
|  | hsa-miR-155-5p |  | hsa-miR-342-5p |  | hsa-miR-19b-3p |
|  | hsa-miR-106b-5p |  | hsa-miR-335-3p |  | hsa-miR-20a-5p |
|  | hsa-miR-29c-3p |  | hsa-miR-423-5p |  | hsa-miR-21-5p |
|  | hsa-miR-301a-3p |  | hsa-miR-654-3p |  | hsa-miR-22-3p |
|  | hsa-miR-130b-3p |  | hsa-miR-374b-5p |  | hsa-miR-24-3p |
|  | hsa-miR-374a-5p |  | hsa-miR-301b-3p |  | hsa-miR-25-3p |
|  | hsa-miR-379-5p |  | hsa-miR-941 |  | hsa-miR-26b-5p |
|  | hsa-miR-148b-3p |  | hsa-miR-320b |  | hsa-miR-27a-3p |
|  | hsa-miR-424-5p |  | hsa-miR-320c |  | hsa-miR-29a-3p |
|  | hsa-miR-20b-5p |  | hsa-miR-1271-5p |  | hsa-miR-30a-5p |
|  | hsa-miR-429 |  | hsa-miR-1275 |  | hsa-miR-31-5p |
|  | hsa-miR-449a |  | hsa-miR-320d |  | hsa-miR-32-5p |
|  | hsa-miR-92b-3p |  | hsa-miR-2110 |  | hsa-miR-92a-3p |
|  | hsa-miR-579-3p | MAFG | hsa-let-7a-5p |  | hsa-miR-93-5p |
|  | hsa-miR-597-5p |  | hsa-let-7b-5p |  | hsa-miR-96-5p |
|  | hsa-miR-608 |  | hsa-let-7c-5p |  | hsa-miR-98-5p |
|  | hsa-miR-649 |  | hsa-let-7d-5p |  | hsa-miR-100-5p |
|  | hsa-miR-652-3p |  | hsa-let-7e-5p |  | hsa-miR-101-3p |
|  | hsa-miR-376a-5p |  | hsa-let-7f-5p |  | hsa-miR-29b-3p |
|  | hsa-miR-425-5p |  | hsa-miR-15a-5p |  | hsa-miR-103a-3p |
|  | hsa-let-7a-3p |  | hsa-miR-16-5p |  | hsa-miR-106a-5p |
| MAFG | hsa-miR-107 | MAFG | hsa-miR-1-3p | MAFG | hsa-miR-345-5p |
|  | hsa-miR-196a-5p |  | hsa-miR-15b-5p |  | hsa-miR-424-5p |
|  | hsa-miR-197-3p |  | hsa-miR-27b-3p |  | hsa-miR-18b-5p |
|  | hsa-miR-199a-5p |  | hsa-miR-30b-5p |  | hsa-miR-20b-5p |
|  | hsa-miR-148a-3p |  | hsa-miR-122-5p |  | hsa-miR-200a-5p |
|  | hsa-miR-30c-5p |  | hsa-miR-124-3p |  | hsa-miR-484 |
|  | hsa-miR-30d-5p |  | hsa-miR-128-3p |  | hsa-miR-489-3p |
|  | hsa-miR-139-5p |  | hsa-miR-191-5p |  | hsa-miR-491-5p |
|  | hsa-miR-147a |  | hsa-miR-185-5p |  | hsa-miR-146b-5p |
|  | hsa-miR-7-5p |  | hsa-miR-188-5p |  | hsa-miR-494-3p |
|  | hsa-miR-10b-5p |  | hsa-miR-193a-3p |  | hsa-miR-193b-3p |
|  | hsa-miR-34a-5p |  | hsa-miR-194-5p |  | hsa-miR-497-5p |
|  | hsa-miR-181a-5p |  | hsa-miR-195-5p |  | hsa-miR-503-5p |
|  | hsa-miR-181b-5p |  | hsa-miR-155-5p |  | hsa-miR-504-5p |
|  | hsa-miR-182-5p |  | hsa-miR-106b-5p |  | hsa-miR-18a-3p |
|  | hsa-miR-183-5p |  | hsa-miR-29c-3p |  | hsa-miR-455-5p |
|  | hsa-miR-204-5p |  | hsa-miR-130b-3p |  | hsa-miR-92b-3p |
|  | hsa-miR-210-3p |  | hsa-miR-30e-5p |  | hsa-miR-577 |
|  | hsa-miR-211-5p |  | hsa-miR-30e-3p |  | hsa-miR-588 |
|  | hsa-miR-181a-3p |  | hsa-miR-363-3p |  | hsa-miR-592 |
|  | hsa-miR-218-5p |  | hsa-miR-374a-5p |  | hsa-miR-608 |
|  | hsa-miR-221-3p |  | hsa-miR-377-3p |  | hsa-miR-623 |
|  | hsa-miR-224-5p |  | hsa-miR-151a-3p |  | hsa-miR-625-5p |
|  | hsa-let-7g-5p |  | hsa-miR-148b-3p |  | hsa-miR-631 |
|  | hsa-let-7i-5p |  | hsa-miR-338-3p |  | hsa-miR-634 |
| MAFG | hsa-miR-641 | MAFG | hsa-miR-186-3p | MAFG | hsa-miR-2278 |
|  | hsa-miR-652-3p |  | hsa-miR-188-3p |  | hsa-miR-3179 |
|  | hsa-miR-421 |  | hsa-miR-340-5p |  | hsa-miR-3184-5p |
|  | hsa-miR-363-5p |  | hsa-miR-151a-5p |  | hsa-miR-3187-3p |
|  | hsa-miR-671-5p |  | hsa-miR-423-5p |  | hsa-miR-3202 |
|  | hsa-miR-454-3p |  | hsa-miR-483-5p |  | hsa-miR-3065-3p |
|  | hsa-miR-769-5p |  | hsa-miR-486-3p |  | hsa-miR-378c |
|  | hsa-miR-766-3p |  | hsa-miR-574-5p |  | hsa-miR-4271 |
|  | hsa-miR-15a-3p |  | hsa-miR-593-3p |  | hsa-miR-3619-5p |
|  | hsa-miR-24-2-5p |  | hsa-miR-615-5p |  | hsa-miR-3661 |
|  | hsa-miR-25-5p |  | hsa-miR-876-3p |  | hsa-miR-3921 |
|  | hsa-miR-93-3p |  | hsa-miR-744-5p |  | hsa-miR-3928-3p |
|  | hsa-miR-29b-1-5p |  | hsa-miR-873-5p |  | hsa-miR-642b-3p |
|  | hsa-miR-30d-3p |  | hsa-miR-760 |  | hsa-miR-4446-3p |
|  | hsa-miR-10a-3p |  | hsa-miR-301b-3p |  | hsa-miR-4454 |
|  | hsa-miR-10b-3p |  | hsa-miR-940 |  | hsa-miR-4539 |
|  | hsa-miR-34a-3p |  | hsa-miR-522-5p |  | hsa-miR-3074-5p |
|  | hsa-let-7g-3p |  | hsa-miR-320c |  | hsa-miR-3157-3p |
|  | hsa-let-7i-3p |  | hsa-miR-1301-3p |  | hsa-miR-3177-5p |
|  | hsa-miR-15b-3p |  | hsa-miR-548k |  | hsa-miR-4651 |
|  | hsa-miR-30b-3p |  | hsa-miR-1908-5p |  | hsa-miR-4653-5p |
|  | hsa-miR-127-5p |  | hsa-miR-1914-3p |  | hsa-miR-4656 |
|  | hsa-miR-129-2-3p |  | hsa-let-7a-2-3p |  | hsa-miR-4659a-3p |
|  | hsa-miR-146a-3p |  | hsa-miR-151b |  | hsa-miR-4660 |
|  | hsa-miR-185-3p |  | hsa-miR-2114-5p |  | hsa-miR-4659b-3p |
| MAFG | hsa-miR-4685-5p | MAFG | hsa-miR-6780b-5p | FUT8 | hsa-miR-23a-3p |
|  | hsa-miR-203b-5p |  | hsa-miR-6836-5p |  | hsa-miR-25-3p |
|  | hsa-miR-4725-3p |  | hsa-miR-6837-5p |  | hsa-miR-26a-5p |
|  | hsa-miR-4726-3p |  | hsa-miR-6875-3p |  | hsa-miR-26b-5p |
|  | hsa-miR-4747-5p |  | hsa-miR-6880-5p |  | hsa-miR-27a-3p |
|  | hsa-miR-4755-5p |  | hsa-miR-6886-5p |  | hsa-miR-28-5p |
|  | hsa-miR-5006-3p |  | hsa-miR-7113-5p |  | hsa-miR-29a-3p |
|  | hsa-miR-548ap-3p |  | hsa-miR-301b-5p |  | hsa-miR-31-5p |
|  | hsa-miR-5194 |  | hsa-miR-218 |  | hsa-miR-32-5p |
|  | hsa-miR-5196-5p |  | hsa-miR-320a-3p |  | hsa-miR-33a-5p |
|  | hsa-miR-5584-3p | FUT8 | hsa-let-7a-5p |  | hsa-miR-92a-3p |
|  | hsa-miR-212-5p |  | hsa-let-7b-5p |  | hsa-miR-93-5p |
|  | hsa-miR-301a-5p |  | hsa-let-7c-5p |  | hsa-miR-98-5p |
|  | hsa-miR-660-3p |  | hsa-let-7d-5p |  | hsa-miR-101-3p |
|  | hsa-miR-766-5p |  | hsa-let-7e-5p |  | hsa-miR-29b-3p |
|  | hsa-miR-873-3p |  | hsa-let-7f-5p |  | hsa-miR-106a-5p |
|  | hsa-miR-1306-5p |  | hsa-miR-15a-5p |  | hsa-miR-196a-5p |
|  | hsa-miR-6077 |  | hsa-miR-16-5p |  | hsa-miR-139-5p |
|  | hsa-miR-6132 |  | hsa-miR-17-5p |  | hsa-miR-7-5p |
|  | hsa-miR-504-3p |  | hsa-miR-17-3p |  | hsa-miR-10b-5p |
|  | hsa-miR-6738-5p |  | hsa-miR-19a-3p |  | hsa-miR-34a-5p |
|  | hsa-miR-6745 |  | hsa-miR-19b-3p |  | hsa-miR-181a-5p |
|  | hsa-miR-6782-5p |  | hsa-miR-20a-5p |  | hsa-miR-182-5p |
|  | hsa-miR-6783-5p |  | hsa-miR-21-5p |  | hsa-miR-183-5p |
|  | hsa-miR-6832-3p |  | hsa-miR-22-3p |  | hsa-miR-203a-3p |
| FUT8 | hsa-miR-210-3p | FUT8 | hsa-miR-155-5p | FUT8 | hsa-miR-550a-3p |
|  | hsa-miR-218-5p |  | hsa-miR-106b-5p |  | hsa-miR-597-5p |
|  | hsa-miR-221-3p |  | hsa-miR-29c-3p |  | hsa-miR-33b-5p |
|  | hsa-miR-222-3p |  | hsa-miR-296-5p |  | hsa-miR-652-3p |
|  | hsa-miR-200b-3p |  | hsa-miR-363-3p |  | hsa-miR-449b-5p |
|  | hsa-let-7g-5p |  | hsa-miR-374a-5p |  | hsa-miR-542-5p |
|  | hsa-let-7i-5p |  | hsa-miR-375 |  | hsa-miR-376a-5p |
|  | hsa-miR-1-3p |  | hsa-miR-379-5p |  | hsa-miR-671-5p |
|  | hsa-miR-23b-3p |  | hsa-miR-340-3p |  | hsa-miR-767-5p |
|  | hsa-miR-27b-3p |  | hsa-miR-342-3p |  | hsa-miR-454-3p |
|  | hsa-miR-122-5p |  | hsa-miR-337-3p |  | hsa-let-7a-3p |
|  | hsa-miR-124-3p |  | hsa-miR-135b-5p |  | hsa-let-7d-3p |
|  | hsa-miR-128-3p |  | hsa-miR-424-5p |  | hsa-let-7f-2-3p |
|  | hsa-miR-132-3p |  | hsa-miR-425-3p |  | hsa-miR-20a-3p |
|  | hsa-miR-138-5p |  | hsa-miR-20b-5p |  | hsa-miR-21-3p |
|  | hsa-miR-142-5p |  | hsa-miR-449a |  | hsa-miR-23a-5p |
|  | hsa-miR-145-5p |  | hsa-miR-433-3p |  | hsa-miR-29a-5p |
|  | hsa-miR-191-5p |  | hsa-miR-329-3p |  | hsa-miR-101-5p |
|  | hsa-miR-9-5p |  | hsa-miR-432-5p |  | hsa-miR-192-3p |
|  | hsa-miR-126-5p |  | hsa-miR-517a-3p |  | hsa-miR-30d-3p |
|  | hsa-miR-126-3p |  | hsa-miR-500a-3p |  | hsa-miR-10a-3p |
|  | hsa-miR-146a-5p |  | hsa-miR-92b-3p |  | hsa-miR-221-5p |
|  | hsa-miR-185-5p |  | hsa-miR-570-3p |  | hsa-miR-222-5p |
|  | hsa-miR-186-5p |  | hsa-miR-574-3p |  | hsa-miR-30b-3p |
|  | hsa-miR-194-5p |  | hsa-miR-582-5p |  | hsa-miR-138-2-3p |
| FUT8 | hsa-miR-129-2-3p | FUT8 | hsa-miR-224-3p | FUT8 | hsa-miR-147b-3p |
|  | hsa-miR-195-3p |  | hsa-miR-3200-3p |  | hsa-miR-1843 |
|  | hsa-miR-30c-1-3p |  | hsa-miR-4254 |  | hsa-miR-190b-5p |
|  | hsa-miR-219a-2-3p |  | hsa-miR-3613-5p |  | hsa-miR-320a-3p |
|  | hsa-miR-362-3p |  | hsa-miR-4429 |  |  |
|  | hsa-miR-340-5p |  | hsa-miR-4446-3p |  |  |
|  | hsa-miR-342-5p |  | hsa-miR-4664-5p |  |  |
|  | hsa-miR-135b-3p |  | hsa-miR-4677-3p |  |  |
|  | hsa-miR-148b-5p |  | hsa-miR-1343-3p |  |  |
|  | hsa-miR-423-5p |  | hsa-miR-203b-5p |  |  |
|  | hsa-miR-424-3p |  | hsa-miR-5100 |  |  |
|  | hsa-miR-455-3p |  | hsa-miR-664b-3p |  |  |
|  | hsa-miR-582-3p |  | hsa-miR-197-5p |  |  |
|  | hsa-miR-876-3p |  | hsa-miR-561-5p |  |  |
|  | hsa-miR-708-5p |  | hsa-miR-660-3p |  |  |
|  | hsa-miR-744-5p |  | hsa-miR-766-5p |  |  |
|  | hsa-miR-877-5p |  | hsa-miR-873-3p |  |  |
|  | hsa-miR-873-5p |  | hsa-miR-381-5p |  |  |
|  | hsa-miR-522-5p |  | hsa-miR-370-5p |  |  |
|  | hsa-miR-1225-5p |  | hsa-miR-889-5p |  |  |
|  | hsa-miR-320c |  | hsa-miR-7843-5p |  |  |
|  | hsa-miR-1301-3p |  | hsa-miR-450a-2-3p |  |  |
|  | hsa-miR-1266-5p |  | hsa-miR-7977 |  |  |
|  | hsa-miR-1910-5p |  | hsa-miR-181b-2-3p |  |  |
|  | hsa-miR-103a-2-5p |  | hsa-miR-10399-3p |  |  |
